# Simplified Modular Access to Enantiopure 1,2-Aminoalcohols via Ni-Electrocatalytic Decarboxylative Arylation

**DOI:** 10.1021/jacs.3c14119

**Published:** 2024-02-22

**Authors:** Jiawei Sun, Hirofumi Endo, Megan A. Emmanuel, Martins S. Oderinde, Yu Kawamata, Phil S. Baran

**Affiliations:** † Department of Chemistry, Scripps Research, 10550 North Torrey Pines Road, La Jolla, California 92037, United States; ‡ Chemical Process Development, Bristol Myers Squibb, 1 Squibb Drive, New Brunswick, New Jersey 08901, United States; § Small Molecule Drug Discovery, Bristol Myers Squibb Research & Early Development, Route 206 & Province Line Road, Princeton, New Jersey 08543, United States

## Abstract

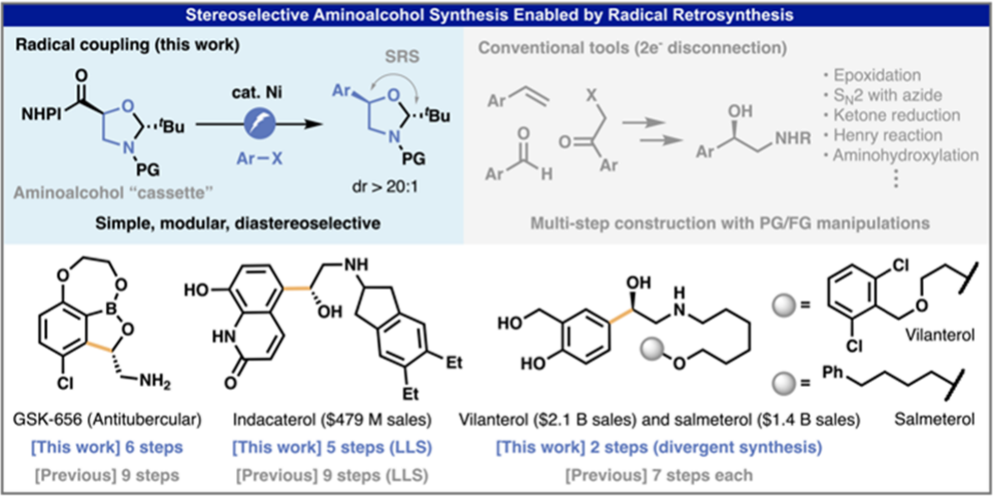

Chiral aminoalcohols are omnipresent in bioactive compounds. Conventional strategies to access this motif involve multiple-step reactions to install the requisite functionalities stereoselectively using conventional polar bond analysis. This study reveals that a simple chiral oxazolidine-based carboxylic acid can be readily transformed to substituted chiral aminoalcohols with high stereochemical control by Ni-electrocatalytic decarboxylative arylation. This general, robust, and scalable coupling can be used to synthesize a variety of medicinally important compounds, avoiding protecting and functional group manipulations, thereby dramatically simplifying their preparation.

## Introduction

**Figure 1:**
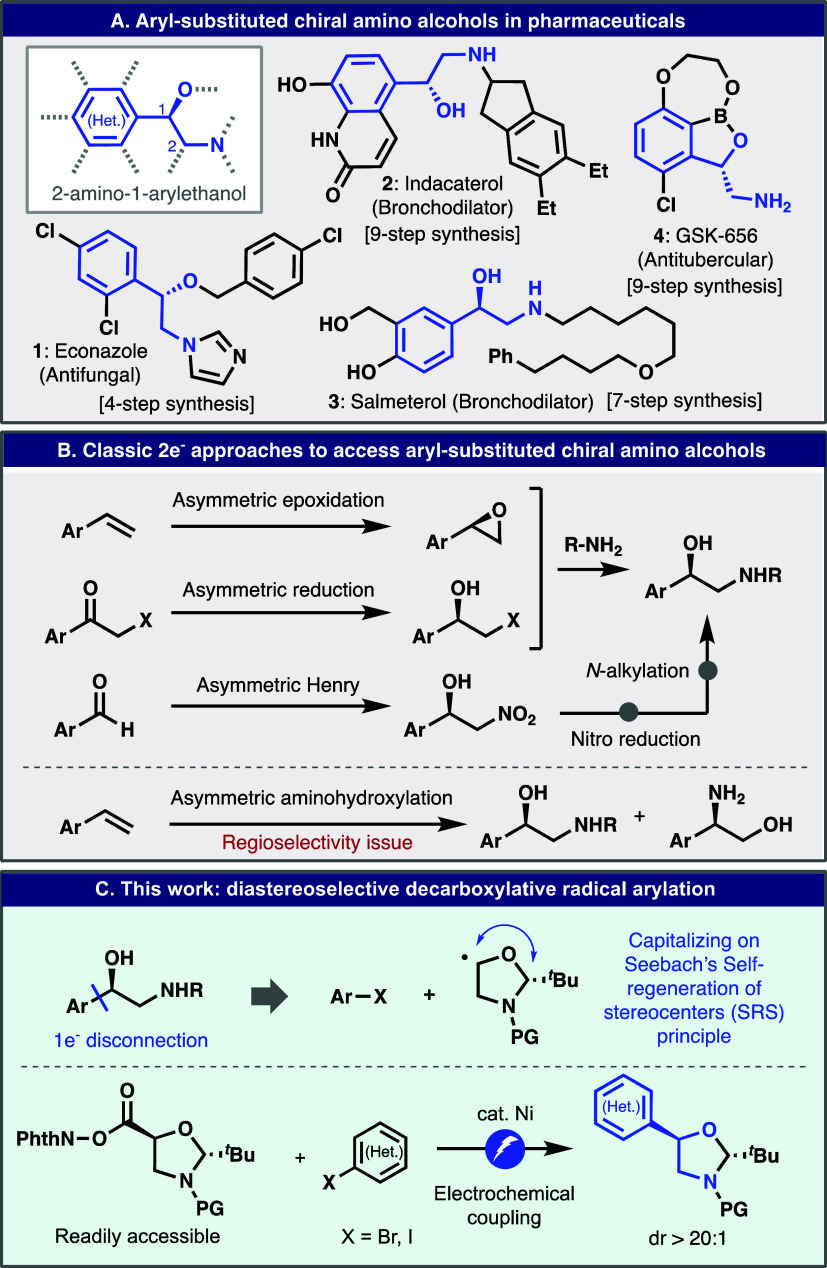
Utility of aryl-substituted chiral aminoalcohols and their synthesis via polar- and radical-based strategies. (A) Chiral aminoalcohols are a privileged structural motif for bioactive molecules. (B) Mainstream methods for preparing substituted aminoalcohols exclusively rely on polar (2e^–^) disconnections. (C) Radical (1e^–^) disconnection enables access to chiral aminoalcohols via modular cross-coupling, where the stereochemistry of the new C–C bond is controlled by SRS.

Enantiopure aminoalcohols are ubiquitous in natural products, active pharmaceutical ingredients (APIs), and agrochemicals. The 2-amino-1-arylethanol unit, in particular, is frequently encountered (Figure 1A).^1−4^ For example, econazole (**1**) is widely used as an antifungal medication;^5,6^ indacaterol (**2**) and salmeterol (**3**) are effective bronchodilators and enlisted as top-selling small-molecule drugs;^7^ and a unique boron-containing molecule GSK-656 (**4**) is a promising antituberculosis drug with a new mechanism of action.^8,9^ Synthetic approaches to molecules of this sort generally rely on a deliberate construction of the aminoalcohol in a stepwise fashion rather than a modular installation through cross-coupling.^1−3^ Indeed, constructing the chiral aminoalcohol motifs in **1**–**4** requires multiple steps, all of which are reliant on polar bond retrosynthetic analysis (Figure 1B). Thus, asymmetric epoxidation, asymmetric ketone reduction followed by S_N_2 with a nitrogen-based nucleophile, and an asymmetric Henry reaction followed by hydrogenation of the nitro group are the go-to transformations to access such structures. Although Sharpless asymmetric aminohydroxylation enables the single-step construction of chiral aminoalcohols from styrene,^10,11^ it can be complicated by regioisomeric impurities^12^ and requires expensive and toxic osmium catalysts. The aforementioned reliance on polar bond disconnections (2e^–^ logic) necessitates precise choreography of protecting/functional group manipulations.

This study builds on the pioneering work of Seebach and co-workers who employed oxazolidine-based auxiliaries through the principle of “self-regeneration of stereocenters” (SRS, Figure 1C).^13^ In SRS, simple amino acid feedstocks are protected at a distal site with high diastereoselectivity. Subsequent reactions (both radical and polar bond formations) at the C-terminus generally take place with near-complete stereocontrol to “regenerate” the original stereocenter in a predictable way. The SRS approach has been applied in numerous contexts over the years,^13,14^ although its use in radical chemistry has seen only limited applications. Indeed, several examples of intramolecular radical C–C bond formation have been reported.^15,16^ Intermolecular C–C bond formations in this context are all reliant on Giese-type additions^17−19^ to electron-deficient olefins such as Inoue’s acyltellurium studies.^20^ To our knowledge, the use of Seebach-type SRS in transition metal-catalyzed radical cross-coupling has not been disclosed.^21^ Meanwhile, radical retrosynthesis has been demonstrated in a variety of contexts to achieve more intuitive, perhaps even “LEGO”-like modular approaches to synthesis.^22−25^ This article discloses how the principle of SRS can be leveraged in the union of inexpensive isoserine-derived redox-active esters (RAEs) to serve as convenient “cassettes” for the reliable and facile construction of chiral aminoalcohols via Ni-electrocatalytic decarboxylative coupling. As documented herein, this reaction manifold is applicable in both the early and late stages of drug/agrochemical discovery due to its inherent modularity and robust scalability.

## Results and Discussion

**Figure 2:**
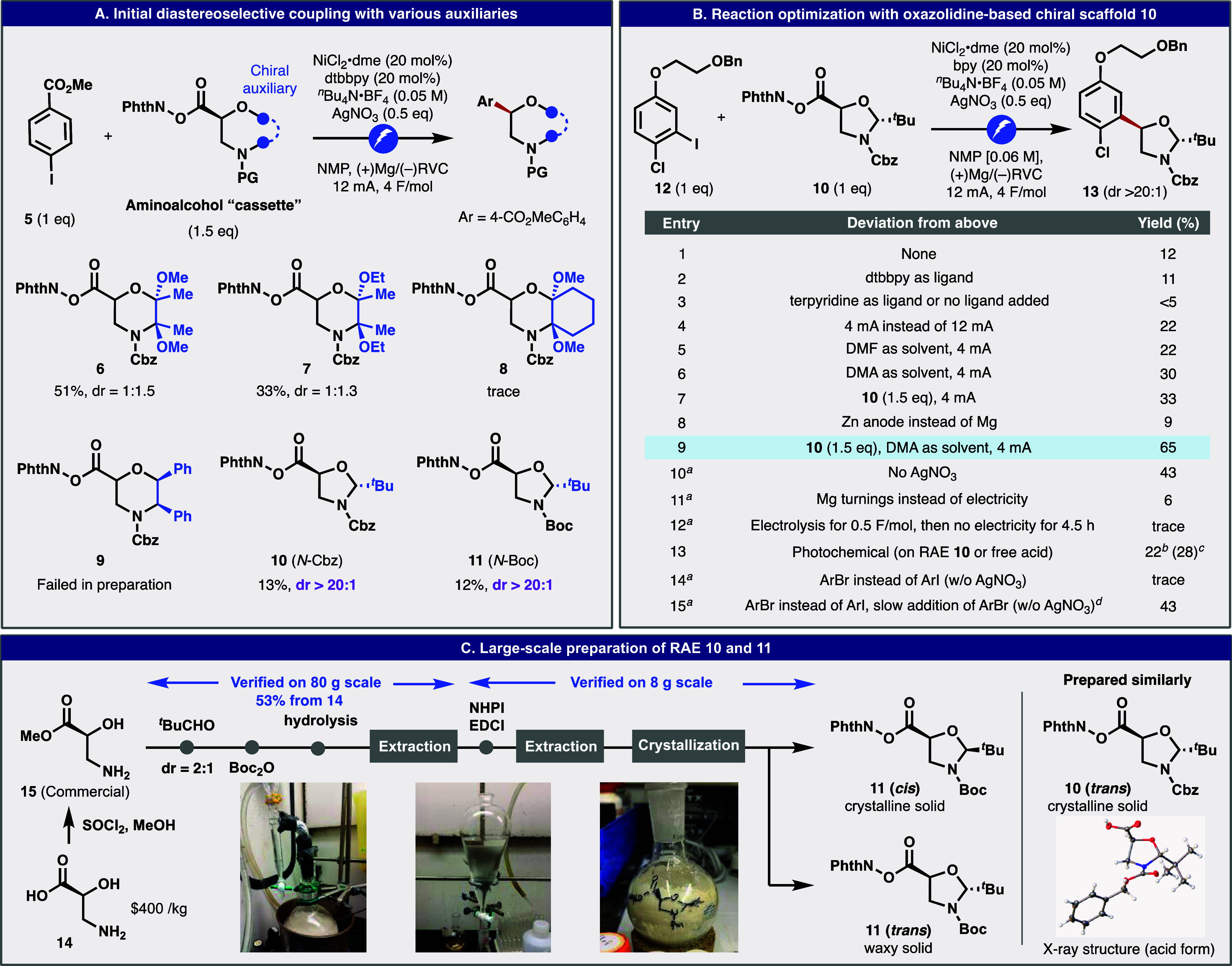
Development of the key aminoalcohol coupling unit and reaction optimization. (A) Initial exploration of various chiral auxiliaries revealed that oxazolidine is uniquely effective for highly diastereoselective decarboxylative arylation. (B) Reaction optimization and control experiments. (C) Practical preparation of oxazolidine-based RAEs. ^a^The reaction was performed using the conditions of entry 9. ^b^Photochemical conditions were based on the free acid starting material (see the Supporting Information for the full conditions). ^c^Photochemical conditions using **RAE 10** as the starting material (see the Supporting Information for the full conditions). ^d^ArBr was added over 100 min, such that the addition was finished slightly before the completion of electrolysis.

The pursuit of a reliable means to access the 2-amino-1-arylethanol unit in high enantiopurity via modular cross-coupling was built off of prior studies from this lab, specifically, the recently disclosed electrochemical decarboxylative alkenylation/arylation uniquely promoted by Ag nanoparticles (AgNPs).^26,27^ Since Csp^2^–Csp^3^ bonds are ubiquitous across natural products and pharmacophores, this transformation is highly useful for the rapid and modular construction of carbon skeletons from readily available carboxylic acids and alkynyl/aryl halides. The feasibility of controlling the stereochemistry in this radical-based cross-coupling was supported by the recent disclosure of second-generation doubly decarboxylative coupling, where careful selection of building block structures as well as reaction conditions rendered the alkyl–alkyl bond formation highly diastereoselective.^28^ Notably, Ley auxiliary-based RAE **6** (Figure 2A) was used for the highly stereoselective synthesis of *ent*-SF2768 and complanine, which set the stage for our exploration in the context of diastereoselective arylation. Initial forays were directed at identifying an inexpensive aminoalcohol “cassette” that could lead to high dr and conversion. Numerous constructs based on Ley’s auxiliary were evaluated such as **6**–**9** in the cross-coupling with aryl iodide **5**. Unfortunately, the observed dr (**6** and **7**) or yield (**8**) was too low, or the requisite RAE could not be easily prepared (**9**). Extensive ligand screening to improve the diastereoselectivity was fruitless, although ligand structures seemed to modestly affect the diastereoselectivity (see the Supporting Information for details). The promising leads emerged when exploring Seebach oxazolidines such as **10** wherein high dr was observed, albeit in low yield. Changing the nitrogen protecting group to Boc (**11**) maintained high diastereoselectivity, confirming robust stereochemical control regardless of the steric bulk of the protecting group.

Based on this observation, extensive optimization was conducted, as outlined in Figure 2B between RAE **10** and aryl iodide **12**. The latter was chosen for an eventual application in the synthesis of GSK-656 (**4**, Figure 1). The organometallic and electrochemical parameters were thus explored in a systematic fashion (for a more comprehensive summary, see the Supporting Information). For instance, the use of simple bipyridine (bpy) as the ligand resulted in the highest yield of all ligands screened (entry 1). The absence of ligands or the use of tridentate ligands such as terpyridine shut down the reaction (entry 3). Reducing the current from 12 to 4 mA doubled the observed yield (entry 4). A further improvement was observed after solvent screening, with DMA emerging as the best (entry 5–6). Increasing the loading of RAE **10**–1.5 equiv was also beneficial (entry 7), presumably due to the preferential consumption of **10** over ArI. A Mg sacrificial anode proved to be crucial (entry 8). The optimum conditions emerged by combining these observations (entry 9). Control studies showed that in this coupling, Ag is not crucial but improved the yield moderately (entry 10). This effect can be ascribed to the suppression of RAE degradation on the cathode by deposited AgNPs.^26^ To rule out the in situ generation of a Grignard reagent, purely chemical conditions using activated Mg turnings (entry 11) and the reaction progress on an electrochemically activated Mg surface (entry 12) were evaluated. Drastically reduced yields in both entries confirmed that Mg itself is insufficient to facilitate the reaction. The reaction was also benchmarked against photochemical conditions by using both RAE^29^ and the corresponding free carboxylic acid^30,31^ as a substrate (entry 13), confirming that the electrochemical method described here offers much simpler reaction conditions, an important aspect for large-scale execution (vide infra). Finally, under the optimized conditions, the corresponding aryl bromide poorly reacted, resulting in a low yield of **13** due to the preferential consumption of RAE **10** (entry 14). This reactivity difference was overcome by the slow addition of RAE **10** via a syringe pump, furnishing the product in the identical yield that was obtained by using ArI (comparing entry 10 and entry 15).^32^

After identifying the practical aminoalcohol “cassettes” **10** and **11** and the requisite optimal cross-coupling conditions, their practical and scalable synthesis was pursued. The analogous oxazolidine synthesis described by Schmidt^33^ and Li^34^ was modified to improve yields and operational simplicity by minimizing chromatography. The synthesis can be readily achieved, as depicted in Figure 2C, by using inexpensive (*S*)-isoserine as a starting material ($ 0.4/g,^35^ the cost per mol is even less than a bulk chemical PPh_3_) after a sequence of trivial interconversions such as esterification, condensation with pivalaldehyde, and *N*-protection followed by hydrolysis of the ester. This simple sequence can be accomplished by a single chemist within several days on an 80 g-scale to deliver the parent carboxylic acid for **11** in >50% overall yield from isoserine **14** without column chromatography. The subsequent RAE formation was facile and clean (8 g-scale). Fortunately, a large difference in the crystallinity provided a simple way to separate the diastereomers at this stage. Both diastereomers are a useful building block to access both enantiomers of an aminoalcohol. Analogous RAE **10** can also be prepared by a similar procedure. The stereochemistry of RAE *trans-***10** was unambiguously determined by X-ray analysis of the parent carboxylic acid.

With a general set of conditions and optimized access to RAEs **10** and **11** in hand, the scope of this methodology was evaluated across a range of aryl iodides (and an aryl bromide), as shown in Table 1. Many functional groups that would be problematic in conventional cross-couplings are well tolerated in this transformation. For instance, ortho-substituted arenes do not diminish reactivity (**17b**, **17d**, **17e**, **17o**, **18d**). Boronic ester and halide-containing arenes can be employed (**17c**, **17f**, **17o**, **17p**, **18c**, **18d**). Reducible functionality such as free aldehydes **17i** and **18b** or nitrile **17e** can be employed. The presence of sulfur atoms does not inhibit the reaction (**17d**, **17m**, **17n**). Easily oxidizable electron-rich arenes remain unscathed in this coupling (**17h**, **17q**, **18a**). Of note, highly oxidatively sensitive motifs such as free phenols and anilines participate smoothly (**17g**, **18b**, **18c**). Finally, a range of Lewis-basic heterocycles can be easily coupled (**17j**, **17k**, **17l**, **17o**, **17p**). This electrocatalytic method is uniformly superior to state-of-the-art photocatalytic conditions, as benchmarked on substrates **17a**, **17c**, **17d**, **17e**, **17j**, **17l**, and **17q**. An electron-deficient aryl bromide (**18c**) was also employed to demonstrate satisfactory coupling efficiency.

**Table 1:**
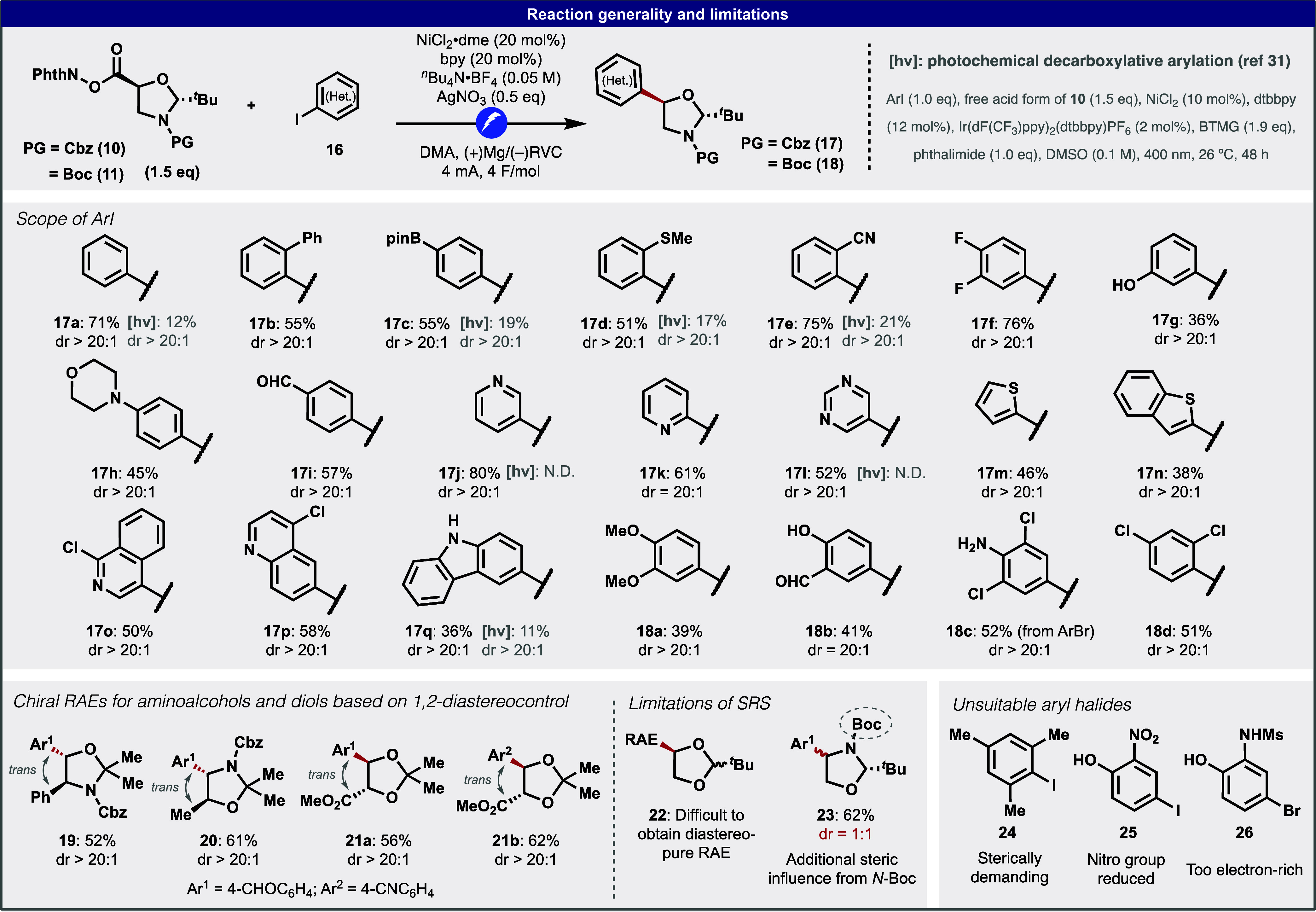
Reaction Generality and Limitations

The strategy outlined herein is also applicable to other chiral scaffolds based on α-heteroatom-substituted acids, as exemplified with substrates **19**–**21**. In these cases, 1,2-stereocontrol (rather than SRS) leads to uniformly high dr in the cross-coupling. Thus, it opens the door to a limitless range of structures containing aminoalcohols and chiral diols without recourse to conventional methods that lack modularity (chiral epoxide opening, aminohydroxylation, and dihydroxylation).^36^ This chemistry is easily scaled up, as will be discussed in the next section. With regards to limitations, cyclic acetal **22** could not be easily obtained as a single diastereomer. In accord with Seebach’s studies, RAE **23**, a regioisomeric variant of RAEs **10** and **11**, led to low dr in the cross-coupling, presumably because the neighboring N-Boc group affects the ring conformation.^37^ Finally, 2,6-disubstitution (**24**), nitro groups (**25**), and substrates that were extremely electron-donating (**26**) represent limitations of the aryl donor.

**Figure 3:**
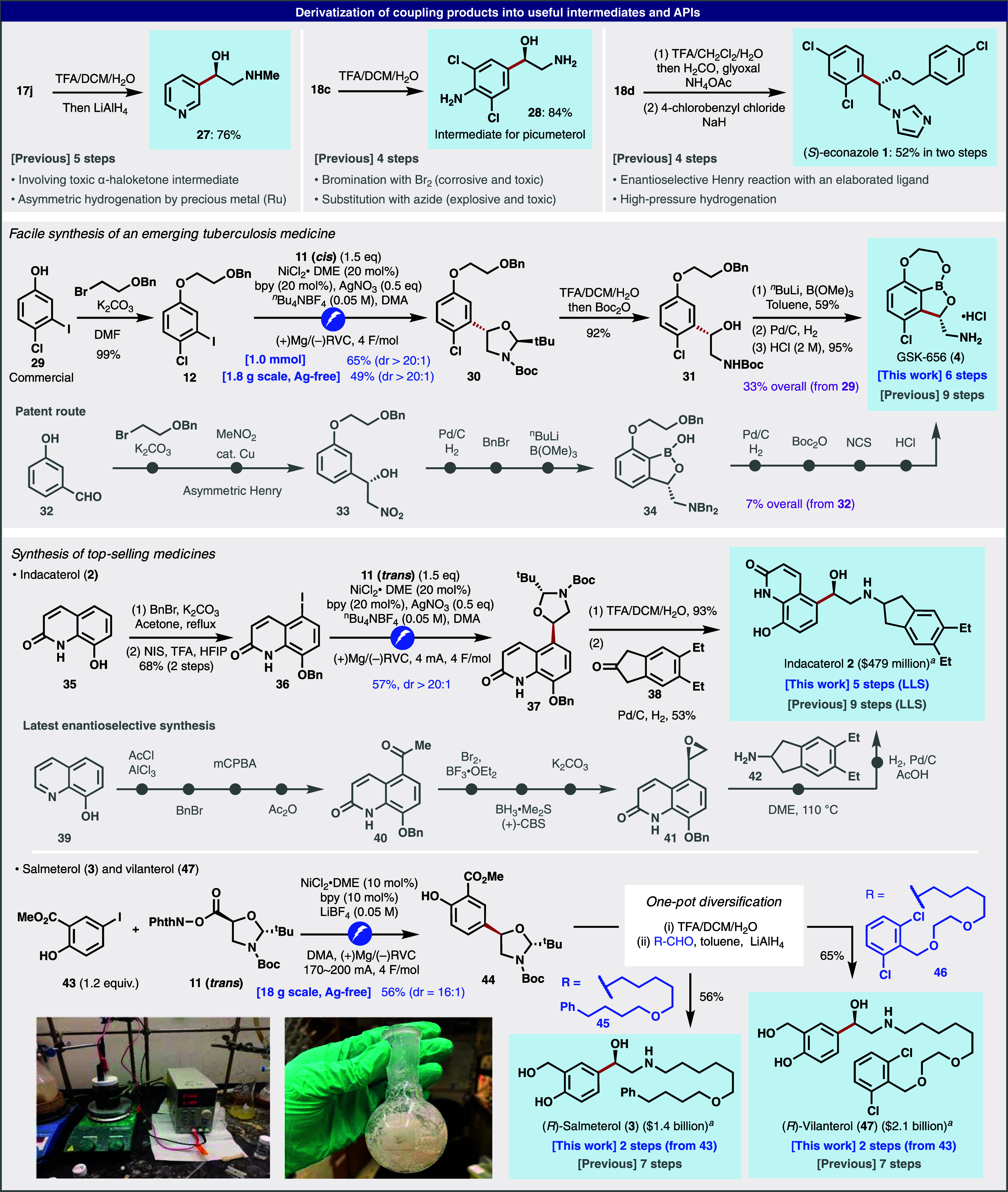
Preparation of useful intermediates and APIs via decarboxylative arylation. ^a^From ref (7).

Radical retrosynthetic logic has now been shown on numerous occasions to simplify synthetic routes.^22,23^ Similarly, the radical cross-coupling approach delineated herein can be leveraged to procure chiral aminoalcohol-containing structures that previously required tedious routes guided by polar bond analysis. At a high level, the strategic advantage exemplified with this approach involves the modular attachment of the aminoalcohol motif stereoselectively, rather than its stepwise construction. As a result, the current approach provides a much simpler and more intuitive avenue. For example, the simple derivatization of the selected coupling products shown in Figure 3 led to medicinally useful building blocks (**27**, **28**) or a marketed drug (**1**). Previous routes to synthesize these compounds are much longer, involving numerous reactions that are undesired from both safety (toxic intermediates, hazardous/explosive reagents, high-pressure reaction) and sustainability (precious metal-based hydrogenation) perspectives.^38−40^ In some cases, the enantioselective step requires Ru-based catalysts (Noyori reduction for **27**)^40^ or complex thiourea catalysts (asymmetric Henry reaction for **1**).^39^ The Ni-electrocatalytic approach can now offer new access to an emerging tuberculosis medicine, GSK-656 (**4**). Thus, the unique boron-containing drug candidate exhibits a highly selective inhibitory activity to *Mycobacterium tuberculosis* leucyl-tRNA synthetase (LeuRS) and is currently in Phase II clinical trials as a promising candidate for multidrug-resistant tuberculosis.^8,9^ The current most practical synthesis involves a 9-step route using an asymmetric Cu-catalyzed Henry reaction as a key step for the construction of the aminoalcohol motif.^41^ Although the route is optimized and scalable, multiple Pd-based hydrogenation steps and redox manipulations reduce ideality. In contrast, the Ni-electrocatalytic approach enables straightforward access to **4** by simply coupling the aminoalcohol unit into aryl iodide **12**, followed by boron installation and protecting group removal. Notably, the overall yield was considerably improved (33% compared to 7% in the previous route). This particular coupling (**12** + **11**) was easily performed on gram scale without the Ag additive, albeit in a slightly diminished yield, demonstrating the robustness of the electrochemical coupling. Finally, the Ni-electrocatalytic approach can provide a new route to well-established drugs that have a large market size.^7^ For example, indacaterol **2** is a long-acting β-adrenoceptor agonist developed by Novartis.^42^ Its enantioselective synthesis involves the laborious construction of the chiral aminoalcohol motif from 8-hydroxyquinoline **39**, resulting in a 9-step synthesis (the longest linear sequence).^42−44^ Instead, by just “attaching” the key aminoalcohol motif **11** to readily accessible heteroaryl iodide **36**, the synthesis was considerably truncated to 5 steps. The routes to salmeterol **3**(45) and vilanterol **47**,^46^ widely used bronchodilators, can be similarly simplified. Due to their structural similarity, a divergent synthesis of these two top-selling drugs was envisioned using **44** as a common intermediate. The key Ni-electrocatalytic coupling of free phenol **43** with **11** was performed on decagram scale (18 g) without Ag to demonstrate the practicality of this approach. With ample supplies of **44** in hand, the trivial disposal of the hemiaminal and Boc groups (TFA) followed by reductive amination (conveniently performed in one pot) successfully furnished **3** and **47** in merely two steps from the inexpensive precursor **43**.

## Conclusions

In this study, modular and stereocontrolled access to a variety of substituted chiral aminoalcohols was developed by leveraging the power of Ni-electrocatalytic decarboxylative coupling. A simple, isoserine-derived oxazolidine was identified as a useful template to enable the highly diastereoselective installation of an aminoalcohol unit. The high stereochemical fidelity is based on Seebach’s SRS principle, which is an underutilized strategy in the context of stereocontrolled radical cross-coupling. The reaction allows for the coupling of a variety of (hetero)aryl halides and tolerates functional groups that are problematic for cross-coupling in general such as free phenols and anilines. The reaction is robust and scalable, which is evident in the success of gram-scale couplings for compounds **30** and **44**. In addition, omission of the Ag additive on scale further improves the practicality and reduces the heterogeneity of reaction conditions. The utility of the reaction is illustrated in the syntheses of 7 useful intermediates or drugs, 4 of which are highly important drugs (GSK-656: emerging multidrug-resistant tuberculosis; indacaterol/salmeterol/vilanterol: >hundreds of million $ in sales). Of note, the routes developed in this work are considerably more concise than their latest process routes due to completely different disconnections enabled by modular Ni-electrocatalytic coupling and radical retrosynthetic logic. Such 1e^–^ disconnections that are polarity agnostic enable the modular “attachment” of an aminoalcohol unit rather than tedious construction via canonical 2e^–^ reactions such as epoxidation, ketone reduction, and carbonyl-based C–C bond formations, which are invariably accompanied by protecting/functional group/redox manipulations. This work adds to the growing body of literature demonstrating the value of stereocontrolled radical coupling to simplify synthesis.^47−50^

## Supporting Information Available

Experimental procedures; additional experimental data; NMR characterization data; and X-ray characterization detail (PDF)
