# Supplementary material for: Simplified Modular Access to Enantiopure 1,2-Aminoalcohols via Ni-Electrocatalytic Decarboxylative Arylation
Source: J Am Chem Soc. 2024 Feb 22;146(9):6209–16. doi: 10.1021/jacs.3c14119 (PMC10962872; doi:10.1021/jacs.3c14119)
Supplement: Supplementary file 1 — ja3c14119_si_001.pdf [file ja3c14119_si_001.pdf]

Supporting Information

Simplified Modular Access to Enantiopure 1,2-Aminoalcohols via Ni-Electrocatalytic Decarboxylative Arylation

Jiawei Sun,[1] Hirofumi Endo,[1] Megan A. Emmanuel,[2] Martins S. Oderinde,[3] Yu Kawamata,*[1] Phil S. Baran*[1]

[1] Department of Chemistry, Scripps Research, 10550 North Torrey Pines Road, La Jolla, CA, 92037, United States.

[2] Chemical Process Development, Bristol Myers Squibb, 1 Squibb Drive, New Brunswick, NJ, 08901, United States.

[3] Small Molecule Drug Discovery, Bristol Myers Squibb Research & Early Development, Route 206 & Province Line Road, Princeton, NJ, 08543, United States.

*E-mail: yukawama@scripps.edu, pbaran@scripps.edu

Table of Contents

General Experimental 3

General Procedure A: Ni-Electrocatalytic Diastereoselective Decarboxylative Arylation with Aryl Iodide as Coupling Partner 4

Graphical Guide for Ni-Electrocatalytic Diastereoselective Decarboxylative Arylation with Aryl Iodide as Coupling Partner 5

General procedure B: Ni-Electrocatalytic Diastereoselective Decarboxylative Arylation with Aryl Bromide as Coupling Partner (Slow Addition) 8

Graphical Guide for Ni-Electrocatalytic Diastereoselective Decarboxylative Arylation with Aryl Bromide as Coupling Partner (Slow Addition) 9

Optimization of Reaction Parameters for the Ni-Electrocatalytic Diastereoselective Decarboxylative Arylation 13

Optimization for Gram-Scale Electrochemical Coupling of GSK-656 Intermediate (no $AgNO_3$ Condition) 17

Gram-Scale Synthesis for Salmeterol and Vilanterol Intermediate (no $AgNO_3$ Condition) ... 18

Photochemical/Nickel-Mediated Decarboxylative Arylation 23

Frequently Asked Questions 24

Experimental Procedures and Characterization Data for Preparation of RAE 11 (*cis*) 27

Experimental Procedures and Characterization Data for Preparation of RAE 11 (*trans*) 29

Experimental Procedures and Characterization Data for Preparation of RAE 10 (*trans*) 32

Experimental Procedures and Characterization Data for Preparation of Other RAE 35

Experimental Procedures and Characterization Data for Model Reaction 38

Checking Stereochemical Erosion during Oxazolidine Removal 40

Experimental Procedures and Characterization Data for Decarboxylative Coupling Products 45

Synthetic Applications 63

Summary and Comparison with Previous Routes 81

X-Ray Structure 86

Reference 88

NMR Spectra: 90

General Experimental

Tetrahydrofuran (THF), dichloromethane (CH_2Cl_2), triethylamine (TEA) and toluene were obtained by passing the previously degassed solvents through an activated alumina column. Anhydrous DMA and $LiAlH_4$ (2 M in THF) were purchased from Sigma-Aldrich. $NiCl_2$·glyme was purchased from ChemScene LLC. All substrates that were purchased (from cheapest supplier), were used without further purification. Yields refer to chromatographically and spectroscopically (^{1}H NMR) homogeneous material, unless otherwise stated. Reactions were monitored by GC/MS, LC/MS, and thin layer chromatography (TLC). TLC was performed using 0.25 mm E. Merck silica plates (60F-254), using short-wave UV light as the visualizing agent, and cerium ammonium molybdate (CAM) or $KMnO_4$ and heat as developing agents. NMR spectra were recorded on Bruker DRX-600, DRX-500, and AMX-400 instruments and are referenced using residual undeuterated solvent ($CHCl_3$ at 7.26 ppm ^{1}H NMR, 77.16 ppm ^{13}C NMR; DMSO-*d6* at 2.50 ppm ^{1}H NMR, 39.52 ppm ^{13}C NMR; CH_3OH at 3.31 ppm ^{1}H NMR, 49.0 ppm ^{13}C NMR). The following abbreviations were used to explain multiplicities: s = singlet, d = doublet, t = triplet, q = quartet, m = multiplet, br = broad. Column chromatography was performed using E. Merck silica (60, particle size 0.043-0.063 mm), and pTLC was performed on Merck silica plates (60F-254). High-resolution mass spectra (HRMS) were recorded on an Agilent LC/MSD TOF mass spectrometer by electrospray ionization time of flight reflectron experiments. Gas chromatography-mass spectrometry (GCMS) was recorded on an Agilent 5975 MSD Series spectrometer.

General Procedure A: Ni-Electrocatalytic Diastereoselective Decarboxylative Arylation with Aryl Iodide as Coupling Partner

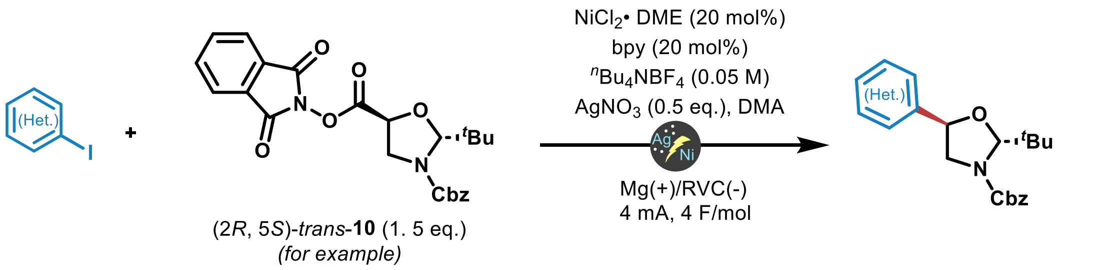

Electrochemical setup: An ElectraSyn vial (5 mL) with a magnetic stir bar was charged with aryl iodide (0.2 mmol, 1.0 equiv.), redox-active ester (RAE) (0.3 mmol, 1.5 equiv.), $NiCl_2$·DME (20 mol%), 2,2'-bipyridine (bpy) (20 mol%), $AgNO_3$ (0.5 eq) and $n$$Bu_4NBF_4$ (0.05 M). The ElectraSyn vial cap equipped with anode (magnesium) and cathode (RVC) (5.2 cm×0.7 cm×0.2 cm) was inserted into the mixture. The vial was then evacuated and backfilled with an argon balloon for three cycles, and DMA (3.5 mL) was added to the vial *via* a syringe and the resulting solution was stirred for another 1 min. The vial was connected to the ElectraSyn, and the ElectraSyn was set up as follow: New exp. > Constant current > 4 mA > No ref. electrode > Total charge > 0.2 mmol, 4.0 F/mol > No alternating polarity > Start. After electrolysis, the ElectraSyn vial cap was removed and electrodes were rinsed with Et_2O, which was combined with the crude mixture. The crude mixture was further diluted with Et_2O and aqueous HCl (0.1 N) was then added [for products containing basic motifs, washing with 0.1 N HCl was omitted]. The organic layers were further washed with brine, dried over anhydrous Na_2SO_4 and concentrated *in vacuo*. The crude material was purified by flash column chromatography or preparative thin layer chromatography (pTLC) to furnish the desired product.

Graphical Guide for Ni-Electrocatalytic Diastereoselective Decarboxylative Arylation with Aryl Iodide as Coupling Partner

Representative example (0.2 mmol scale)

Photos were taken from the coupling between 4-(2-(benzyloxy)ethoxy)-1-chloro-2-iodobenzene (**12**) and (2*R*, 5*S*)-*trans*-Cbz-**10**

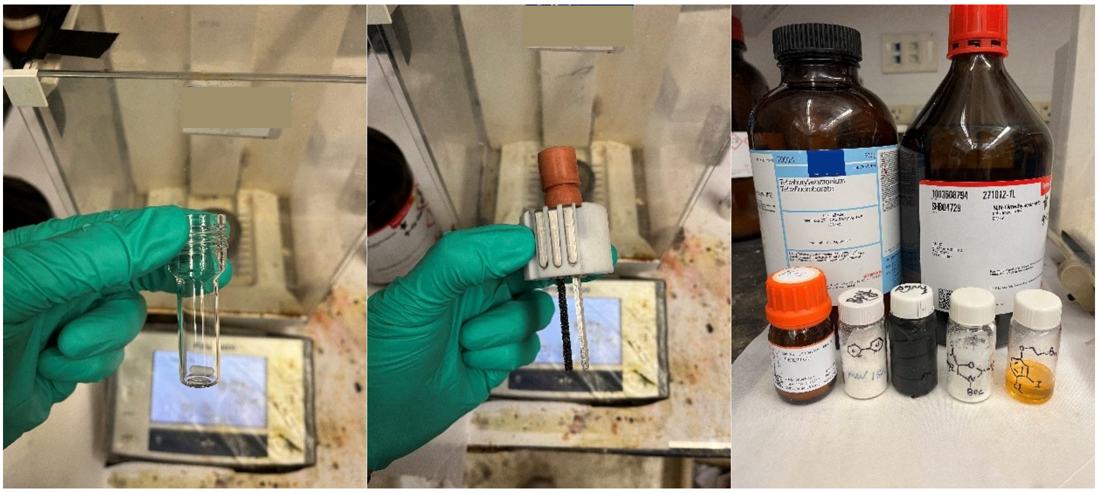

(**Left**) Clean 5 mL Electrasyn 2.0 vial with a stirring bar. (**Center**) ElectraSyn 2.0 cap equipped with anode (magnesium) and cathode (RVC). (**Right**) All reagents for the reaction.

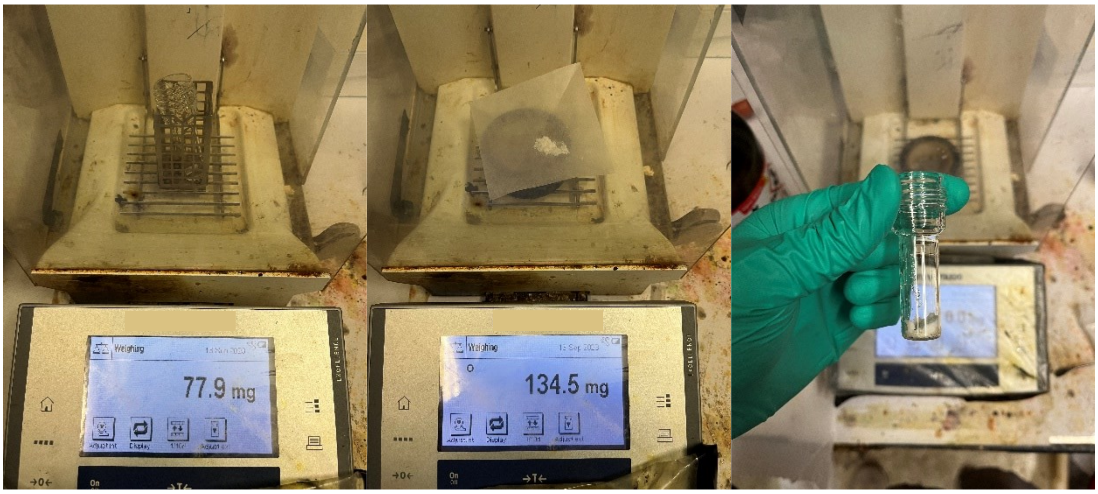

(Left) Addition of 4-(2-(benzyloxy)ethoxy)-1-chloro-2-iodobenzene (**12**) (78 mg, 0.2 mmol) to the vial. **(Center)** (2R, 5S)-*trans*-Cbz-**10** (135 mg, 0.3 mmol) was weighed. **(Right)** All reagents were weighed out and combined in the vial.

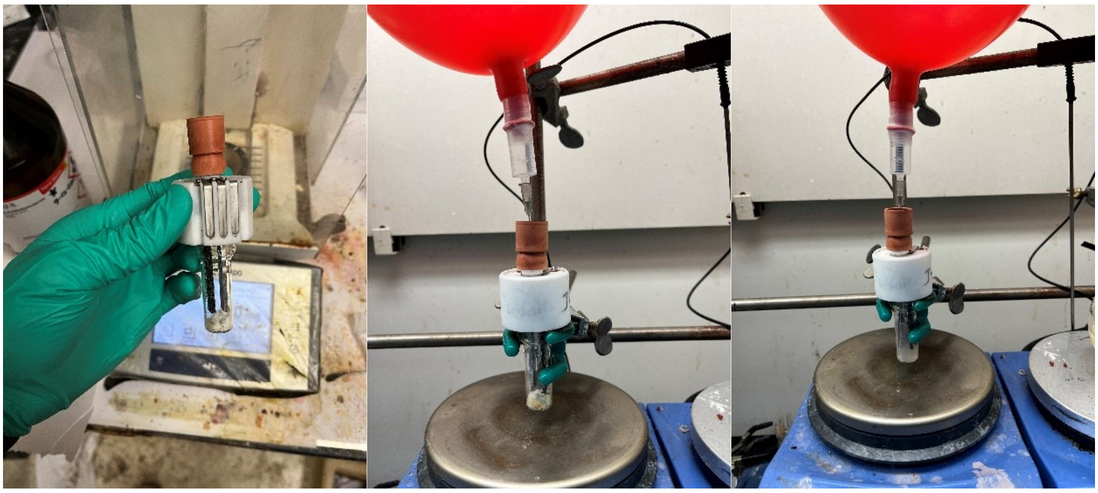

(Left) ElectraSyn cap was tightly screwed onto the reaction vial. **(Center)** The vial was then evacuated and backfilled with an argon balloon. **(Right)** DMA (3.5 mL) was added, the reaction solution was stirred for roughly 60 seconds.

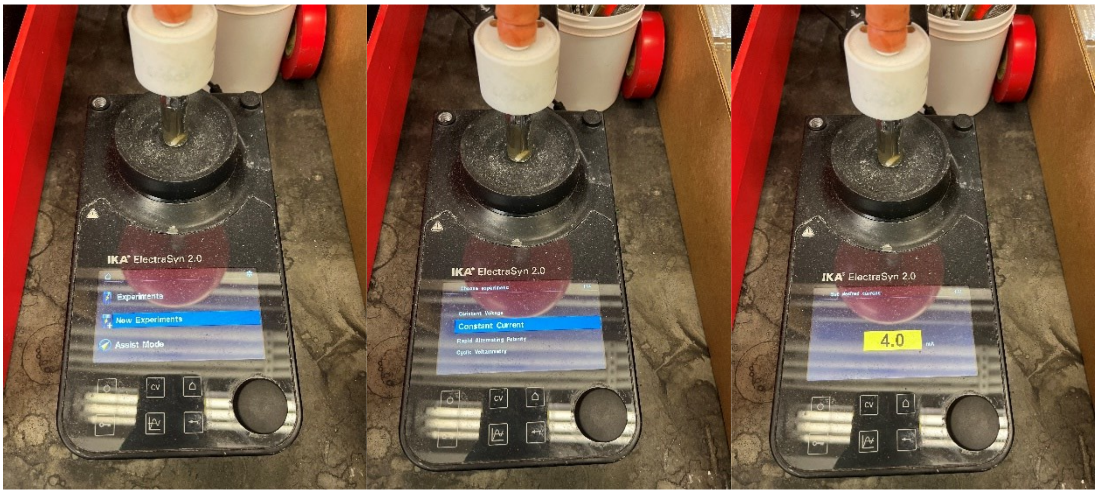

(Left) Select a new experiment. **(Center)** Select constant current. **(Right)** Set the current to 4 mA (for a 0.20 mmol scale). Permission is granted by IKA.

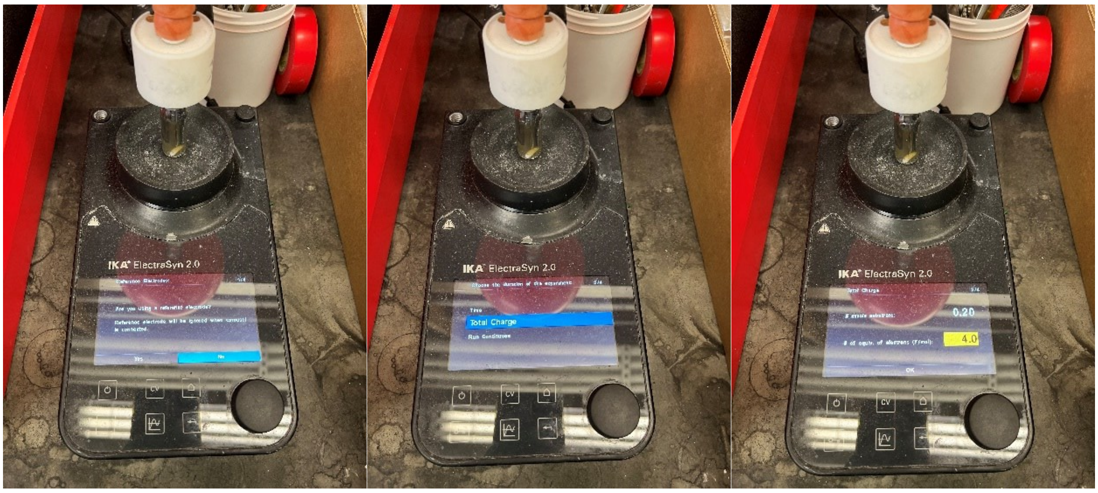

(Left) No need to use a reference electrode. **(Center)** Select "Total Charge". **(Right)** Select 0.20 mmol of substrate at 4 F/mol. Permission is granted by IKA.

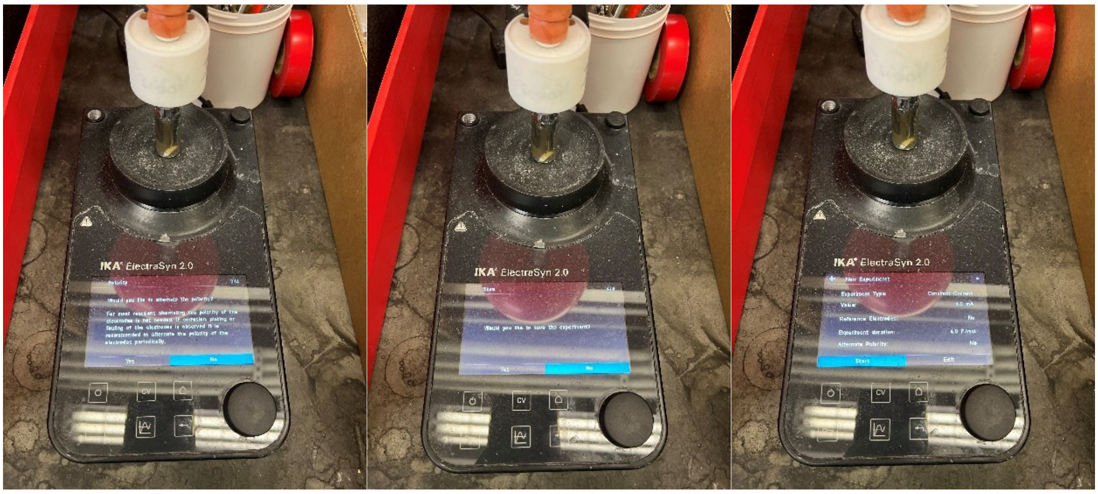

(Left) No need to alternate the polarity. **(Center)** Saving reaction parameters is optional. **(Right)** Select "Start" when ready. Permission is granted by IKA.

General procedure B: Ni-Electrocatalytic Diastereoselective Decarboxylative Arylation with Aryl Bromide as Coupling Partner (Slow Addition)

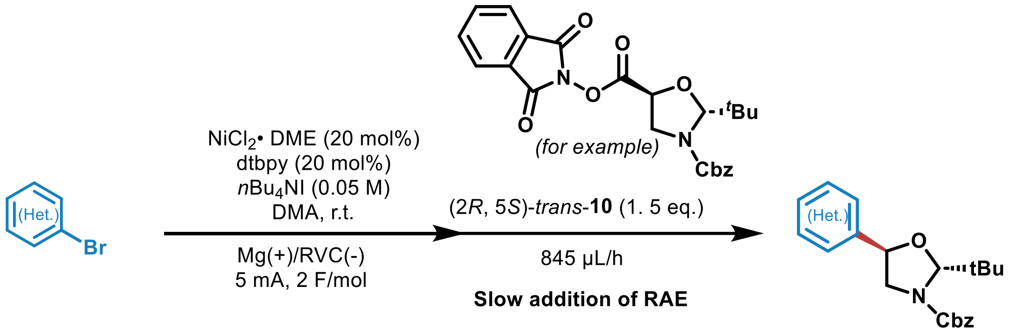

Electrochemical setup: An ElectraSyn vial (5 mL) with a magnetic stir bar was charged with aryl bromide (0.1 mmol, 1.0 equiv.), $NiCl_2$·DME (20 mol%), 2,2'-bipyridine (bpy) (20 mol%), and *n*Bu_4I (0.05 M). The ElectraSyn vial cap equipped with anode (magnesium) and cathode (RVC) (5.2 cm×0.7 cm×0.2 cm) was inserted into the mixture. The vial was then evacuated and backfilled with an argon balloon for three cycles, and DMA (2.5 mL) was added to the vial *via* a syringe and the resulting solution was stirred for another 1 min. The vial was connected to the ElectraSyn, and the ElectraSyn was set up as follow: New exp. > Constant current > 5 mA > No ref. electrode > Total charge > 0.15 mmol, 2.0 F/mol > No alternating polarity > Start. During electrolysis, redox-active ester (RAE) (0.15 mmol, 1.5 equiv.) in DMA (1.0 mL) was added at the speed of 845 µL/h *via* syringe pump. After electrolysis, the ElectraSyn vial cap was removed and electrodes were rinsed with Et_2O, which was combined with the crude mixture. The crude mixture was further diluted with Et_2O and aqueous HCl (0.1 N) was then added [for products containing basic motifs, washing with 0.1 N HCl was omitted]. The organic layers were further washed with brine, dried over anhydrous Na_2SO_4 and concentrated *in vacuo*. The crude material was purified by flash column chromatography or preparative thin layer chromatography (pTLC) to furnish the desired product.

Graphical Guide for Ni-Electrocatalytic Diastereoselective Decarboxylative Arylation with Aryl Bromide as Coupling Partner (Slow Addition)

Representative Example (0.1 mmol scale)

Photos were taken from the coupling between 4-(2-(benzyloxy)ethoxy)-1-chloro-2-bromobenzene (**12-Br**) and (2*R*, 5*S*)-*trans*-Cbz-**10**

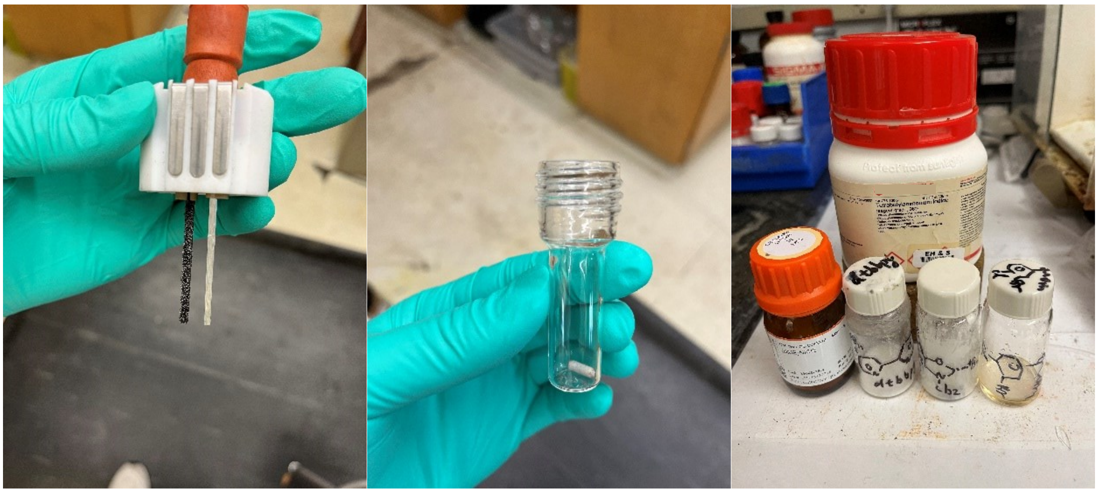

(**Left**) ElectraSyn 2.0 cap equipped with anode (magnesium) and cathode (RVC). (**Center**) Clean 5 mL Electrasyn 2.0 vial with a stirring bar. (**Right**) All reagents for the reaction.

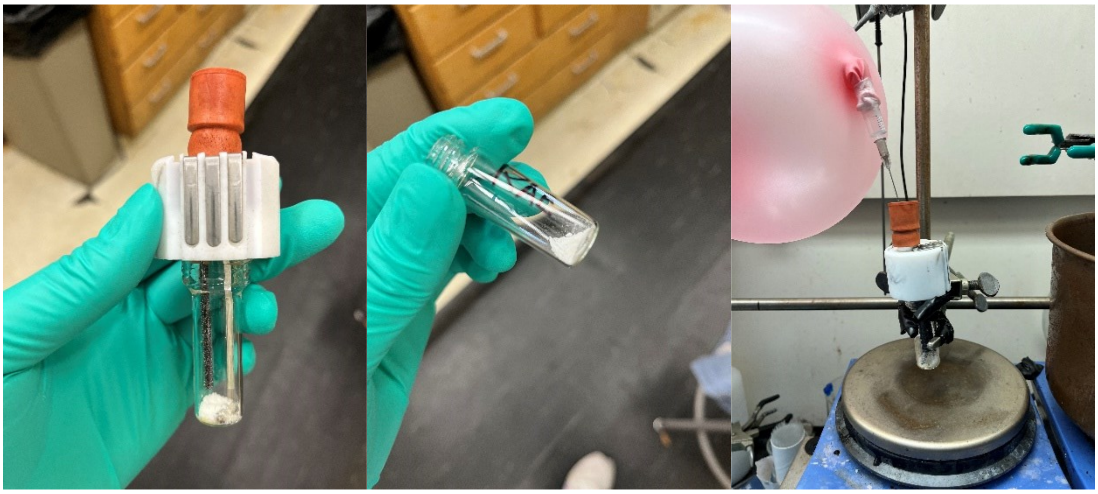

(**Left**) Add reagents into the ElectraSyn vial and attach the cap. (**Center**) Add redox active ether (RAE) **10** (*trans*) to separate 7 mL vial. (**Right**) The vial was then evacuated and backfilled with an argon balloon.

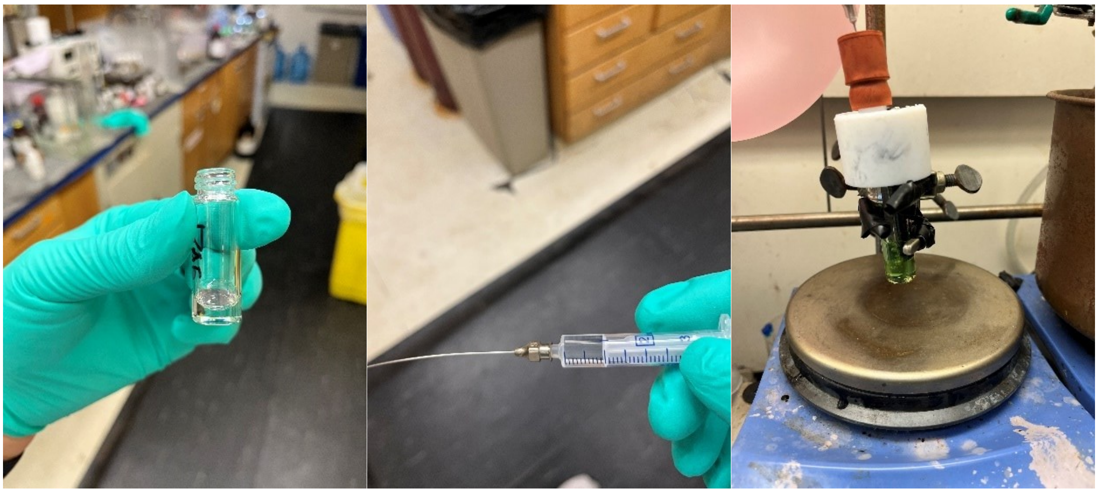

(**Left**) Dissolve RAE in 1.0 mL of DMA. (**Center**) Take RAE solution from vial to 3 mL syringe. (**Right**) Dissolved reagent in 2.5 mL *N*,*N*-dimethylacetamide (DMA) and attached the vial to the ElectraSyn 2.0.

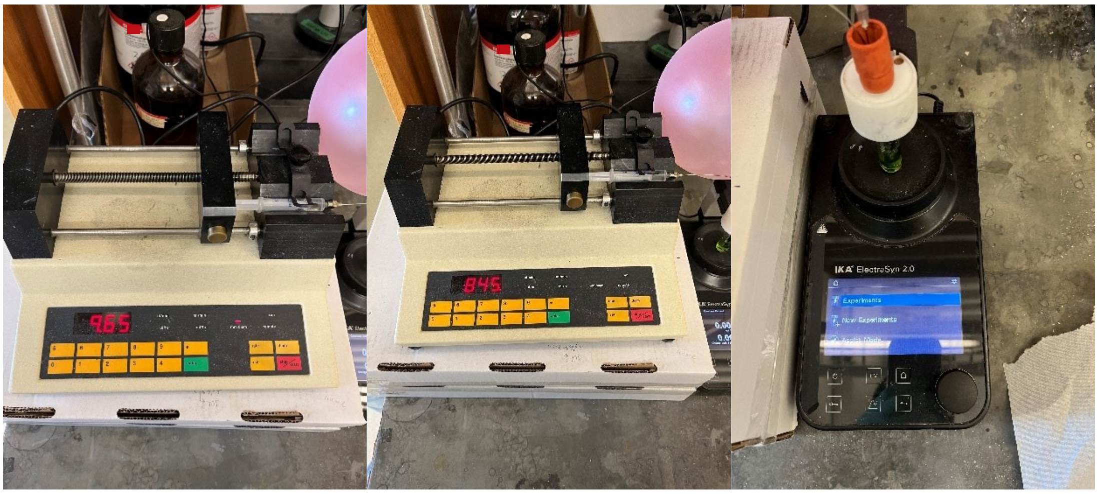

(**Left**) Set up diameter parameter (9.65 mm) of the syringe pump. (**Center**) Set up the injection speed (845 μL) of the syringe pump. (**Right**) Select "New Experiments" Permission is granted by IKA.

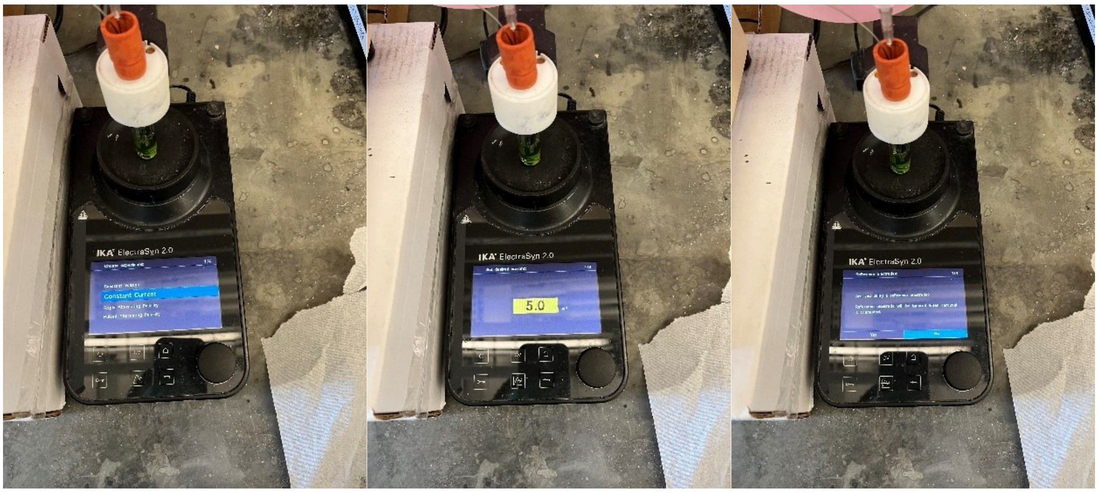

(**Left**) Select “Constant Current”. (**Center**) Set the current to 5.0 mA. (**Right**) Select “No” without a reference electrode. Permission is granted by IKA.

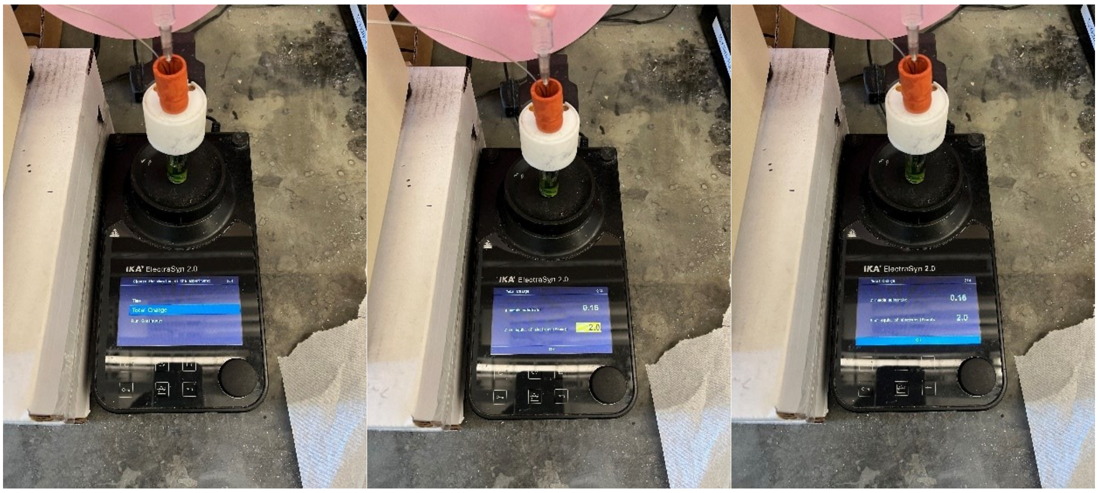

(**Left**) Select “Total Charge”. (**Center**) Set the scale to 0.15 mmol, and set the “equivalents of electrons” to 2.0 F/mol. (**Right**) Select “OK”. Permission is granted by IKA.

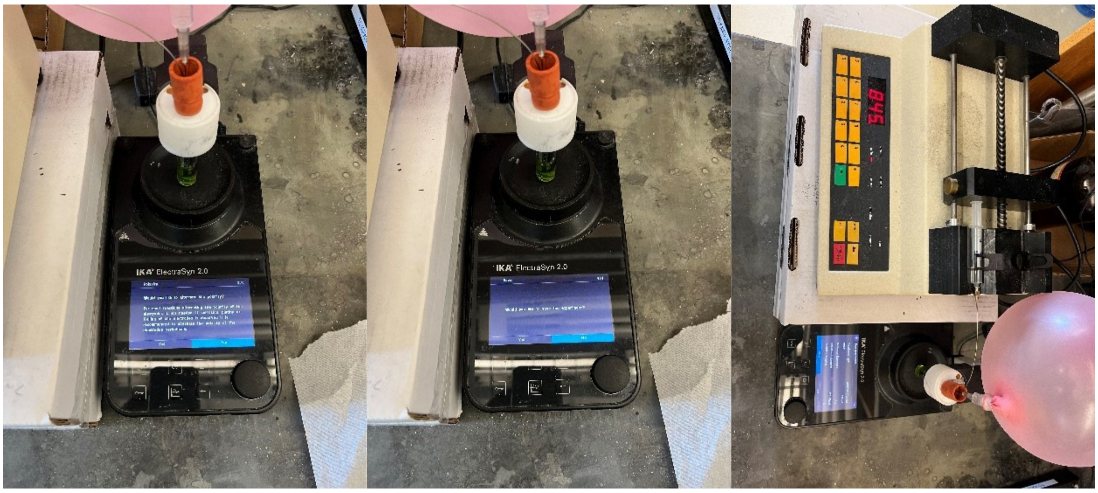

(**Left**) Select “No” when prompted about alternating polarity. (**Center**) Select “No” to not save the experiment. (**Right**) Needle of the syringe was inserted into the reaction vessel, and select “Start” to launch the reaction. Permission is granted by IKA.

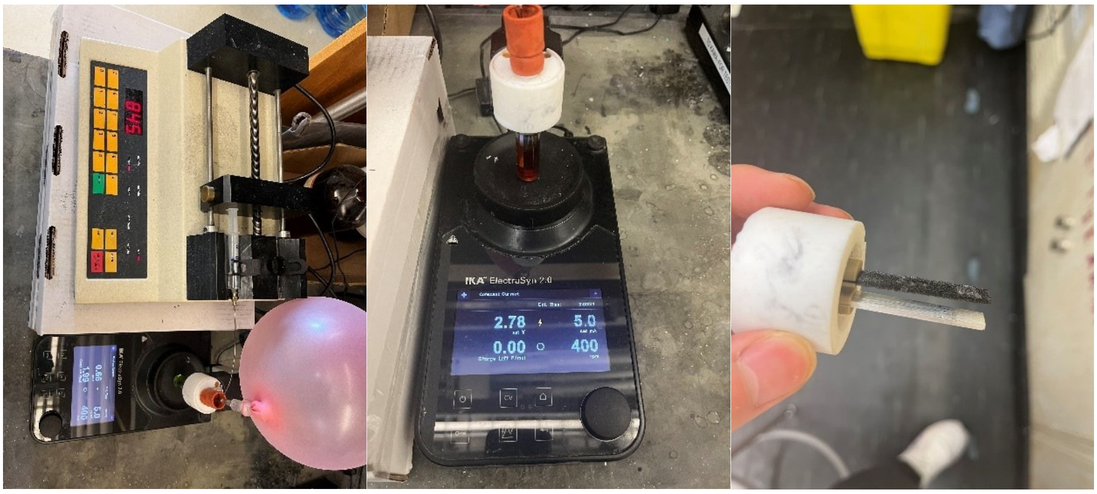

(**Left**) Press “Run/Stop” button of the syringe pump after electrolysis started. (**Center**) Reaction mixture at the end of electrolysis (**Right**) Electrode after electrolysis. Permission is granted by IKA.

Optimization of Reaction Parameters for the Ni-Electrocatalytic Diastereoselective Decarboxylative Arylation

Reaction parameter screening of Ley auxiliary-base RAE

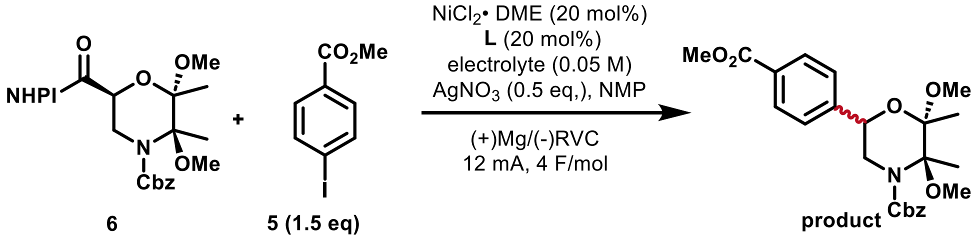

Entry	L	Electrolyte	Combined yield (%)	*dr*
1	dtbbpy	*n*Bu$_4$NBF$_4$	51	1:1.5
2	none	*n*Bu$_4$NBF$_4$	trace	-
3	dtbbpy	$MgCl_2$	5	-
4	dtbbpy	$MgBr_2$•Et_2O	3	-
5	dtbbpy	$Mg(ClO_4)_2$	trace	-
6	dtbbpy	LiCl	1	-
7	dtbbpy	$ZnCl_2$	8	1:2
8	dtbbpy	TBACl	17	1:1.4
9	dtbbpy	TBABr	11	1:1.3
10	dtbbpy	TBAI	6	1:1.3
11	**L1**	*n*Bu$_4$NBF$_4$	8	1:1.9
12	**L2**	*n*Bu$_4$NBF$_4$	10	1:1.5
13	**L3**	*n*Bu$_4$NBF$_4$	0	-
14	**L4**	*n*Bu$_4$NBF$_4$	52	1:2.8
15	**L5**	*n*Bu$_4$NBF$_4$	10	1:1.2
16	**L6**	nBu$_4$NBF$_4$	26	1:3.7
17	**L7**	*n*Bu$_4$NBF$_4$	8	1.1:1
18	**L8**	*n*Bu$_4$NBF$_4$	2	1:1.1
19	**L9**	*n*Bu$_4$NBF$_4$	0	-

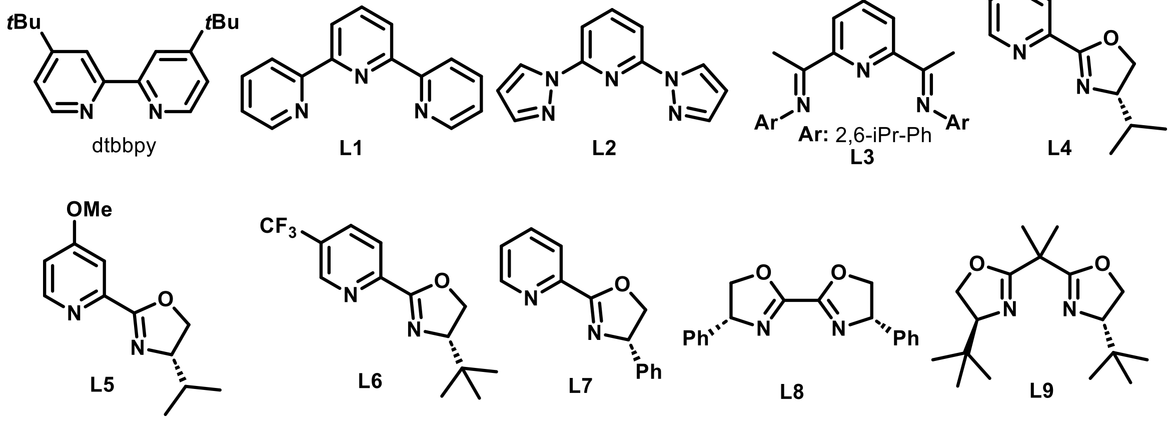

Aryl iodide as coupling partner

Ligand effects

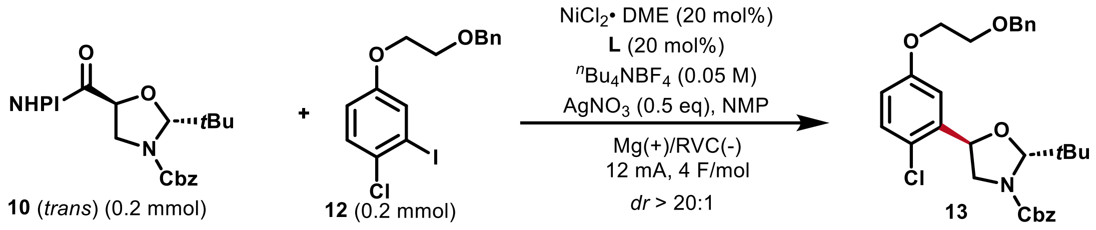

Entry	Variations	Yield (%)
1	**L1**	11
2	**L2**	12
3	**L3**	2
4	**L4**	4
5	**L5**	1
6	**L6**	1
7	**L7**	0
8	**no Ligand**	0

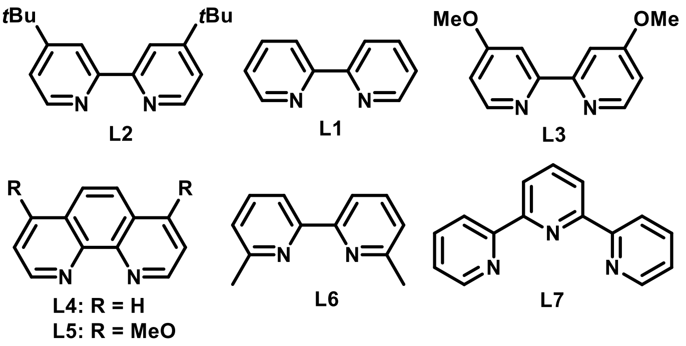

Other effects

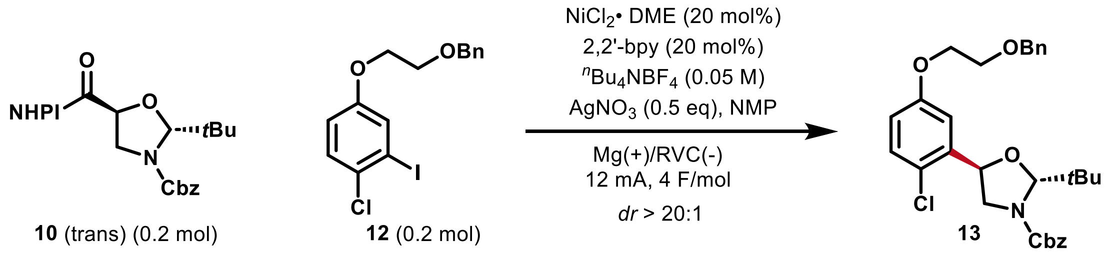

Entry	Variations	Yield (%)
1	8 mA	11
2	4 mA	22
3	2 mA	17
4	DMF, 4 mA	22
5	DMA, 4 mA	30
6	RAE (1.5), 4 mA	33
7	RAE (2.0), 4 mA	39
8	RAE (1.5), DMA, 4 mA	65
9	RAE (1.5), DMA, 4 mA<br>Zn as cathod	9

Ni source and electrolyte effects

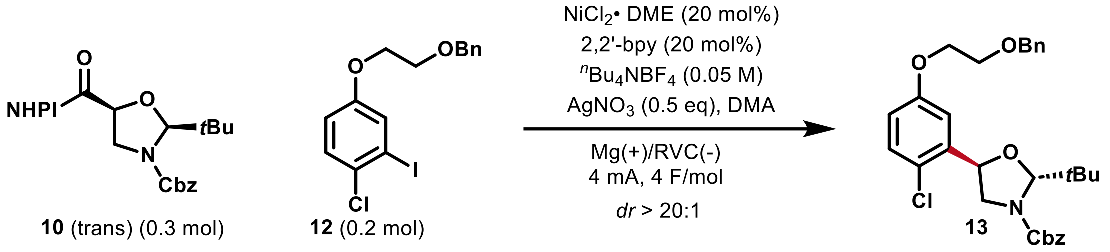

10 (trans) (0.3 mol) **12** (0.2 mol) **13**

Entry	Variations	Yield (%)
1	none	65
2	$NiBr_2{\bullet}3H_2O$	49
3	$NiCl_2{\bullet}6H_2O$	14
4	NiI_2	4
5	$NiBr_2{\bullet}DME$	54
6	$Ni(acac)_2$	0
7	$Ni(NO_3)_2{\bullet}6H_2O$	14
8	$^nBu_4NCl$	trace
9	$^nBu_4NBr$	18
10	$^nBu_4NI$	44
11	$LiBF_4$	36

Solvent effects

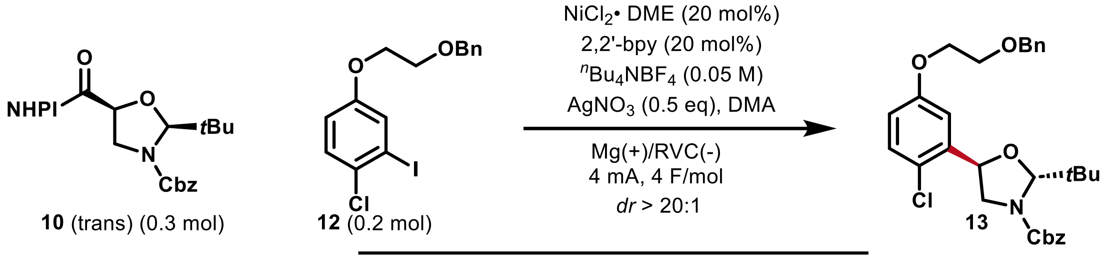

10 (trans) (0.3 mol) **12** (0.2 mol) **13**

Entry	Variations	Result yield (%)
1	none	65
2	DMF	21
3	NMP	61
4	DMSO	trace
5	MeCN	trace
6	Acetone	0
7	DMPU	45
8	NMP:DCM (1:1)	48

Additive effects

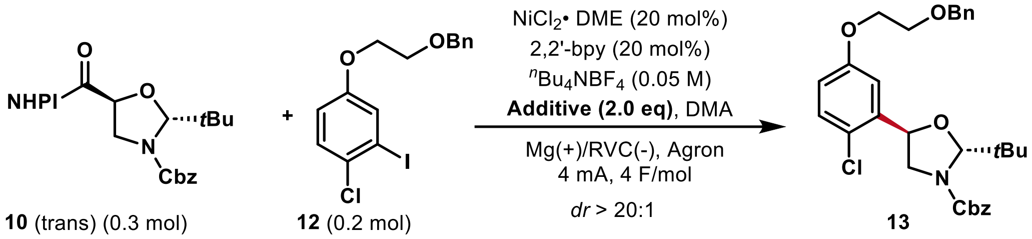

10 (trans) (0.3 mol) **12** (0.2 mol) **13**

Entry	Additives	Yield (%)
1	$AgNO_3$	65
2	no $AgNO_3$	43
3	Et_3N	0
4	AcOH	39
5	$PhB(OH)_2$	44
6	*t*BuOH	60
7	*t*BuOH + $AgNO_3$	59
8	water (2.0 eq)	61
9	water (4.0 eq)	45

Control experiments

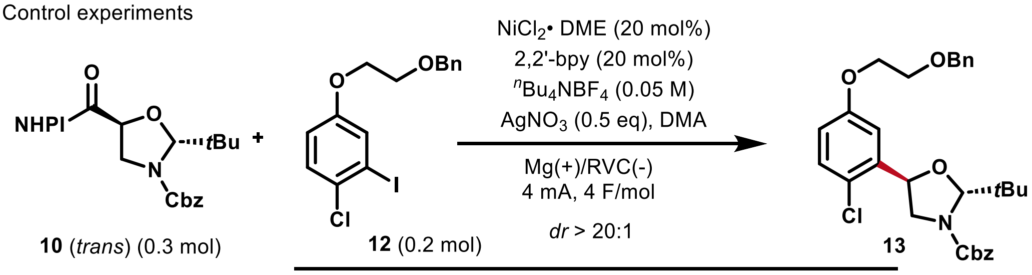

10 (*trans*) (0.3 mol) **12** (0.2 mol) **13**

Entry	Variations	Yield (%)
1	none	65
2	Electrolysis for 0.5 F/mol then no electricity for 4.5 h	trace
3	Electrolysis for 0.5 F/mol then no electricity for 4.5 h (without $AgNO_3$)	7
4	Mg powder as reductant (no electricity)	6
5	Mg powder as reductant (without $AgNO_3$ (no electricity)	trace
6	Mg powder as reductant (without $AgNO_3$) (no electricity) (add 1 drop AcOH)	trace
7	(Ni + L) (10 mol%)	41

Optimization for Gram-Scale Electrochemical Coupling of GSK-656 Intermediate (no $AgNO_3$ Condition)

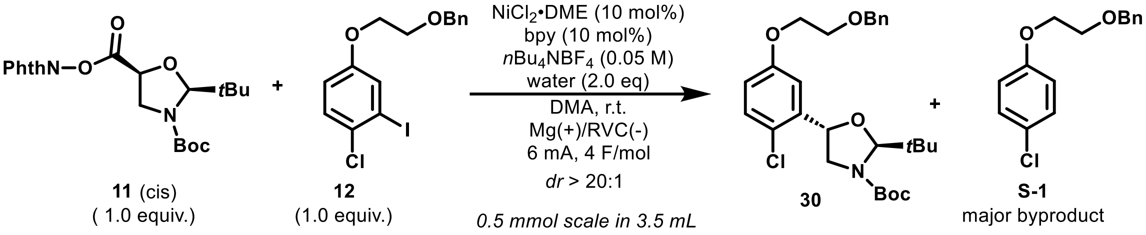

Entry	Variations	Yield 30 (%)	S-1 (%)
1	none	28	38
2	12 mA	17	37
3	$LiBF_4$ as electrolyte, 12 mA	15	44
4	RAE (1.5 eq), 12 mA	15	29
5	Aryl Bromide	trace	-
6	20 mol% Ni/L	40	10
7	iodide (1.2 eq)	41	36
8	iodide (1.5 eq)	41	35
	**RAE 11 (1.2 eq), Halide 12 (1.0 eq)**		
1	$NiCl_2$ (10 mol%)	22	7
2	$NiCl_2$ (15 mol%)	28	9
3	$NiCl_2$ (20 mol%)	53	14

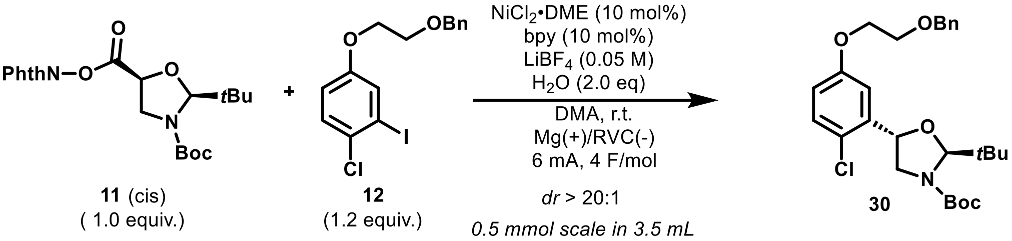

Entry	Variations	Yield 30 (%)
1	$LiBF_4$ + no H_2O	36
2	none	51
3	H_2O (1.0 eq)	43
4	H_2O (4.0 eq)	44
5	$LiBF_4$ (0.1 M)	47
7	+ $MgBr_2$ (2.0 eq)	44
8	LiBr as electrolyte	20
9	Ni+L (15 mol%)	42
10	Ni+L (20 mol%)	29
11	18 mA	38
12	graphite as cathode	12

Gram-Scale Synthesis for Salmeterol and Vilanterol Intermediate (no $AgNO_3$ Condition)

(1) Optimization

Scale up study of echem coupling (0.1 mmol scale)

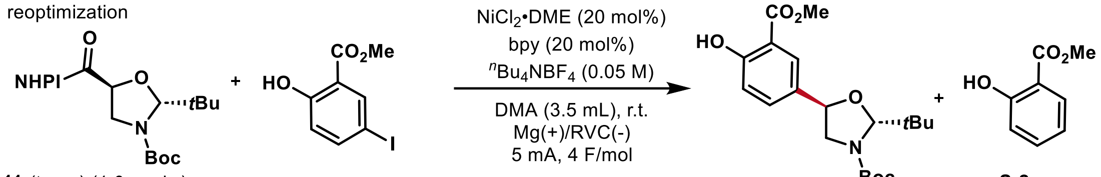

11 (trans) (1.0 equiv.) **43** (1.2 equiv.) **44** **S-2** (major byproduct)

Entry	Variations	Yield (%)	Conv. (%)	S-2 (%)
1	$NiCl_2$•$6H_2O$ + 2 eq. DME	45	99>	53
2	$NiCl_2$•$6H_2O$ + 50 eq. DME	34	93	42
3	none	42	95	40
4	$NiCl_2$•$6H_2O$ +4 A molecular seives (20 mg)	30	83	25
5	$NiCl_2$•$6H_2O$	33	97	40
6	10 mol% (catalyst + ligand)	50	99	43
7	5 mol% (catalyst + ligand)	22	68	35
8	10 mol% (catalyst + ligand) 20 mA	43	95	44
9	10 mol% (catalyst + ligand) 20 mA, 2.2 F/mol	32	59	24

Scale-up study of echem coupling (0.5 mmol scale)

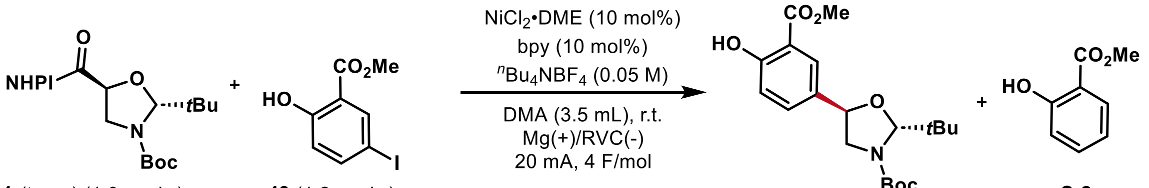

11 (trans) (1.0 equiv.) **43** (1.2 equiv.) **44** **S-2** (major byproduct)

Entry	Variations	Yield (%)	Conv. (%)	S-2 (%)
1	none	43	99>	44
2	0.1 M $^{n}Bu_4BF_4$	36	99>	43
3	$LiBF_4$ as electrolyte	50	99>	42
4	LiBr as electrolyte	40	95	45
5	$LiBF_4$ as electrolyte 10 mA	55	98	34
6	$LiBF_4$ as electrolyte 1.0 mmol scale	51	99	36
7	open to air	55	99	35

(2) Graphical Guide for a Large-Scale Synthesis of 44

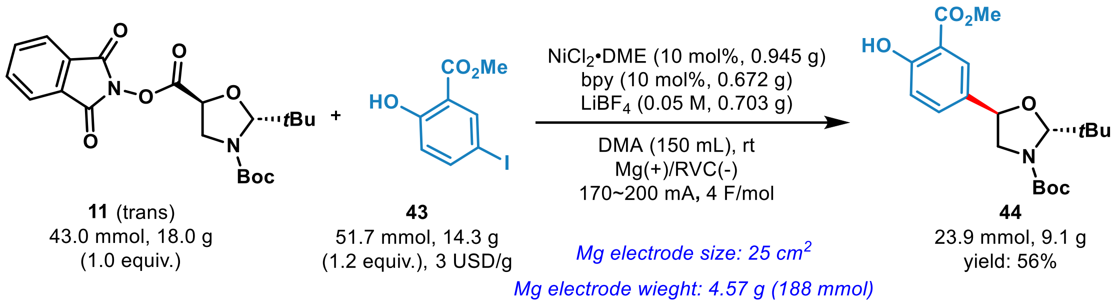

A commercial glass vessel (diameter: 7 cm, height: 9 cm) with a stirring bar was charged with **11** (*trans)*-Boc-RAE (18.0 g, 43.0 mmol, 1.0 equiv.), methyl 2-hydroxy-5-iodobenzoate **43** (14.3 g, 51.7 mmol, 1.2 equiv.), $NiCl_2$·DME (0.945 g, 4.30 mmol, 0.1 equiv.), 2,2'-bipyridine (bpy) (0.672 g, 4.30 mmol, 0.1 equiv.) and $LiBF_4$ (0.703 g, 7.50 mmol, 0.05 mol/L). The mixture was dissolved in *N*,*N*'-dimethylacetamide (DMA) (150 mL) and stirred. A modified plastic cap (see graphical guide below) equipped with magnesium anode and RVC cathode was inserted into the vessel. The reaction mixture was electrolyzed under a constant current of 170 mA for 4.0 F/mol. After 1 hour, the constant current was increased from 170 mA to 200 mA. After electrolysis, the cap was removed and electrodes were rinsed with Et_2O. The crude mixture was diluted in 250 mL of Et_2O. Then, the combined organic layers were washed with 0.1 N *aq.* HCl (50 mL) and H_2O (50 mL). The organic layers were dried over anhydrous Na_2SO_4, filtered and concentrated under reduced pressure. The crude material was purified by silica gel column chromatography (hexanes:EtOAc = 20:1 to 15:1) to give desired product **44** (9.10 g, 23.9 mmol, 56% yield) as white solid.

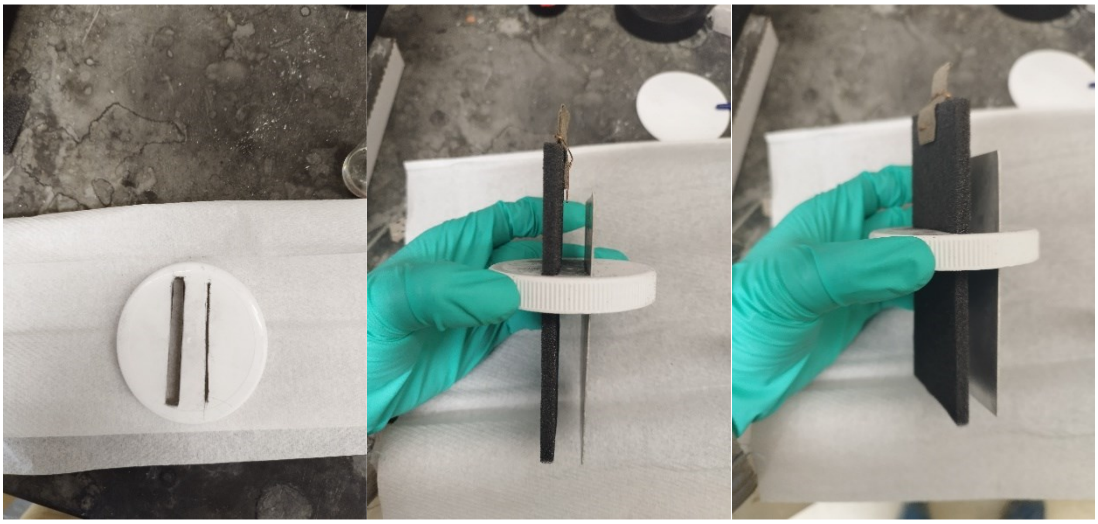

(**Left**) Slits of appropriate sizes were cut out on the original cap of the glass chamber to accommodate the electrodes. (**Center**) Attach the magnesium anode (10.0 cm×5.0 cm×0.1 cm) and the RVC cathode (11.5 cm×5.0 cm×0.4 cm) to the cap. (**Right**) The nickel foam is attached to the top of RVC for connection.

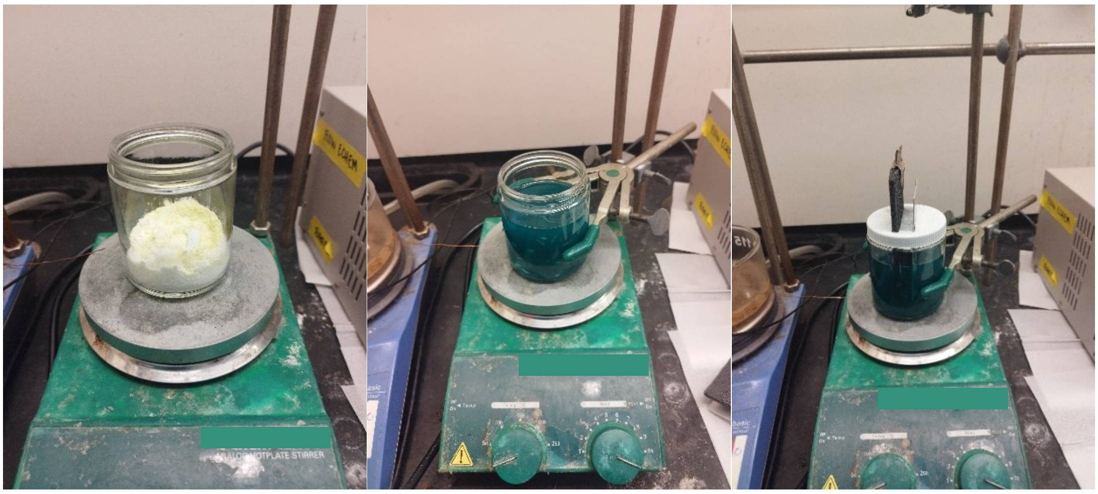

(**Left**) Add reagents into the reaction vessel. (**Center**) Add 150 mL of DMA into the reaction vessel. (**Right**) Attach the electrode equipped cap on the reaction vessel.

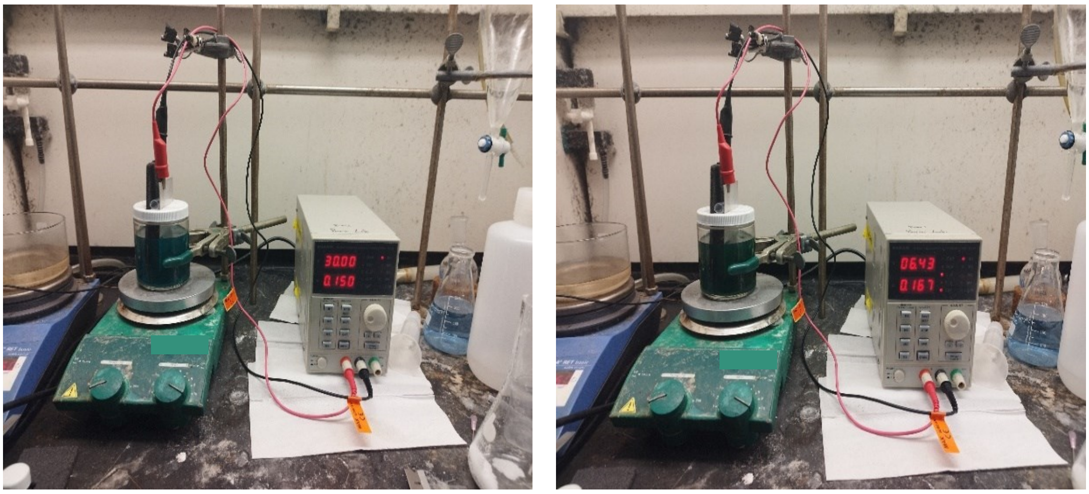

(**Left**) Connect DC power supplier and set the condition. (**Right**) Start the reaction.

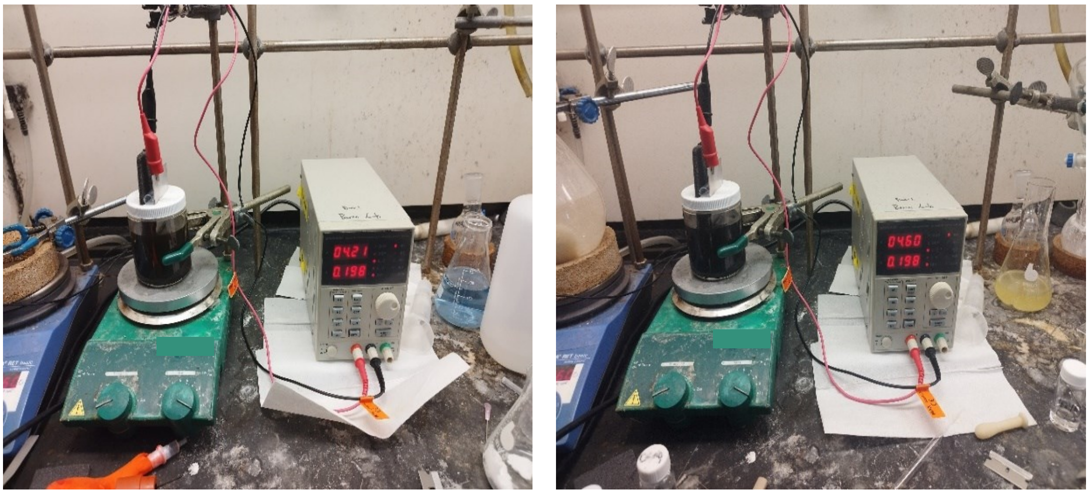

(**Left**) Reaction after 15 hours (2.5 F/mol). (**Right**) Reaction after 24 hours (4.0 F/mol).

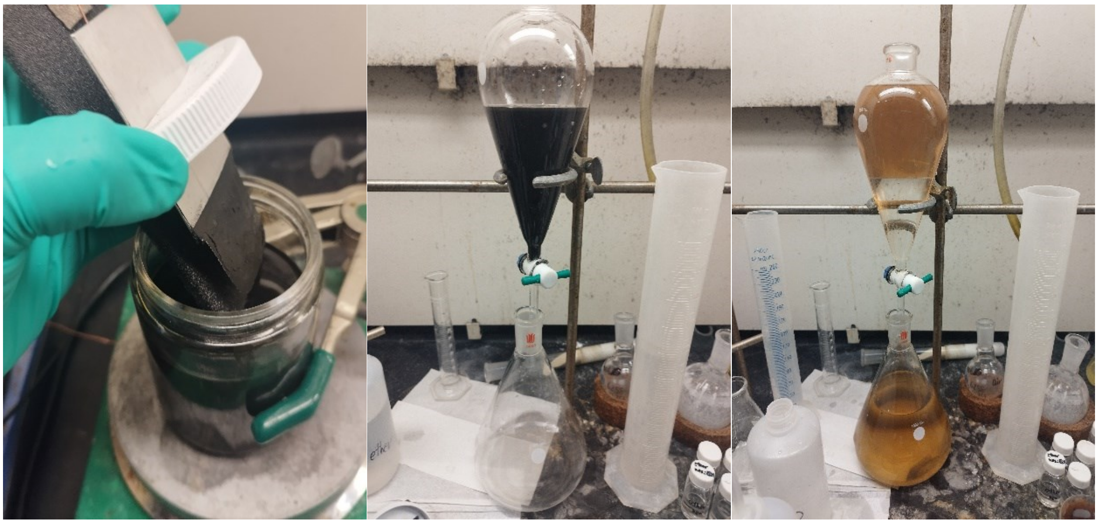

(**Left**) Rinse electrodes with Et_2O. (**Center**) Wash with Et_2O/0.1N HCl (*aq.*). (**Right**) Completion of wash.

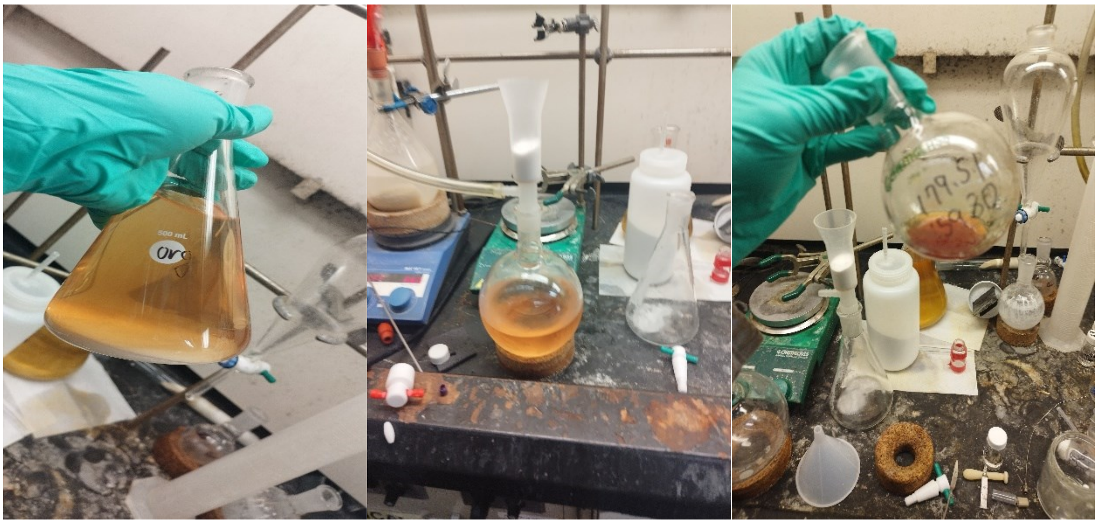

(**Left**) Dry with Na_2SO_4. (**Center**) Filter Na_2SO_4 and remove solvent. (**Right**) Crude product 20.2 g.

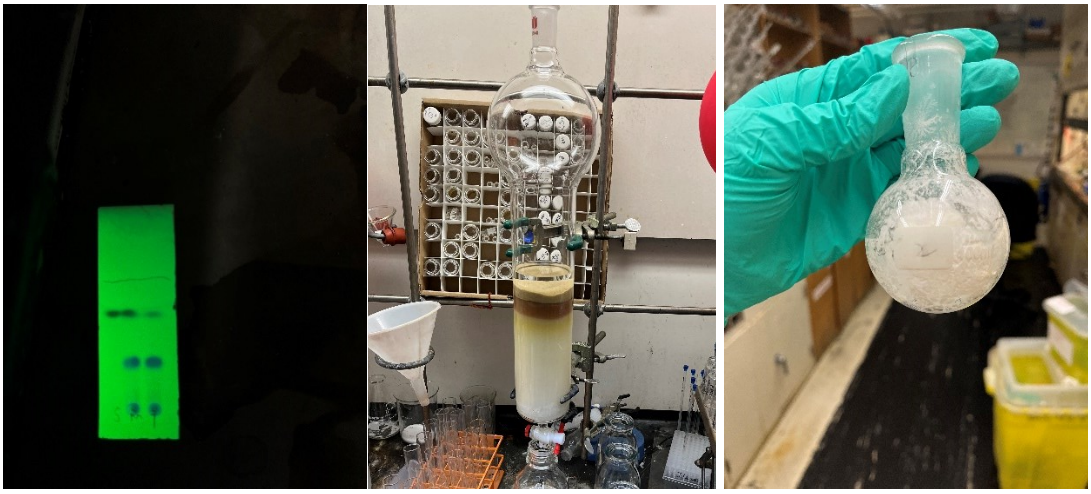

(Left) Crude TLC (8:1 hexanes:EtOAc). **(Center)** Purification by flash column chromatography (15:1 hexanes:EtOAc). **(Right)** Desired product (9.1 g, 56% yield).

Photochemical/Nickel-Mediated Decarboxylative Arylation

(1) Carboxylic Acid as Coupling Partner[1]

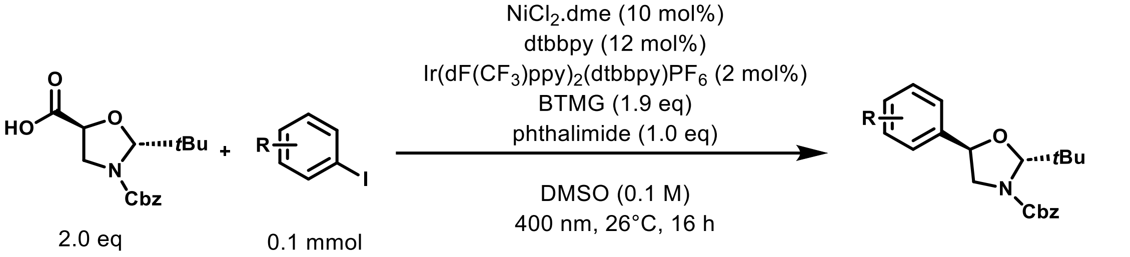

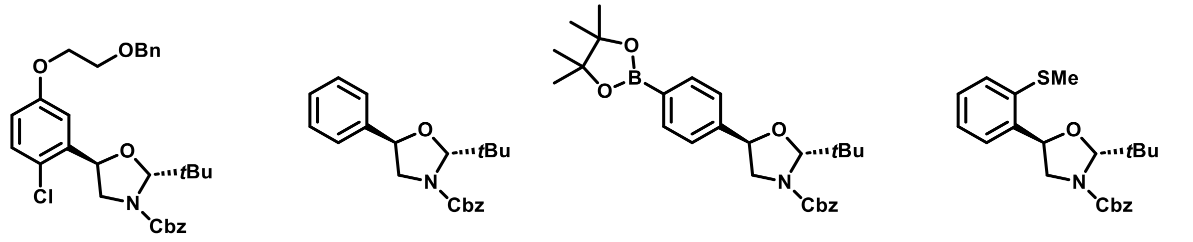

22% yield, *dr* > 20:1 | 12% yield, *dr* > 20:1 | 19% yield, *dr* > 20:1 | 17% yield, *dr* > 20:1

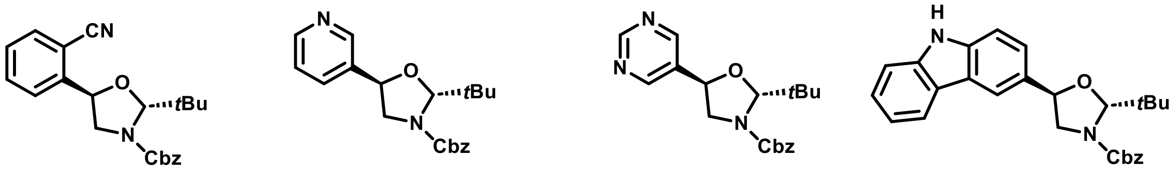

21% yield, *dr* > 20:1 | no product observed | no product observed | X=I: 11% yield, *dr* >20:1; X=Br: 23% yield, *dr* >20:1

(2) RAE as Coupling Partner[2]

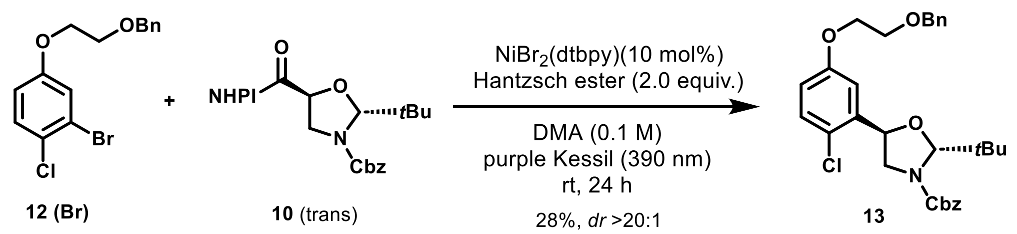

12 (Br) | **10** (trans) | 28%, *dr* >20:1 | **13**

Frequently Asked Questions

Question 1:

How do I clean the Mg sacrificial electrodes before use?

Answer:

The magnesium anode was typically submerged into 1.0 M HCl solution for 15 seconds, and washed with water and acetone. Surface of the magnesium electrode is then scraped with a razor blade to expose some fresh surface.

Question 2:

Is this reaction sensitive to water and air?

Answer:

The reaction is not sensitive to the water; In fact, H_2O (2.0 equiv.) as additive will not affect this reaction. On the other hand, the reaction can be somewhat air sensitive depending on the scale. On 0.1-0.2 mmol scale, we observed slightly decreased yield when running the reaction open to air. In contrast, no variation in yield was observed on 0.5 mmol scale.

Question 3:

How do I monitor the reaction?

Answer: We use TLC analysis with UV visualization (254 nm) to see the starting material and desired products. If UV absorption was too weak, chemical stain was recommended. Typically, TLC plate was dipped in cerium ammonium molybdate (CAM) stain or permanganate stain for a few seconds, then it is common to heat the TLC plate to speed up visualization.

Question 4:

What are the typical side products of the reaction?

Answer:

Regarding the aryl halide, dehalogenation and homodimerization were the major side reactions. Regarding the RAE, decarboxylative reduction was the most common side product observed.

Question 5:

Which situation was the slow addition of RAE (Redox-Active Esters) into the reaction mixture required?

Answer:

Slow addition of the RAE (see General Procedure B) is required when an aryl bromide was used as the coupling partner, the reason being that the RAE was found to be consumed faster than the aryl bromide during electrolysis. We also noticed that the addition rate of the RAE solution can affect the yield. It is recommended to adjust and experiment with different addition rates to achieve the optimal result.

Question 6:

Is $AgNO_3$ necessary for this decarboxylative arylation?

Answer:

We found $AgNO_3$ is not critical to the product formation, but generally improves the yield moderately. The role of $AgNO_3$ is that it could be reduced to Ag nanoparticle layer on the cathode surface, which reduces undesired direct cathodic reduction of RAE (*Science* **2022**, *375*, 745-752.).

Question 7:

What reaction voltage and the suitable current should I expect for successful reaction?

Answer:

We typically use 4 mA on 0.2 mmol scales and 12 mA on 1.0 mmol scale. Normal operational voltage range for this reaction is around 0 V - 5.0 V. When a reaction needs to be run on different scale, all the parameters except for substrate concentration (i.e.

solvent amount) should follow general procedures.

Question 8:

How did you determine the diastereoselectivity of this Ni-catalytic decarboxylative arylation?

Answer:

^{1}H-NMR was used to determine the diastereoselectivity. During the reconstruction of the oxazolidine oing (In this SI, Page 39), we got *cis*-oxazolidine ring compound **13** (*cis*), which was very helpful to determine the diastereoselectivity. Generally, we integrated the peak at ~5.2 ppm (dd, major diastereomer) and ~2.8 ppm (t, minor diastereomer) to get the d.r.

Question 9:

Is this oxazolidine structure sensitive to the acid during work-up?

Answer:

This structure is not sensitive to the acid. The oxazolidine structure with Cbz protecting group on the nitrogen atom could survive in HCl (conc.)/MeOH at 50 °C, and the oxazolidine structure with Boc protecting group on the nitrogen atom can be treated with 4 M HCl (*aq.*) during work-up.

Experimental Procedures and Characterization Data for Preparation of RAE 11 (*cis*)

(1) Preparation of S-5 (mixture)

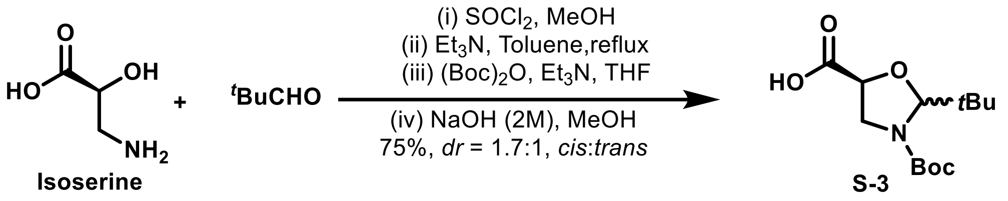

To a solution of **(*S*)-isoserine** (42.5 g, 405 mmol, 1.0 equiv.) in methanol (500 mL) at 0 ºC was added thionyl chloride (88 mL, 1.21 mol, 3.0 equiv.). After 4 h, the solution was concentrated under reduced pressure to give amine·HCl salt. Then, to a solution of amine·HCl salt in toluene (500 mL) was added triethylamine (112 mL, 810 mmol, 3.0 equiv.) and pivalaldehyde (67 mL, 607 mmol, 1.5 equiv.). The mixture was heated to reflux with a Dean-stark apparatus attached until no additional water was collected in the trap. The Et_3N·HCl salt was filtered and rinsed with Et_2O. The solvent was removed under reduced pressure to give a crude yellow oil, which was redissolved in tetrahydrofuran (200 mL) and treated with di-*tert*-butyl dicarbonate (Boc_2O) (93 mL, 405 mmol, 1.0 equiv.) at 0 ºC. The reaction was allowed to reach room temperature and stirred overnight. After the reaction, solvent was removed under reduced pressure to give the crude mixture. Then MeOH (360 mL) was added to dissolve the crude mixture and *aq.* 2 M NaOH (120 mL) was added in a single portion. The reaction was stirred at ambient temperature for 12 hours at which point the TLC analysis indicated the complete consumption of the starting material. MeOH was removed under reduced pressure, and the aqueous phase was washed with EtOAc (400 mL) first to remove excess Boc_2O. The aqueous phase was acidified with 1N HCl (*aq.*) to a pH = 1-4 and extracted with EtOAc (3 × 400 mL) until the aqueous phase become a transparent solution. The combined organic layers were washed with brine, dried over Na_2SO_4, filtered and concentrated *in vacuo*. Colorless oil **S-3** (mixture) [84 g, 75% yield, *dr* = 1.7 (*cis*):1 (*trans*)] was obtained as an inseparable mixture.

(2) Preparation of RAE 11 (*cis*)

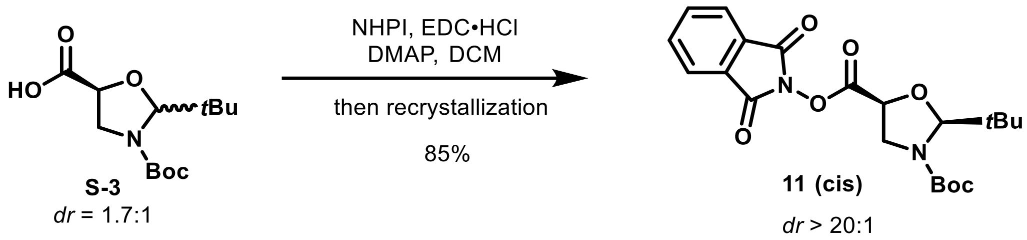

A round-bottom flask was charged with **S-3** (8.5 g, *dr* = 1.7:1, 31.1 mmol, 1.0 equiv.), *N*-hydroxyphthalimide (5.0 g, 31.1 mmol, 1.0 equiv.), EDC·HCl (6.0 g, 31.1 mmol,1.0 equiv.), and DMAP (379 mg, 3.11 mmol,0.1 equiv.), DCM (124 mL) was added, and the mixture was stirred vigorously at ambient temperature for 3 h at which point the TLC analysis indicated complete consumption of the starting material. The mixture was diluted with in CH_2Cl_2 (100 mL), and washed with *aq.* 1 N HCl (50 mL × 2), and brine. The organic layer was dried over anhydrous Na_2SO_4, filtered and concentrated under reduced pressure to give the crude activated ester which was then recrystallized in EtOAc to give **11** (cis) (7.5 g, 58% yield, 85% yield based on *cis*-acid, *dr* > 20:1) as a white solid. [After 3 cycles of recrystallizations, **11** (*trans*) could obtained in 10:1 *dr*]

Physical State: white solid.

R_f = 0.58 (hexanes/EtOAc 1:1, UV).

$[\alpha]_D^{25}$ = -14.5 (c 1.0, $CHCl_3$).

^{1}H NMR (600 MHz, $CDCl_3$) δ 7.90 (dd, *J* = 5.4, 3.1 Hz, 2H), 7.80 (dd, *J* = 5.5, 3.0 Hz, 2H), 5.24 (s, 1H), 4.75 (dd, *J* = 9.7, 6.9 Hz, 1H), 4.59 (s, 1H), 3.47 (dd, *J* = 11.6, 9.8 Hz, 1H), 1.50 (s, 9H), 0.97 (s, 9H).

^{13}C NMR (151 MHz, $CDCl_3$) δ 166.3, 161.6, 154.4, 135.1, 128.9, 124.3, 97.6, 81.7, 72.8, 48.6, 37.7, 28.4, 28.4, 25.3.

Experimental Procedures and Characterization Data for Preparation of RAE 11 (*trans*)

(1) Preparation of acid 11 (*cis*-Boc)

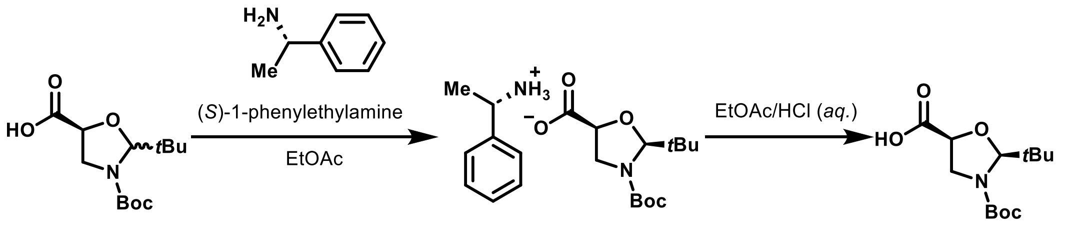

S-3 *dr* = 1.7:1 | **white solid** | **11 (cis-Boc-acid)** *dr* > 20:1

To a solution of **S-3** (mixture) (66.0 g, 242 mmol, *dr* = 1.7:1.0) in EtOAc (400 mL) was added (*S*)-phenylethylamine (19.4 mL, 151 mmol). After 1 hour of stirring, the white precipitates formed and collected by filtration, and the mother liquor was collected and washed with 1N HCl (*aq.*) for recycling. The white precipitates were redissolved in EtOAc and washed with 1N HCl (*aq.*) and brine. The organic layer was dried over anhydrous Na_2SO_4, filtered and concentrated under reduced pressure to give acid **11** (*cis*-Boc) (34.2 g, 52%, *dr* > 20:1) as a white waxy solid.

Physical State: white waxy solid.

R*f* = 0.30 (EtOAc, UV).

$[\alpha]_D^{25}$ = -44.9 (c 1.0, $CHCl_3$).

^{1}H NMR (600 MHz, CD_3OD) δ 5.11 (s, 1H), 4.95 (s, 1H), 4.38 (dd, *J* = 10.0, 6.7 Hz, 1H), 4.31 (dd, *J* = 11.2, 6.7 Hz, 1H), 3.21 – 3.14 (m, 1H), 1.49 (s, 9H), 0.94 (s, 9H).

^{13}C NMR (151 MHz, CD_3OD) δ 172.4, 172.4, 156.1, 97.8, 82.4, 75.6, 49.5, 38.5, 28.5, 28.5, 25.7.

HRMS: Calc'd for $C_{13}H_{22}NO_5$, $[M-H]^-$ 272.1503; found 272.1499.

(2) Preparation of acid 11 (*trans*-Boc)

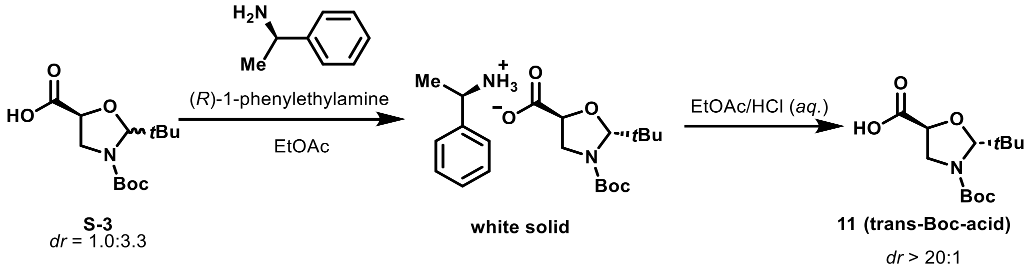

To a solution of **S-3 (mixture)** (31.6 g, 116 mmol, *dr* = 1.0:3.3) in EtOAc (200 mL) was added (*R*)-(+)-phenylethylamine (10.4 mL, 80.5 mmol). After 1 hour stirring, the white precipitates formed were collected by filtration, and the mother liquor was collected and washed with 1N HCl (*aq.*) for recycling. The white precipitates were dissolved in EtOAc and washed with 1N HCl (*aq.*) and brine. The organic layer was dried over anhydrous Na_2SO_4, filtered and concentrated under reduced pressure to give acid **11** (*trans*-Boc) (14.6 g, 22%, *dr* >20:1) as a white solid.

Physical State: white waxy solid.

R_f = 0.30 (EtOAc, UV).

[α]D^{25} = +66.7 (c 1.0, $CHCl_3$).

^{1}H NMR (600 MHz, CD_3OD) δ 5.27 (s, 1H), 4.57 (dd, *J* = 7.2, 1.3 Hz, 1H), 4.26 (d, *J* = 11.9 Hz, 1H), 3.42 (dd, *J* = 11.8, 7.1 Hz, 1H), 1.46 (s, 9H), 0.92 (s, 9H).

^{13}C NMR (151 MHz, CD_3OD) 175.3 (br), 157.0 (br), 97.7 (br), 82.3 (br), 75.6 (br), 50.3 (br), 38.1, 28.5, 25.6.

HRMS: Calc'd for $C_{13}H_{22}NO_5$, $[M-H]^-$ 272.1503; found 272.1498.

(3) Preparation of RAE 11 (*trans*)

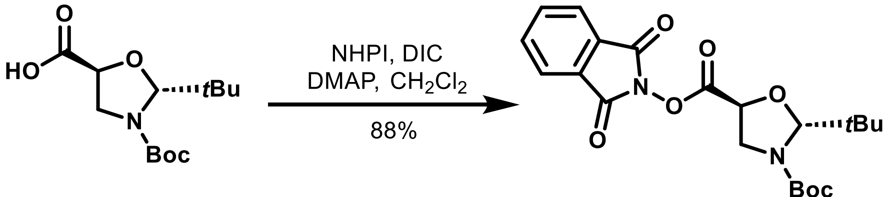

11 (trans-Boc-acid) **11 (trans)**

A round-bottom flask was charged with carboxylic acid (10.0 g, 36.6 mmol, 1.0 equiv.), *N*-hydroxyphthalimide (6.6 g, 40.3 mmol, 1.1 equiv.), and DMAP (451 mg, 3.7 mmol, 1.0 equiv.). DCM (180 mL) was added, and the mixture was stirred vigorously. DIC (6.2 ml, 40.3 mmol, 1.1 equiv) was then added dropwise *via* a syringe, and the reaction mixture was allowed to stir until the acid was fully consumed (determined by TLC). Typical reaction times were between 0.5 and 2 hours. Upon completion, the mixture was concentrated *in vacuo* and directly purified by silica gel column chromatography (eluent: hexanes/EtOAc = 2/1) to afford the RAE **11** (*trans*) (13.5 g, 88%) as a waxy solid.

Physical State: white solid.

$\boldsymbol{R_f}$ = 0.58 (hexanes/EtOAc 1:1, UV).

$[\alpha]_D^{25}$ = +47.0 (c 1.0, $CHCl_3$).

^{1}H NMR (600 MHz, $CDCl_3$) δ 7.88 (dd, *J* = 5.5, 3.1 Hz, 2H), 7.79 (dd, *J* = 5.5, 3.1 Hz, 2H), 5.41 (s, 1H), 4.95 (dd, *J* = 7.3, 1.4 Hz, 1H), 4.57 (s, 1H), 3.67 – 3.54 (m, 1H), 1.47 (s, 9H), 0.94 (s, 9H).

^{13}C NMR (151 MHz, $CDCl_3$) δ 168.1, 161.3, 134.8, 128.9, 124.1, 97.2, 81.4, 72.8, 49.4 (br), 37.5, 28.1, 25.1.

Experimental Procedures and Characterization Data for Preparation of RAE 10 (*trans*)

(1) Preparation of acid 10 (*trans*-Cbz)

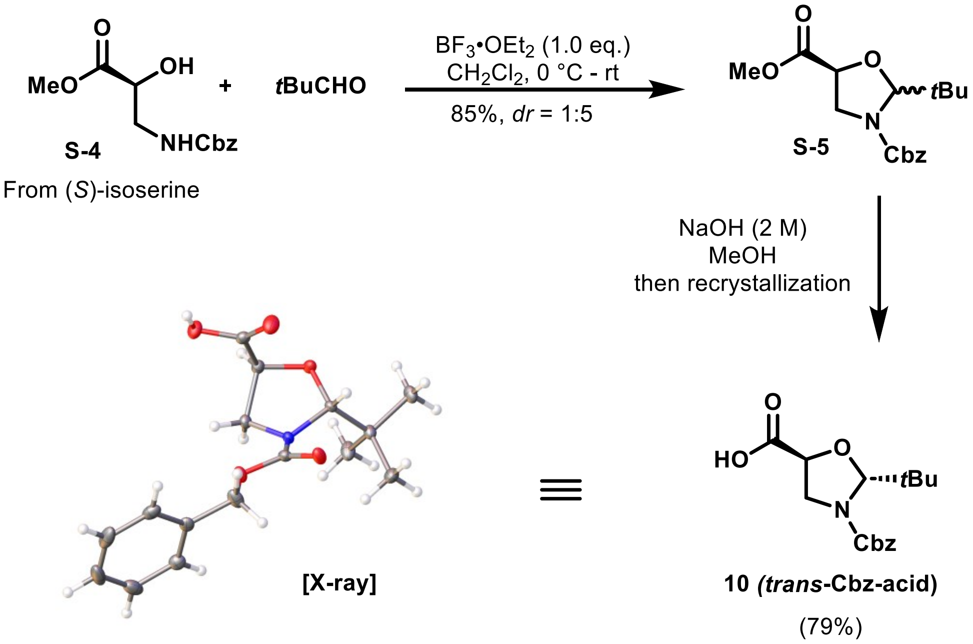

To a solution of **methyl (S)-3-(((benzyloxy)carbonyl)amino)-2-hydroxypropanoate S-4** (6.0 g, 23.7 mmol, 1.0 equiv.) and pivalaldehyde (7.7 mL, 71.1 mmol, 3.0 equiv.) in anhydrous CH_2Cl_2 (50.0 mL) was added $BF_3 \cdot OEt_2$ (2.9 mL, 23.7 mmol, 1.0 equiv.) over a period of 2 h at 0 ºC. The reaction was warmed to ambient temperature and stirred overnight. The reaction was quenched by the addition of *sat. aq.* $NaHCO_3$ (10 mL) and diluted with CH_2Cl_2 (20 mL). The layers were separated and the aqueous layer was extracted with CH_2Cl_2 (2 × 20 mL). The combined layers were washed with brine, dried over Na_2SO_4, filtered and concentrated *in vacuo*. The crude residue was purified by SiO_2 flash chromatography (eluent: hexanes/EtOAc = 4/1) to give colorless oil **S-5**[6.5 g, *dr* = 1(*cis*):5 (*trans*)] as an inseparable mixture.

To a solution of **S-5** (4.00 g, 12.5 mmol, 1.0 equiv.) in MeOH (42.0 mL) was added 2 M NaOH (aq) (12.0 mL) at room temperature. The reaction was stirred at ambient temperature for 12 hours at which point the TLC analysis indicated the complete consumption of the starting material. After the reaction, MeOH was removed under

reduced pressure. The aqueous phase was acidified with 1N HCl (*aq.*) to pH = 1-4 and extracted with EtOAc (3 × 60 mL). The combined organic layers were washed with brine, dried over Na_2SO_4, filtered and concentrated *in vacuo*. The crude residue was recrystallized in EtOAc to give **10** (*trans*-Cbz-acid) (3.0 g, 79%, *dr* >20:1) as a white solid. After 2 cycles of recrystallization, **10** (*cis*-Cbz-acid) (610 mg, *dr* = 5:1) was obtained from mother liquor as a colorless oil which gradually solidified into a white waxy solid over 12 h.

Physical State: white solid.

R_f = 0.30 (EtOAc, UV).

$[\alpha]_D^{25}$ = +60.0 (c 1.0, $CHCl_3$).

^{1}H NMR (600 MHz, CD_3OD) δ 7.51 – 7.12 (m, 5H), 5.33 (s, 1H), 5.16 (d, *J* = 12.4 Hz, 1H), 5.11 (d, *J* = 12.4 Hz, 1H), 4.63 (dd, *J* = 7.3, 1.4 Hz, 1H), 4.31 (d, *J* = 11.8 Hz, 1H), 3.52 (dd, *J* = 11.8, 7.2 Hz, 1H), 0.91 (s, 9H).

^{13}C NMR (151 MHz, CD_3OD) δ 175.1 (br), 157.5 (br), 137.6 (br), 129.6, 129.2, 128.9 (br), 97.8, 75.8 (br), 68.7 (br), 50.3 (br), 38.2, 25.6.

HRMS: Calc'd for $C_{16}H_{20}NO_5$, $[M-H]^-$ 306.1346; found 306.1343.

(2) Preparation of acid 10 (*trans*-Cbz) and acid 10 (*cis*-Cbz)

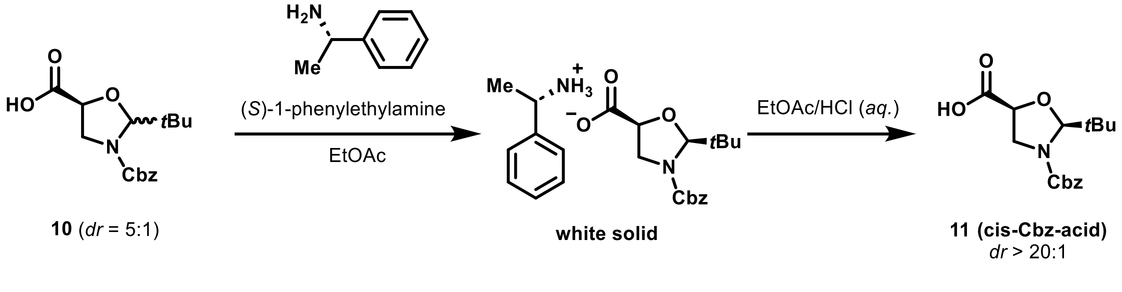

The preparation of **acid 11 (cis-Cbz)** *was carried out in analogy to* **acid 11 (cis-Boc)**.

Physical State: white waxy solid.

R_f = 0.30 (EtOAc, UV).

$[\alpha]_D^{25}$ = -41.4 (c 1.0, $CHCl_3$).

^{1}H NMR (600 MHz, CD_3OD) δ 7.42 – 7.29 (m, 5H), 5.20 – 5.11 (m, 3H), 4.39 (m,

2H), 3.25 (t, J = 10.4 Hz, 1H), 0.92 (s, 9H).

^{13}C NMR (151 MHz, CD_3OD) δ 172.3, 156.9, 137.5, 129.6, 129.3, 129.3, 98.0, 75.8, 68.9, 49.7, 38.4, 25.6.

HRMS: Calc'd for $C_{16}H_{20}NO_5$, $[M-H]^-$ 306.1346; found 306.1340.

(3) Preparation of RAE 10 (*trans*-Cbz)

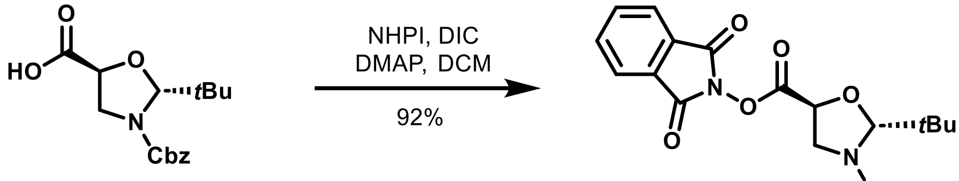

10 (*cis*-Cbz-acid) **10 (*cis*)**

The preparation of **RAE 1o (*trans*)** *was carried out in analogy to* **RAE 11 (*trans*)** in the 6-gram scale**.**

Physical State: white solid.

R_f = 0.55 (hexanes/EtOAc 1:1, UV).

$[\alpha]_D^{25}$ = +48.3 (c 1.0, $CHCl_3$).

^{1}H NMR (400 MHz, $CDCl_3$) δ 7.88 (dd, J = 5.5, 3.1 Hz, 2H), 7.80 (dd, J = 5.5, 3.1 Hz, 2H), 7.48 – 7.24 (m, 5H), 5.50 (s, 1H), 5.20 (s, 2H), 4.99 (dd, J = 7.2, 1.3 Hz, 1H), 4.62 (s, 1H), 3.65 (dd, J = 12.2, 7.2 Hz, 1H), 0.95 (s, 9H).

^{13}C NMR (100 MHz, $CDCl_3$) δ 168.1, 161.4, 155.6 (br), 136.1 (br), 135.0, 128.9, 128.5, 128.5, 128.2, 124.2, 97.3 (br), 73.3 (br), 68.1 (br), 49.7 (br), 37.5, 25.1.

Experimental Procedures and Characterization Data for Preparation of Other RAE

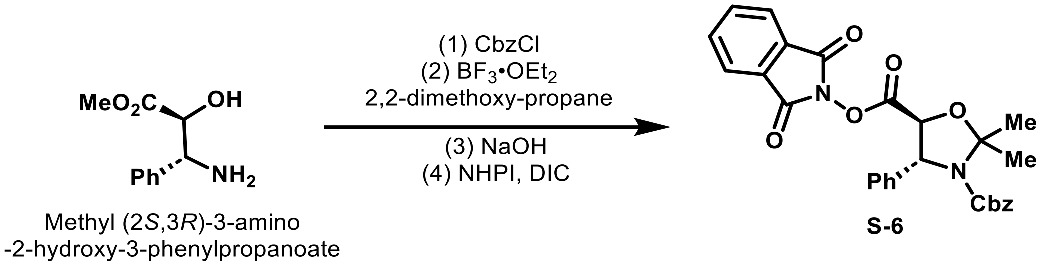

The preparation of **S-6** (RAE) was carried out in analogy to RAE **10** (cis-Cbz), starting from (2S,3R)-methyl 2-hydroxy-3-amino-3-phenylpropionate.

Physical State: colorless oil.

R_f = 0.51 (hexanes/EtOAc 1:1, UV).

$[\alpha]_D^{25}$ = -97.1 (c 1.0, $CHCl_3$).

^{1}H NMR (600 MHz, $CDCl_3$) δ 7.88 (dd, *J* = 5.5, 3.1 Hz, 2H), 7.78 (dd, *J* = 5.5, 3.1 Hz, 2H), 7.48 – 7.26 (m, 6H, br), 7.25 – 7.10 (m, 3H, br), 6.83 (s, 1H, br), 5.44 (s, 1H, br), 5.24 – 4.76 (m, 3H), 1.85 (s, 3H, br), 1.81 (s, 3H, br).

^{13}C NMR (151 MHz, $CDCl_3$) δ 167.4, 166.6, 161.9, 161.4, 152.1 (br), 135.8, 135.0, 134.8, 128.9, 128.8, 128.3, 127.5, 126.5, 124.1, 124.0, 98.4 (br), 79.8 (br), 67.0 (br), 63.8 (br), 17.6.

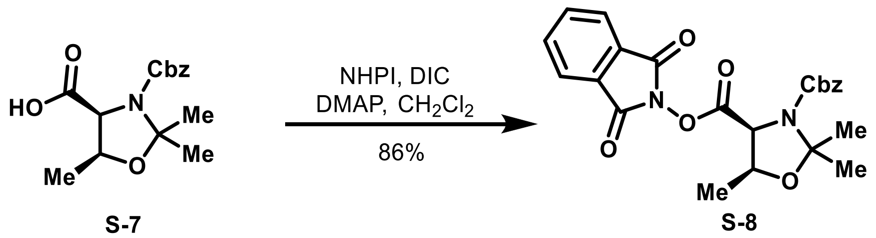

Acid **S-7** was prepared according to literature precedent.[3] The preparation of **RAE S-8** was carried out in analogy to **RAE 11 (*trans*)**.

Physical State: colorless oil.

R_f = 0.48 (hexanes/EtOAc 1:1, UV).

[α]$_D^{25}$ = -65.8 (c 1.0, $CHCl_3$).

Two rotamers in a ratio of 3.6:1 were detected by ^{1}H NMR.

Major rotamers:

^{1}H NMR (600 MHz, $CDCl_3$) δ 7.93 – 7.88 (m, 2H), 7.84 – 7.78 (m, 2H), 7.45 – 7.41 (m, 2H), 7.39 – 7.34 (m, 2H), 7.34 – 7.29 (m, 1H), 5.38 (d, *J* = 12.2 Hz, 1H), 5.07 (d, *J* = 12.2 Hz, 1H), 4.52 – 4.44 (m, 1H), 4.33 (d, *J* = 7.7 Hz, 1H), 1.71 (s, 3H), 1.63 (s, 3H), 1.56 (d, *J* = 6.0 Hz, 3H).

^{13}C NMR (151 MHz, $CDCl_3$) δ 167.6, 161.6, 151.5, 136.4, 135.1, 129.0, 128.6, 128.6, 128.2, 124.2, 96.3, 74.9, 67.4, 63.8, 26.6, 24.1, 18.8.

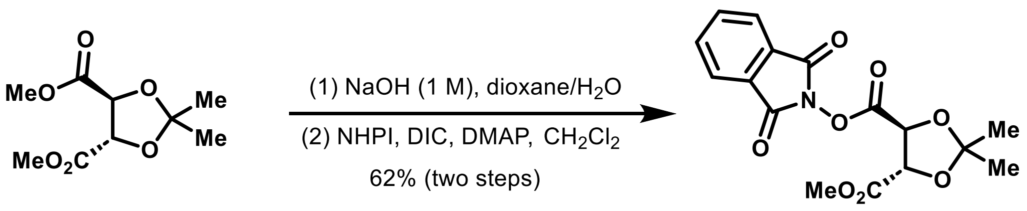

Dimethyl (4*S*,5*S*)-2,2-dimethyl -1,3-dioxolane-4,5-dicarboxylate

S-9

To a solution of **Dimethyl (4S,5S)-2,2-dimethyl-1,3-dioxolane-4,5-dicarboxylate** (3.0 g, 13.8 mmol, 1.0 equiv.) in dioxane (35 ml) and H_2O (35 ml) at rt, was added an aqueous 1M solution of NaOH (13,8 mL, 13.8 mmol, 1.0 equiv.) over 30 min. After stirring for 1 hour at rt, the reaction mixture was extracted twice with CH_2Cl_2 and the resulting organic phases were discarded. The aqueous phase was acidified to pH = 2-3 by addition of aqueous 1M HCl and CH_2Cl_2 was added. The layers were separated and the aqueous phase was extracted twice with CH_2Cl_2. The combined organic extracts were dried over Na_2SO_4, filtered and concentrated under reduced pressure to afford the crude monoacid.

The residue was dissolved in CH_2Cl_2 (69 mL) at rt and DMAP (183 mg, 1.38 mmol, 0.1 equiv.), NHPI (2.24 g, 13.8 mmol, 1.0 equiv.) and DIC (2.13 mL, 13.8 mmol, 1.0 equiv.) was added sequentially. The reaction flask was sealed and was allowed to stir at rt for 2 h. The resulting suspension was filtered over Celite (Et_2O) and the filtrate was

concentrated under vacuum. The residue was purified by flash chromatography (hexanes/EtOAc 5:1) on silica gel to afford the **S-9** (RAE) (2.97 g, 62%) as a white solid.

Physical State: white solid.

R_f = 0.52 (hexanes/EtOAc 2:1, UV).

$[\alpha]_D^{25}$ = +46.8 (c 1.0, $CHCl_3$).

^{1}H NMR (600 MHz, $CDCl_3$) δ 7.89 (dd, J = 5.5, 3.1 Hz, 2H), 7.81 (dd, J = 5.5, 3.0 Hz, 2H), 5.23 (d, J = 4.6 Hz, 1H), 5.07 (d, J = 4.7 Hz, 1H), 3.86 (s, 3H), 1.56 (d, J = 0.8 Hz, 3H), 1.54 (d, J = 0.8 Hz, 3H).

^{13}C NMR (151 MHz, $CDCl_3$) δ 169.5, 167.1, 161.5, 135.1, 128.9, 124.3, 115.3, 77.4, 75.4, 53.2, 26.6, 26.2.

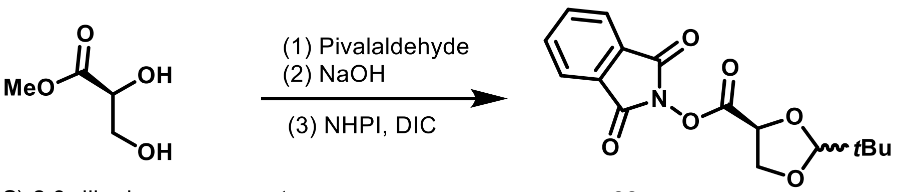

methyl (*S*)-2,3-dihydroxypropanoate

22 (*dr* = 1:1.5)

(The preparation of **22** (*RAE) was carried out in analogy to* **10** *(cis-Cbz-RAE), starting from methyl (S)-2,3-dihydroxypropanoate*)

Physical State: white solid.

R_f = 0.61 (hexanes/EtOAc1:1, UV).

Two diastereomer in a ratio of 1:1.3 were detected by NMR.

Minor diastereomer:

^{1}H NMR (600 MHz, $CDCl_3$) δ 7.90 -7.85 (m, 2H), 7.81 – 7.76 (m, 2H), 4.92 (dd, J = 7.7, 6.2 Hz, 1H), 4.83 (s, 1H), 4.45 (dd, J = 8.5, 7.5 Hz, 1H), 4.17 (dd, J = 8.5, 6.0 Hz, 1H), 0.95 (s, 9H).

^{13}C NMR (151 MHz, $CDCl_3$) δ 168.0, 161.6, 135.1, 128.9, 124.2, 112.3, 72.4, 68.5, 34.4, 24.2.

Major diastereomer:

^{1}H NMR (600 MHz, CDCl$_3$) δ 7.90 -7.85 (m, 2H), 7.81 – 7.76 (m, 2H), 4.91 (dd, *J* = 7.9, 3.1 Hz, 1H), 4.65 (s, 1H), 4.53 (dd, *J* = 9.0, 3.1 Hz, 1H), 4.20 (dd, *J* = 9.1, 7.9 Hz, 1H), 0.99 (s, 9H).

^{13}C NMR (151 MHz, CDCl$_3$) δ 167.4, 161.6, 135.0, 128.9, 124.2, 112.9, 71.9, 69.0, 34.0, 24.4.

Experimental Procedures and Characterization Data for Model Reaction

4-(2-(benzyloxy)ethoxy)-1-chloro-2-iodobenzene (12)

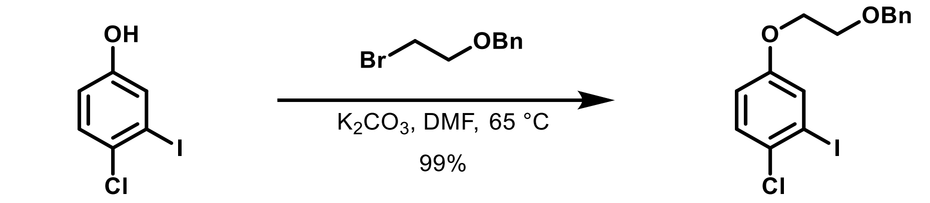

4-chloro-3-iodophenol **12**

To a solution of **4-chloro-3-iodophenol** (1.0 g, 3.9 mmol, 1.0 equiv.) in *N,N*-dimethylformamide (10 mL) was added potassium carbonate (1.1 g, 5.9 mmol, 2.0 equiv.), and stirring continued for additional 15 minutes prior to the addition of **benzyl 2-bromoethyl ether** (0. 89 g, 4.1 mmol, 1.05 equiv.). The mixture was submerged into a preheated oil bath at 65 °C. The reaction was stirred at ambient temperature for 8 hours at which point the TLC analysis indicated complete consumption of the starting material. The reaction was diluted with water (15 mL) and ether (30 mL), and the layers were separated. The aqueous layer was extracted with ether (30 mL), and the combined organic layers were washed with water (15 mL × 2) and brine (20 mL), dried over anhydrous Na_2SO_4, concentrated, and purified by silica gel column chromatography (hexanes/EtOAc = 10:1) to give the desired product **12** (1.5 g, 99%) as a colorless oil.

Physical State: colorless oil.

R$_f$ = 0.50 (hexanes/EtOAc 5:1, UV).

^{1}H NMR (600 MHz, CDCl$_3$) δ 7.41 (d, *J* = 2.9 Hz, 1H), 7.38 – 7.34 (m, 4H), 7.34 –

7.27 (m, 2H), 6.86 (dd, J = 8.8, 2.9 Hz, 1H), 4.62 (s, 2H), 4.15 – 4.07 (m, 2H), 3.84 – 3.77 (m, 2H).

^{13}C NMR (151 MHz, CDCl$_3$) δ 157.6, 138.0, 130.4, 129.4, 128.6, 127.9, 127.9, 126.1, 116.5, 98.0, 73.6, 68.4, 68.2.

EI: Calc'd for $C_{15}H_{14}ClIO_2$, $[M]^+$ 388; found 388, 237, 152, 91.

4-(2-(benzyloxy)ethoxy)-2-bromo-1-chlorobenzene (12-Br)

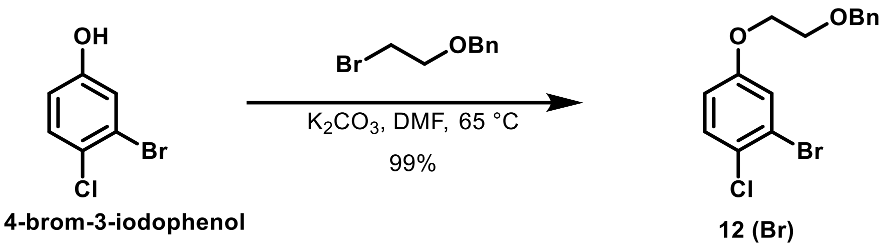

To a solution of **4-brom-3-iodophenol** (2.0 g, 9.7 mmol, 1.0 equiv.) in *N,N*-dimethylformamide (20 mL) was added potassium carbonate (2.7 g, 19.4 mmol, 2.0 equiv.), and stirring continued for additional 15 minutes prior to the addition of **benzyl 2-bromoethyl ether** (1.6 g, 10 mmol, 1.05 equiv.). The mixture was submerged into a preheated oil bath at 65 °C. The reaction was stirred at ambient temperature for 10 hours at which point the TLC analysis indicated the complete consumption of the starting material. The reaction was diluted with water (30 mL) and ether (60 mL), and the layers were separated. The aqueous layer was extracted with ether (60 mL), and the combined organic layers were washed with water (30 mL × 2) and brine (40 mL), dried over anhydrous Na_2SO_4, concentrated, and purified by silica gel column chromatography (hexanes/EtOAc = 10:1) to give the desired product **12 (Br)** (3.3 g, 99%) as a colorless oil.

Physical State: colorless oil.

R_f = 0.50 (hexanes/EtOAc 5:1, UV).

^{1}H NMR (600 MHz, CDCl$_3$) δ 7.40 – 7.29 (m, 6H), 7.20 (d, J = 2.9 Hz, 1H), 6.83 (dd, J = 8.8, 2.9 Hz, 1H), 4.63 (s, 2H), 4.22 – 4.04 (m, 2H), 3.98 – 3.57 (m, 2H).

^{13}C NMR (151 MHz, CDCl$_3$) δ 157.9, 137.9, 130.6, 128.6, 127.9, 127.9, 126.2, 122.6, 119.8, 115.5, 73.6, 68.4, 68.2.

EI: Calc'd for $C_{15}H_{14}ClBrO_2$, $[M]^+$ 340; found 340, 191, 105, 91.

Checking Stereochemical Erosion during Oxazolidine Removal

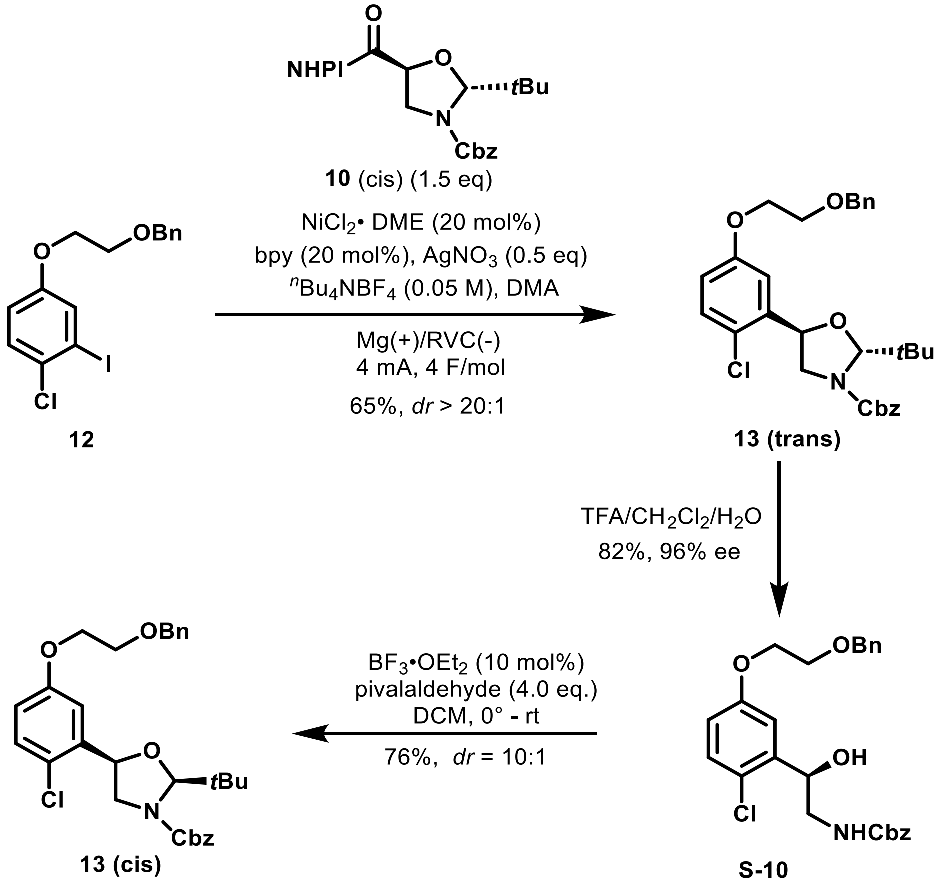

To make sure that the stereochemistry purity is maintained during oxazolidine removal, the ring-opened product **S-10** was subject to the condensation with pivalaldehyde to reform the oxazolidine, which allows facile analysis of the diastereomeric ratio. The result (**13-*cis*** with >10:1 *dr*) suggested that there was virtually no epimerization during downstream chemical treatment.

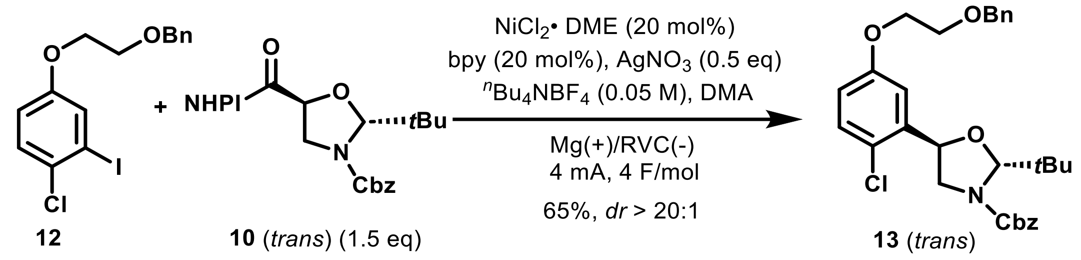

Following **General Procedure A** on 0.2 mmol scale. Purification by flash column chromatography (hexane/EA = 15:1) afforded the **13** (*trans*) (68.2 mg, 65% yield, *dr* >20:1).

Physical State: colorless oil.

***R*$_f$** = 0.47 (hexanes/EtOAc 5:1, UV).

[α]$_D^{25}$ = +2.6 (c 1.0, $CHCl_3$).

^{1}H NMR (600 MHz, $CDCl_3$) δ 7.49 – 7.23 (m, 8H), 7.22 (d, *J* = 8.7 Hz, 1H), 7.03 (s, 1H, br), 6.91 (d, *J* = 3.0 Hz, 1H), 6.78 (dd, *J* = 8.7, 3.0 Hz, 1H), 5.55 (s, 1H, br), 5.49 (dd, *J* = 7.3, 2.7 Hz, 1H), 5.08 (d, *J* = 12.2 Hz, 1H), 4.97 (s, 1H, br), 4.62 (s, 2H), 4.10 (dt, *J* = 10.0, 4.8 Hz, 1H), 4.02 (s, 1H, br), 4.00 (dd, *J* = 10.1, 4.8 Hz, 1H), 3.83 (dd, *J* = 11.5, 7.3 Hz, 1H), 3.79 (t, *J* = 4.8 Hz, 2H), 1.01 (s, 9H, br).

^{13}C NMR (151 MHz, $CDCl_3$) δ 157.9, 155.5, 140.8, 138.0, 136.3 (br), 130.2, 128.5, 127.84 (s and br, 2C), 127.78, 127.3 (br), 122.3 (br), 115.3 (br), 112.2 (br), 97.3 (br), 76.3 (br), 73.4, 68.4, 67.75, 67.70, 52.0, 38.2, 25.4.

HRMS: Calc'd for $C_{30}H_{35}ClNO_5$, $[M+H]^+$ 524.2199; found 524.2208.

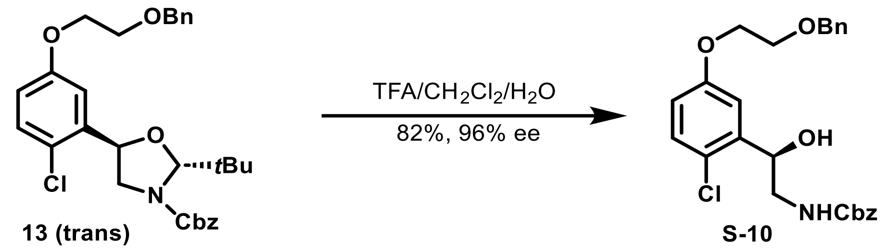

To a solution of **13** (*trans*) (20 mg, 38.2 μmol, 1.0 equiv.) in CH_2Cl_2 (1.0 mL) was added H_2O (0.1 mL) and trifluoroacetic acid (TFA) (0.5 mL). The reaction was stirred vigorously for 24 h at room temperature. The solvent was removed under reduced pressure. The crude residue was purified by SiO_2 flash chromatography (eluent: hexanes/EtOAc = 4/1) to give **chiral alcohol S-10** (14.3 mg, 82%, 96% ee) as a colorless oil. (*Racemic compound was obtained following the similar procedure, using an acetonide as the protecting group for the aminoalcohol moiety*)

The enantiomeric purity was determined by HPLC analysis on a CHIRALPAK® IC column (30% IPA/hexanes, 1.0 mL/min) with retention time 9.22 min (major) and 11.63 min (minor).

Physical State: colorless oil.

R_f = 0.42 (hexanes/EtOAc 2:1, UV).

$[\alpha]_D^{25}$ = -37.8 (c 1.0, $CHCl_3$).

^{1}H NMR (600 MHz, $CDCl_3$) δ 7.41 – 7.26 (m, 10H), 7.23 – 7.13 (m, 2H), 6.78 (dd, J = 8.9, 3.0 Hz, 1H), 5.28 (s, 1H), 5.19 – 5.01 (m, 3H), 4.61 (d, J = 1.7 Hz, 2H), 4.17 – 4.05 (m, 2H), 3.77 -3.81 (m, 2H), 3.65 – 3.52 (m, 1H), 3.37 – 3.18 (m, 1H, -NH-).

^{13}C NMR (151 MHz, $CDCl_3$) δ 158.0, 157.8, 140.0, 138.0, 136.4, 130.2, 128.7, 128.6, 128.3, 128.2, 127.9, 123.2, 115.6, 113.5, 73.5, 71.0, 68.5, 67.8, 67.2, 46.9.

HRMS: Calc'd for $C_{25}H_{27}ClNO_5$, $[M+H]^+$ 456.1573; found 456.1577.

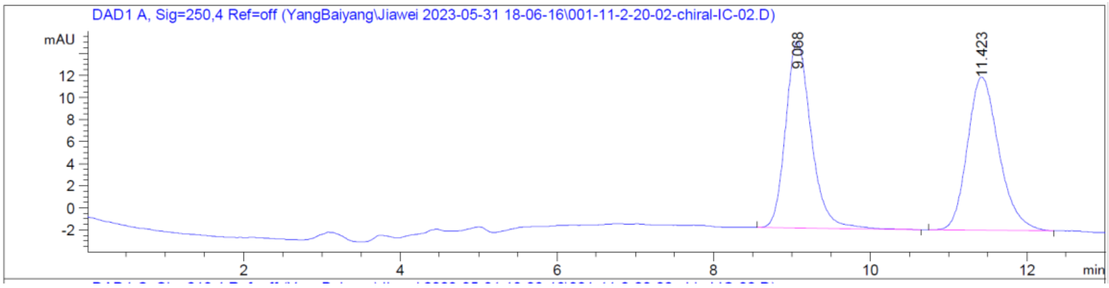

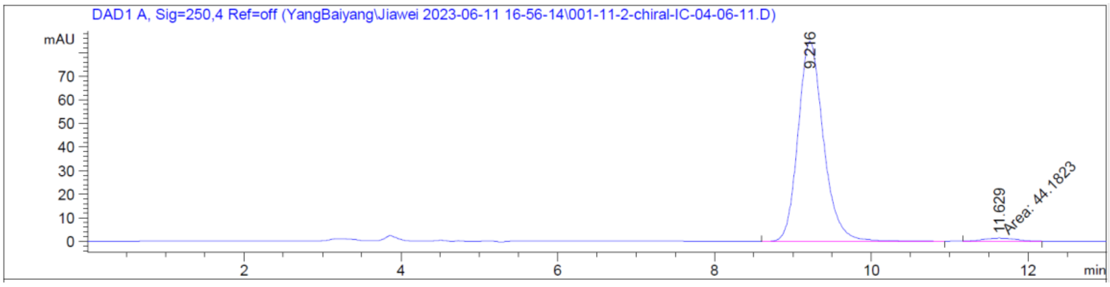

Signal 1: DAD1 A, Sig=250,4 Ref=off

Peak #	RetTime [min]	Type	Width [min]	Area [mAU*s]	Height [mAU]	Area %
1	9.068	BB	0.3446	383.85104	16.92480	49.6918
2	11.423	BB	0.4284	388.61176	13.89055	50.3082
Totals :				772.46280	30.81535	

Signal 1: DAD1 A, Sig=250,4 Ref=off

Peak #	RetTime [min]	Type	Width [min]	Area [mAU*s]	Height [mAU]	Area %
1	9.216	BB	0.3436	1919.09790	84.93749	97.7496
2	11.629	MM	0.5013	44.18227	1.46881	2.2504
Totals :				1963.28017	86.40630	

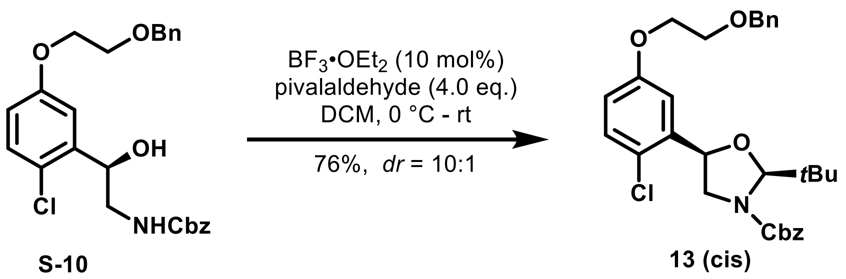

To a solution of **chiral alcohol S-10** (100 mg, 220 μmol, 1.0 equiv.) and pivalaldehyde (85 μL, 879 μmol, 4.0 equiv.) in anhydrous CH_2Cl_2 (2.0 mL) was added $BF_3{\cdot}OEt_2$ (2.4 μL, 22 μmol, 0.1 equiv.). The reaction was warmed to ambient temperature and stirred for 4 h. The reaction was quenched by the addition of Et_3N (0.1 mL) and *sat. aq.* $NaHCO_3$ (2 mL) and diluted with CH_2Cl_2 (5 mL). The layers were separated and the aqueous layer was extracted with CH_2Cl_2 (2 × 50 mL). The combined layers were washed with brine, dried over Na_2SO_4, filtered and concentrated *in vacuo*. The crude residue was purified by SiO_2 flash chromatography (eluent: hexanes/EtOAc = 15/1) to give colorless oil **13 (*cis*)** [87 mg, *dr* = 10(*cis*):1(*trans*)] as an inseparable mixture.

Physical State: colorless oil.

R_f = 0.47 (hexanes/EtOAc 5:1, UV).

$[\alpha]_D^{25}$ = -22.2 (c 1.0, $CHCl_3$).

^{1}H NMR (600 MHz, $CDCl_3$) δ 7.42 – 7.32 (m, 9H), 7.32 – 7.27 (m, 2H), 7.23 (d, *J* = 9.0 Hz, 1H), 6.81 (dd, *J* = 8.8, 3.1 Hz, 1H), 5.25 (s, 1H), 5.20 (d, *J* = 2.0 Hz, 2H), 5.00 (dd, *J* = 10.1, 5.2 Hz, 1H), 4.67 (s, 1H, br), 4.63 (s, 2H), 4.14 (t, *J* = 4.8 Hz, 2H), 3.83 (t, *J* = 4.8 Hz, 2H), 2.88 – 2.76 (m, 1H), 1.01 (s, 9H).

^{13}C NMR (151 MHz, $CDCl_3$) δ 158.0, 155.9, 138.0, 136.9, 130.3, 128.7, 128.6, 128.6, 128.4, 127.9, 127.9, 123.3, 115.4, 113.1, 95.7, 75.7, 73.6, 68.5, 67.9, 66.0, 51.6, 37.7, 25.7.

HRMS: Calc'd for $C_{30}H_{35}ClNO_5$, $[M+H]^+$ 524.2199; found 524.2205.

Experimental Procedures and Characterization Data for Decarboxylative Coupling Products

benzyl (2*R*,5*R*)-2-(*tert*-butyl)-5-phenyloxazolidine-3-carboxylate (17a)

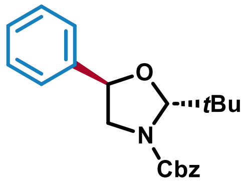

Following **General Procedure A** on 0.2 mmol scale with **10** (*trans*-Cbz-RAE). Purification by flash column chromatography (hexane/EA = 20:1) afforded the title compound **17a** (48.0 mg, 71% yield, *dr* >20:1).

Physical State: white needle solid.

R_f = 0.50 (hexanes/EtOAc 10:1, UV).

$[\alpha]_D^{25}$ = +27.6 (c 1.0, $CHCl_3$).

^{1}H NMR (600 MHz, $CDCl_3$) δ 7.44 – 7.24 (m, 6H), 7.23 – 7.03 (m, 4H, br), 5.44 (s, 1H, br), 5.27 (dd, *J* = 7.0, 2.6 Hz, 1H), 5.20 – 4.88 (m, 1H, br), 4.06 (s, 1H, br), 3.72 (dd, *J* = 11.1, 7.0 Hz, 1H), 1.00 (s, 9H).

^{1}H NMR (600 MHz, CD_3OD) δ 7.42 – 7.16 (m, 9H), 7.03 (s, 1H, br), 5.38 (s, 1H), 5.29 (dd, *J* = 6.8, 1.9 Hz, 1H), 5.06 (d, *J* = 11.2 Hz, 1H), 4.99 – 4.84 (s, 1H, br), 4.08 (dd, *J* = 11.3, 1.8 Hz, 1H), 3.72 (dd, *J* = 11.3, 6.8 Hz, 1H), 0.98 (s, 9H).

^{13}C NMR (151 MHz, $CDCl_3$) δ 155.8, 142.0, 128.8, 128.6, 128.1 (br), 127.9, 125.3, 52.8, 38.2, 25.5.

^{13}C NMR (151 MHz, CD_3OD) δ 157.4, 143.5, 129.6, 129.5, 128.7, 126.0, 53.9, 38.8, 25.8.

HRMS: Calc'd for $C_{21}H_{26}NO_3$, $[M+H]^+$ 340.1908; found 340.1919.

benzyl (2*R*,5*R*)-5-([1,1'-biphenyl]-2-yl)-2-(*tert*-butyl)oxazolidine-3-carboxylate (17b)

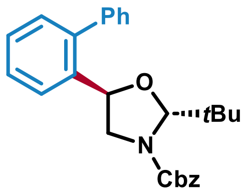

Following **General Procedure A** on 0.2 mmol scale with **10** (*trans*-Cbz-RAE). Purification by flash column chromatography (hexane/EA = 20:1) afforded the title compound **17b** (45.8 mg, 55% yield, *dr* >20:1).

Physical State: colorless oil.

R_f = 0.50 (hexanes/EtOAc 10:1, UV).

[α]$_D^{25}$ = -37.4 (c 1.0, $CHCl_3$).

^{1}H NMR (600 MHz, $CDCl_3$) δ 7.46 – 7.04 (m, 14H), 5.50 (s, 1H, br), 5.43 – 5.29 (m, 1H, br), 5.12 (d, *J* = 10.7 Hz, 1H), 4.97 (s, 1H, br), 4.04 – 3.76 (m, 1H, br), 3.51 – 3.36 (m, 1H), 0.91 (s, 9H).

^{13}C NMR (151 MHz, $CDCl_3$) δ 155.7, 140.9, 130.3, 129.4 (br), 128.7, 128.5, 128.1 (br), 127.6, 127.4, 125.1 (br), 52.8, 38.4, 25.5.

HRMS: Calc'd for $C_{27}H_{30}NO_3$, $[M+H]^+$ 416.2221; found 416.2226.

benzyl (2*R*,5*R*)-2-(*tert*-butyl)-5-(4-(4,4,5,5-tetramethyl-1,3,2-dioxaborolan-2-yl)phenyl)oxazolidine-3-carboxylate (17c)

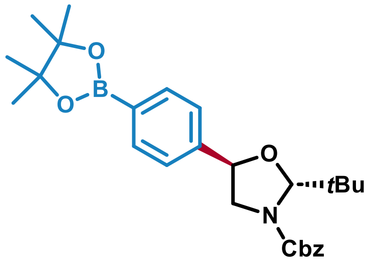

Following **General Procedure A** on 0.2 mmol scale with **10** (*trans*-Cbz-RAE). Purification by flash column chromatography (hexane/EA = 15:1) afforded the title

compound **17c** (51.3 mg, 55% yield, *dr* >20:1).

Physical State: white solid.

R_f = 0.51 (hexanes/EtOAc 6:1, UV).

$[\alpha]_D^{25}$ = +40.8 (c 1.0, $CHCl_3$).

^{1}H NMR (600 MHz, $CDCl_3$) δ 7.74 (d, *J* = 8.1 Hz, 2H), 7.39 – 7.21 (m, 4H, br), 7.21 (d, *J* = 7.9 Hz, 2H), 7.03 (s, 1H, br), 5.44 (s, 1H), 5.28 (dd, *J* = 7.0, 2.4 Hz, 1H), 5.17 – 4.85 (m, 2H, br), 4.04 (s, 1H, br), 3.71 (dd, *J* = 11.1, 7.0 Hz, 1H), 1.35 (s, 12H), 0.99 (s, 9H).

^{13}C NMR (151 MHz, $CDCl_3$) δ 155.8, 135.3, 128.6, 128.0 (br), 124.4, 83.9, 52.9, 38.2, 25.5, 25.05, 25.01.

HRMS: Calc'd for $C_{27}H_{37}BNO_5$, $[M+H]^+$ 465.2796; found 465.2803.

benzyl (2*R*,5*R*)-2-(*tert*-butyl)-5-(2-(methylthio)phenyl)oxazolidine-3-carboxylate (17d)

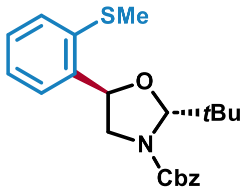

Following **General Procedure A** on 0.2 mmol scale with **10** (*trans*-Cbz-RAE). Purification by flash column chromatography (hexane/EA = 20:1) afforded the title compound **17d** (40.0 mg, 51% yield, *dr* >20:1).

Physical State: colorless oil.

R_f = 0.43 (hexanes/EtOAc 10:1, UV).

$[\alpha]_D^{25}$ = -31.1 (c 1.0, $CHCl_3$).

^{1}H NMR (600 MHz, $CDCl_3$) δ 7.42 – 7.17 (m, 7H), 7.11 (s, 1H, br), 7.02 – 6.92 (s, 1H, br), 5.62 – 5.46 (s, 1H, br), 5.56 (dd, *J* = 7.3, 2.7 Hz, 1H), 5.07 (d, *J* = 11.9 Hz, 1H), 4.93 (s, 1H, br), 4.00 (s, 1H), 3.78 (dd, *J* = 11.3, 7.2 Hz, 1H), 2.45 (s, 3H), 1.01 (s, 9H).

^{13}C NMR (151 MHz, $CDCl_3$) δ 155.8, 140.5, 136.5 (br), 128.6, 128.2, 127.3 (br), 126.6

(br), 125.6 (br), 124.8 (br), 97.4 (br), 67.1 (br), 52.2, 38.3, 25.5, 16.4.

HRMS: Calc'd for $C_{22}H_{28}NO_3S$, $[M+H]^+$ 386.1785; found 386.1791.

benzyl (2R,5R)-2-(tert-butyl)-5-(2-cyanophenyl)oxazolidine-3-carboxylate (17e)

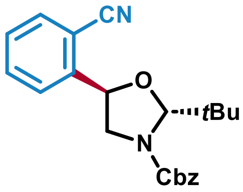

Following **General Procedure A** on 0.2 mmol scale with **10** (*trans*-Cbz-RAE). Purification by flash column chromatography (hexane/EA = 15:1) afforded the title compound **17e** (54.6 mg, 75% yield, *dr* >20:1).

Physical State: white solid.

R_f = 0.45 (hexanes/EtOAc 6:1, UV).

$[\alpha]_D^{25}$ = -37.0 (c 1.0, $CHCl_3$).

^{1}H NMR (600 MHz, $CDCl_3$) δ 7.62 (d, *J* = 7.7 Hz, 1H), 7.47 (t, *J* = 7.7 Hz, 1H), 7.43 – 7.17 (m, 6H), 7.06 (s, 1H, br), 5.59 (dd, *J* = 7.3, 2.6 Hz, 1H), 5.55 (s, 1H, br), 5.09 (d, *J* = 12.0 Hz, 1H), 5.06 – 4.86 (s, 1H, br), 4.09 (s, 1H, br), 3.89 (dd, *J* = 11.6, 7.3 Hz, 1H), 1.01 (s, 9H).

^{13}C NMR (151 MHz, $CDCl_3$) δ 155.6, 146.4, 136.2, 133.3, 128.6, 128.2, 125.7, 117.3, 97.6, 76.8 (br), 52.5, 38.3, 25.4.

HRMS: Calc'd for $C_{22}H_{24}N_2O_3Na$, $[M+H]^+$ 387.1680; found 387.1685.

benzyl (2*R*,5*R*)-2-(*tert*-butyl)-5-(3,4-difluorophenyl)oxazolidine-3-carboxylate (17f)

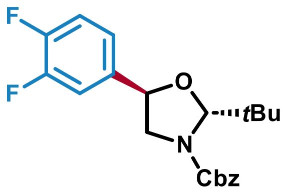

Following **General Procedure A** on 0.2 mmol scale with **10** (*trans*-Cbz-RAE).

Purification by flash column chromatography (hexane/EA = 15:1) afforded the title compound **17f** (57.1 mg, 76% yield, *dr* >20:1).

Physical State: light yellow solid.

***R**f* = 0.44 (hexanes/EtOAc 10:1, UV).

[α]$_D$^{25} = +38.1 (c 1.0, $CHCl_3$).

^{1}H NMR (600 MHz, $CDCl_3$) δ 7.30 (s, 3H, br), 7.15 (s, 2H, br), 7.06 – 6.96 (m, 2H), 6.92- 6.84 (m, 1H), 5.39 (s, 1H, br), 5.19 (d, *J* = 7.1 Hz, 1H), 5.19 -4.80 (m, 2H, br), 4.25 -3.90 (m, 1H, br), 3.68 (dd, *J* = 11.3, 6.8 Hz, 1H), 0.98 (s, 9H, br).

^{13}C NMR (151 MHz, $CDCl_3$) δ 155.7, 151.0 (dd, *J* = 249.2, 12.7 Hz), 149.9 (dd, *J* = 248.2, 12.7 Hz), 139.1 (dd, *J* = 4.4, 3.5 Hz), 136.2 (br), 128.6, 128.3, 128.0 (br), 121.1 (br), 117.5 (d, *J* = 17.4 Hz), 114.4 (d, *J* = 17.6 Hz), 96.7 (br), 67.5 (br), 52.7, 38.1, 25.4.

HRMS: Calc'd for $C_{21}H_{24}F_2NO_3$, $[M+H]^+$ 376.1719; found 376.1726.

benzyl (2*R*,5*R*)-2-(*tert*-butyl)-5-(3-hydroxyphenyl)oxazolidine-3-carboxylate (17g)

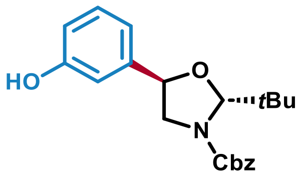

Following **General Procedure A** on 0.2 mmol scale with **10** (*trans*-Cbz-RAE). Purification by flash column chromatography (hexane/EA = 15:1) afforded the title compound **17g** (23.9 mg, 36% yield, *dr* >20:1).

Physical State: colorless oil.

***R**f* = 0.41 (hexanes/EtOAc 4:1, UV).

[α]$_D$^{25} = +28.6 (c 1.0, $CHCl_3$).

^{1}H NMR (600 MHz, $CDCl_3$) δ 7.29 (s, 3H, br), 7.22 – 7.02 (m, 2H, br), 7.16 (t, *J* = 7.8 Hz, 1H), 6.78 – 6.71 (m, 2H), 6.64 (s, 1H), 5.42 (s, 1H, br), 5.20 (d, *J* = 5.8 Hz, 1H), 5.16 -4.85 (m, 2H, br), 4.21 – 3.94 (m, 1H, br), 3.69 (dd, *J* = 11.2, 7.0 Hz, 1H), 0.99 (s, 9H, br).

^{13}C NMR (151 MHz, $CDCl_3$) δ 156.1 (br), 155.9 (br), 144.0, 136.4, 130.1, 128.7, 128.2 (br), 117.7, 114.9, 112.0, 96.9 (br), 67.4 (br), 52.7, 38.2, 25.5.

HRMS: Calc'd for $C_{21}H_{26}NO_4$, $[M+H]^+$ 356.1857; found 356.1861

benzyl (2*R*,5*R*)-2-(*tert*-butyl)-5-(4-morpholinophenyl)oxazolidine-3-carboxylate (17h)

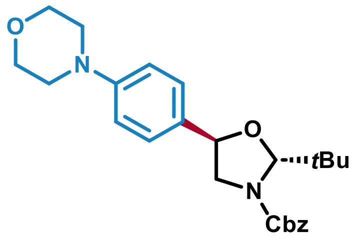

Following **General Procedure A** on 0.2 mmol scale with **10** (*trans*-Cbz-RAE). Purification by flash column chromatography (hexane/EA = 12:1) afforded the title compound **17h** (38.4 mg, 45% yield).

Physical State: white solid.

$\boldsymbol{R_f}$ = 0.40 (hexanes/EtOAc 4:1, UV).

$[\alpha]_D^{25}$ = +36.7 (c 1.0, $CHCl_3$).

^{1}H NMR (600 MHz, $CDCl_3$) δ 7.30 (s, 3H, br), 7.25 – 7.13 (m, 2H, br), 7.10 (d, *J* = 8.6 Hz, 2H), 6.81 (d, *J* = 8.7 Hz, 2H), 5.38 (s, 1H, br), 5.20 (dd, *J* = 6.9, 2.7 Hz, 1H), 5.16 – 4.96 (m, 2H, br), 4.20 -3.93 (m, 1H, br), 3.92 – 3.80 (m, 4H), 3.68 (dd, *J* = 11.1, 6.9 Hz, 1H), 3.19 – 3.07 (m, 4H), 0.98 (s, 9H, br).

^{13}C NMR (151 MHz, $CDCl_3$) δ 155.8, 151.0, 136.5 (br), 133.1, 128.6, 128.1 (br), 126.4, 115.8, 67.0, 52.7, 49.4, 38.3, 25.6.

HRMS: Calc'd for $C_{25}H_{33}N_2O_4$, $[M+H]^+$ 425.2435; found 425.2437.

benzyl (2*R*,5*R*)-2-(*tert*-butyl)-5-(4-formylphenyl)oxazolidine-3-carboxylate (17i)

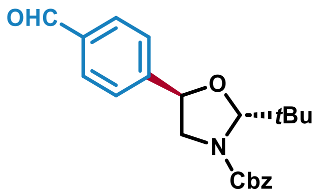

Following **General Procedure A** on 0.2 mmol scale with **10** (*trans*-Cbz-RAE). Purification by flash column chromatography (hexane/EA = 12:1) afforded the title compound **17i** (42.1 mg, 57% yield, *dr* >20:1).

Physical State: light yellow solid.

R_f = 0.57 (hexanes/EtOAc 5:1, UV).

$[\alpha]_D^{25}$ = +35.6 (c 1.0, $CHCl_3$).

^{1}H NMR (600 MHz, $CDCl_3$) δ 9.98 (s, 1H), 7.77 (d, *J* = 8.1 Hz, 2H), 7.35 (d, *J* = 7.9 Hz, 2H), 7.32 – 6,98 (m, 5H, br), 5.46 (s, 1H, br), 5.31 (dd, *J* = 7.0, 2.2 Hz, 1H), 5.25 - 4.84 (m, 2H, br), 4.23 – 3.97 (m, 1H, br), 3.74 (dd, *J* = 11.4, 7.0 Hz, 1H), 1.00 (s, 9H, br).

^{13}C NMR (151 MHz, $CDCl_3$) δ 191.9, 155.7, 148.9, 136.2, 136.0, 130.2, 128.6, 128.3 (br), 125.8, 52.6, 38.1, 25.4.

HRMS: Calc'd for $C_{22}H_{26}NO_4$, $[M+H]^+$ 368.1857; found 368.1853.

benzyl (2*R*,5*R*)-2-(*tert*-butyl)-5-(pyridin-3-yl)oxazolidine-3-carboxylate (17j)

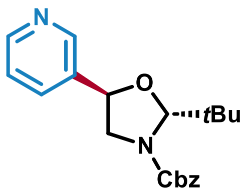

Following **General Procedure A** on 1.0 mmol scale (12 mA) with **10** (*trans*-Cbz-RAE). Purification by flash column chromatography (hexane/EA = 6:1) afforded the title compound **17j** (273 mg, 80% yield, *dr* >20:1).

Physical State: white solid.

R_f = 0.50 (hexanes/EtOAc 2:1, UV).

[α]$_D^{25}$ = +41.1 (c 1.0, $CHCl_3$).

^{1}H NMR (600 MHz, $CDCl_3$) δ 8.57 -8.45 (m, 2H), 7.48 (dd, *J* = 8.0, 2.4 Hz, 1H), 7.39 – 7.26 (m, 3H, br), 7.26 – 7.08 (m, 2H, br), 7.18 (dd, *J* = 7.9, 4.9 Hz, 1H), 5.41 (s, 1H, br), 5.30 (dd, *J* = 7.0, 2.5 Hz, 1H), 5.09 (d, *J* = 11.5 Hz, 1H), 5.07 – 4.92 (s, 1H, br), 4.22 – 3.98 (m, 1H, br), 3.75 (dd, *J* = 11.3, 7.0 Hz, 1H), 0.99 (s, 9H).

^{13}C NMR (151 MHz, $CDCl_3$) δ 155.6, 149.4, 147.4, 137.3, 136.2, 132.9, 128.7, 128.3, 128.1 (br), 123.7, 96.9 (br), 67.7 (br), 52.4, 38.2, 25.5.

HRMS: Calc'd for $C_{20}H_{25}N_2O_3$, $[M+H]^+$ 341.1860; found 341.1870.

benzyl (2*R*,5*S*)-2-(*tert*-butyl)-5-(pyridin-2-yl)oxazolidine-3-carboxylate (17k)

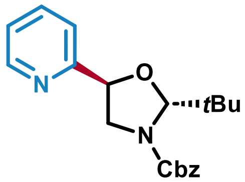

Following **General Procedure A** on 0.2 mmol scale with **10** (*trans*-Cbz-RAE). Purification by flash column chromatography (hexane/EA = 10:1) afforded the title compound **17k** (41.7 mg, 61% yield, *dr* =20:1).

Physical State: white solid.

R_f = 0.51 (hexanes/EtOAc 4:1, UV).

[α]$_D^{25}$ = +35.5 (c 1.0, $CHCl_3$).

^{1}H NMR (600 MHz, $CDCl_3$) δ 8.48 (d, *J* = 4.5 Hz, 1H), 7.60 (t, *J* = 7.6 Hz, 1H), 7.40 – 7.21 (m, 5H), 7.20 – 6.99 (m, 2H), 5.44 (d, *J* = 1.1 Hz, 1H), 5.28 (dd, *J* = 7.1, 2.0 Hz, 1H), 5.06 (d, *J* = 11.8 Hz, 1H), 5.00 - 4.91 (m, 1H, br), 4.37 (dd, *J* = 11.3, 2.0 Hz, 1H), 3.72 (dd, *J* = 11.3, 7.0 Hz, 1H), 1.00 (s, 9H).

^{13}C NMR (151 MHz, $CDCl_3$) δ 155.9, 149.5 (br), 136.9 (br), 136.5 (br), 128.6, 128.0 (br), 122.6, 119.8 (br), 96.7, 79.2 (br), 67.2 (br), 51.6 (br), 38.0, 25.4.

HRMS: Calc'd for $C_{20}H_{25}N_2O_3$, $[M+H]^+$ 341.1860; found 341.1868.

benzyl (2*R*,5*R*)-2-(*tert*-butyl)-5-(pyrimidin-5-yl)oxazolidine-3-carboxylate (17l)

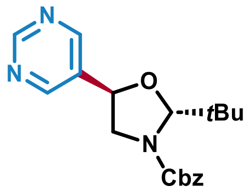

Following **General Procedure A** on 0.2 mmol scale with **10** (*trans*-Cbz-RAE). Purification by flash column chromatography (hexane/EA = 8:1) afforded the title compound **17l** (35.4 mg, 52% yield, *dr* >20:1).

Physical State: white solid.

R_f = 0.50 (hexanes/EtOAc 2:1, UV).

$[\alpha]_D^{25}$ = +49.3 (c 1.0, $CHCl_3$).

^{1}H NMR (600 MHz, $CDCl_3$) δ 9.15 (s, 1H), 8.62 (s, 2H), 7.40 – 7.28 (m, 3H), 7.21 (s, 2H, br), 5.40 (s, 1H, br), 5.30 (dd, *J* = 7.0, 2.4 Hz, 1H), 5.10 (d, *J* = 12.0 Hz, 1H), 5.07 – 5.0 (m, 1H, br), 4.35 – 3.98 (s, 1H, br), 3.78 (ddd, *J* = 11.5, 7.0, 0.8 Hz, 1H), 0.98 (s, 9H).

^{13}C NMR (151 MHz, $CDCl_3$) δ 158.5, 154.5, 134.9, 128.7, 128.5, 51.9, 38.2, 25.4.

HRMS: Calc'd for $C_{19}H_{24}N_3O_3$, $[M+H]^+$ 342.1813; found 342.1821.

benzyl (2*R*,5*S*)-2-(*tert*-butyl)-5-(thiophen-2-yl)oxazolidine-3-carboxylate (17m)

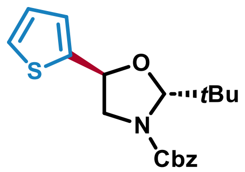

Following **General Procedure A** on 0.2 mmol scale with **10** (*trans*- Cbz-RAE). Purification by flash column chromatography (hexane/EA = 20:1) afforded the title compound **17m** (31.9 mg, 46% yield, *dr* >20:1).

Physical State: white solid.

R_f = 0.56 (hexanes/EtOAc 10:1, UV).

$[\alpha]_D^{25}$ = +81.6 (c 1.0, $CHCl_3$).

^{1}H NMR (600 MHz, CDCl$_3$) δ 7.37-7.28 (m, 3H, br), δ 7.27-7.16 (m, 3H, br), 7.08 (s, 1H, br), 6.88 (s, 1H, br), 5.32 (s, 1H, br), 5.29 (dd, *J* = 6.6, 2.0 Hz, 1H), 5.23 – 4.90 (m, 2H, br), 4.34 – 3.98 (m, 1H, br), 3.62 (dd, *J* = 11.2, 6.6 Hz, 1H), 0.97 (s, 9H, br).

^{13}C NMR (151 MHz, CDCl$_3$) δ 142.8 (br), 128.7, 128.2, 126.9, 125.4 (br) 121.4, 96.1 (br), 52.1, 38.0, 25.5.

HRMS: Calc'd for $C_{19}H_{24}NO_3S$, $[M+H]^+$ 346.1472; found 346.1478.

benzyl (2*R*,5*S*)-5-(benzo[b]thiophen-2-yl)-2-(*tert*-butyl)oxazolidine-3-carboxylate (17n)

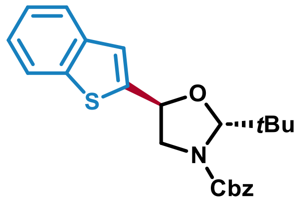

Following **General Procedure A** on 0.2 mmol scale with **10** (*trans*-Cbz-RAE). Purification by flash column chromatography (hexane/EA = 20:1) afforded the title compound **17n** (30.3 mg, 38% yield, *dr* >20:1).

Physical State: white solid.

R$_f$ = 0.44 (hexanes/EtOAc 10:1, UV).

$[\alpha]_D^{25}$ = +86.0 (c 1.0, CHCl$_3$).

^{1}H NMR (600 MHz, CDCl$_3$) δ 7.75 (d, *J* = 7.7 Hz, 1H), 7.66 (d, *J* = 7.9 Hz, 1H), 7.37 – 7.28 (m, 2H), 7.28 – 7.05 (m, 6H), 5.51 (d, *J* = 6.6, 1H), 5.49 – 4.90 (m, 3H, br), 4.50-4.15 (m, 1H, br), 3.70 (dd, *J* = 11.4, 6.5 Hz, 1H), 0.99 (s, 9H, br).

^{13}C NMR (151 MHz, CDCl$_3$) δ 145.8, 139.5, 128.5, 128.2, 124.5, 124.5, 123.8, 122.6, 120.8, 52.4, 37.9, 25.4.

HRMS: Calc'd for $C_{23}H_{26}NO_3S$, $[M+H]^+$ 396.1628; found 396.1635.

benzyl (2*R*,5*R*)-2-(*tert*-butyl)-5-(1-chloroisoquinolin-4-yl)oxazolidine-3-carboxylate (17o)

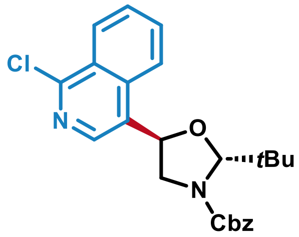

Following **General Procedure A** on 0.2 mmol scale with **10** (*trans*-Cbz-RAE). Purification by flash column chromatography (hexane/EA = 15:1) afforded the title compound **17o** (42.4 mg, 50% yield, *dr* >20:1).

Physical State: colorless oil.

R_f = 0.58 (hexanes/EtOAc 5:1, UV).

$[\alpha]_D^{25}$ = -30.9 (c 1.0, $CHCl_3$).

^{1}H NMR (600 MHz, $CDCl_3$) δ 8.41 (dt, *J* = 8.4, 1.1 Hz, 1H), 8.23 (d, *J* = 1.0 Hz, 1H), 7.78 – 7.73 (m, 2H), 7.71 (ddd, *J* = 8.2, 5.5, 2.6 Hz, 1H), 7.40 – 6.85 (m, 5H, br), 5.94 – 5.81 (m, 1H), 5.56 (s, 1H, br), 5.07 (d, *J* = 11.9 Hz, 1H), 4.92 (s, 1H, br), 4.30 – 4.10 (m, 1H, br), 3.94 (dd, *J* = 11.2, 7.4 Hz, 1H), 1.13 – 0.94 (m, 9H, br).

^{13}C NMR (151 MHz, $CDCl_3$) δ 152.2, 134.8, 131.6, 128.6 (br), 128.4, 127.7, 126.6, 122.6, 51.9, 38.4, 25.5.

HRMS: Calc'd for $C_{24}H_{26}ClN_2O_3$, $[M+H]^+$ 425.1627; found 425.1631.

benzyl (2*R*,5*R*)-2-(*tert*-butyl)-5-(4-chloroquinolin-6-yl)oxazolidine-3-carboxylate (17p)

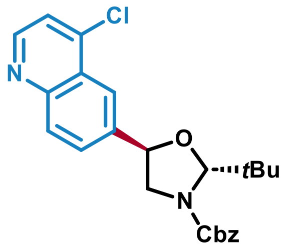

Following **General Procedure A** on 0.2 mmol scale with **10** (*trans*-Cbz-RAE).

Purification by flash column chromatography (hexane/EA = 10:1) afforded the title compound **17p** (49.2 mg, 58% yield, *dr* >20:1).

Physical State: colorless oil.

***R**$_f$* = 0.37 (hexanes/EtOAc 4:1, UV).

[α]$_D$^{25} = +59.1 (c 1.0, $CHCl_3$).

^{1}H NMR (600 MHz, $CDCl_3$) δ 8.78 (t, *J* = 4.3 Hz, 1H), 8.11 – 8.01 (m, 2H), 7.58 (ddd, *J* = 8.7, 4.1, 2.0 Hz, 1H), 7.50 (t, *J* = 4.4 Hz, 1H), 7.31 – 6.87 (m, 5H, br), 5.55 (s, 1H, br), 5.48 (dd, *J* = 7.1, 3.2 Hz, 1H), 5.18 – 4.82 (m, 2H, br), 4.17 (s, 1H, br), 3.83 (ddd, *J* = 11.4, 7.1, 4.0 Hz, 1H), 1.04 (s, 9H, br).

^{13}C NMR (151 MHz, $CDCl_3$) δ 155.7, 150.0, 148.9, 142.9, 141.6, 136.1, 130.7, 128.5, 128.2 (br), 127.8 (br), 126.4, 121.8, 120.4, 97.1, 52.7, 38.3, 25.5.

HRMS: Calc'd for $C_{24}H_{26}ClN_2O_3$, $[M+H]^+$ 425.1627; found 425.1635.

benzyl (2*R*,5*R*)-2-(tert-butyl)-5-(9H-carbazol-3-yl)oxazolidine-3-carboxylate (17q)

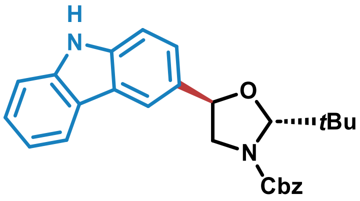

Following **General Procedure A** on 0.2 mmol scale with **10** (*trans*-Cbz-RAE). Purification by flash column chromatography (hexane/EA = 10:1) afforded the title compound **17q** (30.8 mg, 36% yield, *dr* >20:1).

Physical State: white solid.

***R**$_f$* = 0.57 (hexanes/EtOAc 4:1, UV).

[α]$_D$^{25} = +47.2 (c 1.0, $CHCl_3$).

^{1}H NMR (600 MHz, $CDCl_3$) δ 8.07 (s, 1H), 8.02 (d, *J* = 7.8 Hz, 1H), 7.93 (s, 1H), 7.47 – 7.38 (m, 2H), 7.34 (d, *J* = 8.4 Hz,1H), 7.26 – 7.20 (m, 3H), 7.08 (s, 4H, br), 5.54 (s, 1H, br), 5.47 (dd, *J* = 7.0, 2.9 Hz, 1H), 5.08 (d, *J* = 11.7 Hz, 1H), 5.06 – 4.91 (m, 1H, br), 4.32 – 4.04 (m, 1H, br), 3.81 (dd, *J* = 11.1, 7.0 Hz, 1H), 1.04 (s, 9H, br).

^{13}C NMR (151 MHz, $CDCl_3$) δ 155.9, 140.0, 139.2, 136.4 (br), 133.1 (br), 128.5, 128.0 (br), 126.1, 123.6 (br), 123.4, 120.6, 119.7, 117.4 (br), 110.9, 110.8, 53.4, 38.4, 25.7.

HRMS: Calc'd for $C_{27}H_{29}N_2O_3$, $[M+H]^+$ 429.2173; found 429.2180.

tert-butyl (2*R*,5*R*)-2-(*tert*-butyl)-5-(3,4-dimethoxyphenyl)oxazolidine-3-carboxylate (18a)

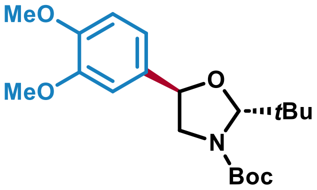

Following **General Procedure A** on 0.2 mmol scale with **11** (*trans*-Boc-RAE). Purification by flash column chromatography (hexane/EA = 15:1) afforded the title compound **18a** (28 mg, 39% yield, *dr* >20:1).

Physical State: white solid.

R_f = 0.38 (hexanes/EtOAc 6:1, UV).

$[\alpha]_D^{25}$ = +45.0 (c 1.0, $CHCl_3$).

^{1}H NMR (600 MHz, $CDCl_3$) δ 6.85 – 6.71 (m, 3H), 5.34 (s, 1H, br), 5.22 – 5.13 (m, 1H), 4.04 – 3.87 (m, 4H), 3.85 (s, 3H), 3.64 – 3.58 (m, 1H), 1.37 (s, 9H, br), 0.98 (s, 9H).

^{13}C NMR (151 MHz, $CDCl_3$) δ 149.3, 148.7, 111.2, 96.4, 56.1, 56.0, 38.2, 28.4, 25.6.

HRMS: Calc'd for $C_{20}H_{31}NO_5Na$, $[M+H]^+$ 388.2095; found 388.2103.

benzyl (2*R*,5*R*)-2-(*tert*-butyl)-5-(3-formyl-4-hydroxyphenyl)oxazolidine-3-carboxylate (18b)

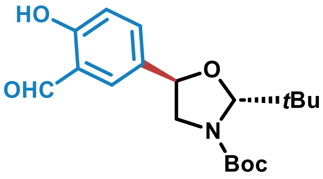

Following **General Procedure A** on 0.2 mmol scale with **11** (*trans*-Boc-RAE). Purification by flash column chromatography (hexane/EA = 15:1) afforded the title compound **18b** (29.3 mg, 41% yield, *dr* =20:1).

Physical State: white solid.

R_f = 0.50 (hexanes/EtOAc 5:1, UV).

$[\alpha]_D^{25}$ = +57.4 (c 0.5, $CHCl_3$).

^{1}H NMR (600 MHz, $CDCl_3$) δ 10.97 (s, 1H), 9.88 (s, 1H), 7.45 (d, *J* = 2.3 Hz, 1H), 7.40 (d, *J* = 8.8 Hz, 1H), 6.97 (d, *J* = 8.6 Hz, 1H), 5.54 – 5.23 (m, 1H, br), 5.21 (dd, *J* = 6.7, 2.2 Hz, 1H), 4.20 – 3.80 (m, 1H, br), 3.62 (dd, *J* = 11.3, 6.8 Hz, 1H), 1.35 (s, 9H, br), 0.99 (s, 9H).

^{13}C NMR (151 MHz, $CDCl_3$) δ 196.6, 161.3, 155.2, 134.4 (br), 130.4, 120.4, 118.2, 96.4, 38.1, 28.3, 25.5.

HRMS: Calc'd for $C_{14}H_{20}NO_3$, $[M\text{-}Boc+2H]^+$ 250.1438; found 250.1442.

tert-butyl (2*R*,5*R*)-5-(4-amino-3,5-dichlorophenyl)-2-(*tert*-butyl)oxazolidine-3-carboxylate (18c)

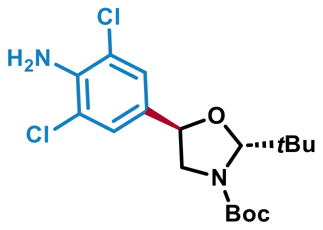

Following **General Procedure B** (slow addition) on 0.1 mmol scale with **11** (*trans*-Boc-RAE). Purification by flash column chromatography (hexane/EA = 15:1) afforded the title compound **18c** (20.2 mg, 52% yield, *dr* >20:1).

Physical State: colorless oil.

R_f = 0.50 (hexanes/EtOAc 10:1, UV).

$[\alpha]_D^{25}$ = +45.2 (c 1.0, $CHCl_3$).

^{1}H NMR (600 MHz, $CDCl_3$) δ 7.05 (s, 2H), 5.29 (s, 1H, br), 5.05 (dd, *J* = 6.8, 2.1 Hz,

1H), 4.47 – 4.35 (m, 2H, -NH_2), 4.01 (s, 1H, br), 3.55 (dd, J = 11.4, 6.7 Hz, 1H), 1.41 (s, 9H, br), 0.96 (s, 9H).

^{13}C NMR (151 MHz, $CDCl_3$) δ 155.2, 139.6, 125.1, 119.8, 96.4, 38.1, 28.3, 25.5.

HRMS: Calc'd for $C_{18}H_{27}Cl_2N_2O_3$, $[M+H]^+$ 389.1394; found 389.1399.

tert-butyl (2*S*,5*S*)-2-(*tert*-butyl)-5-(2,4-dichlorophenyl)oxazolidine-3-carboxylate (18d)

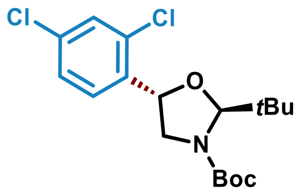

Following **General Procedure A** on 1.0 mmol scale (12 mA) with **11** (*cis*-Boc-RAE). Purification by flash column chromatography (hexane/EA = 20:1) afforded the title compound **18d** (190.3 mg, 51% yield, *dr* >20:1).

Physical State: white solid.

R_f = 0.49 (hexanes/EtOAc 10:1, UV).

$[\alpha]_D^{25}$ = +1.7 (c 1.0, $CHCl_3$).

^{1}H NMR (600 MHz, $CDCl_3$) δ 7.35 (d, J = 2.0 Hz, 1H), 7.29 – 7.23 (m, 1H, br), 7.21 (dd, J = 8.3, 2.1 Hz, 1H), 5.61 – 5.29 (m, 2H, br), 4.00 (s, 1H, br), 3.68 (dd, J = 11.6, 7.1 Hz, 1H), 1.49 – 1.15 (m, 9H, br), 0.99 (s, 9H).

^{13}C NMR (151 MHz, $CDCl_3$) δ 138.7 (br), 133.8, 131.8 (br), 129.3 (br), 127.0 (br), 97.0 (br), 75.6 (br), 38.0, 28.1, 25.4.

HRMS: Calc'd for $C_{13}H_{18}Cl_2NO$, $[M\text{-}Boc+2H]^+$ 274.0760; found 274.0768.

benzyl (4*R*,5*R*)-5-(4-formylphenyl)-2,2-dimethyl-4-phenyloxazolidine-3-carboxylate (19)

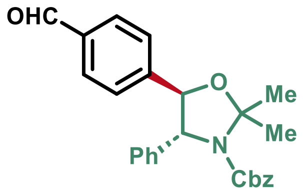

Following **General Procedure A** on 0.2 mmol scale with **RAE S-6**. Purification by flash column chromatography (hexane/EA = 10:1) afforded the title compound **19** (43 mg, 52% yield, *dr* >20:1).

Physical State: colorless oil.

$\boldsymbol{R_f}$ = 0.51 (hexanes/EtOAc 3:1, UV).

[α]$_D^{25}$ = -88.6 (c 1.0, $CHCl_3$).

^{1}H NMR (600 MHz, $CDCl_3$) δ 10.00 (s, 1H), 7.82 (d, *J* = 8.3 Hz, 2H), 7.39 -7.23 (m, 6H, br), 7.22 -7.02 (m, 5H, br), 6.60 (s, 1H, br), 4.94 (d, *J* = 8.7 Hz, 1H), 4.88 (s, 1H, br), 4.77 (s, 1H, br), 4.53 (s, 1H, br), 1.89 (s, 3H, br), 1.87 (s, 3H, br).

^{13}C NMR (151 MHz, $CDCl_3$) δ 192.0, 143.9, 136.6, 130.0, 128.9, 128.1 (br) 127.4, 126.6.

HRMS: Calc'd for $C_{26}H_{25}NO_4$, $[M+H]^+$ 415.1779; not found.

benzyl (4*S*,5*S*)-4-(4-formylphenyl)-2,2,5-trimethyloxazolidine-3-carboxylate (20)

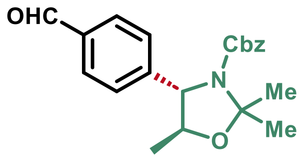

Following **General Procedure A** on 0.2 mmol scale with **RAE S-7**. Purification by flash column chromatography (hexane/EA = 10:1) afforded the title compound **20** (43 mg, 61% yield, *dr* >20:1).

Physical State: colorless oil.

$\boldsymbol{R_f}$ = 0.47 (hexanes/EtOAc 5:1, UV).

[α]$_D$^{25} = -65.8 (c 1.0, $CHCl_3$).

^{1}H NMR (600 MHz, $CDCl_3$) δ 9.99 (s, 1H), 7.94 – 7.70 (m, 2H), 7.51 – 7.27 (m, 4H), 7.23 – 7.02 (m, 2H), 6.72 – 6.61 (m, 1H), 5.18 – 5.02 (m, 1H), 4.91 (d, *J* = 12.4 Hz, 1H), 4.70 (d, *J* = 12.4 Hz, 1H), 4.47 – 4.29 (m, 1H), 3.95 (dq, *J* = 8.5, 6.0 Hz, 1H), 1.81 – 1.65 (m, 6H), 1.29 (d, *J* = 6.0 Hz, 3H).

^{13}C NMR (151 MHz, $CDCl_3$) δ 191.7 (br), 152.3 (br), 146.9 (br), 135.9 (br), 130.2 (br), 128.1 (br), 127.8 (br), 126.8, 95.1 (br), 78.6 (br), 68.6 (br), 66.7 (br), 26.5 (br), 25.3 (br), 16.8 (br).

HRMS: Calc'd for $C_{21}H_{23}NO_4$, $[M+H]^+$ 353.1622; not found

methyl (4*S*,5*R*)-5-(4-formylphenyl)-2,2-dimethyl-1,3-dioxolane-4-carboxylate (21a)

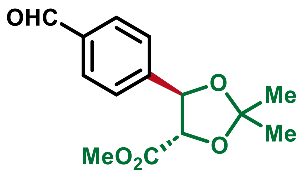

Following **General Procedure A** on 0.2 mmol scale with **RAE S-9** Purification by flash column chromatography (hexane/EA = 10:1) afforded the title compound **21a** (29.6 mg, 56% yield, *dr* >20:1).

Physical State: colorless oil.

R_f = 0.49 (hexanes/EtOAc 3:1, UV).

[α]$_D$^{25} = +18.2 (c 1.0, $CHCl_3$).

^{1}H NMR (600 MHz, $CDCl_3$) δ 10.03 (s, 1H), 7.90 (d, *J* = 8.2 Hz, 2H), 7.62 (d, *J* = 8.2 Hz, 2H), 5.25 (d, *J* = 7.5 Hz, 1H), 4.34 (d, *J* = 7.5 Hz, 1H), 3.82 (s, 3H), 1.63 (s, 3H), 1.57 (s, 3H).

^{13}C NMR (151 MHz, $CDCl_3$) δ 191.9, 170.6, 144.8, 136.6, 130.2, 127.1, 112.3, 81.2, 80.1, 52.8, 26.9, 25.9.

HRMS: Calc'd for $C_{14}H_{16}O_5$, $[M+H]^+$ 264.0093; not found

methyl (4*S*,5*R*)-5-(4-formylphenyl)-2,2-dimethyl-1,3-dioxolane-4-carboxylate (21b)

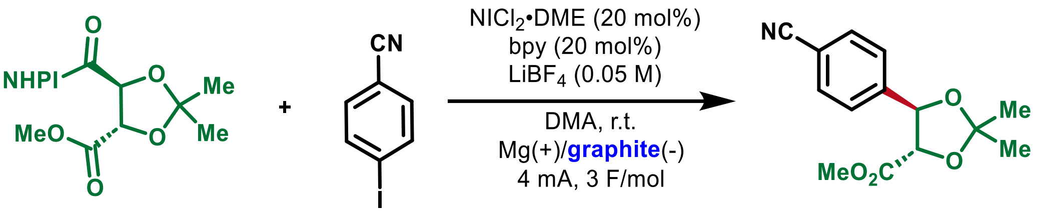

S-9 (1.0 equiv.) 4-iodobenzonitrile (2.0 equiv.) 0.2 mmol in 3.5 mL 21b (62% yield, dr > 20:1)

(Using graphite as cathood with modified electrolysis condition)

An ElectraSyn vial (5 mL) with a magnetic stir bar was charged with **RAE S-9** (0.2 mmol, 1.0 equiv.), **4-iodobenzonitrile** (0.4 mmol, 2.0 equiv.), $NiCl_2$·DME (20 mol%), 2,2'-bipyridine (bpy) (20 mol%) and $LiBF_4$ (0.05 M). The ElectraSyn vial cap equipped with anode (magnesium) and cathode (Graphite) was inserted into the mixture. The vial was then evacuated and backfilled with an argon balloon, and DMA (3.5 mL) were added to the vial via syringe and the resulting solution was stirred for another 1 min. The vial was connected to the ElectraSyn, and the ElectraSyn was set up as follow: New exp. > Constant current > 4 mA > No ref. electrode > Total charge > 0.2 mmol, 3.0 F/mol > No alternating polarity > Start. After electrolysis, the ElectraSyn vial cap was removed and electrodes were rinsed with Et_2O, which was combined with the crude mixture. The combined solvent was concentrated *in vacuo*, purified by flash column chromatography (hexane/EA = 10:1) afforded the title compound **21b** (32.3 mg, 62% yield, *dr* >20:1).

Physical State: colorless oil.

R_f = 0.45 (hexanes/EtOAc 3:1, UV).

$[\alpha]_D^{25}$ = +24.5 (c 1.0, $CHCl_3$).

^{1}H NMR (600 MHz, $CDCl_3$) δ 7.68 (dt, *J* = 8.4, 1.8 Hz, 2H), 7.61 – 7.52 (m, 2H), 5.22 (d, *J* = 7.5 Hz, 1H), 4.30 (d, *J* = 7.4 Hz, 1H), 3.82 (s, 3H), 1.61 (s, 3H), 1.55 (s, 3H).

^{13}C NMR (151 MHz, $CDCl_3$) δ 170.5, 143.5, 132.6, 127.2, 118.7, 112.5, 112.4, 81.1, 79.8, 52.9, 26.9, 25.8.

HRMS: Calc'd for $C_{14}H_{15}NO_4$, $[M+H]^+$ 261.0996; not found

Synthetic Applications

Synthesis of 27

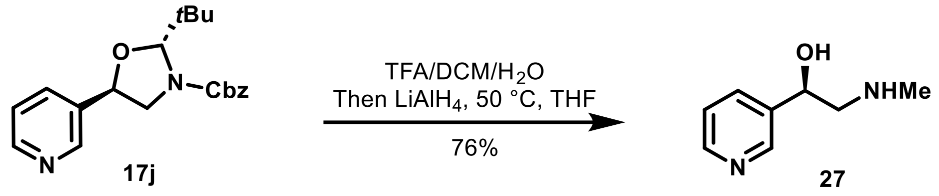

To a solution of **17j** (20 mg, 58.8 μmol, 1.0 equiv.) in CH_2Cl_2 (2.0 mL) was added H_2O (0.2 mL) and trifluoroacetic acid (TFA) (1.0 mL). The reaction was stirred vigorously for 24 h at room temperature. The solvent was removed under reduced pressure to give a colorless oil. THF (4.0 mL) was added to dissolve the oil, and reaction was cooled to 0 ºC prior to the slow addition of $LiAlH_4$ (0.19 mL, 293 μmol, 5.0 equiv., 2 M in the THF). Upon complete addition, the resulting mixture was submerged in a preheated oil bath at 60 °C. The starting material was fully consumed after 4 h, the reaction was removed from the bath and allowed to cool to ambient temperature. The reaction was quenched by slow addition of *sat. aq.* potassium sodium tartrate (5 mL) and diluted with EtOAc (5.0 mL). The mixture was stirred vigorously at room temperature until the two phases separated. The aqueous phase was extracted with EtOAc (3 × 10 mL). The combined organic phases were washed with brine, dried over Na_2SO_4, filtered and concentrated. The crude product was purified by pTLC (CH_2Cl_2/MeOH/$NH_3·H_2O$ = 4:1:0.05) to give the desired benzylic alcohol product **27**[4] (6.8 mg, 76%) as a colorless oil.

Physical State: colorless oil.

$\boldsymbol{R_f}$ = 0.35 (CH_2Cl_2/MeOH/$NH_3·H_2O$ = 4:1:0.05, UV).

$[\alpha]_D^{25}$ = -78.0 (c 0.2, $CHCl_3$).

^{1}H NMR (600 MHz, $CDCl_3$) δ 8.60 (d, *J* = 2.2 Hz, 1H), 8.52 (dd, *J* = 4.8, 1.7 Hz, 1H), 7.73 (dt, *J* = 7.8, 2.2 Hz, 1H), 7.28 (dd, *J* = 7.8, 4.8 Hz, 1H), 4.75 (dd, *J* = 9.3, 3.6 Hz, 1H), 2.86 (dd, *J* = 12.3, 3.6 Hz, 1H), 2.70 (dd, *J* = 12.2, 9.3 Hz, 1H), 2.49 (s, 3H).

^{13}C NMR (151 MHz, $CDCl_3$) δ 149.1, 148.0, 138.0, 133.7, 123.6, 69.3, 59.0, 36.1.

HRMS: Calc'd for $C_8H_{13}N_2O$, $[M+H]^+$ 153.1023; found 153.1028.

Synthesis of 28

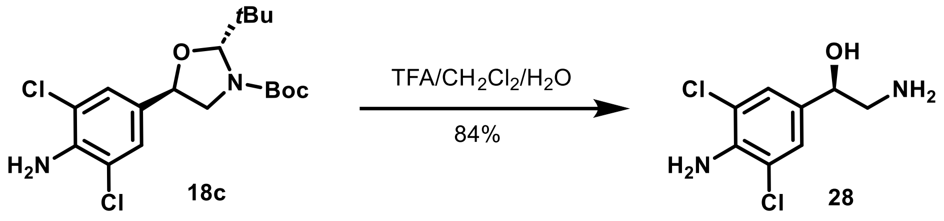

To a solution of **18c** (20 mg, 58.8 μmol, 1.0 equiv.) in CH_2Cl_2 (2 mL) was added H_2O (0.2 mL) and trifluoroacetic acid (TFA) (1 mL). The reaction was vigorously stirred for 24 h at room temperature. The solvent was removed under reduced pressure. The crude mixture was diluted with EtOAc/MeOH (50:1) (10 mL) and washed with NaOH (1 M). The layers were separated, and the aqueous layer was extracted with EtOAc/MeOH (50:1) (10 mL × 2). The combined organic layers were washed with water (2 mL) and brine (5 mL), dried over anhydrous Na_2SO_4, and concentrated *in vacuo*. The crude residue was purified by pTLC (CH_2Cl_2/MeOH/$NH_3\cdot H_2O$ = 4:1:0.05) to give **28**[5] (9.5 mg, 84%) as a colorless oil.

Physical State: colorless oil.

R_f = 0.41 (CH_2Cl_2/MeOH/$NH_3\cdot H_2O$ = 4:1:0.05 4:1, UV).

$[\alpha]_D^{25}$ = -2.3 (c 0.4, $CHCl_3$).

^{1}H NMR (600 MHz, $CDCl_3$) δ 7.19 (s, 2H), 4.47 (dd, J = 7.8, 4.0 Hz, 1H), 4.41 (s, 2H, -NH_2), 2.97 (dd, J = 12.8, 4.0 Hz, 1H), 2.73 (dd, J = 12.7, 7.8 Hz, 1H).

^{13}C NMR (151 MHz, $CDCl_3$) δ 139.5, 133.0, 125.6, 119.7, 73.0, 49.1.

HRMS: Calc'd for $C_8H_9Cl_2N_2$, $[M-OH]^+$ 203.0138; found 203.0141.

Synthesis of S-11

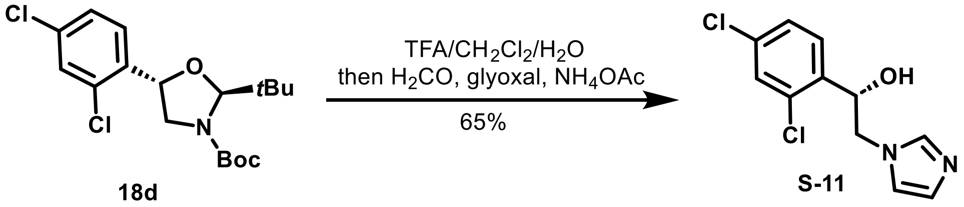

To a solution of **18d** (200 mg, 0.54 mmol, 1.0 equiv.) in CH_2Cl_2 (4.0 mL) was added H_2O (0.4 mL) and trifluoroacetic acid (TFA) (2.0 mL). The reaction was stirred vigorously for 24 h at room temperature. The solvent was removed under reduced pressure to give a colorless oil. Methanol (2.0 mL) was added to dissolve the oil, then 40% aqueous glyoxal (122 µL, 1.1 mmol, 2.0 equiv.), 37% aqueous formaldehyde (83 µL, 1.1 mmol, 2.0 equiv.), and NH_4OAc (83.5 mg, 1.1 mmol, 2.0 equiv.) was added. The reaction was stirred overnight at 80 °C under Ar. After cooling to rt, MeOH was removed *in vacuo*, and the residue was treated with 2 M NaOH (0.5 mL) and extracted with CH_2Cl_2 (10 mL). The layers were separated and the aqueous layer was extracted with CH_2Cl_2 (2 × 10 mL). The combined organic layers were washed with brine (5 mL), dried over Na_2SO_4, filtered and concentrated *in vacu*o. The crude residue was purified by SiO_2 flash chromatography (eluent: EtOAc/hexanes = 1/1) to give alcohol **S-11** (92.5 mg, 65%) as a colorless oil.

Physical State: colorless oil.

$\boldsymbol{R_f}$ = 0.21 (hexanes/EtOAc 1:2, UV).

$[\alpha]_D^{25}$ = +62.8 (c 1.0, $CHCl_3$).

^{1}H NMR (600 MHz, $CDCl_3$) δ 7.57 (dd, *J* = 8.4, 0.6 Hz, 1H), 7.42 (t, *J* = 1.2 Hz, 1H), 7.40 (d, *J* = 2.1 Hz, 1H), 7.31 (dd, *J* = 8.4, 2.1 Hz, 1H), 6.91 (t, *J* = 1.2 Hz, 1H), 6.89 (t, *J* = 1.2 Hz, 1H), 5.25 (dd, *J* = 8.3, 2.4 Hz, 1H), 4.22 (dd, *J* = 14.3, 2.3 Hz, 1H), 3.88 (dd, *J* = 14.2, 8.3 Hz, 1H).

^{13}C NMR (151 MHz, $CDCl_3$) 137.8, 137.2, 134.5, 132.1, 129.3, 128.9, 128.7, 127.9, 119.6, 70.0, 53.4.

HRMS: Calc'd for $C_{11}H_{11}Cl_2N_2O$, $[M+H]^+$ 257.0243; found 257.0247.

Synthesis of (S)-econazole (1)

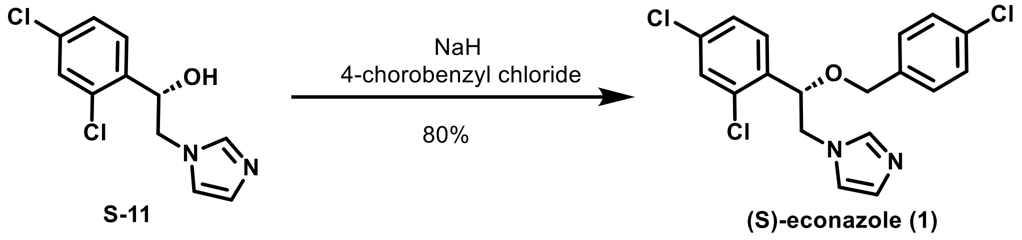

To a solution of **S-11**(50.1 mg, 195 μmol, 1.0 equiv) in THF/DMF (2.2 mL, 10:1 v/v), NaH (11.7 mg, 195 μmol, 1.5 equiv., 60% in oil) was added in small portions at 0 °C. After 5 min, 4-chlorobenzyl chloride (31.4 mg, 195 μmol, 1.0 equiv) was added dropwise, followed by a one-time addition of tetra-*n*-butylammonium iodide (TBAI) (7.0 mg, 19.5 μmol, 10 mol%). The resulting reaction mixture was left stirring at rt overnight under Ar. The reaction was diluted with water (2 mL) and ether (10 mL), and the layers were separated. The aqueous layer was extracted with ether (5 mL × 2) and combined organic layers were washed with water (2 mL) and brine (2 mL), dried over anhydrous Na_2SO_4, concentrated, and purified by silica gel column chromatography (hexanes/EtOAc = 2:1) to give the product **(S)-econazole (1)**[6] (59.1 mg, 80%) as a colorless oil.

Physical State: colorless oil.

$\boldsymbol{R_f}$ = 0.32 (hexanes/EtOAc 1:1, UV).

$[\alpha]_D^{25}$ = +82.6 (c 1.0, $CHCl_3$).

^{1}H NMR (600 MHz, $CDCl_3$) δ 7.46 (s, 1H), 7.44 (d, *J* = 2.0 Hz, 1H), 7.33 (d, *J* = 8.3 Hz, 1H), 7.30 – 7.27 (m, 3H), 7.06 (d, *J* = 8.4 Hz, 2H), 7.03 (s, 1H), 6.89 (t, *J* = 1.3 Hz, 1H), 4.95 (dd, *J* = 7.7, 2.8 Hz, 1H), 4.43 (d, *J* = 11.8 Hz, 1H), 4.22 – 4.16 (m, 2H), 4.03 (dd, *J* = 14.6, 7.7 Hz, 1H).

^{13}C NMR (151 MHz, $CDCl_3$) δ 138.0, 135.5, 135.1, 134.1, 134.0, 133.5, 129.8, 129.4, 129.2, 128.9, 128.6, 128.1, 119.9, 77.0, 70.9, 51.5.

HRMS: Calc'd for $C_{18}H_{16}Cl_3N_2O$, $[M+H]^+$ 381.0323; found 381.0323.

Synthesis of 30

Ag condition

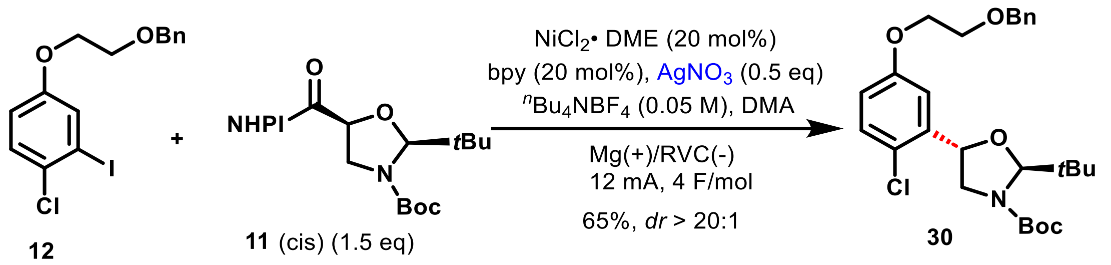

Following **General Procedure A** on 1.0 mmol scale (12 mA). Purification by flash column chromatography (hexane/EA = 20:1) afforded the title compound **30** (317 mg, 65% yield, *dr* > 20:1).

Ag-free condition

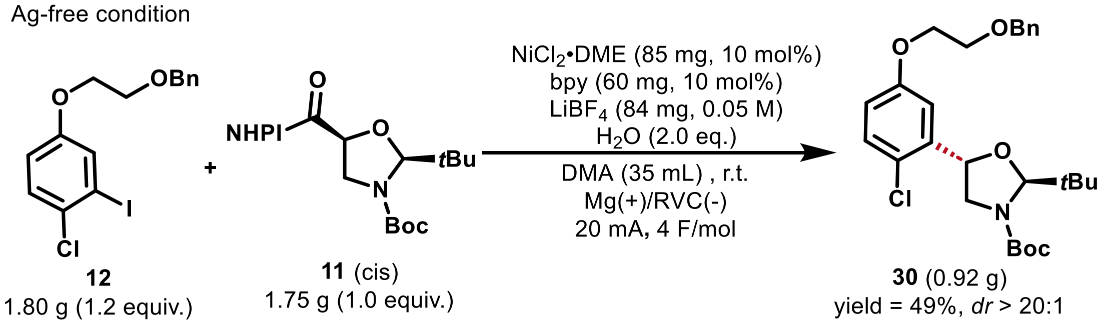

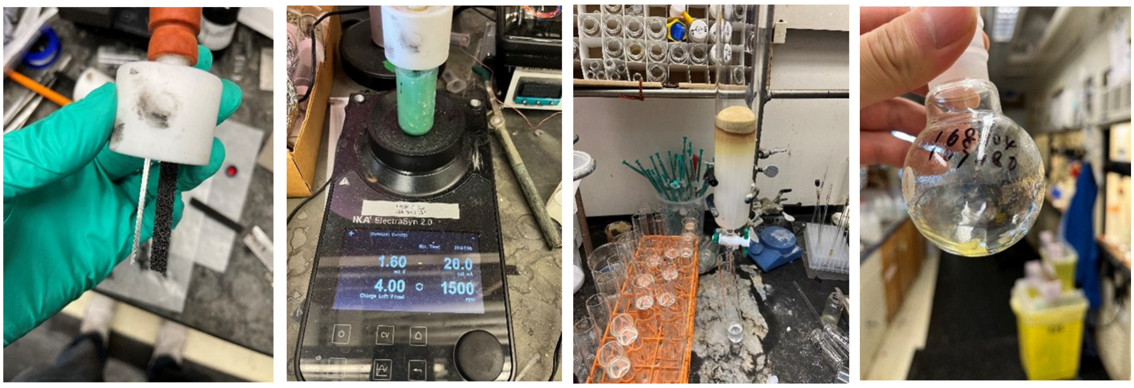

Anode (magnesium) and cathode (RVC) | Start the reaction with ElectraSyn 2.0 | Purification with flash column chromatography | Desired product **30** (0.92 g, 49% yield).

Permission is granted by IKA.

Physical State: colorless oil.

R_f = 0.5 (hexanes/EtOAc 5:1, UV).

$[\alpha]_D^{25}$ = -36.0 (c 1.0, $CHCl_3$).

^{1}H NMR (600 MHz, $CDCl_3$) δ 7.39 – 7.27 (m, 5H), 7.21 (d, *J* = 8.7 Hz, 1H), 6.91 (d,

J = 3.0 Hz, 1H), 6.75 (d, *J* = 8.7 Hz, 1H, br), 5.55 (s, 1H, br), 5.43 (d, *J* = 7.0 Hz, 1H, br), 4.62 (s, 2H), 4.11 (td, *J* = 4.6, 2.4 Hz, 2H), 4.00 (s, 1H, br), 3.81 (t, *J* = 4.9 Hz, 2H), 3.69 (dd, *J* = 11.5, 7.1 Hz, 1H), 1.12-1.41 (m, 9H, br), 1.00 (s, 9H).

^{13}C NMR (151 MHz, $CDCl_3$) δ 157.9 (br), 155.2 (br), 141.2 (br), 138.1, 130.2 (br), 128.6, 127.9, 127.9, 122.7, 113.1, 97.1, 80.6 (br), 76.1 (br), 73.5, 68.5, 67.9, 38.1, 28.2, 25.5.

HRMS: Calc'd for $C_{27}H_{36}ClNO_5Na$, $[M+Na]^+$ 512.2175; found 512.2186.

Synthesis of 31

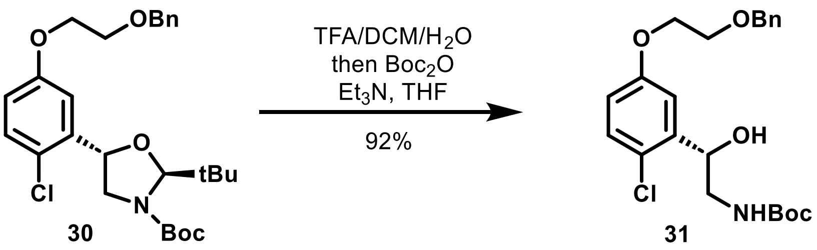

To a solution of **30** (200 mg, 0.41 mmol, 1.0 equiv.) in CH_2Cl_2 (4.0 mL) was added H_2O (0.4 mL) and trifluoroacetic acid (TFA) (2.0 mL). The reaction was stirred vigorously for 24 h at room temperature. The solvent was removed under reduced pressure to give a light red oil. THF (4 mL) was added to dissolve the oil, then triethylamine (0.17 mL, 1.23 mmol, 3.0 equiv.) and di-*tert*-butyl decarbonate (Boc_2O) (0.19 mL, 0.82 mmol, 2.0 equiv.) was added in a single portion. The reaction was stirred at ambient temperature for 12 hours at which point the TLC analysis indicated complete consumption of the starting material. The mixture was concentrated and was directly purified by silica gel column chromatography (eluent: hexanes/EtOAc = 5/1) to give **31** (158 mg, 92%) as a colorless oil.

Physical State: colorless oil.

$\boldsymbol{R_f}$ = 0.50 (hexanes/EtOAc 5:1, UV).

$[\alpha]_D^{25}$ = +26.1 (c 1.0, $CHCl_3$).

^{1}H NMR (600 MHz, $CDCl_3$) δ 7.39 – 7.27 (m, 5H), 7.23 – 7.16 (m, 2H), 6.79 (dd, *J* =

8.8, 3.1 Hz, 1H), 5.21 – 5.07 (m, 1H), 4.94 (s, 1H), 4.62 (s, 2H), 4.14 (td, J = 4.6, 3.0 Hz, 2H), 3.82 (t, J = 4.8 Hz, 2H), 3.54 -3.47 (m, 1H), 3.32 (t, J = 7.9 Hz, 1H, -NH-), 1.44 (s, 9H).

^{13}C NMR (151 MHz, CDCl$_3$) δ 159.1, 158.0, 140.3, 138.1, 130.1, 128.6, 127.9, 127.9, 123.1, 115.6, 113.6, 80.4, 73.5, 71.8, 68.5, 67.9, 46.7, 28.5.

HRMS: Calc'd for $C_{17}H_{21}ClNO_3$, [M-Boc+2H]$^+$ 322.1205; found 322.1210.

Synthesis of S-12

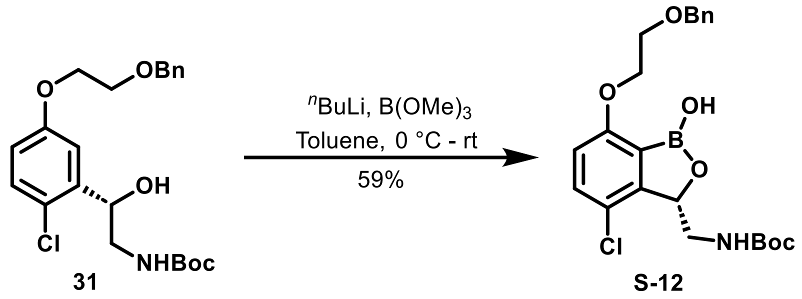

To a solution of **31** (80 mg, 0.19 mmol, 1.0 equiv.) in toluene (4.0 mL) at 0 °C was added *n*BuLi (0.38 mL, 2.5 M in hexane, 0.95 mmol, 5.0 equiv.) under Ar atmosphere. Upon complete addition, the reaction was stirred for 1 hour at 0 °C. Trimethyl borate (0.1 mL, 0.95 mmol, 5.0 equiv.) was added dropwise *via* a syringe. The resulting solution was stirred at 0 °C for 45 min and allowed to warm up to ambient temperature. After 2 h, the reaction was quenched with *sat. aq.* $NaHCO_3$ (1.0 mL) and the layers were separated. The aqueous layer was extracted with EtOAc (2×10 mL). The combined layers were washed with brine (5 mL), dried over Na_2SO_4, filtered and concentrated *in vacuo*. The crude residue was purified by SiO_2 flash chromatography (eluent: EtOAc/hexanes = 1/4) to give **S-12** (50.1 mg, 60% yield) as a colorless oil.

Physical State: colorless oil.

R_f = 0.31 (hexanes/EtOAc 1:1, UV).

$[\alpha]_D^{25}$ = +47.7 (c 1.0, $CHCl_3$).

^{1}H NMR (600 MHz, CDCl$_3$) δ 7.38 – 7.32 (m, 6H), 6.82 (d, J = 8.5 Hz, 1H), 6.38 (s,

1H, B-OH), 5.28 (d, $J = 7.9$ Hz, 1H, br), 4.93 (s, 1H, br), 4.66 (s, 2H), 4.21 – 4.15 (m, 2H), 3.81 (t, $J = 4.5$ Hz, 2H), 3.10 (m, 1H, br), 1.40 (s, 9H).

^{13}C NMR (151 MHz, CDCl$_3$) δ 160.3, 155.9, 152.2, 137.3, 133.9, 128.8, 128.3, 128.2, 121.9, 115.6, 80.4, 73.6, 70.3, 68.4, 43.8, 28.5.

HRMS: Calc'd for $C_{22}H_{27}BClNO_6Na$, $[M+Na]^+$ 469.1549; found 469.1555.

Synthesis of S-12

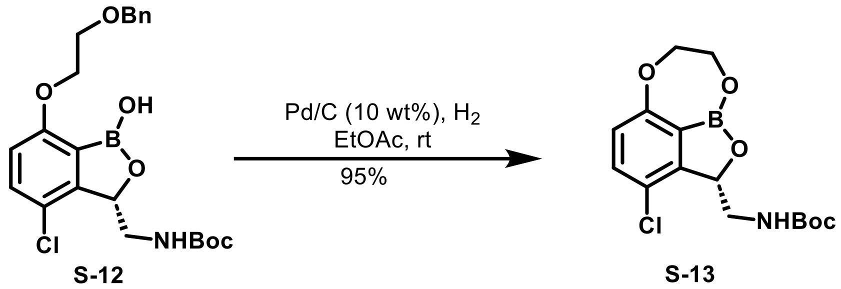

To a solution of **S-12** (50 mg, 0.11 mmol, 1.0 equiv.) in EtOAc (4.0 mL) was added Pd/C (10% on the carbon, dry) (10 mg, 10 wt%). The reaction was vigorously stirred under an atmosphere of H_2 *via* a double-walled balloon. After 2 h, the starting material was fully consumed. The reaction solution was then filtered, concentrated *in vacuo*. The crude residue was purified by SiO_2 flash chromatography (eluent: EtOAc/hexanes = 1/5) to give **S-13** (36.0 mg, 95% yield) as a colorless oil.

Physical State: colorless oil.

R_f = 0.52 (hexanes/EtOAc 1:1, UV).

$[\alpha]_D^{25}$ = +38.4 (c 1.0, CHCl$_3$).

^{1}H NMR (600 MHz, CDCl$_3$) δ 7.33 (d, $J = 8.5$ Hz, 1H), 6.84 (d, $J = 8.5$ Hz, 1H), 5.38 (s, 1H, br), 4.91 (s, 1H, -NH-), 4.65 (m, 1H, br), 4.50 – 4.05 (m, 4H, br), 3.15 (m, 1H, br), 1.40 (s, 9H).

^{13}C NMR (151 MHz, CDCl$_3$) δ 160.8, 155.7, 150.5, 134.4, 120.7, 117.6, 117.3, 82.4, 79.4, 73.8, 69.2, 43.5, 28.3.

HRMS: Calc'd for $C_{10}H_{12}BClNO_3$, $[M-Boc+2H]^+$ 239.0630; found 239.0636.

Synthesis of GSK-656 (4)

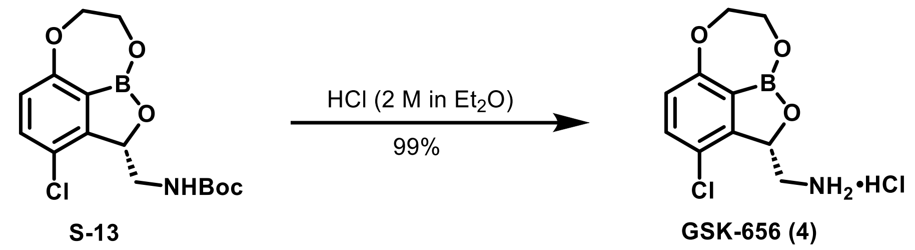

S-13 (20 mg, 59.0 μmol, 1.0 equiv.) was dissolved in 2 M HCl in Et_2O (2.0 mL), and the reaction was stirred at rt for 2 h. The solvent was removed under vacuum, and the residue was dissolved in water and filtered through a membrane filter. The *aq.* solution was concentrated *in vacuo* to give **GSK-656** (**4**)[7] (14 mg, 99%).

Physical State: colorless oil.

$\boldsymbol{R_f}$ = 0.2 (CH_2Cl_2/MeOH/$NH_3 \cdot H_2O$ = 4:1:0.05 4:1, UV).

$[\alpha]_D^{25}$ = +36.0 (c 0.5, MeOH).

^{1}H NMR (600 MHz, DMSO-*d6*) δ 8.43 (s, 3H), 7.50 (d, *J* = 8.6 Hz, 1H), 6.97 (d, *J* = 8.5 Hz, 1H), 5.60 (s, 1H, br), 4.70 (s, 1H, br), 4.35 (m, 3H, br), 3.72 – 3.69 (m, 1H), 3.00 (s, 1H, br).

^{13}C NMR (151 MHz, DMSO-*d6*) δ 160.8, 148.7, 134.2, 119.5, 118.9, 117.9, 78.7, 73.4, 68.8, 41.4.

HRMS: Calc'd for $C_{10}H_{11}BClNO_3$, $[M+H]^+$ 239.0630; found 239.0636.

Synthesis of 36

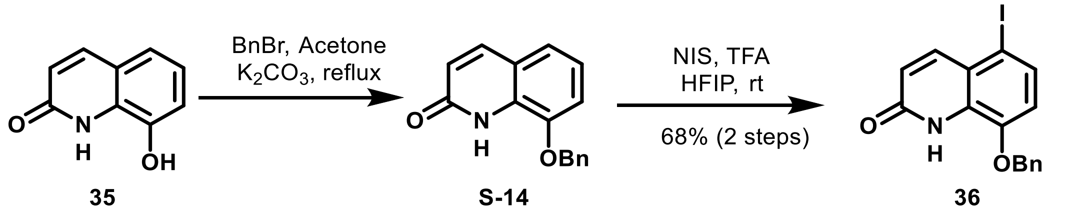

To a solution of **8-hydroxyquinolin-2(1H)-one** (**35**) (3.0 g, 18.3 mmol, 1.0 equiv.) in acetone (36 mL) were added K_2CO_3 (2.8 g, 20.1 mmol, 1.1 equiv.) and benzyl bromide (2.4 mL, 20.1 mmol, 1.1 equiv.) at rt. The mixture was heated to reflux and stirred

overnight. After completion, the solvent was removed under reduced pressure and quenched with 1 N HCl and extracted with ethyl acetate (50 mL × 2) dried over anhydrous Na_2SO_4, concentrated, and purified by silica gel column chromatography (hexanes/EtOAc = 2:1) to give the **S-14** (4.2 g, 91%) as a white solid.[8]

To a solution of **S-14** (2.0 g, 7.97 mmol, 1.0 equiv.) and trifluoroacetic acid (TFA) (0.12 mL, 1.59 mmol, 1.0 equiv.) in hexafluoro-2-propanol (HFIP) (25 mL) was added *N*-iodosuccinimide (2.67 g, 11.9 mmol, 1.5 equiv.). The reaction was stirred for 12 h at which point the TLC analysis indicated complete consumption of the starting material. The solvent was removed under vacuum, and the crude mixture was diluted with EtOAc (50 mL) and *sat. aq.* $NaHCO_3$ (20 mL). The layers were separated, and the aqueous layer was extracted with EtOAc (20 mL). The combined organic layers were washed with water (50 mL × 2) and brine (50 mL), dried over anhydrous Na_2SO_4, concentrated, and purified by silica gel column chromatography (hexanes/EtOAc = 3:1) to give the **36** (2.2 g, 75%) as a colorless oil.

Physical State: white solid.

R_f = 0.46 (hexanes/EtOAc 1:1, UV).

^{1}H NMR (600 MHz, $CDCl_3$) δ 9.21 (s, 1H, -NH-), 7.91 (d, *J* = 9.8 Hz, 1H), 7.59 (d, *J* = 8.5 Hz, 1H), 7.49 – 7.33 (m, 5H), 6.78 (d, *J* = 8.4 Hz, 1H), 6.68 (d, *J* = 9.8 Hz, 1H), 5.17 (s, 2H).

^{13}C NMR (151 MHz, $CDCl_3$) δ 161.7, 145.2, 144.3, 135.2, 132.7, 129.5, 129.1, 129.0, 128.1, 124.1, 112.9, 87.0, 71.4.

HRMS: Calc'd for $C_{17}H_{12}INO_2$, $[M+H]^+$ 377.9986; found 377.9986.

Synthesis of 37

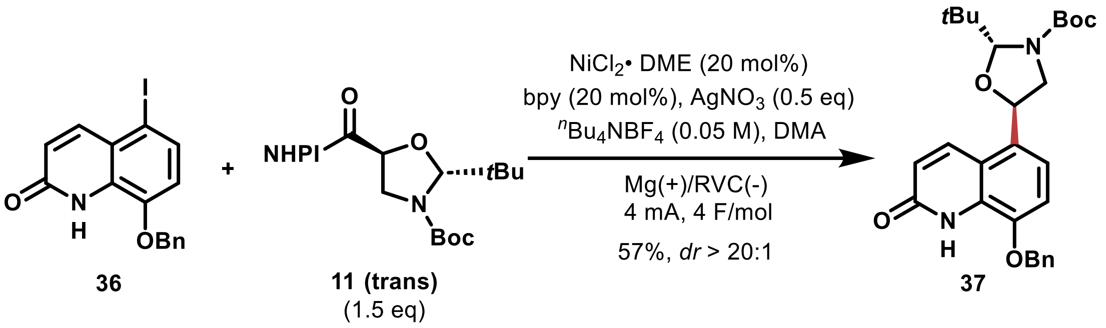

Following General Procedure A on 0.2 mmol scale. Purification by flash column chromatography (hexane/EA = 2:1) afforded the title compound **37** (57% yield, 55 mg, *dr* > 20:1).

Physical State: colorless oil.

R_f = 0.39 (hexanes/EtOAc 1:1, UV).

$[\alpha]_D^{25}$ = +22.2 (c 1.0, $CHCl_3$).

^{1}H NMR (600 MHz, $CDCl_3$) δ 9.24 (s, 1H, br, -NH-), 7.86 (s, 1H, br), 7.50 – 7.32 (m, 5H), 7.06 (d, *J* = 8.4 Hz, 1H), 6.94 (d, *J* = 8.3 Hz, 1H), 6.71 (dd, *J* = 9.8, 1.8 Hz, 1H), 5.62 (dd, *J* = 7.0, 2.4 Hz, 1H), 5.52 – 5.20 (s, 1H, br), 5.22 – 5.11 (m, 2H), 4.30 – 4.19 (m, 1H, br), 3.71 (dd, *J* = 11.3, 7.0 Hz, 1H), 1.42 – 1.25 (m, 9H, br), 1.00 (s, 9H).

^{13}C NMR (151 MHz, $CDCl_3$) δ 161.4, 155.2, 144.4, 136.3 (br), 135.5, 130.5 (br), 129.7 (br), 129.0, 128.8, 128.0, 122.9, 119.0 (br), 110.7 (br), 96.6, 81.0 (br), 75.6 (br), 71.2, 38.2, 28.3, 25.6.

HRMS: Calc'd for $C_{28}H_{35}N_2O_5$, $[M+H]^+$ 479.2541; found 479.2549.

Synthesis of 37

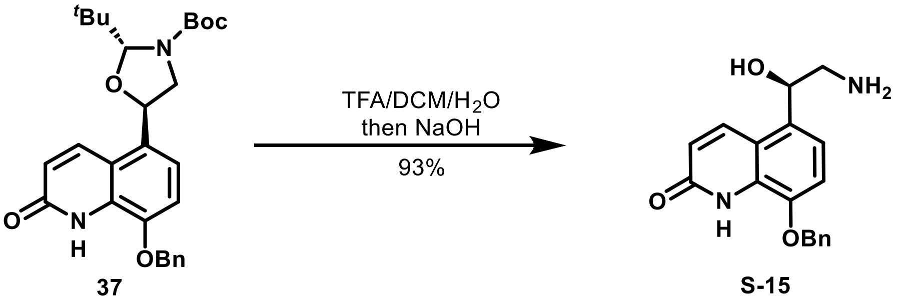

To a solution of **37** (20 mg, 58.8 μmol, 1.0 equiv.) in CH_2Cl_2 (2 mL) was added H_2O

(0.2 mL) and trifluoroacetic acid (TFA) (1 mL), the reaction was stirred vigorously for 24 h at room temperature. The solvent was removed under reduced pressure. The crude mixture was diluted with EtOAc/MeOH (50:1) (10 mL) and washed with NaOH (1 M). The layers were separated and the aqueous layer was extracted with EtOAc/MeOH (50:1) (10 mL × 2) and combined organic layers were washed with water (2 mL) and brine (5 mL), dried over anhydrous Na_2SO_4, and concentrated *in vacuo*, the crude residue was purified *via* pTLC ($CH_2Cl_2/MeOH/NH_3 \cdot H_2O$ = 4:1:0.05) to give **S-15** (12.0 mg, 93%) as a colorless oil.

Physical State: colorless oil.

$\boldsymbol{R_f}$ = 0.50 (hexanes/EtOAc 5:1, UV).

$\boldsymbol{[\alpha]_D^{25}}$ = -25.7 (c 0.5, $CHCl_3$).

^{1}H NMR (600 MHz, $CDCl_3$) 8.10 (d, *J* = 9.9 Hz, 1H), 7.46 – 7.34 (m, 5H), 7.23 (d, *J* = 8.3 Hz, 1H), 7.02 (d, *J* = 8.3 Hz, 1H), 6.66 (d, *J* = 9.9 Hz, 1H), 5.17 (s, 2H), 5.03 (dd, *J* = 8.3, 3.8 Hz, 1H), 3.07 (dd, *J* = 12.8, 3.8 Hz, 1H), 2.86 (dd, *J* = 12.8, 8.3 Hz, 1H).

^{13}C NMR (151 MHz, $CDCl_3$) δ 161.5, 144.1, 136.7, 135.6, 131.3, 129.3, 129.0, 128.8, 128.1, 122.4, 120.0, 117.5, 111.1, 71.3, 71.2, 48.6.

HRMS: Calc'd for $C_{18}H_{19}N_2O_3$, $[M+H]^+$ 311.1391; found 311.1396.

Synthesis of S-16

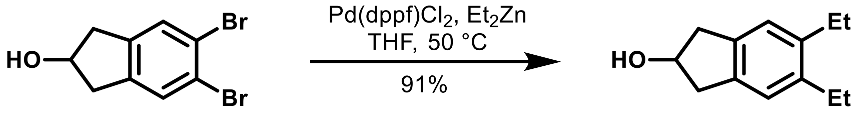

5,6-dibromo-2,3-dihydro-1*H*-inden-2-ol

S-16

A flame-dried, 25 mL flask was charged with 5,6-dibromo-indan-2-ol (220 mg, 0.76 mol, 1.0 equiv.) and $Pd(dppf)Cl_2$ (111 mg, 0.15 mol, 0.2 equiv.). The flask was evacuated and backfilled with argon for three times. Anhydrous THF (5.4 mL) was added *via* a syringe. The resulting mixture was stirred at ambient temperature for 10 min, then Et_2Zn (3.1 mL, 15 wt% in toluene, 0.15 mol, 5.0 equiv.) was added dropwise. After injection, the reaction solution was submerged in a preheated oil bath at 50 °C.

After 20 h, TLC indicated complete consumption of starting material. The reaction was quenched with *sat. aq.* NH_4Cl (5.0 mL) and diluted with EtOAc (20 mL). The layers were separated. The aqueous layer was extracted with EtOAc (10 mL) and the combined organic layers were washed with HCl (1 N) (5 ml), water (10 mL × 2) and brine (10 mL), dried over anhydrous Na_2SO_4, concentrated, and purified by silica gel column chromatography (hexanes/EtOAc = 3:1) to give the desired product (131 mg, 91%) as a colorless oil.

Physical State: colorless oil.

R_f = 0.47 (hexanes/EtOAc 2:1, UV).

^{1}H NMR (600 MHz, $CDCl_3$) δ 7.06 (s, 2H), 4.68 (td, *J* = 6.3, 3.3 Hz, 1H), 3.18 (dd, *J* = 16.2, 5.9 Hz, 2H), 2.87 (dd, *J* = 16.2, 3.3 Hz, 2H), 2.63 (q, *J* = 7.6 Hz, 4H), 1.22 (t, *J* = 7.6 Hz, 6H).

^{13}C NMR (151 MHz, $CDCl_3$) δ 140.4, 138.5, 125.0, 73.7, 42.6, 25.6, 15.7.

EI: Calc'd for $C_{13}H_{18}O$, $[M]^+$ 190; found 190, 161, 128, 115.

Synthesis of 38

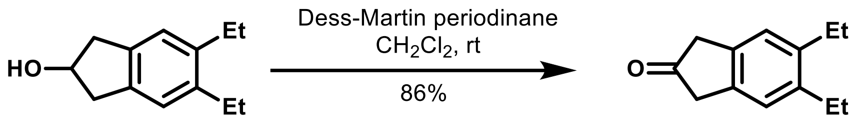

S-16 38

To a solution of **S-16** (130 mg, 0.68 mol, 1.0 equiv.) in CH_2Cl_2 (4.0 mL) was added Dess-Martin periodinane (435 mg, 1.03 mol, 1.5 equiv.). The reaction was stirred for 4 h at ambient temperature. The reaction was quenched with *sat. aq.* NH_4Cl (5.0 mL), and the layers were separated. The aqueous layer was extracted with CH_2Cl_2 (5 mL) and the combined organic layers were washed with water (10 mL × 2) and brine (10 mL), dried over anhydrous Na_2SO_4, concentrated, and purified by silica gel column chromatography (hexanes/EtOAc = 3:1) to give the **38** (111 mg, 86%) as a light-yellow waxy solid

Physical State: light-yellow waxy solid.

R_f = 0.59 (hexanes/EtOAc 8:1, UV).

^{1}H NMR (600 MHz, CDCl$_3$) δ 7.12 (s, 2H), 3.52 (s, 4H), 2.66 (q, *J* = 7.6 Hz, 4H), 1.23 (t, *J* = 7.6 Hz, 6H).

^{13}C NMR (151 MHz, CDCl$_3$) δ 216.2, 141.3, 135.5, 124.9, 44.1, 25.6, 15.6.

EI: Calc'd for $C_{13}H_{16}O$, $[M]^+$ 188; found 188, 160, 145, 128, 115, 91.

Synthesis of indacaterol (2)

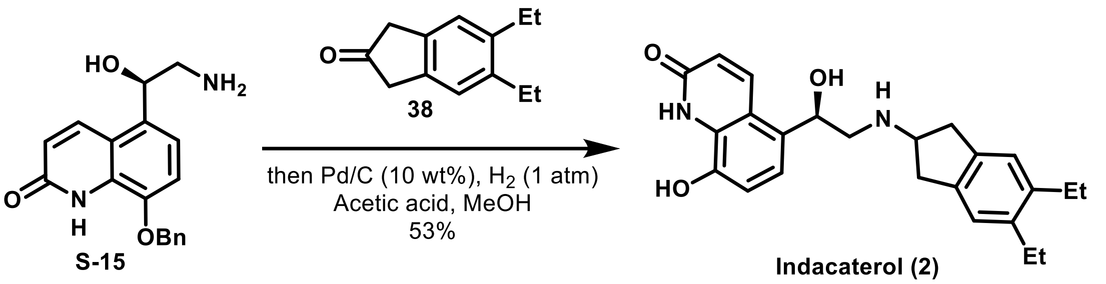

To a solution of **S-15** (9.0 mg, 29.0 μmol, 1.0 equiv) and **38** (10.9 mg, 58.1 μmol, 2,0 equiv) in MeOH (1.0 ml) was added acetic acid (1 drops) and Pd/C (10% on the carbon, dry) (50 wt%, 5 mg). The reaction was vigorously stirred under an atmosphere of H_2 *via* a double-walled balloon. After 8 h, LCMS analysis indicted full consumption of the starting material. The reaction solution was filtered, and the filtrate was concentrated *in vacu*o, and the crude mixture was purified by pTLC (CH_2Cl_2/MeOH/$NH_3 \cdot H_2O$ = 4:1:0.05) to give **indacaterol (2)**[9] (6.1 mg, 53%) as a brown- yellow oil.

Physical State: brown- yellow oil.

R_f = 0.45 (CH_2Cl_2/MeOH/$NH_3 \cdot H_2O$ = 4:1:0.05 4:1, UV).

$[\alpha]_D^{25}$ = -30.4 (c 1.0, MeOH).

^{1}H NMR (600 MHz, DMSO-*d6*) δ 8.18 (d, *J* = 9.8 Hz, 1H), 7.02 (d, *J* = 8.1 Hz, 1H), 6.94 (d, *J* = 5.3 Hz, 2H), 6.83 (d, *J* = 8.0 Hz, 1H), 6.47 (d, *J* = 9.8 Hz, 1H), 4.98 (dd, *J* = 8.3, 4.2 Hz, 1H), 3.50 (d, *J* = 6.8 Hz, 1H), 3.02 – 2.93 (m, 2H), 2.80 – 2.69 (m, 2H), 2.63 – 2.55 (m, 2H), 2.53 (q, *J* = 7.7 Hz, 4H), 1.12 (td, *J* = 7.5, 0.9 Hz, 6H).

^{13}C NMR (151 MHz, DMSO-*d6*) δ 161.2, 142.4, 139.8, 139.8, 139.4, 137.7, 130.1, 129.6, 124.7, 124.7, 121.4, 120.5, 117.4, 114.4, 69.8, 59.7, 56.2, 39.9, 25.3, 16.1.

HRMS: Calc'd for $C_{24}H_{29}N_2O_3$, $[M+H]^+$ 393.2173; found 393.2178.

Synthesis of 44

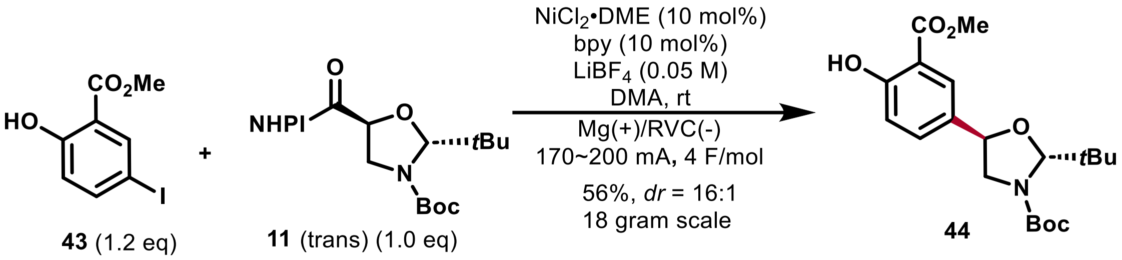

Following **Graphical Guide for a Large-Scale Synthesis of 44**. Purification by flash column chromatography (hexane/EA = 20:1) afforded the title compound **44** (9.1 g, 56% yield, *dr* = 16:1).

Physical State: white solid

Rf = 0.57 (hexanes/EtOAc 5:1, UV).

$[\alpha]_D^{25}$ = +44.9 (c 1.0, $CHCl_3$).

^{1}H NMR (600 MHz, $CDCl_3$) δ 10.71 (s, 1H, -OH), 7.70 (d, *J* = 2.4 Hz, 1H), 7.33 (dd, *J* = 8.6, 2.4 Hz, 1H), 6.95 (d, *J* = 8.6 Hz, 1H), 5.32 (s, 1H, br), 5.16 (dd, *J* = 6.9, 2.4 Hz, 1H), 4.14 - 3.87 (m, 1H, br), 3.94 (s, 3H), 3.60 (dd, *J* = 11.3, 6.8 Hz, 1H), 1.37 (s, 9H), 0.97 (s, 9H).

^{13}C NMR (151 MHz, $CDCl_3$) δ 170.5, 161.3, 155.2, 133.2, 133.1, 126.8, 118.0, 112.2, 96.4, 52.5, 38.1, 28.3, 25.6.

HRMS: Calc'd for $C_{15}H_{22}NO_4$, $[M\text{-}Boc+2H]^+$ 280.1544; found 280.1550.

Synthesis of 45

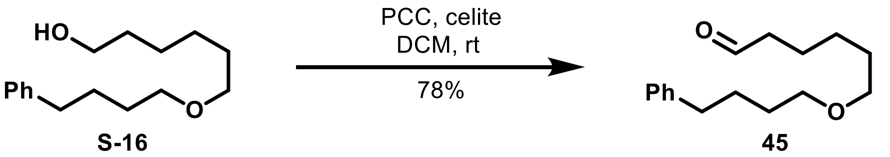

To a solution of **S-16**[10] (910 mg, 3.64 mmol, 1.0 equiv.) and Celite (1.17 g) in CH_2Cl_2 (15 mL) was added pyridinium chlorochromate (PCC) (1.17 g, 5.46 mmol, 1.5 equiv.). The reaction was stirred vigorously for 8 h at room temperature. The reaction solution was then filtered, and the filtrate was concentrated *in vacuo*. The crude mixture was purified by silica gel column chromatography (hexanes/EtOAc = 10:1) to give aldehyde

45 (0.7 g, 78%) as a colorless oil.

Physical State: colorless oil.

***R**$_f$* = 0.57 (hexanes/EtOAc 8:1, UV).

^{1}H NMR (600 MHz, CDCl$_3$) δ 9.76 (s, 1H), 7.27 (d, *J* = 6.8 Hz, 2H), 7.18 (d, *J* = 7.3 Hz, 3H), 3.44 – 3.37 (m, 4H), 2.63 (t, *J* = 7.6 Hz, 2H), 2.43 (td, *J* = 7.4, 1.8 Hz, 2H), 1.74 – 1.54 (m, 8H), 1.48 – 1.34 (m, 2H).

^{13}C NMR (151 MHz, CDCl$_3$) δ 202.8, 142.6, 128.6, 128.4, 125.8, 70.9, 70.7, 44.0, 35.9, 29.6, 29.5, 28.2, 26.0, 22.1.

HRMS: Calc'd for $C_{16}H_{24}O_2Na$, $[M+Na]^+$ 271.1669; found 271.1671.

Synthesis of 46

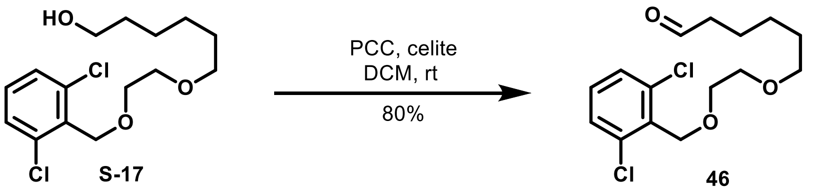

To a solution of **S-I7**[11] (1.80 g, 5.62 mmol, 1.0 equiv.) and Celite (1.45 g) in CH_2Cl_2 (28 mL) was added pyridinium chlorochromate (PCC) (1.45 g, 6.75 mmol, 1.2 equiv.). The reaction was stirred vigorously for 8 h at room temperature. The reaction solution was then filtered, and the filtrate was concentrated *in vacu*o, and the crude mixture was purified by silica gel column chromatography (hexanes/EtOAc = 10:1) to give **46** (1.4 g, 80%) as a colorless oil.

Physical State: colorless oil.

***R**$_f$* = 0.55 (hexanes/EtOAc 8:1, UV).

^{1}H NMR (600 MHz, CDCl$_3$) δ 9.75 (t, *J* = 1.8 Hz, 1H), 7.30 (d, *J* = 8.0 Hz, 2H), 7.17 (t, *J* = 8.0 Hz, 1H), 4.82 (s, 2H), 3.69 (dd, *J* = 5.8, 3.9 Hz, 2H), 3.60 (dd, *J* = 5.8, 4.0 Hz, 2H), 3.46 (t, *J* = 6.5 Hz, 2H), 2.42 (td, *J* = 7.4, 1.8 Hz, 2H), 1.69 – 1.55 (m, 4H), 1.44 – 1.34 (m, 2H).

^{13}C NMR (151 MHz, CDCl$_3$) δ 202.9, 137.1, 133.5, 130.0, 128.5, 71.1, 70.2, 70.1, 67.6, 44.0, 29.6, 25.9, 22.0.

HRMS: Calc'd for $C_{15}H_{20}Cl_2O_3Na$, $[M+Na]^+$ 341.0682; found 341.0683.

Synthesis of (*R*)-salmeterol (3)

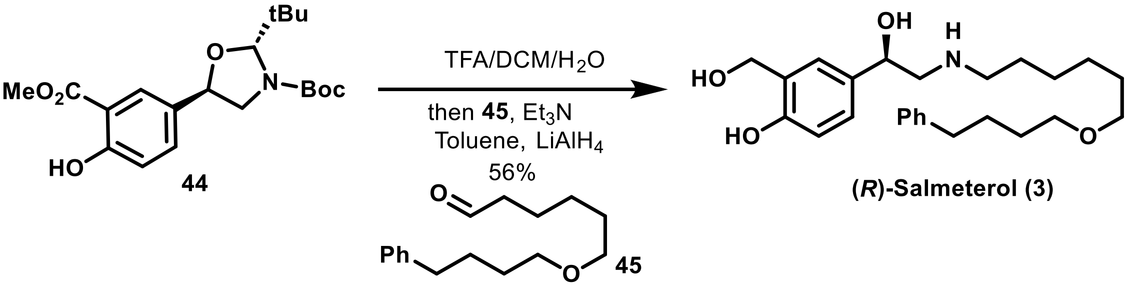

To a solution of **44** (100 mg, 0.26 mmol, 1.0 equiv.) in CH_2Cl_2 (2.0 mL) was added H_2O (0.2 mL) and trifluoroacetic acid (TFA) (1.0 mL). The reaction was vigorously stirred for 24 h at room temperature. The solvent was removed under reduced pressure to give a colorless oil. Toluene (4 mL) was added to dissolve the oil, then triethylamine (54 µL, 0.40 mmol, 1.5 equiv.) and aldehyde **45** (98 mg, 0.40 mmol, 1.5 equiv.) were added in a single portion. The mixture was heated to reflux until no further water was collected in a Dean-Stark trap. The reaction was cooled to 0 °C in an ice/water bath, and $LiAlH_4$ (1.40 mL, 1.32 mmol, 5.0 equiv., 1 M in the THF) was slowly added dropwise. Upon complete addition, the resulting mixture was warmed to ambient temperature. The starting material was consumed after 4 h, the reaction was submerged into an ice/water bath and quenched by slow addition of *sat. aq.* potassium sodium tartrate (4.0 mL) and diluted with EtOAc (4.0 mL). The mixture was stirred vigorously at room temperature until the two phases separated. The aqueous phase was extracted with EtOAc (20 mL) for three times. The combined organic phases were washed with brine, dried over Na_2SO_4, filtered and concentrated. The crude product was purified by flash column chromatography (CH_2Cl_2/MeOH/$NH_3 \cdot H_2O$ = 4:1:0.05) to give the desired benzylic alcohol product **(*R*)-salmeterol (3)**[12] (61 mg, 56%) as a colorless oil.

Physical State: colorless oil.

R_f = 0.32 (CH_2Cl_2/MeOH/$NH_3 \cdot H_2O$ = 4:1:0.05 4:1, UV).

$[\alpha]_D^{25}$ = -5.8 (c 0.2, $CHCl_3$).

^{1}H NMR (600 MHz, CD_3OD) δ 7.30 (d, *J* = 2.3 Hz, 1H), 7.27 – 7.21 (m, 2H), 7.19 –

7.09 (m, 4H), 6.76 (d, *J* = 8.2 Hz, 1H), 4.73 (dd, *J* = 9.1, 4.1 Hz, 1H), 4.65 (s, 2H), 3.44 (t, *J* = 6.4 Hz, 2H), 3.41 (t, *J* = 6.5 Hz, 1H), 2.85 (dd, *J* = 12.3, 9.1 Hz, 1H), 2.79 (dd, *J* = 12.2, 4.2 Hz, 1H), 2.75 – 2.66 (m, 2H), 2.62 (t, *J* = 7.6 Hz, 2H), 1.72 – 1.63 (m, 2H), 1.63 – 1.50 (m, 6H), 1.42 – 1.30 (m, 2H).

^{13}C NMR (151 MHz, CD_3OD) δ 156.0, 143.7, 134.6, 129.4, 129.3, 128.7, 127.1, 127.0, 126.7, 115.9, 72.4, 71.8, 71.7, 61.0, 57.3, 50.0, 36.6, 30.6, 30.3, 29.6, 29.3, 28.0, 27.1.

HRMS: Calc'd for $C_{26}H_{37}NO_4$, $[M+H]^+$ 416.2796; found 416.2796.

Synthesis of (*R*)-vilanterol (47)

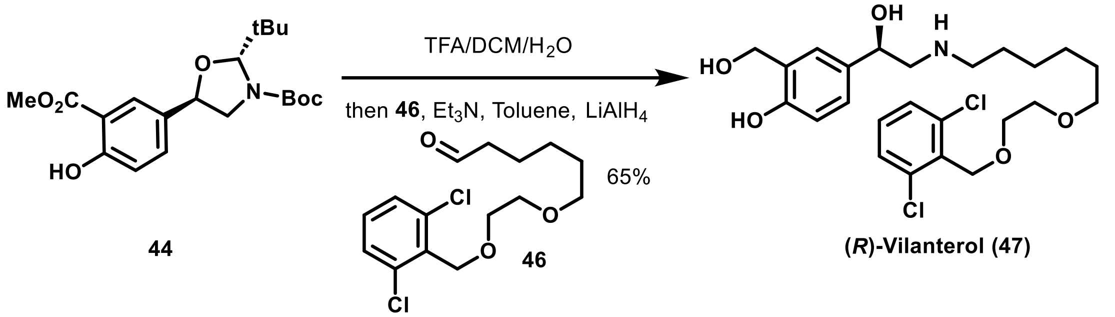

To a solution of **44** (50 mg, 0.13 mmol, 1.0 equiv.) in CH_2Cl_2 (1.0 mL) was added H_2O (0.1 mL) and trifluoroacetic acid (TFA) (0.5 mL), the reaction was stirred vigorously for 24 h at room temperature. The solvent was removed under reduced pressure to give a colorless oil. Toluene (4 mL) was added to dissolve the oil, then triethylamine (27 µL, 0.20 mmol, 1.5 equiv.) and aldehyde **46** (63 mg, 0.20 mmol, 1.5 equiv.) were added in a single portion. The mixture was heated to reflux until no further water was collected in a Dean-Stark trap. The reaction was cooled to 0 °C in an ice/water bath, and $LiAlH_4$ (0.70 mL, 0.66 mmol, 5.0 equiv, 1 M in the THF) was slowly added dropwise. Upon complete addition, the resulting mixture was warmed to ambient temperature. The starting material was consumed after 4 h, the reaction was submerged into an ice/water bath and quenched by slow addition of *sat. aq.* potassium sodium tartrate (2.0 mL) and diluted with EtOAc (2.0 mL). The mixture was stirred vigorously at room temperature until the two phases separated. The aqueous phase was extracted with EtOAc (10 mL) for three times. The combined organic phases were washed with brine, dried over

Na_2SO_4, filtered and concentrated. The crude product was purified by flash column chromatography (CH_2Cl_2/MeOH/$NH_3 \cdot H_2O$ = 4:1:0.05) to give the desired benzylic alcohol product **(*R*)-vilanterol (47)** [13] (42 mg, 65%) as a colorless oil.

Physical State: colorless oil.

R_f = 0.32 (CH_2Cl_2/MeOH/$NH_3 \cdot H_2O$ = 4:1:0.05 4:1, UV).

$[\alpha]_D^{25}$ = +7.9 (c 0.2, $CHCl_3$).

^{1}H NMR (600 MHz, CD_3OD) δ 7.41 – 7.35 (d, *J* = 8.1 Hz, 2H), 7.32 – 7.26 (m, 2H), 7.12 (dd, *J* = 8.3, 2.3 Hz, 1H), 6.76 (d, *J* = 8.2 Hz, 1H), 4.83 (s, 2H), 4.73 (dd, *J* = 9.1, 4.1 Hz, 1H), 4.65 (s, 2H), 3.73 – 3.68 (m, 2H), 3.63 – 3.58 (m, 2H), 3.48 (t, *J* = 6.5 Hz, 2H), 2.86 (dd, *J* = 12.3, 9.1 Hz, 1H), 2.81 (dd, *J* = 12.4, 4.2 Hz, 1H), 2.76 – 2.66 (m, 2H), 1.61 – 1.53 (m, 4H), 1.43 – 1.31 (m, 2H).

^{13}C NMR (151 MHz, CD_3OD) δ 156.0, 138.0, 134.6, 134.5, 131.6, 129.6, 127.1, 127.0, 115.9, 72.3, 72.2, 71.3, 71.1, 68.4, 61.0, 57.3, 50.0, 49.4, 30.6, 29.5, 27.9, 27.0.

HRMS: Calc'd for $C_{24}H_{34}Cl_2NO_5$, $[M+H]^+$ 486.1809; found 486.1811.

Summary and Comparison with Previous Routes

(*R*)-2-(methylamino)-1-(pyridin-3-yl)ethan-1-ol (27) [4]

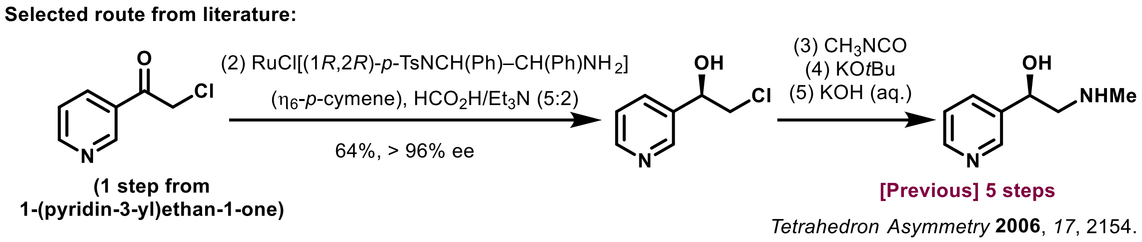

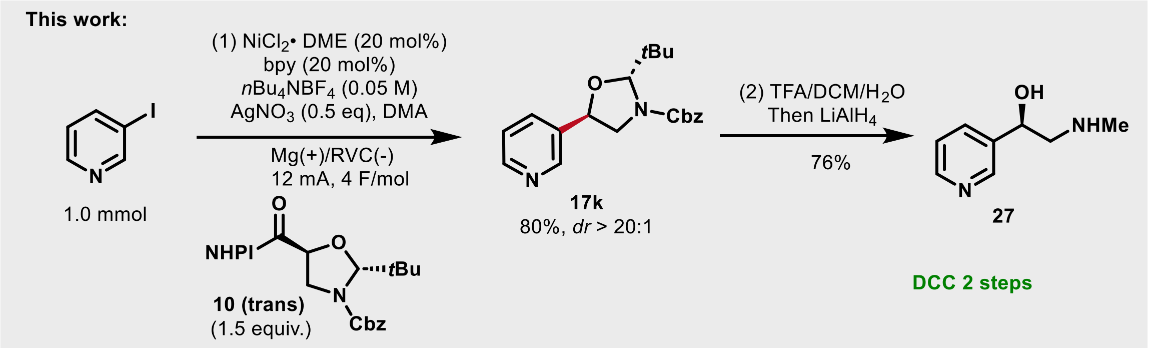

(*R*)-2-amino-1-(4-amino-3,5-dichlorophenyl)ethan-1-ol (28)[5]

Selected route from literature:

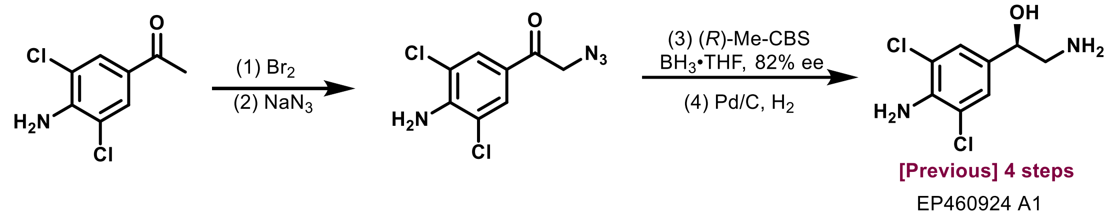

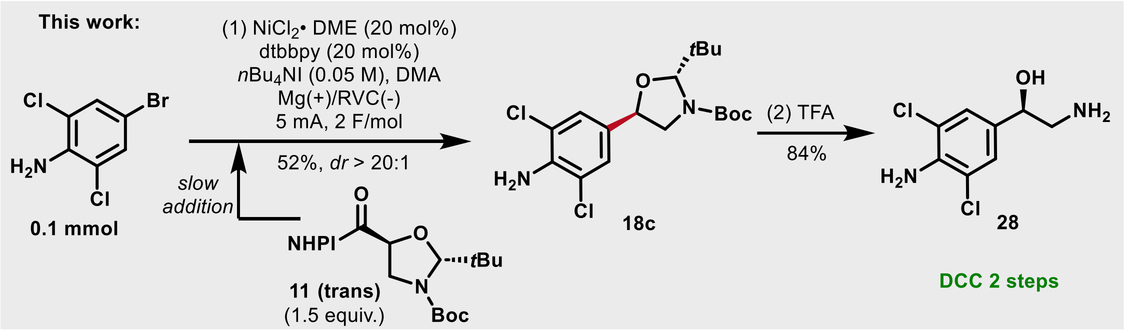

(*S*)-Econazole (1) [6]

Selected route from literature:

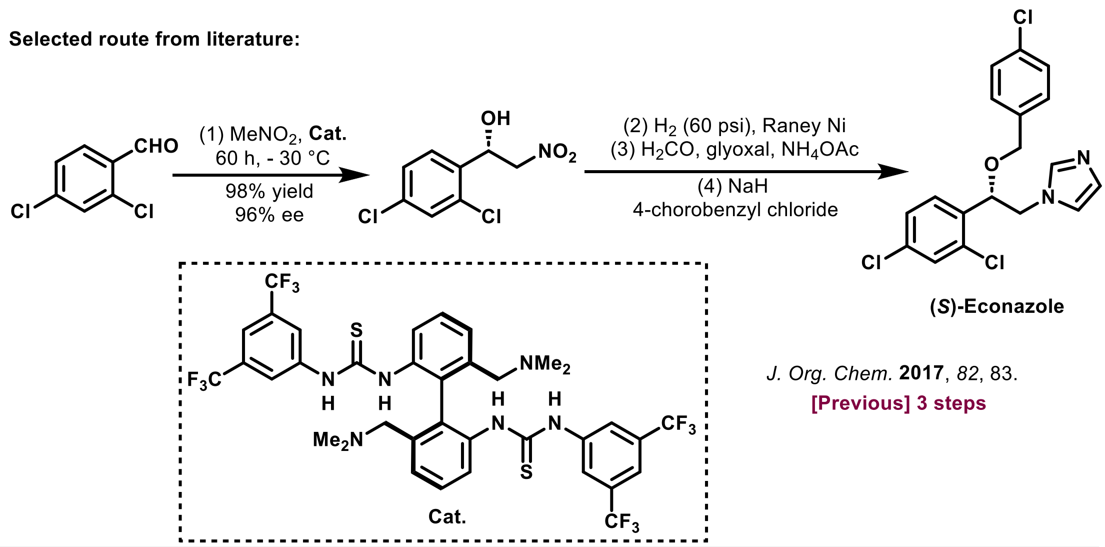

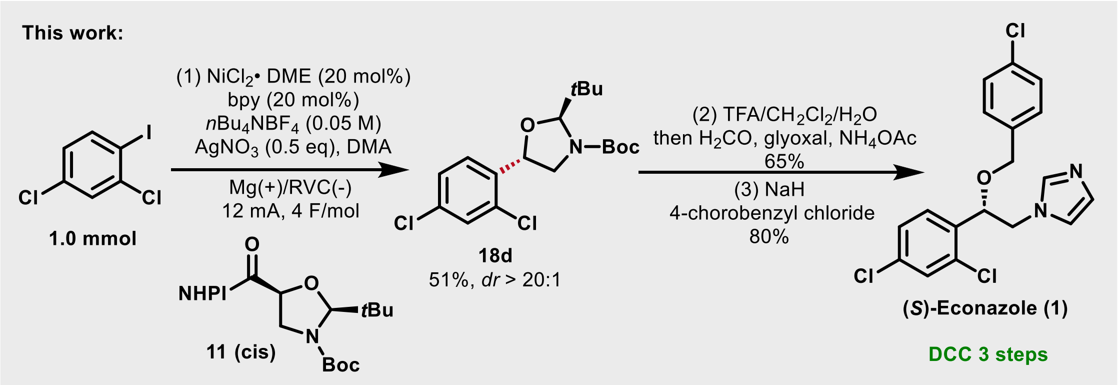

GSK-656 (4)[7]

Selected route from literature:

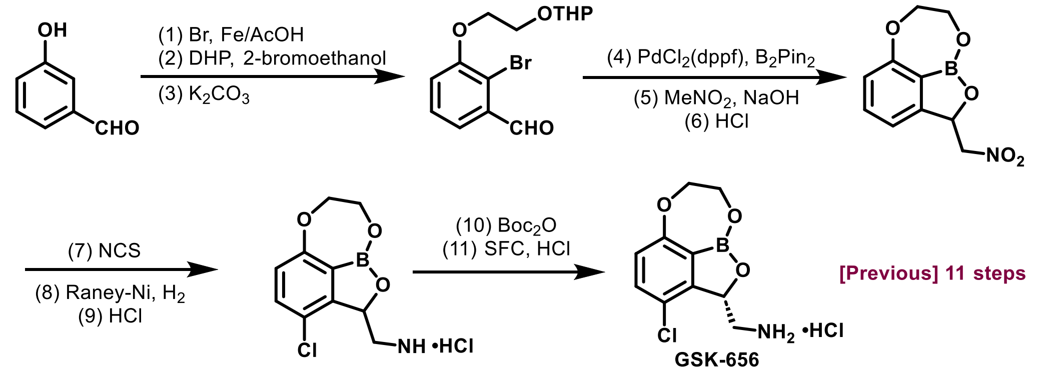

Selected route from literature:

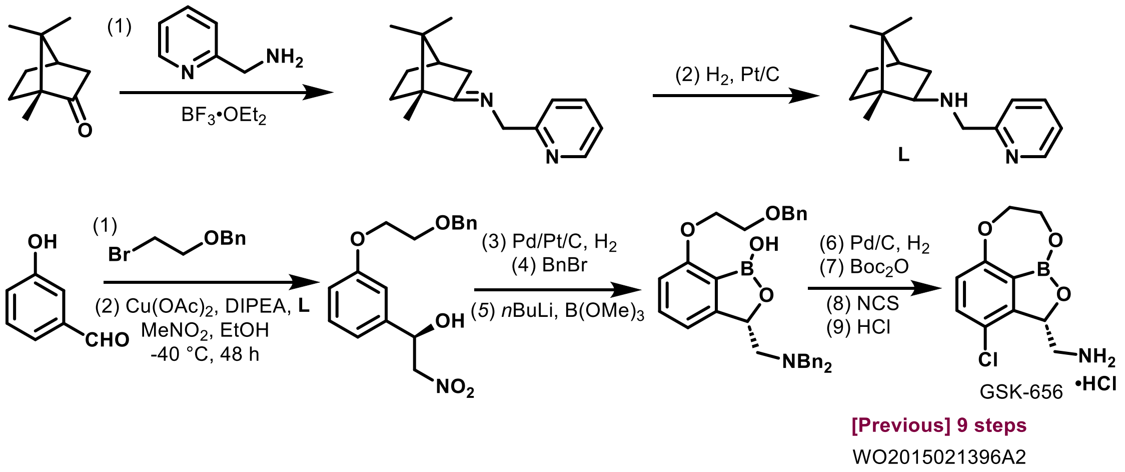

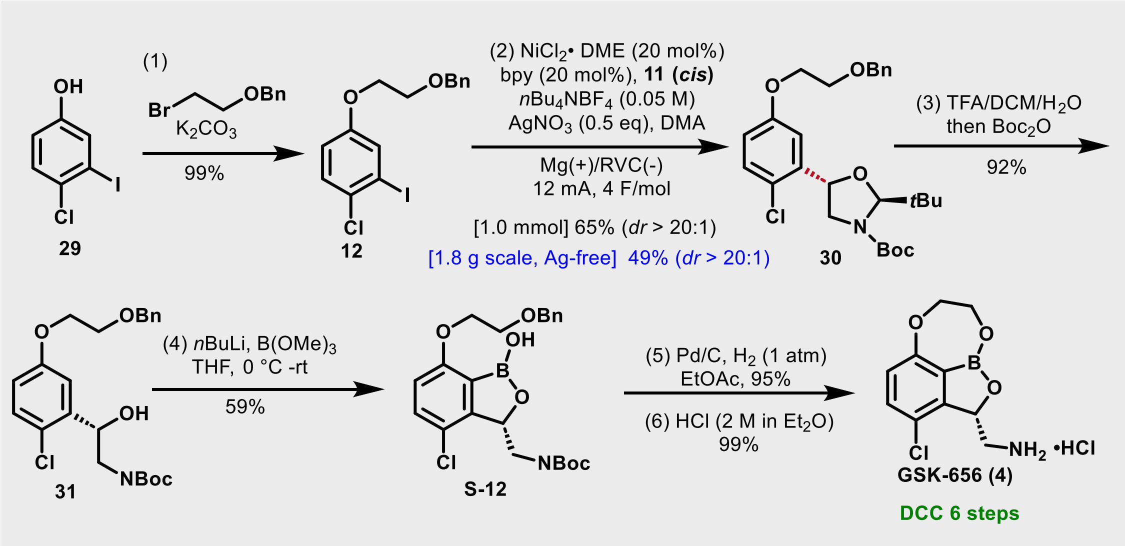

Indacaterol (2) [9, 14, 15, 16]

Selected route from literature:

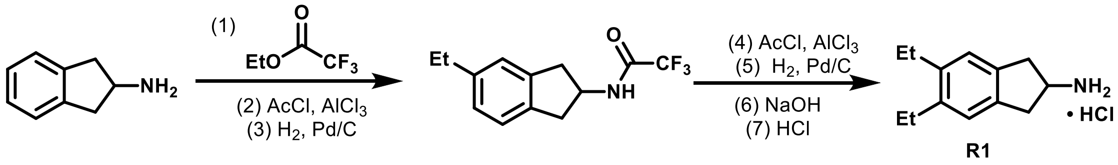

Org. Process Res. Dev. **2006**, *10*, 135.

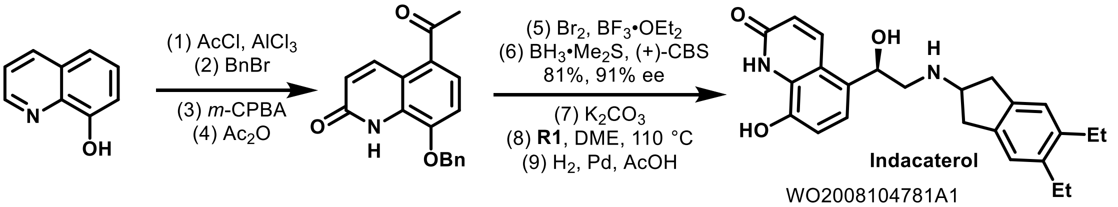

WO2008104781A1
Bioorg. Med. Chem. **2011**, *19*, 1136.
J. Med. Chem. **2010**, *53*, 3675.

[Previous] 9 steps

This work:

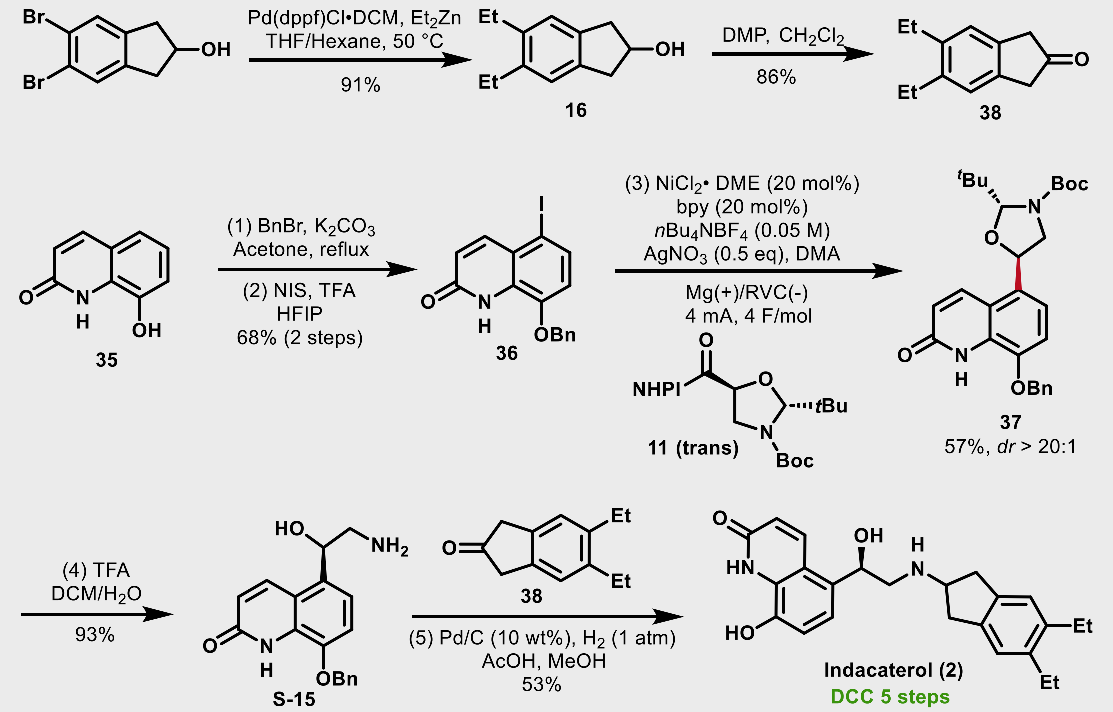

(*R*)-Salmeterol (3)[12] and (*R*)-Vilanterol (47)[13]

Selected route from literature:

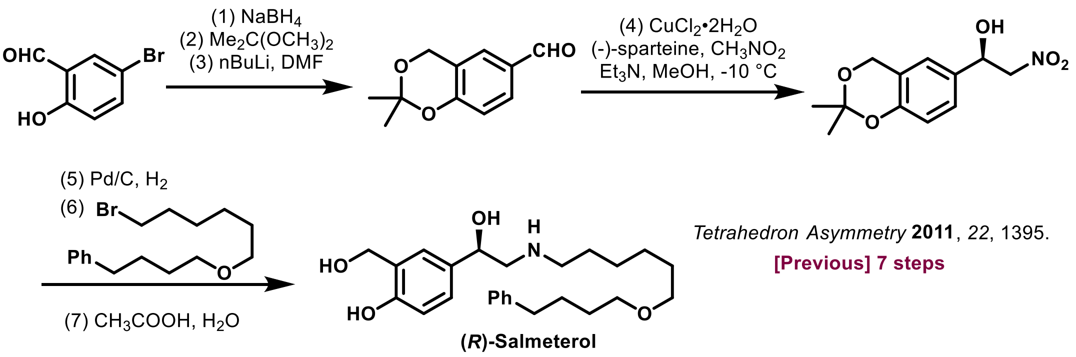

Selected route from literature:

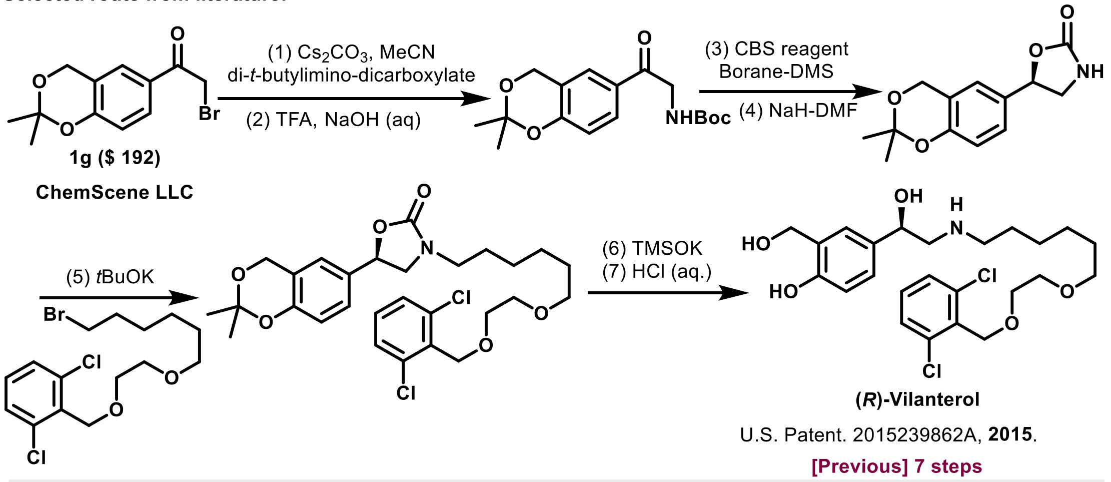

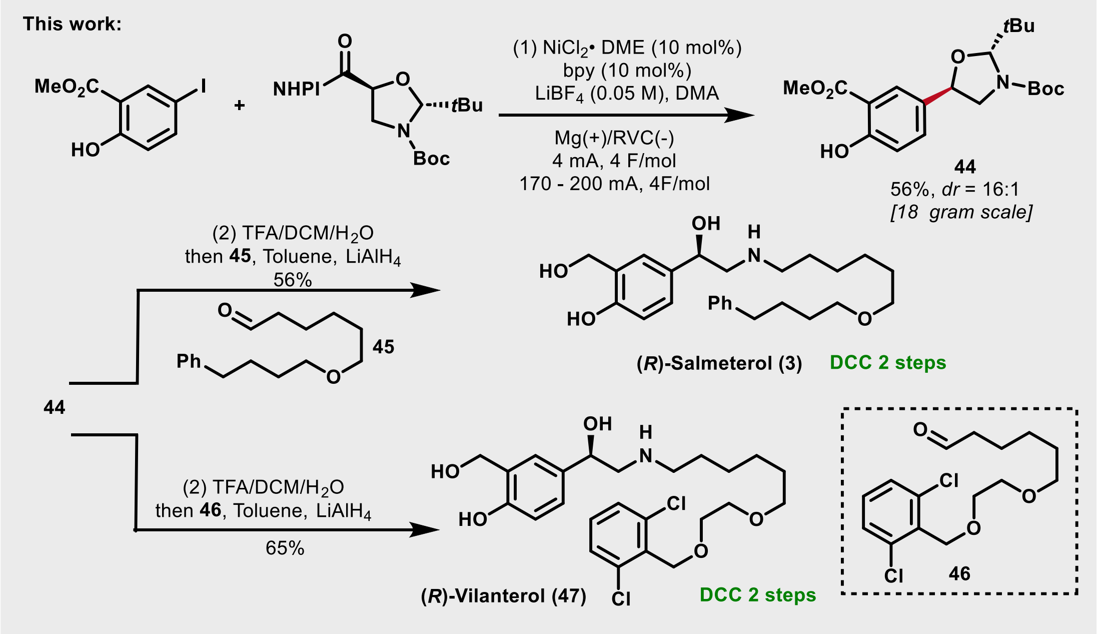

X-Ray Structure

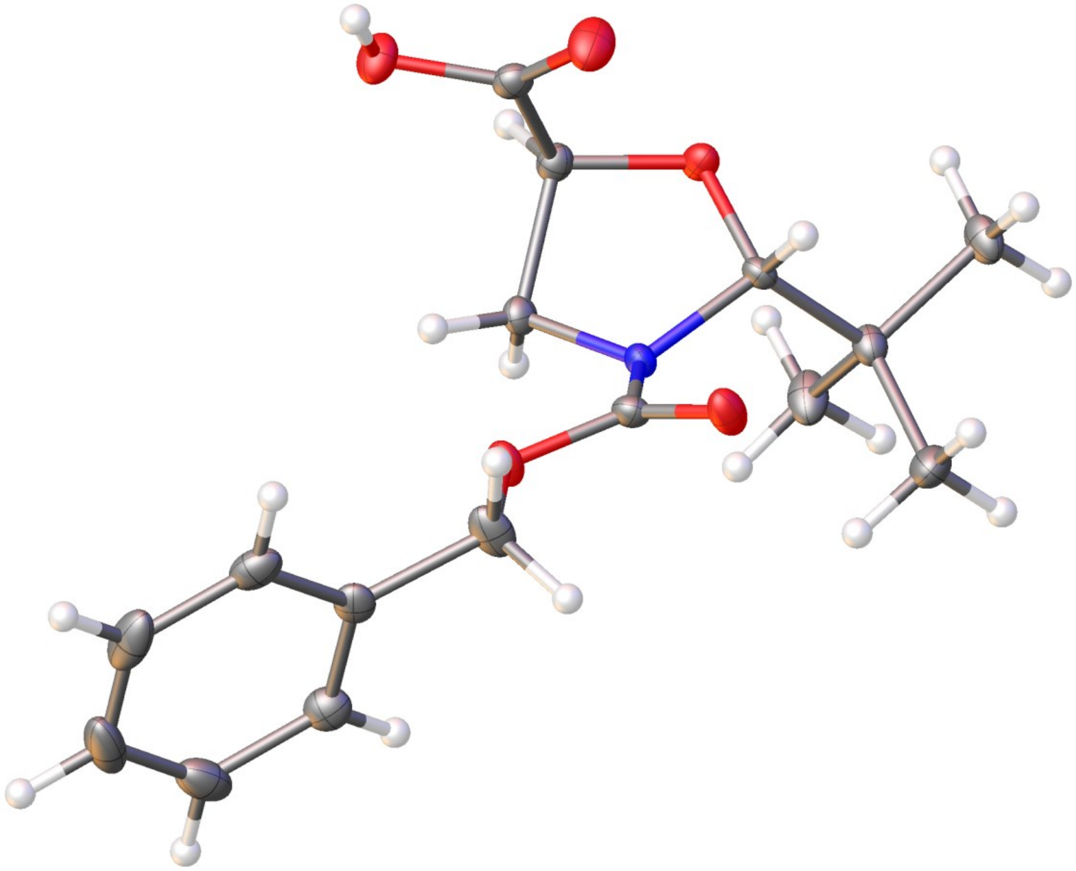

Figure S1. X-ray structure of compound 10 (*trans*-acid).

Method for crystal growth

Compound **10** (*trans*-acid) (5 mg) was dissolved in EtOAc (0.2 mL). With slow evaporation of the solvent, crystal suitable for X-ray diffraction was obtained.

Table 1. Crystal data and structure refinement for **compound 10 (*trans*-acid)**.	
Report date	2023-09-06
Identification code	CCDC 2293697
Empirical formula	C16 H21 N O5
Molecular formula	C16 H21 N O5
Formula weight	307.34
Temperature	100.15 K
Wavelength	1.54178 Å
Crystal system	Orthorhombic
Space group	$P2_12_12_1$

Unit cell dimensions	a = 5.7057(4) Å	α= 90°.
	b = 12.3143(9) Å	β= 90°.
	c = 22.8124(16) Å	γ = 90°.
Volume	1602.8(2) Å^3	
Z	4	
Density (calculated)	1.274 Mg/m^3	
Absorption coefficient	0.785 mm^{-1}	
F(000)	656	
Crystal size	0.21 x 0.18 x 0.16 mm^3	
Crystal color, habit	colorless block	
Theta range for data collection	3.875 to 70.172°.	
Index ranges	-6<=h<=6, -14<=k<=15, -27<=l<=27	
Reflections collected	25743	
Independent reflections	3035 [R(int) = 0.0421]	
Completeness to theta = 67.679°	100.0 %	
Absorption correction	Semi-empirical from equivalents	
Max. and min. transmission	0.7533 and 0.6634	
Refinement method	Full-matrix least-squares on F^2	
Data / restraints / parameters	3035 / 0 / 205	
Goodness-of-fit on F^2	1.090	
Final R indices [I>2sigma(I)]	R1 = 0.0257, wR2 = 0.0652	
R indices (all data)	R1 = 0.0257, wR2 = 0.0652	
Absolute structure parameter	0.05(3)	
Largest diff. peak and hole	0.222 and -0.189 e.Å^{-3}	

Reference

[1] J Sloane, J. L.; Santos, A. B.; Simmons, E. M.; Sherwood, T. C. *Org. Lett.* **2023**, *25*, 4219.

[2] Lisa Marie Kammer, L.M.; Shorouk O. Badir, S.O.; Hu, R.-M., Molander, G.A. *Chem. Sci.* **2021**, *12*, 5450.

[3] Vuljanic, T.; Kihlberg, J.; Somfai, P. *J. Org. Chem.* **1998**, *63*, 279.

[4] Tanis, S. P.; Evans, B. R.; Nieman, J. A.; Parker, T. T.; Taylor, W. D.; Heasley, S. E.; Herrinton, P. M.; Perrault, W. R.; Hohler, R. A.; Dolak, L. A. *Tetrahedron Asymmetry* **2006**, *17*, 2154.

[5] Judkins, B. D.; Evans, B.; Meadows, J. D. Preparation of Pyridine Derivative Having Selective β2-Adrenoreceptor-Stimulant Activity. EP0460924A1, **1991**.

[6] Otevrel, J.; Bobal, P. *J. Org. Chem.* **2017**, *82*, 8342.

[7] Alley, M.R.K.; Hernandez, V. S.; Plattner, J. J.; Li, X.; Barros-Aguirre, D.; Giordano, I. Tricyclic Benzoxaborole Compounds and Uses Thereof, WO2015021396A2, **2015**.

[8] Gaddam, P. R.; Gaddam, S. R.; Gaddam, M. R.; Mosali, U. K. R. A Process for Preparing Batefenterol and Intermediates Thereof, WO2022175982A1, **2022**.

[9] Baur, F.; Beattie, D.; Beer, D.; Bentley, D.; Bradley, M.; Bruce, I.; Charlton, S. J.; Cuenoud, B.; Ernst, R.; Fairhurst, R. A. *J. Med. Chem.* **2010**, *53*, 3675.

[10] Bream, R. N.; Ley, S. V.; Procopiou, P. A. *Org. Lett.* **2002**, *4*, 3793.

[11] Zhang, Z.; Zhao, S.; Cai, W..; Zhang, N.; Kong, X.; Ma, X.; Deng, Y.; Sun, J.; Liu, W.; Li, X.; Zhang, B..; Cui, X..; Yang, L.; Fan, Z. Preparation Method of Vilanterol Key Intermediate 2-((2-((6-Bromohexyl)oxy)ethoxy)methyl)-1,3-dichlorobenzene from 2,6-Dichlorobenzyl Alcohol *via* Two-stage Nucleophilic Substitution, Sulfonic Acid Esterification, Refining and Bromination. CN115286491A, **2022**.

[12] Guo, Z.-L.; Deng, Y.-Q.; Zhong, S.; Lu, G. *Tetrahedron Asymmetry* **2011**, *22*, 1395.

[13] Dammalapati, V. L. N. R.; Mudduluru, H. K.; Aduri, R. Process for the Preparation

of Vilanterol and Intermediates Thereof. US20150239862A1, **2015**.

[14] Prashad, M.; Hu, B.; Har, D.; Repic, O.; Blacklock, T. J.; Lohse, O. *Org. Process Res. Dev.* **2006**, *10*, 135.

[15] Kankan, R. N.; Rao, D. R.; Birari, D.; Sawant, A. A. Process for the Preparation of Stereoisomers of Carmoterol, Their Pharmaceutical Compositions, and Use in Therapy, WO2008104781A1, **2004**.

[16] Kevin K.-C. L.; Subas M. S.; Christopher J. O.; Andrew C. F.; Jin L. *Bioorg. Med. Chem.* **2011**, *19*, 1136.

NMR Spectra:

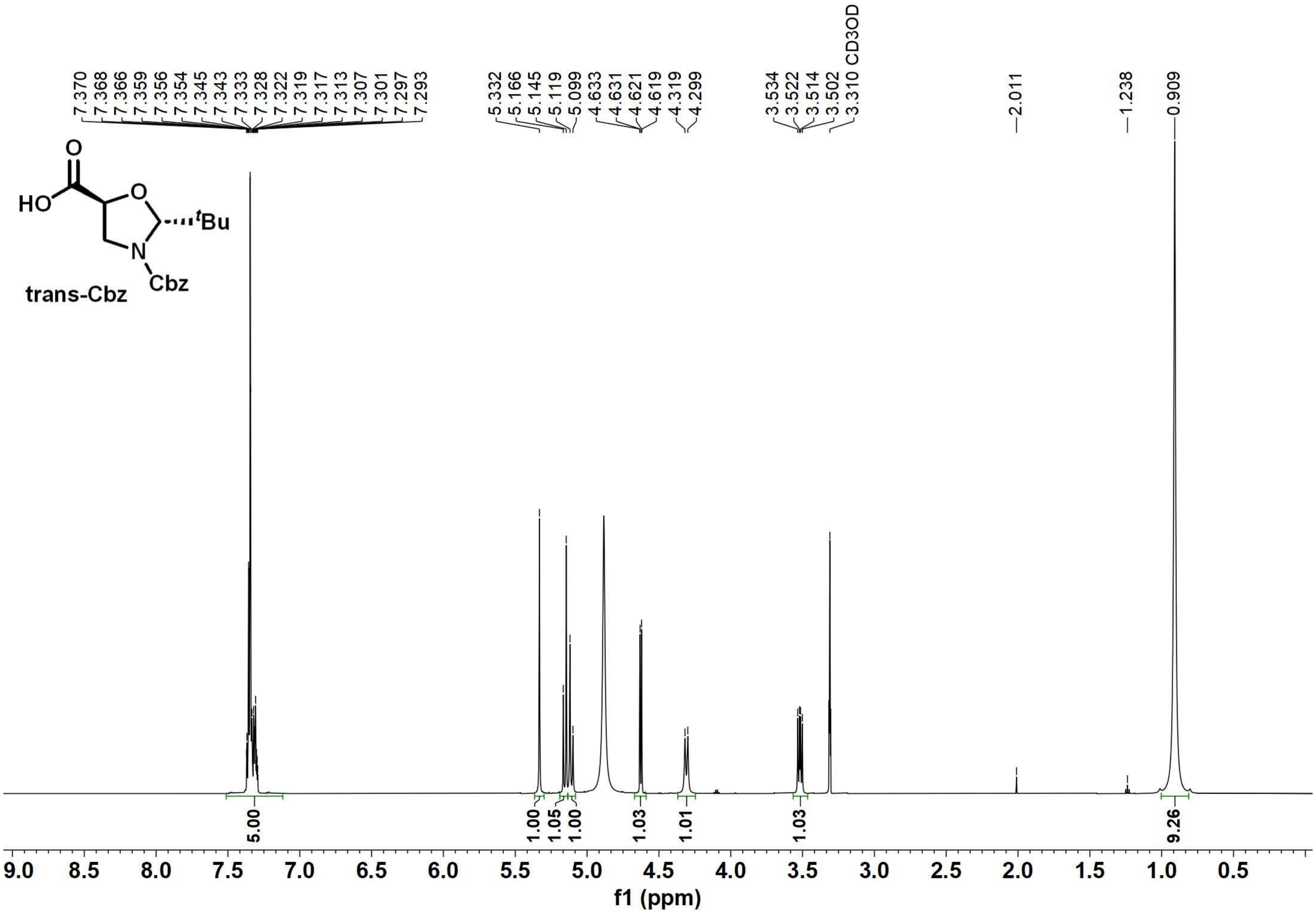

^{1}H NMR of Compound 10 (*trans*-Cbz-acid) (600 MHz, CD_3OD)

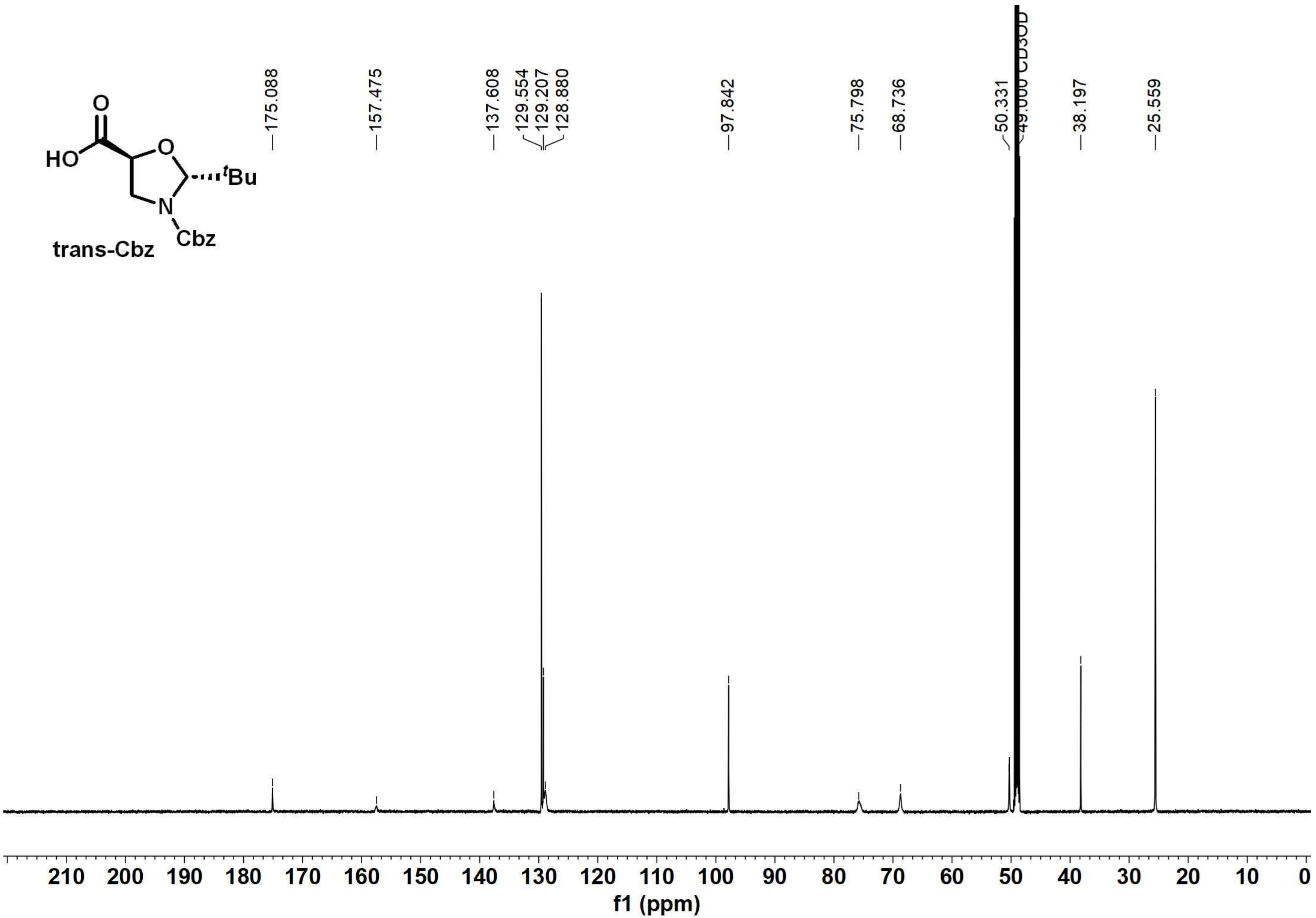

^{13}C NMR of Compound 10 (*trans*-Cbz-acid) (151 MHz, CD_3OD)

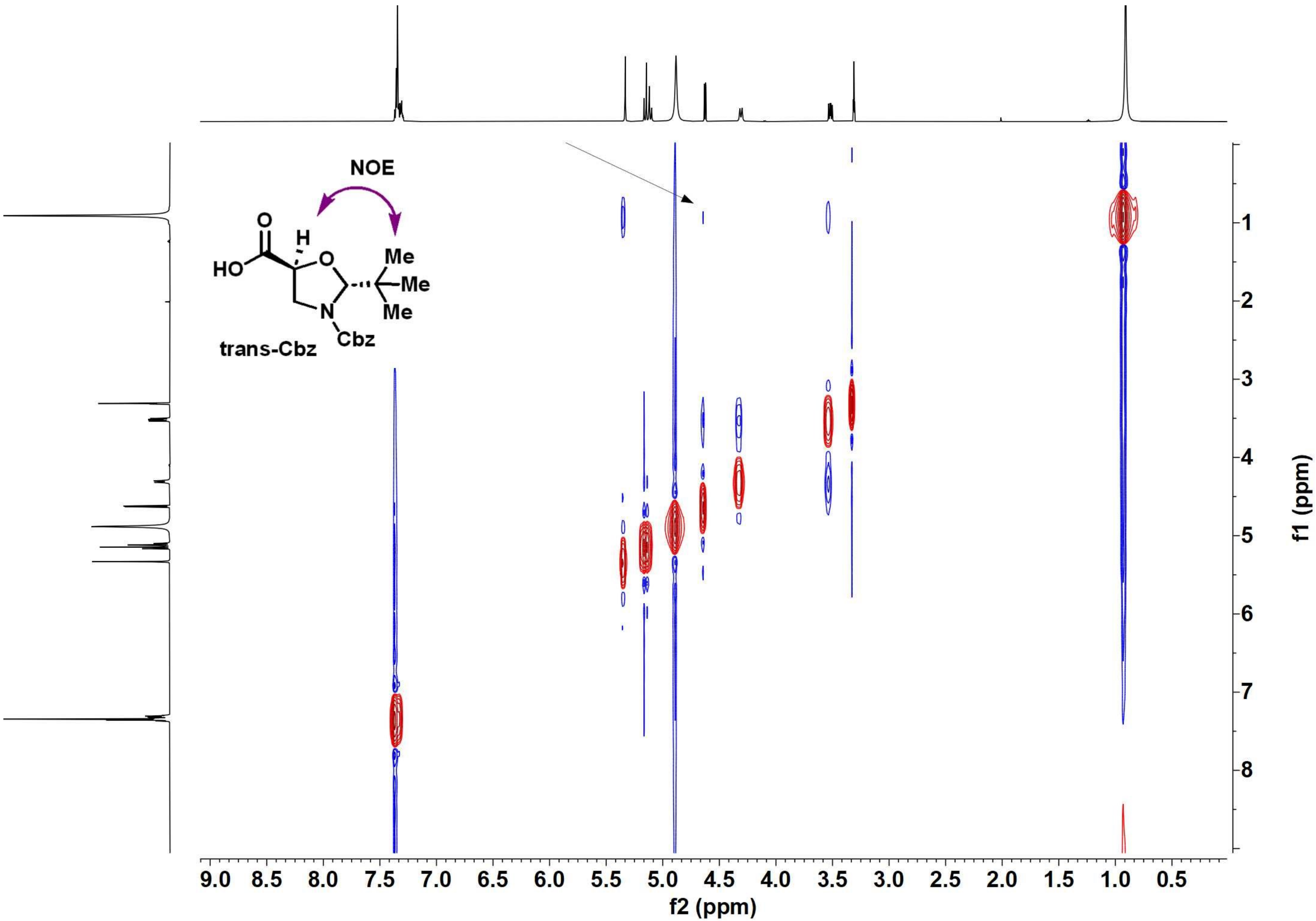

NOESY of Compound 10 (*trans*-Cbz-acid) (600 MHz, CD_3OD)

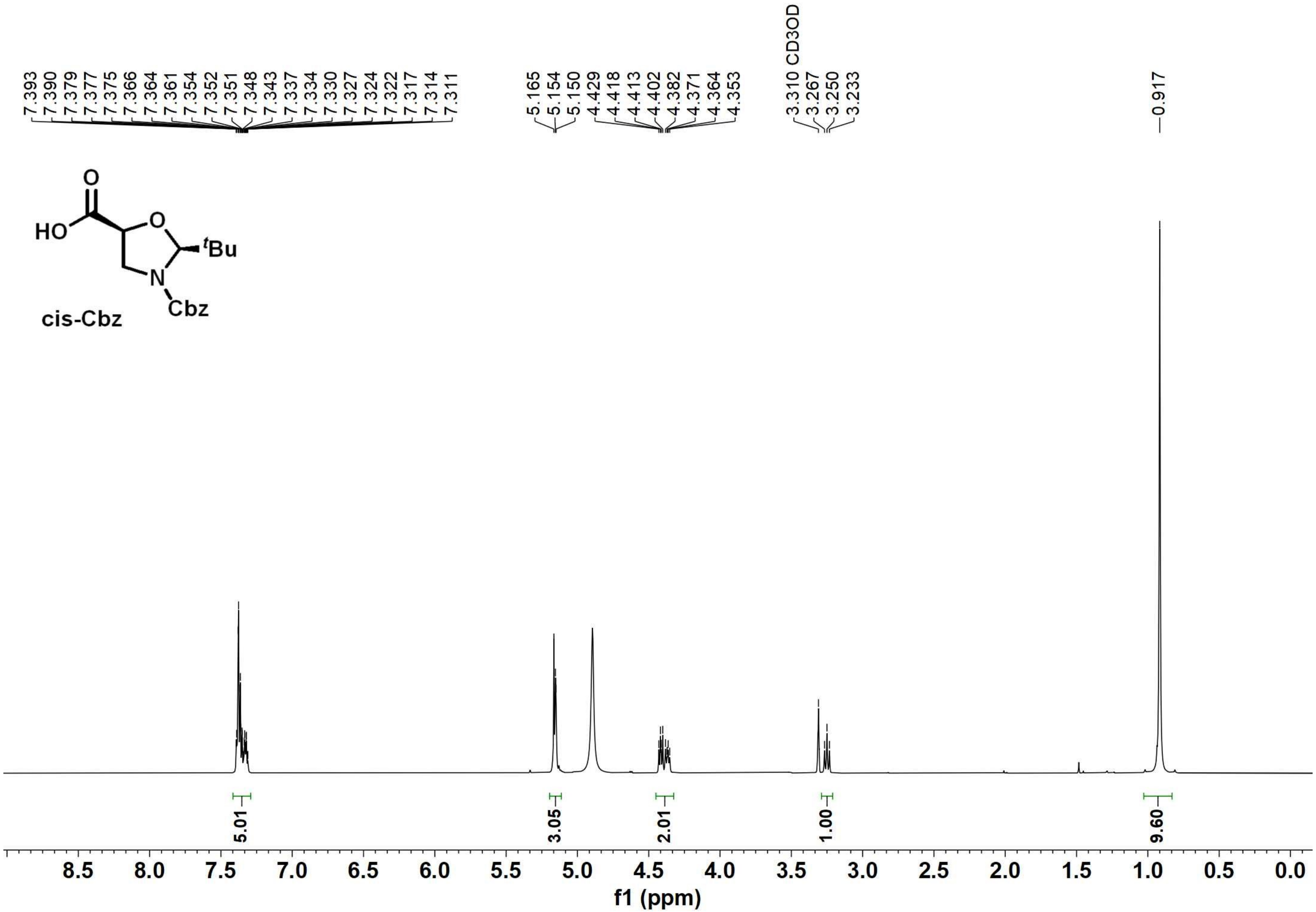

^{1}H NMR of Compound 10 (*cis*-Cbz-acid) (600 MHz, CD_3OD)

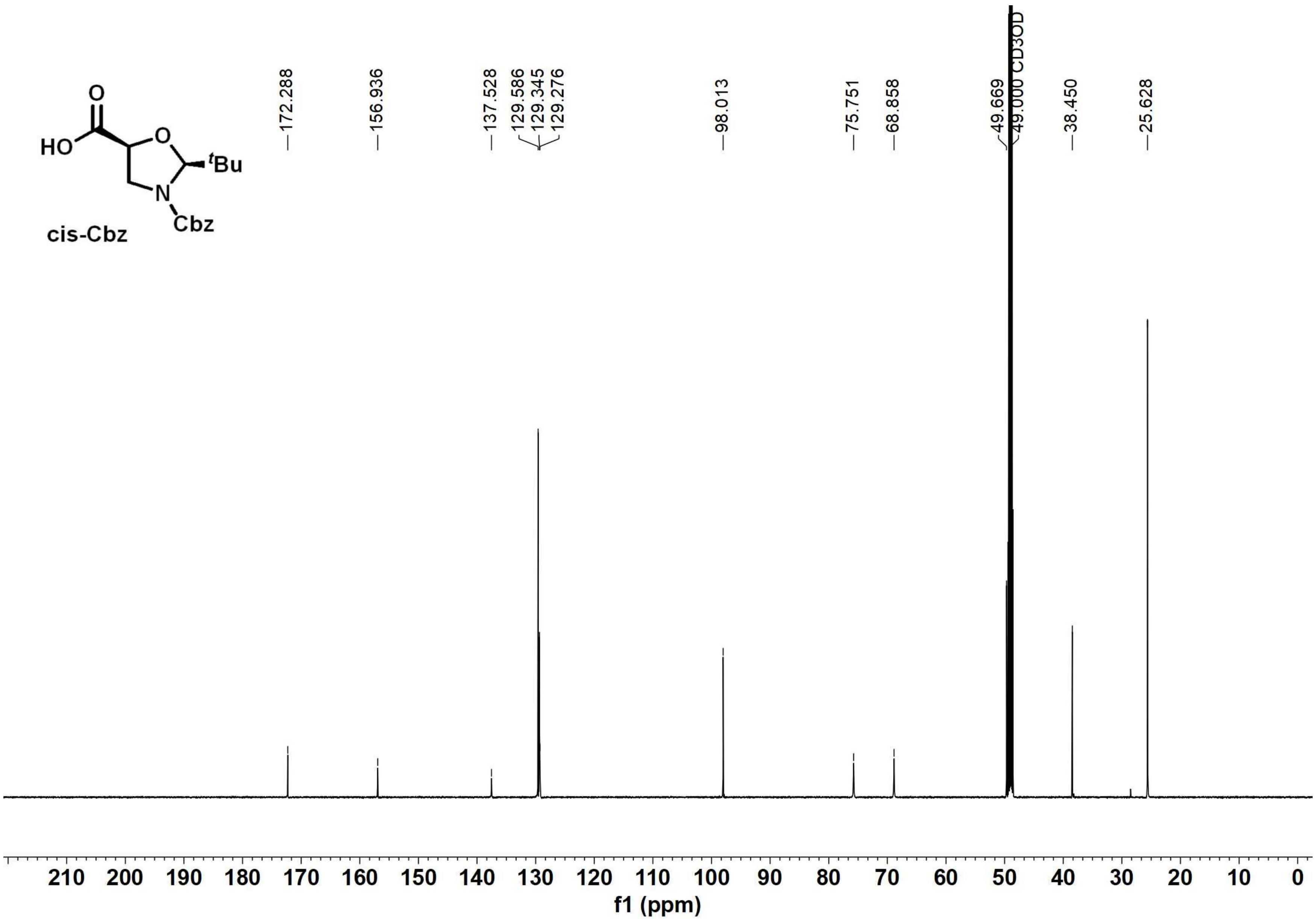

^{13}C NMR of Compound 10 (*cis*-Cbz-acid) (151 MHz, CD_3OD)

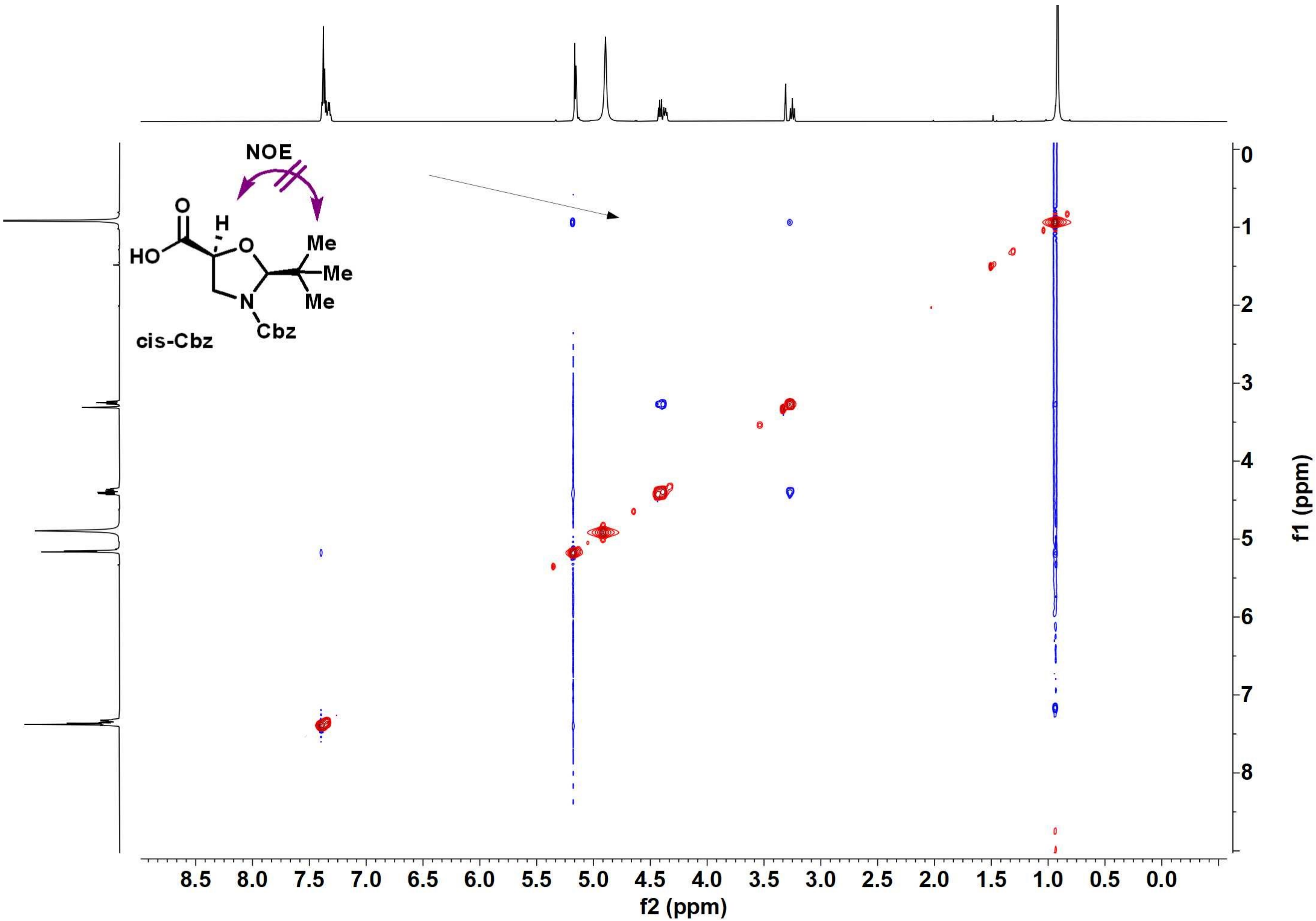

NOESY of Compound 10 (*cis*-Cbz-acid) (600 MHz, CD_3OD)

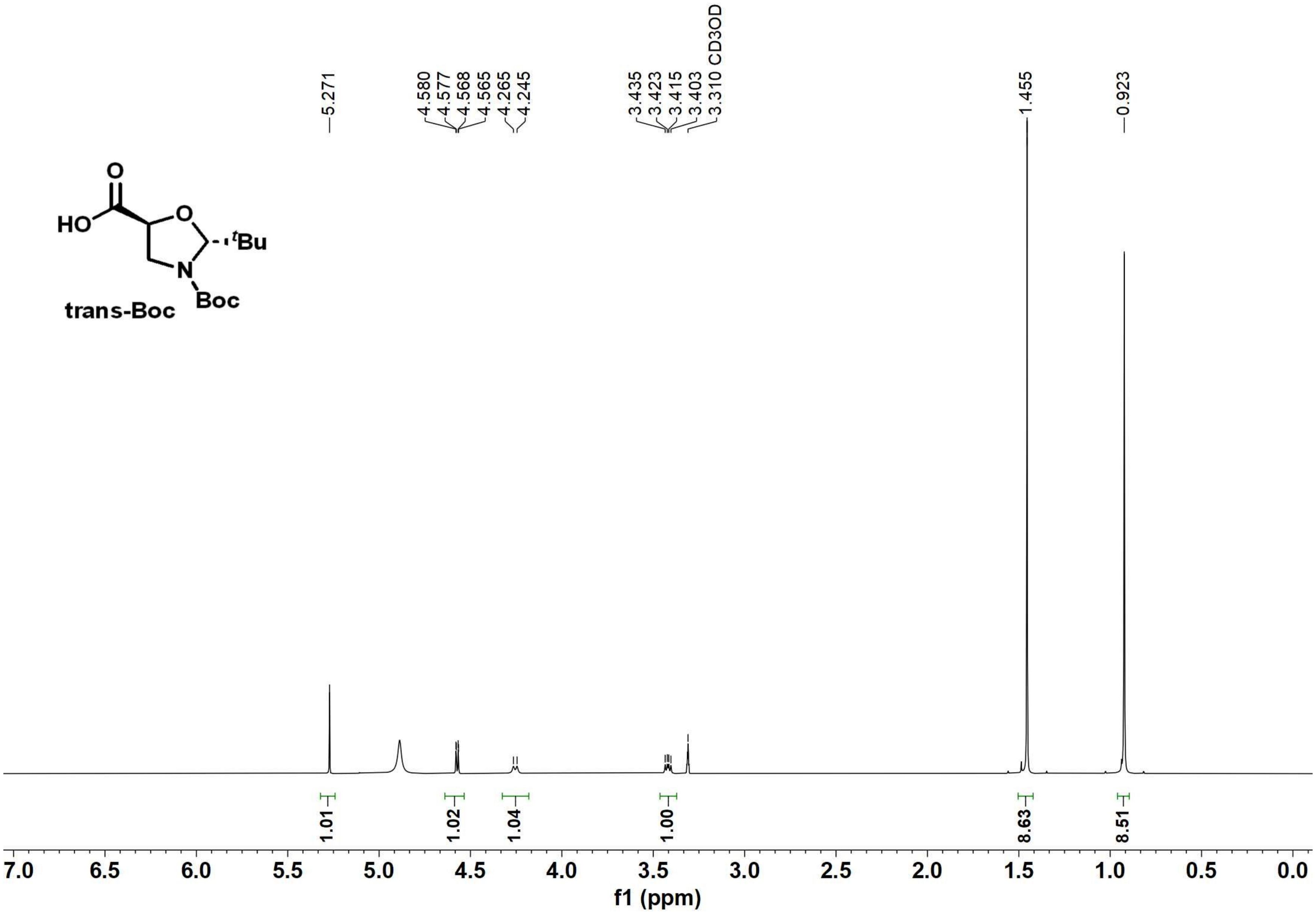

^{1}H NMR of Compound 11 (*trans*-boc-acid) (600 MHz, CD_3OD)

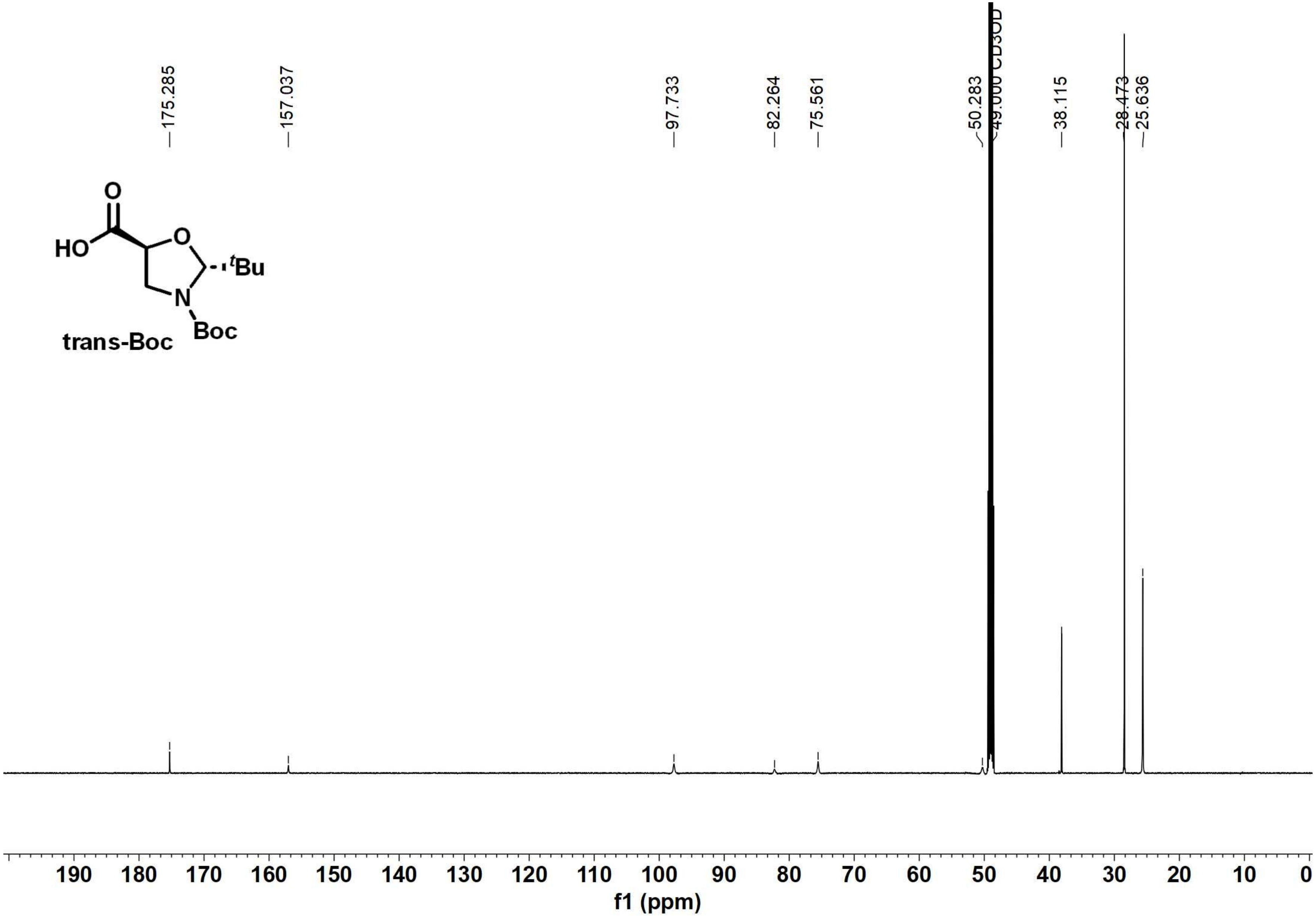

^{13}C NMR of Compound 11 (*trans*-boc-acid) (151 MHz, CD_3OD)

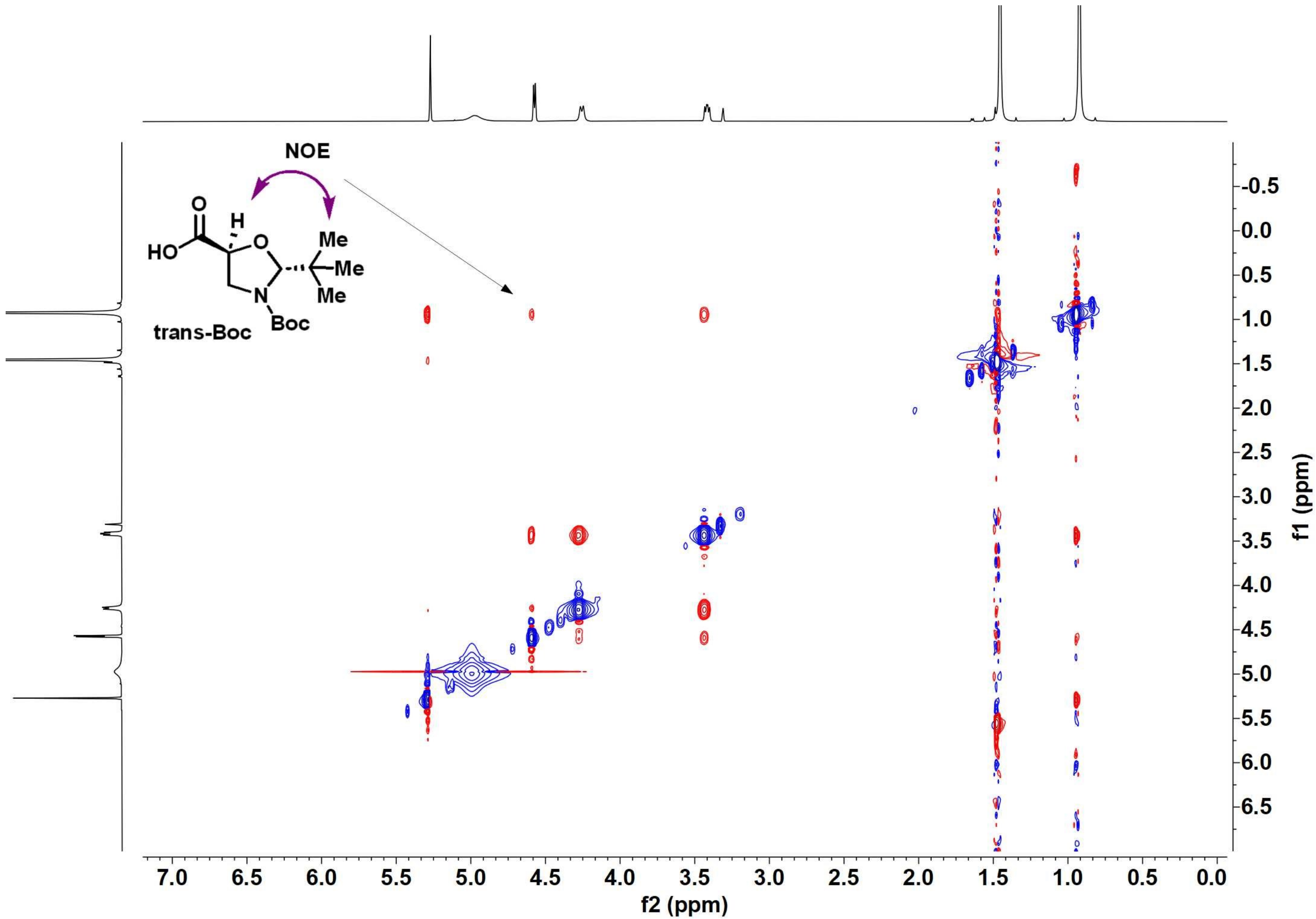

NOESY of Compound 11 (*trans*-boc-acid) (600 MHz, CD_3OD)

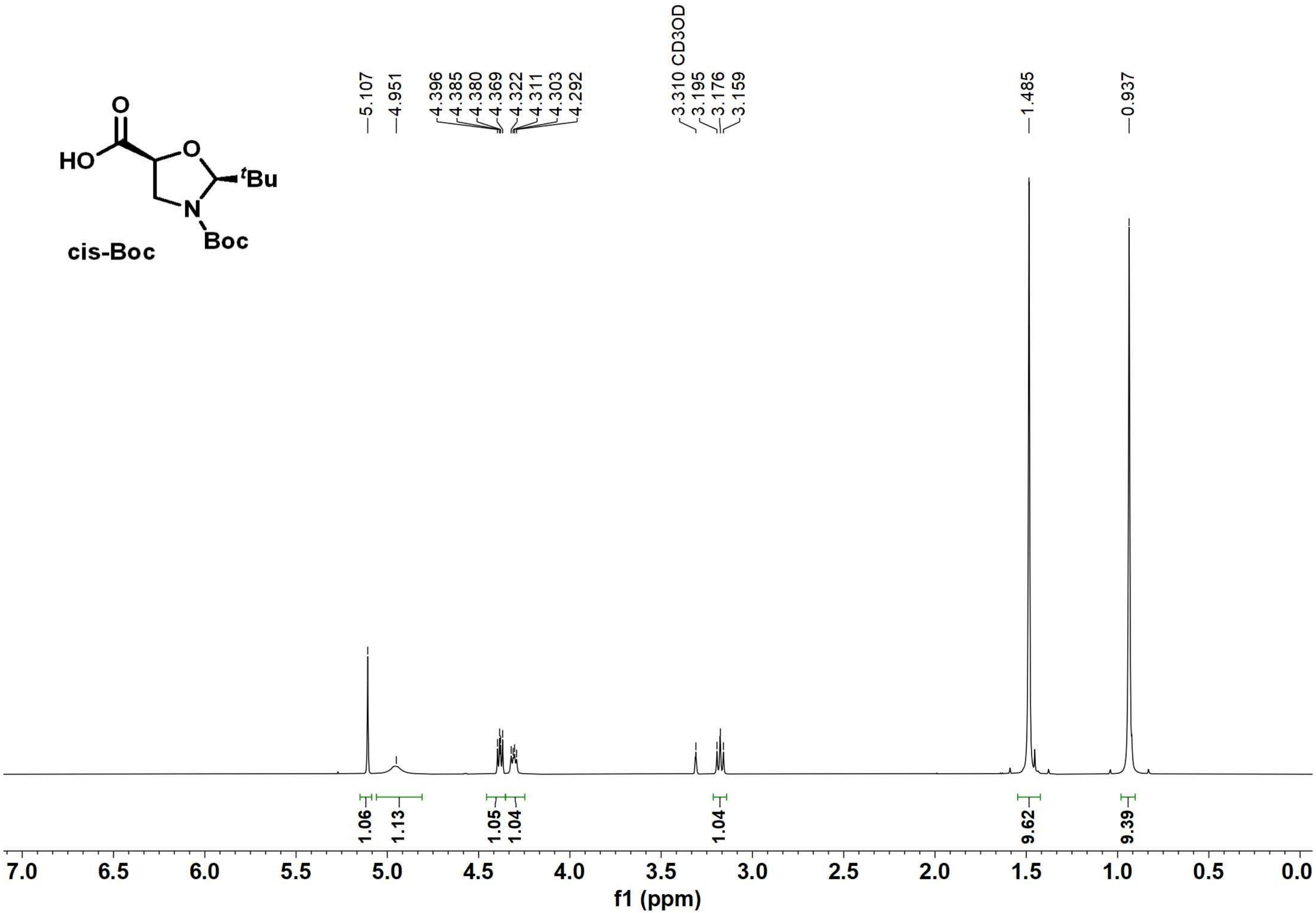

^{1}H NMR of Compound 11 (*cis*-boc-acid) (600 MHz, CD_3OD)

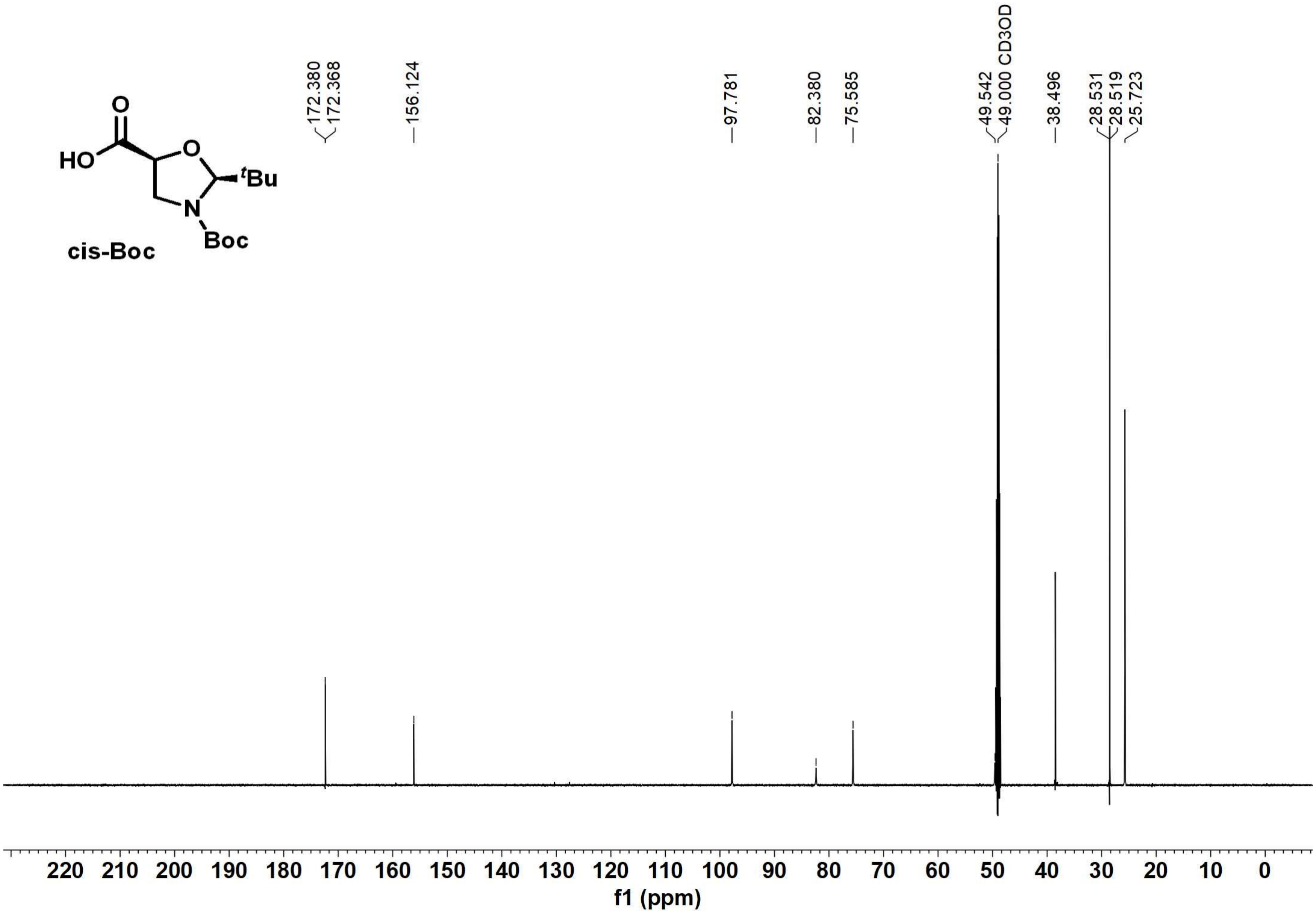

^{13}C NMR of Compound 11 (*cis*-boc-acid) (151 MHz, CD_3OD)

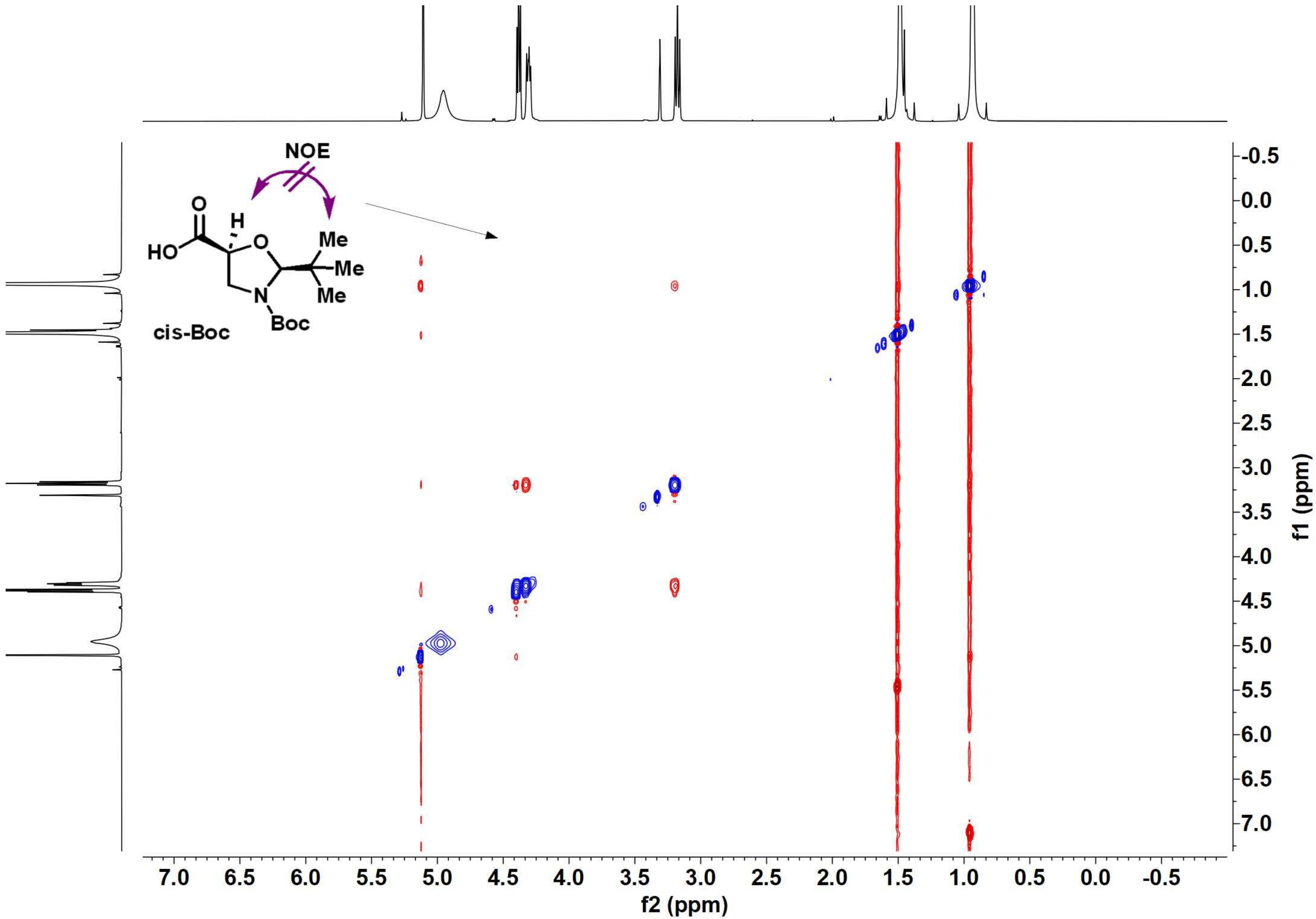

NOESY of Compound 11 (*cis*-boc-acid) (600 MHz, CD_3OD)

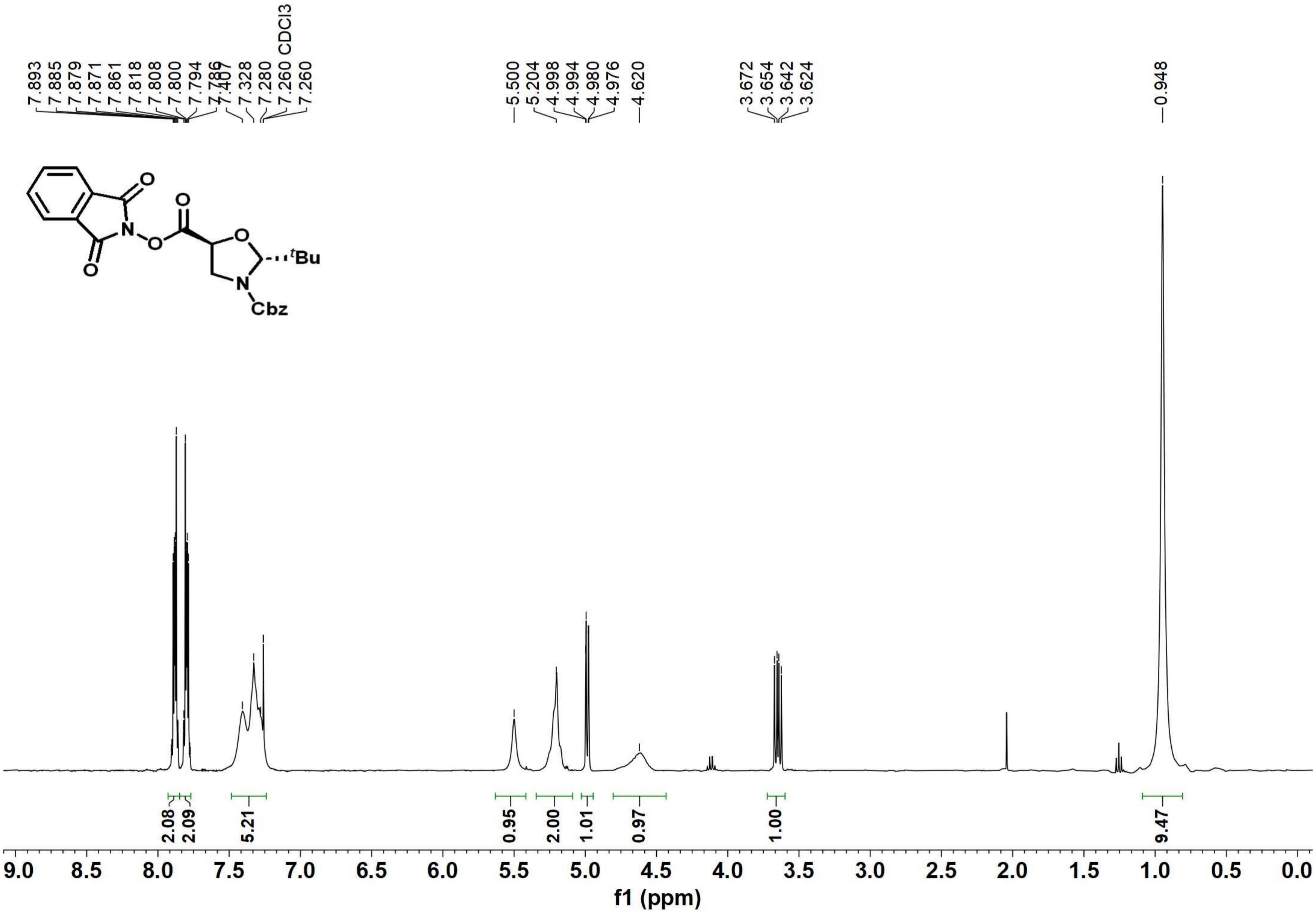

^{1}H NMR of Compound 10 (*trans*) (600 MHz, $CDCl_3$)

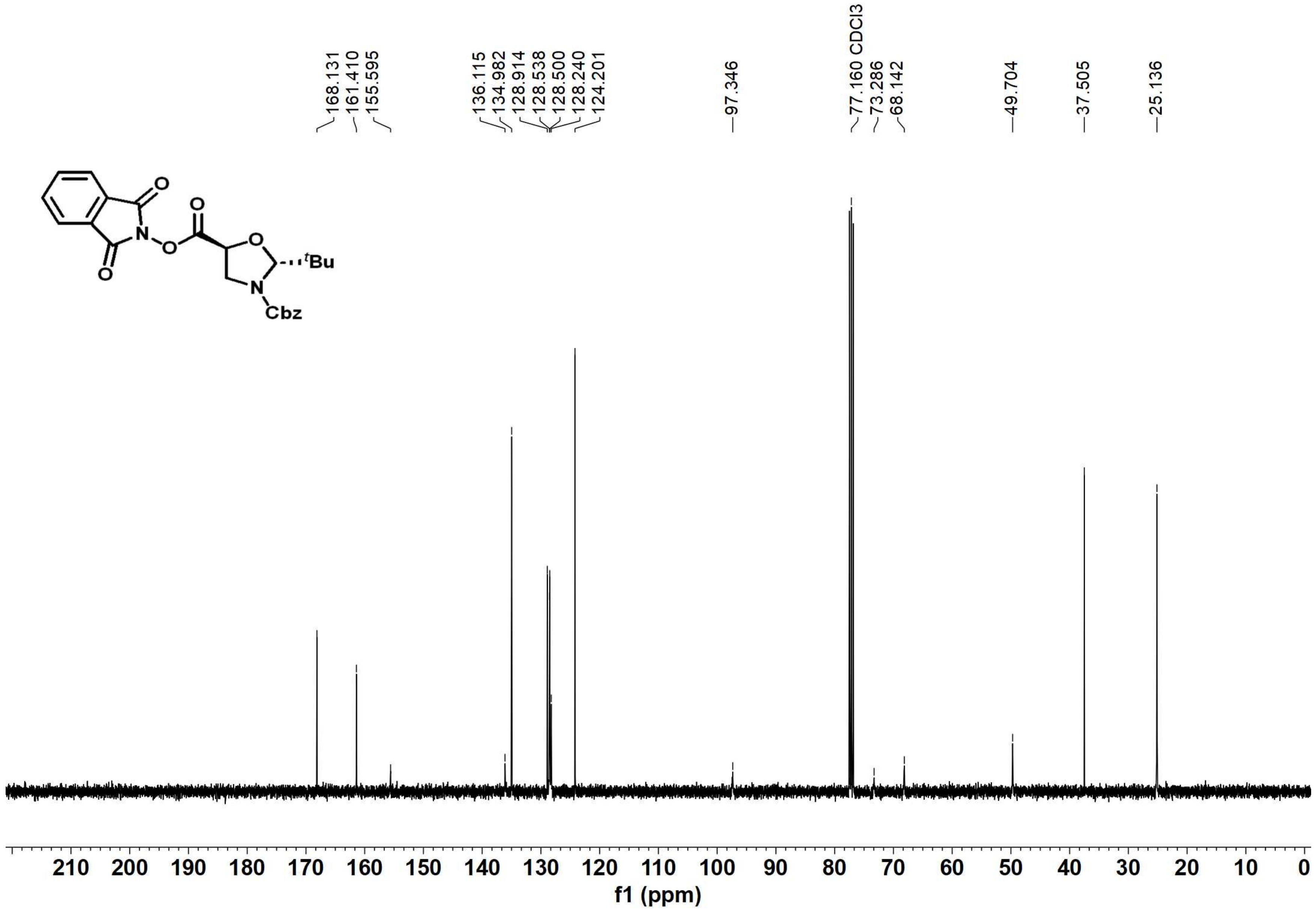

^{13}C NMR of Compound 10 (*trans*) (151 MHz, $CDCl_3$)

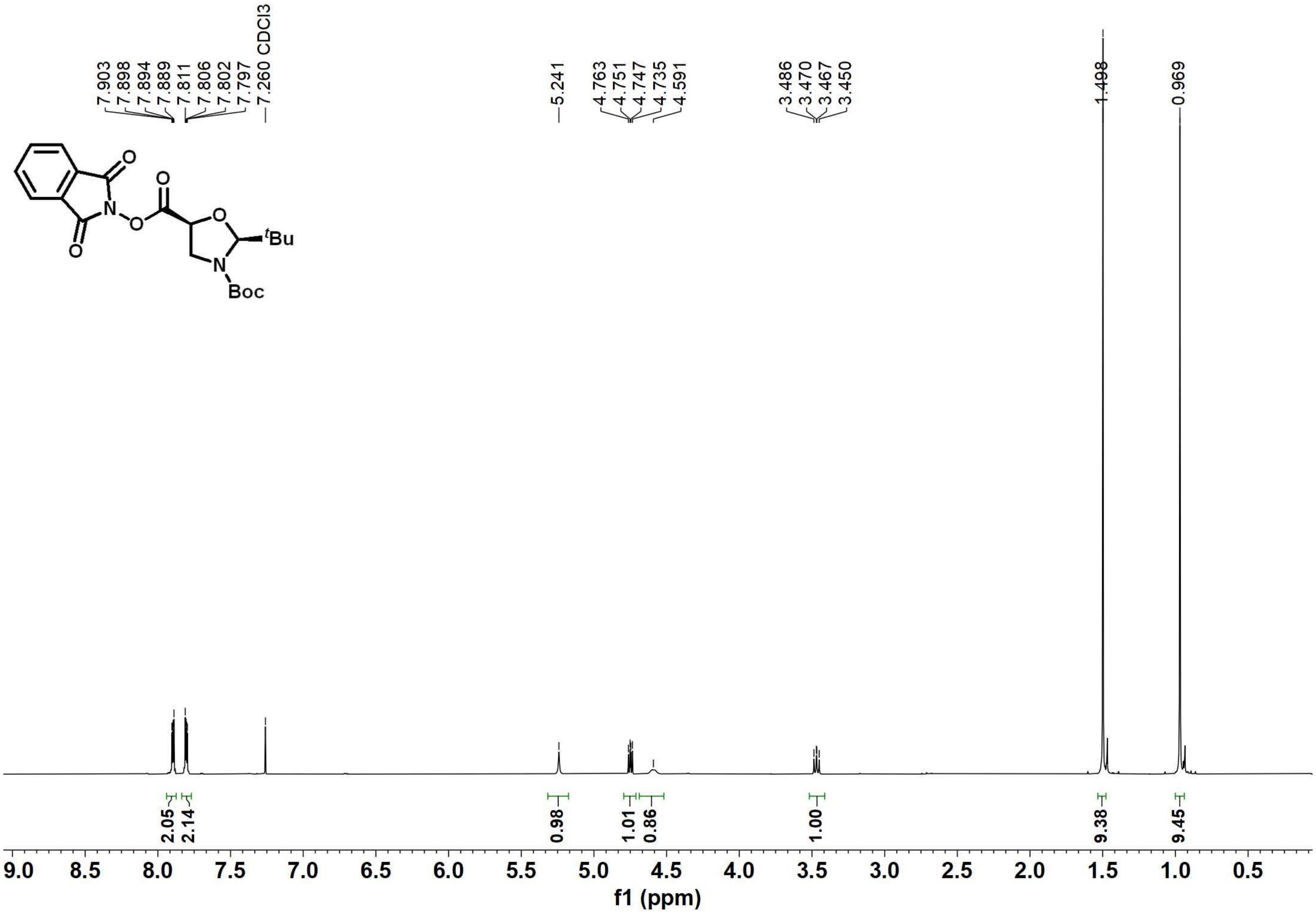

^{1}H NMR of Compound 11 (*cis*) (600 MHz, CDCl$_3$)

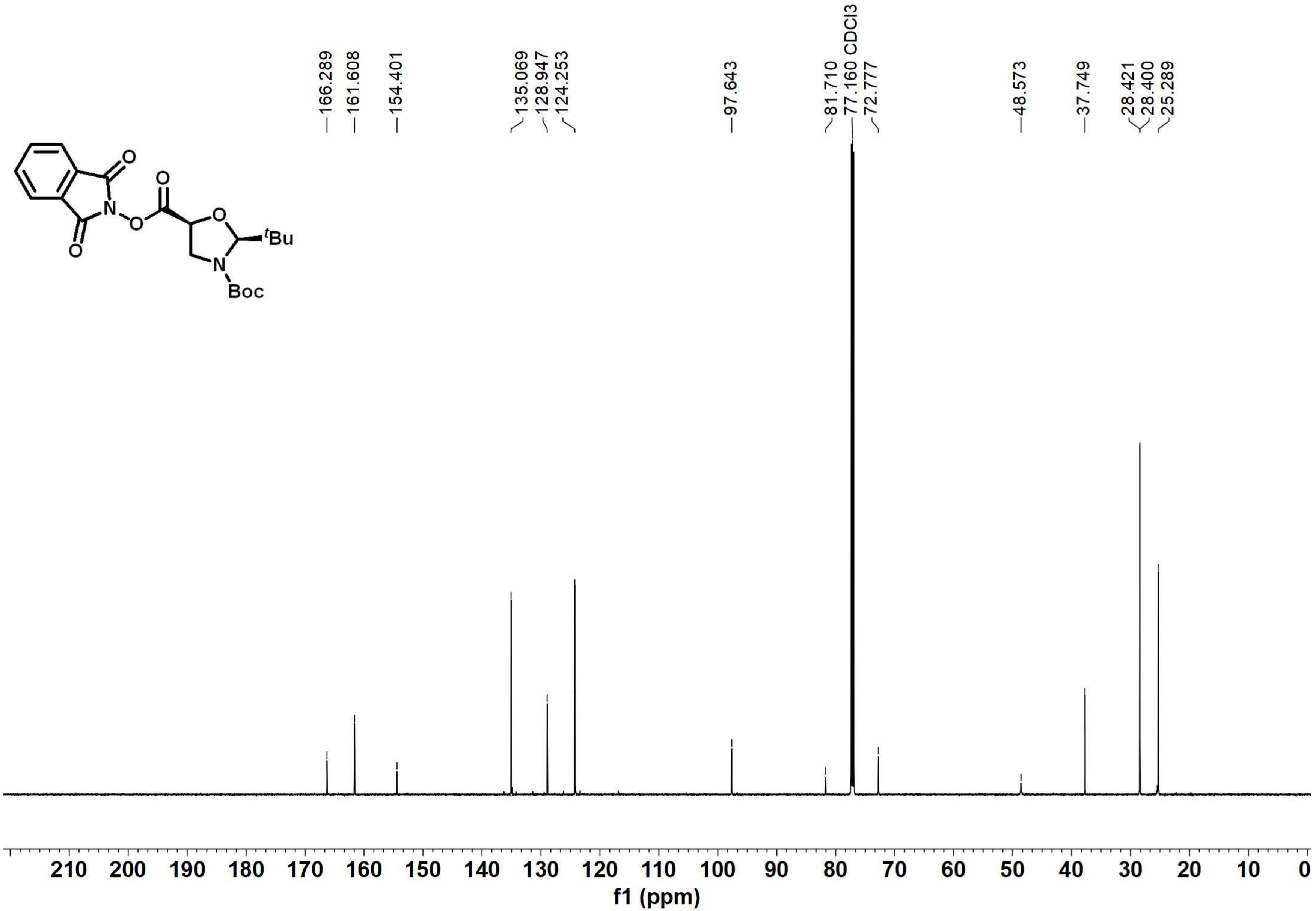

^{13}C NMR of Compound 11 (*cis*) (151 MHz, $CDCl_3$)

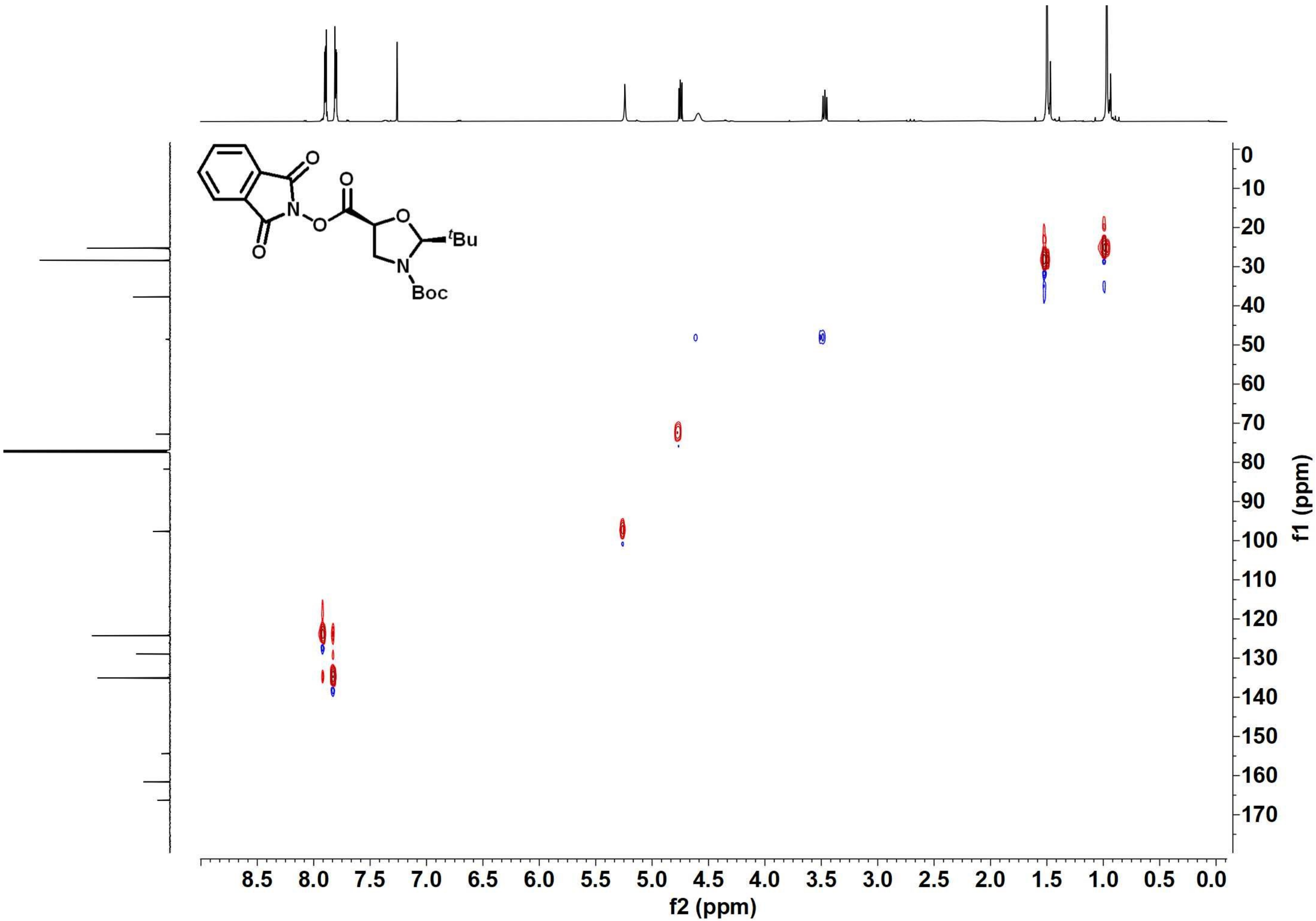

HSQC of Compound 11 (*cis*) ($CDCl_3$)

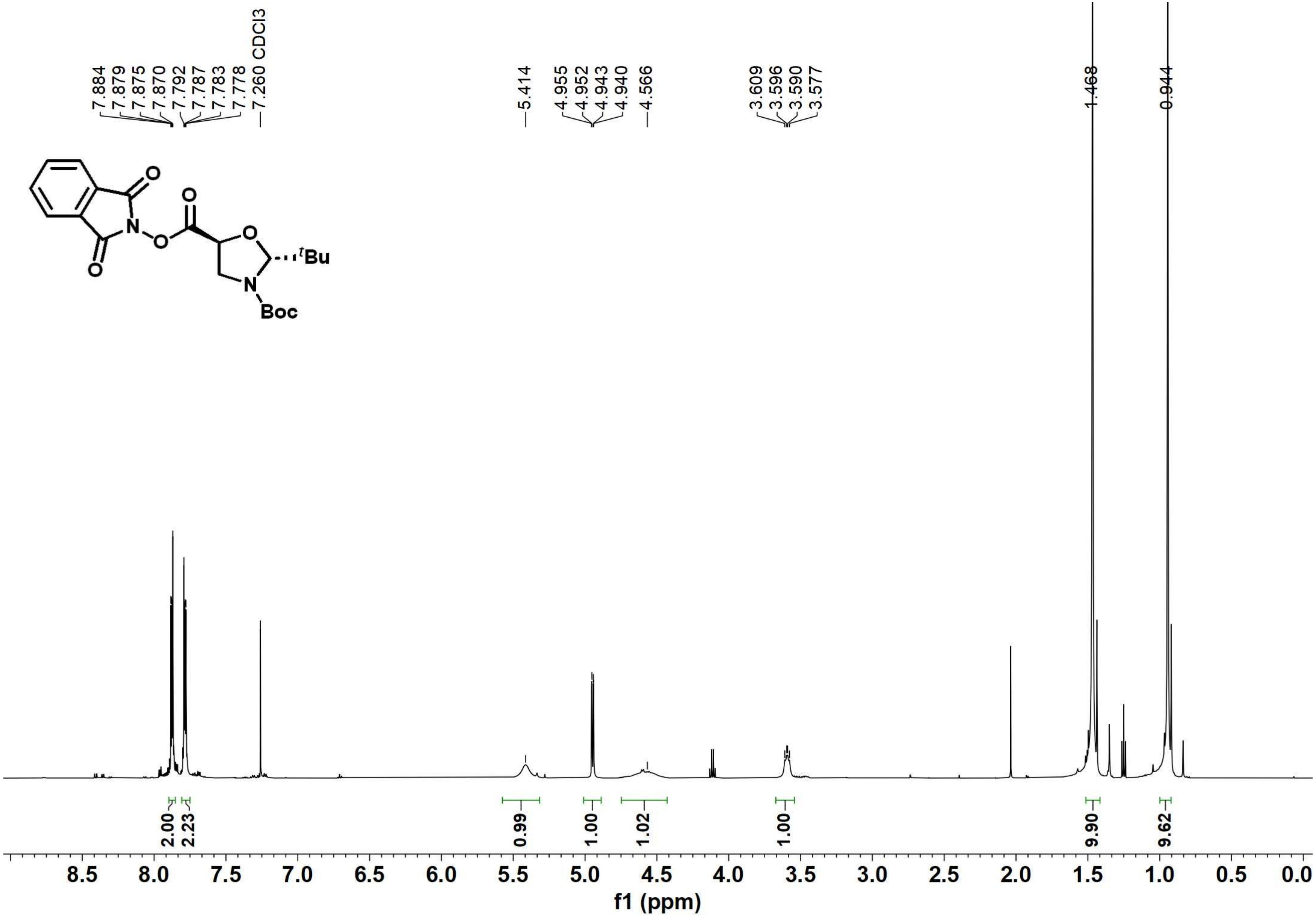

^{1}H NMR of Compound 11 (*trans*) (600 MHz, $CDCl_3$)

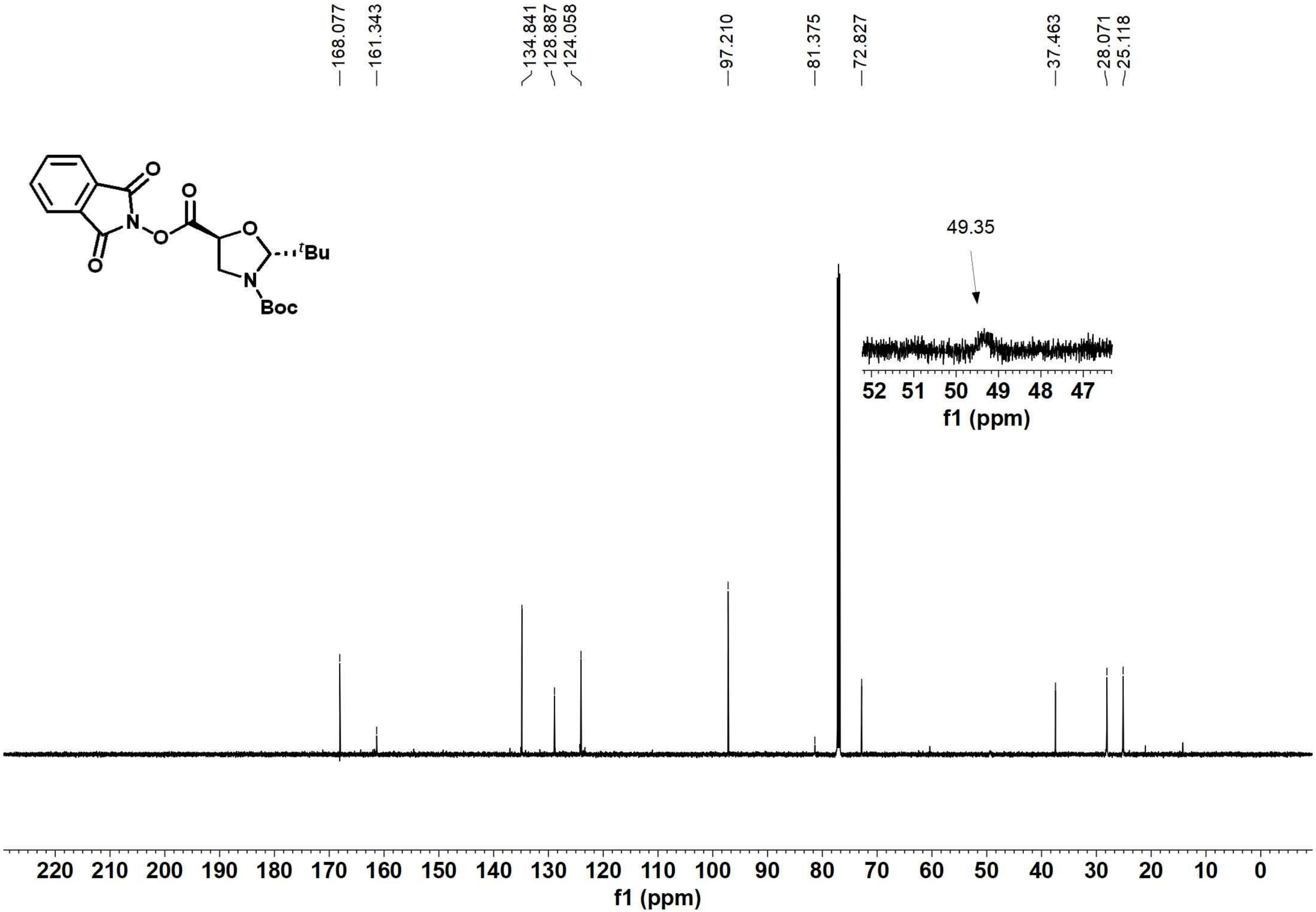

^{13}C NMR of Compound 11 (*trans*) (151 MHz, $CDCl_3$)

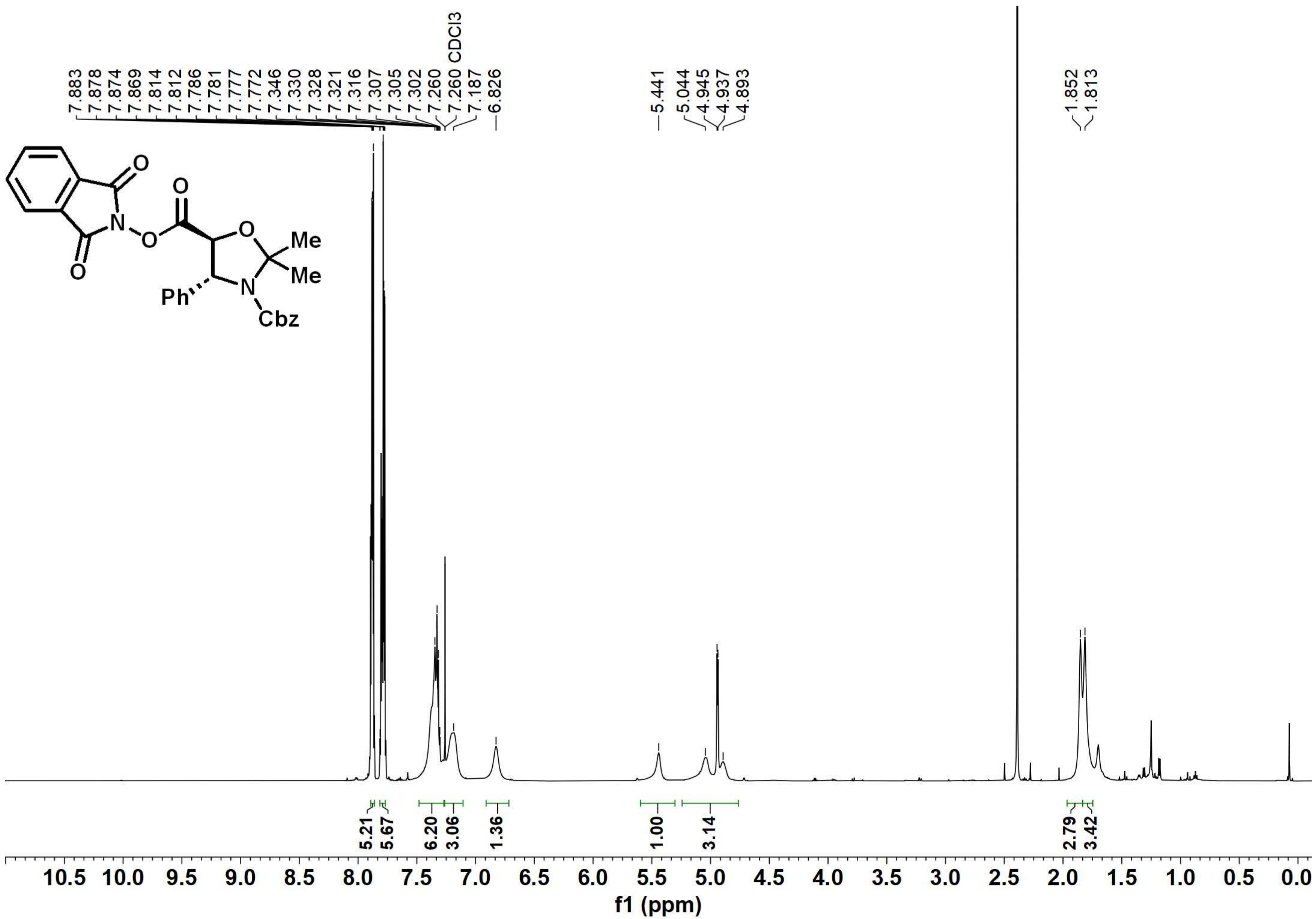

^{1}H NMR of Compound S-6 (600 MHz, $CDCl_3$)

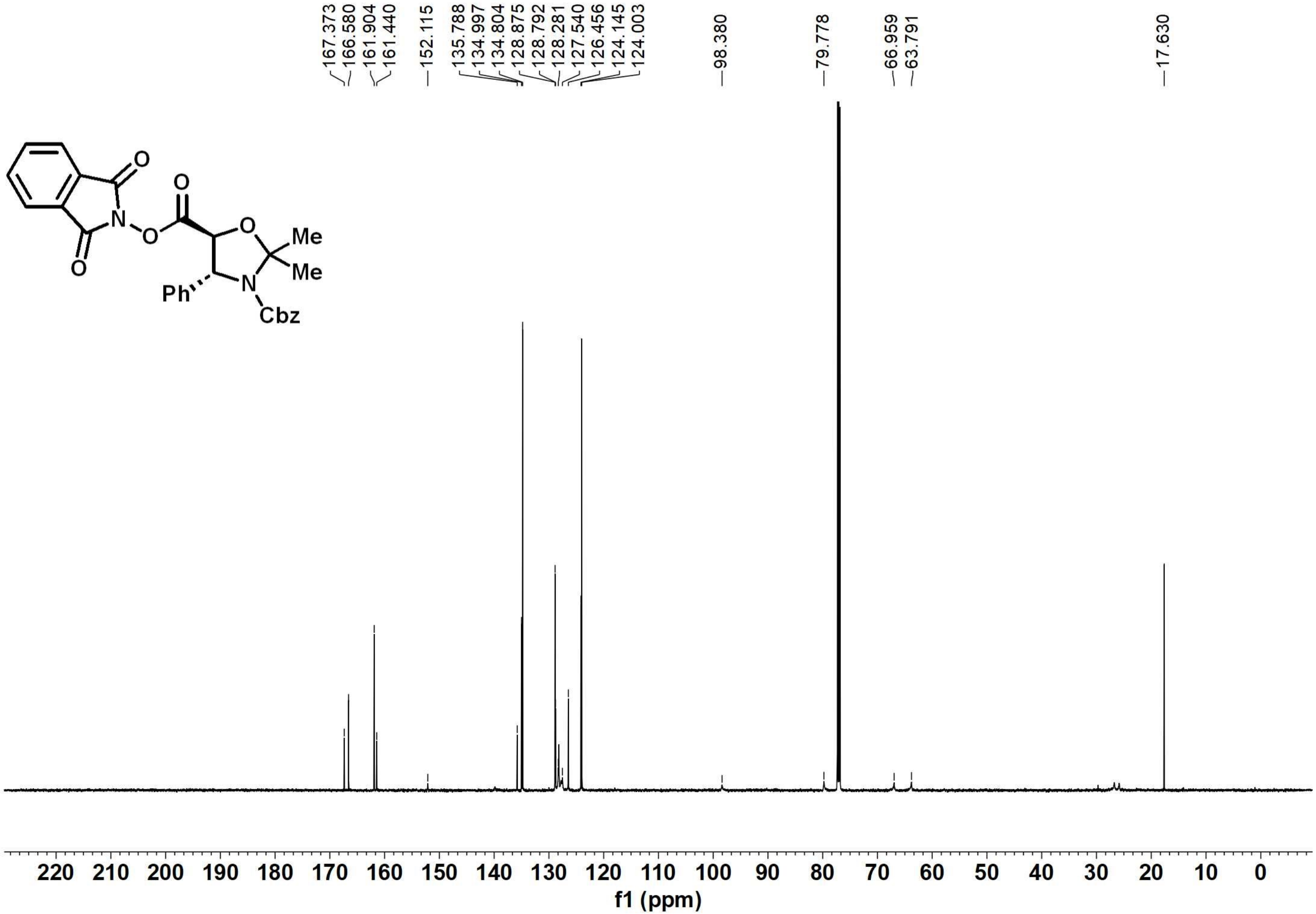

^{13}C NMR of Compound S-6 (151 MHz, $CDCl_3$)

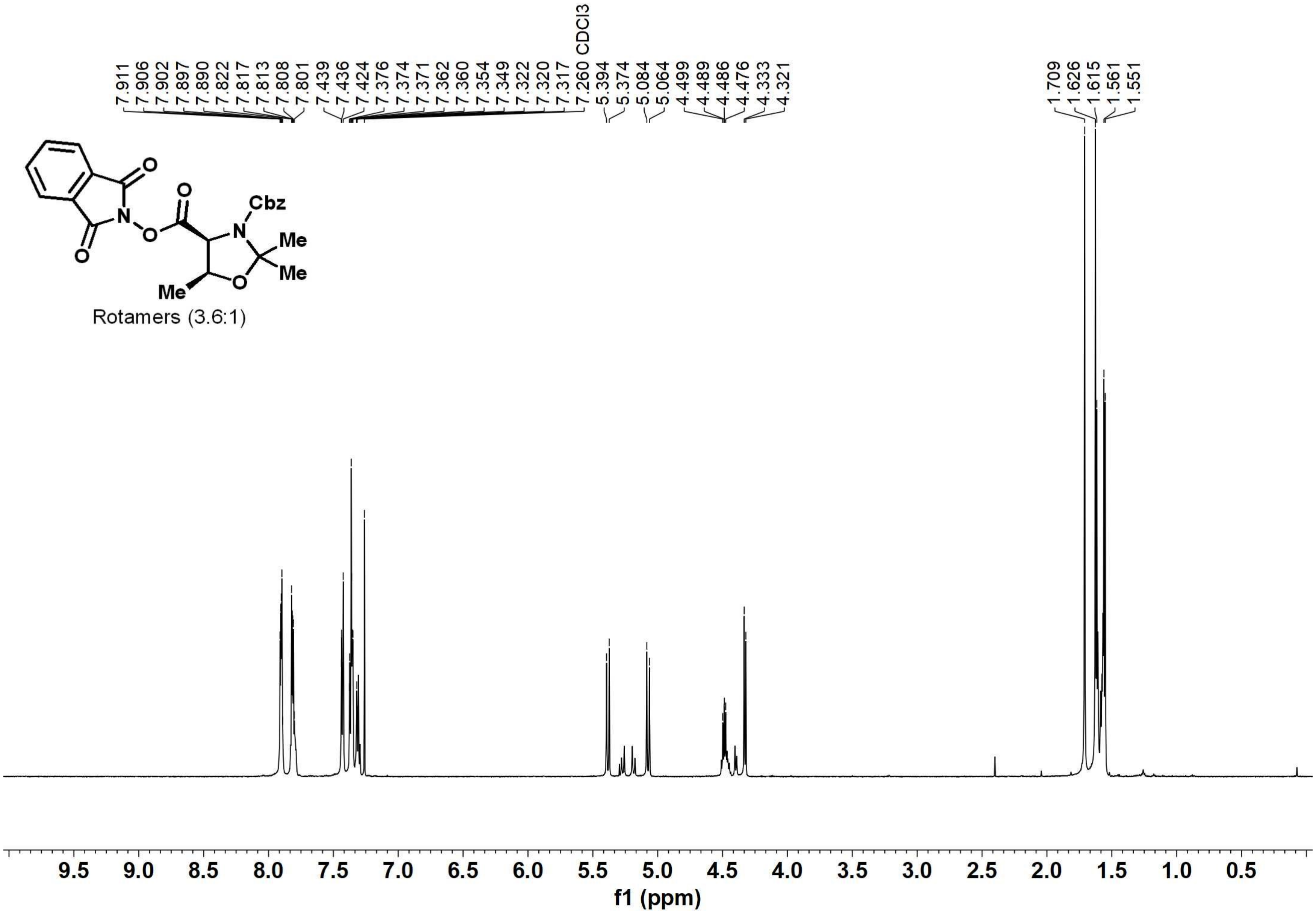

^{1}H NMR of Compound S-8 (600 MHz, $CDCl_3$)

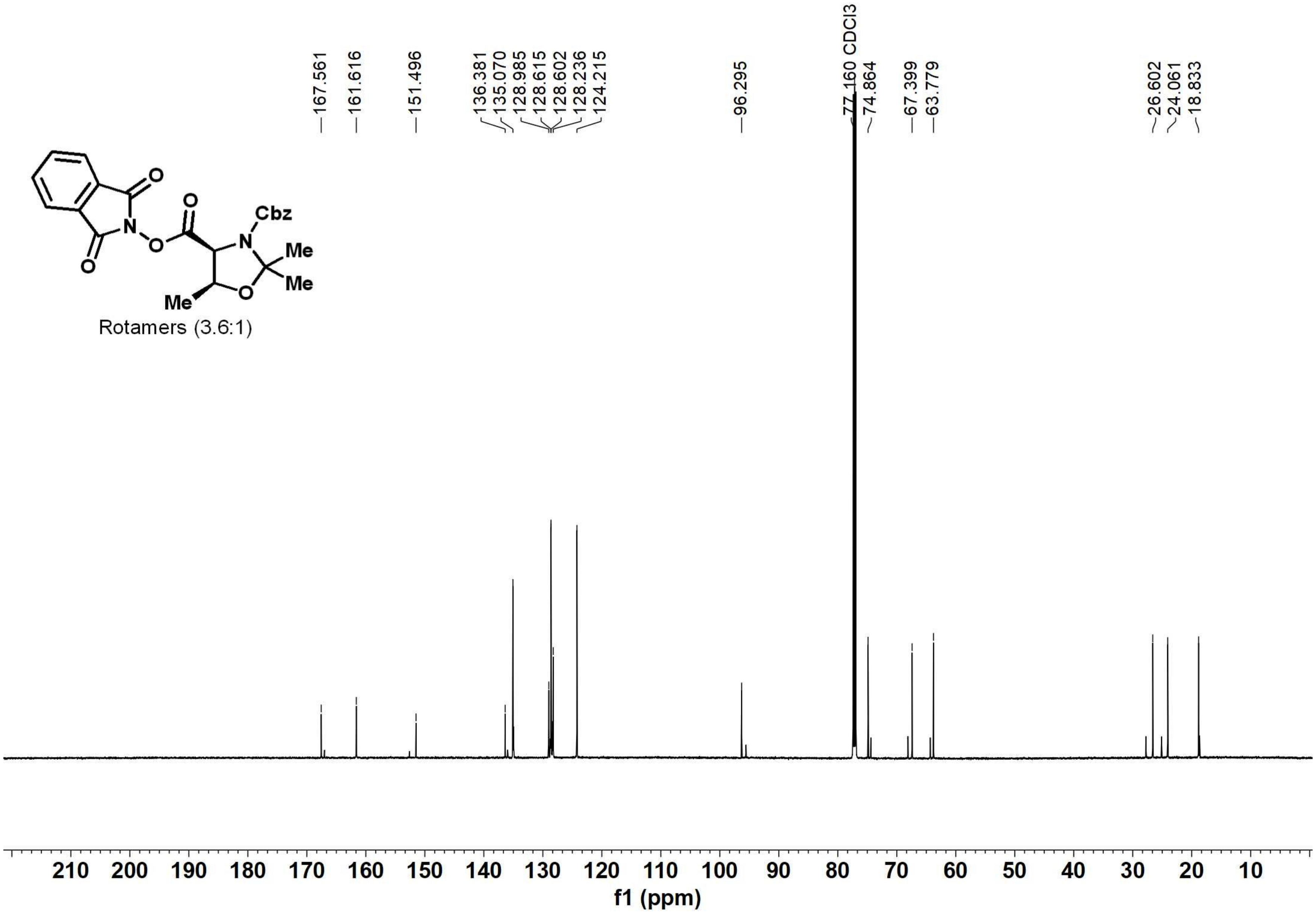

^{13}C NMR of Compound S-8 (151 MHz, $CDCl_3$)

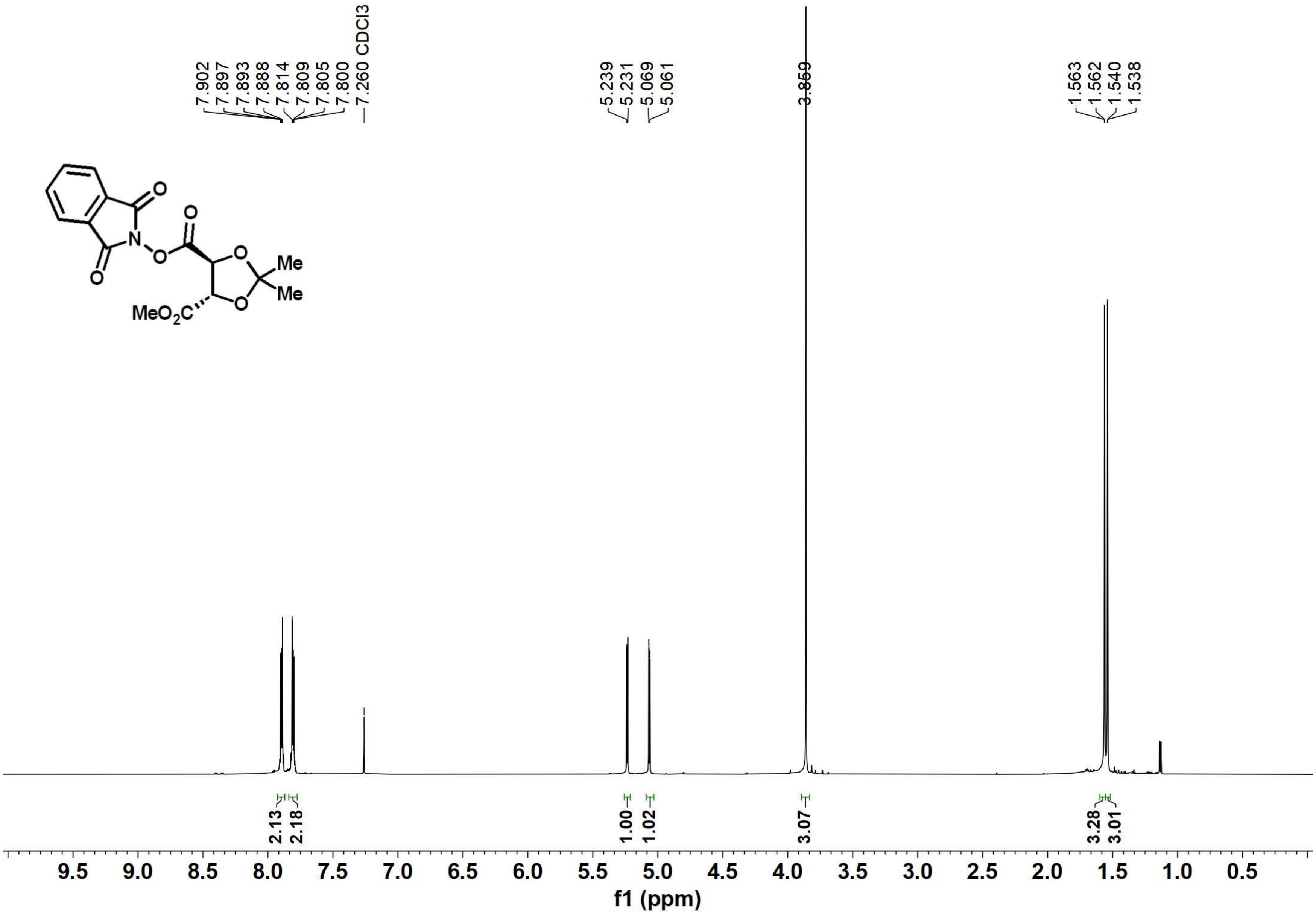

^{1}H NMR of Compound S-9 (600 MHz, $CDCl_3$)

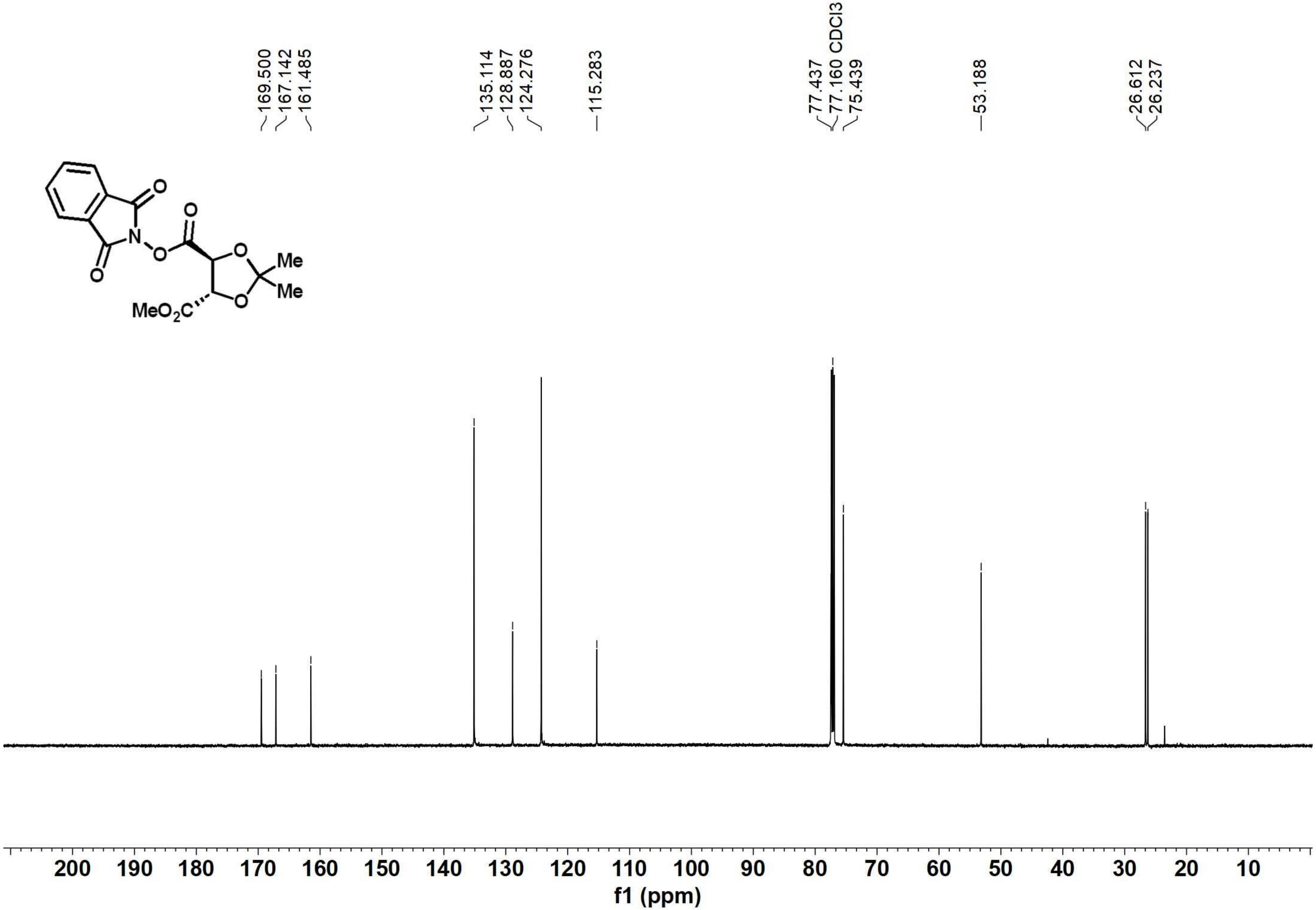

^{13}C NMR of Compound S-9 (151 MHz, $CDCl_3$)

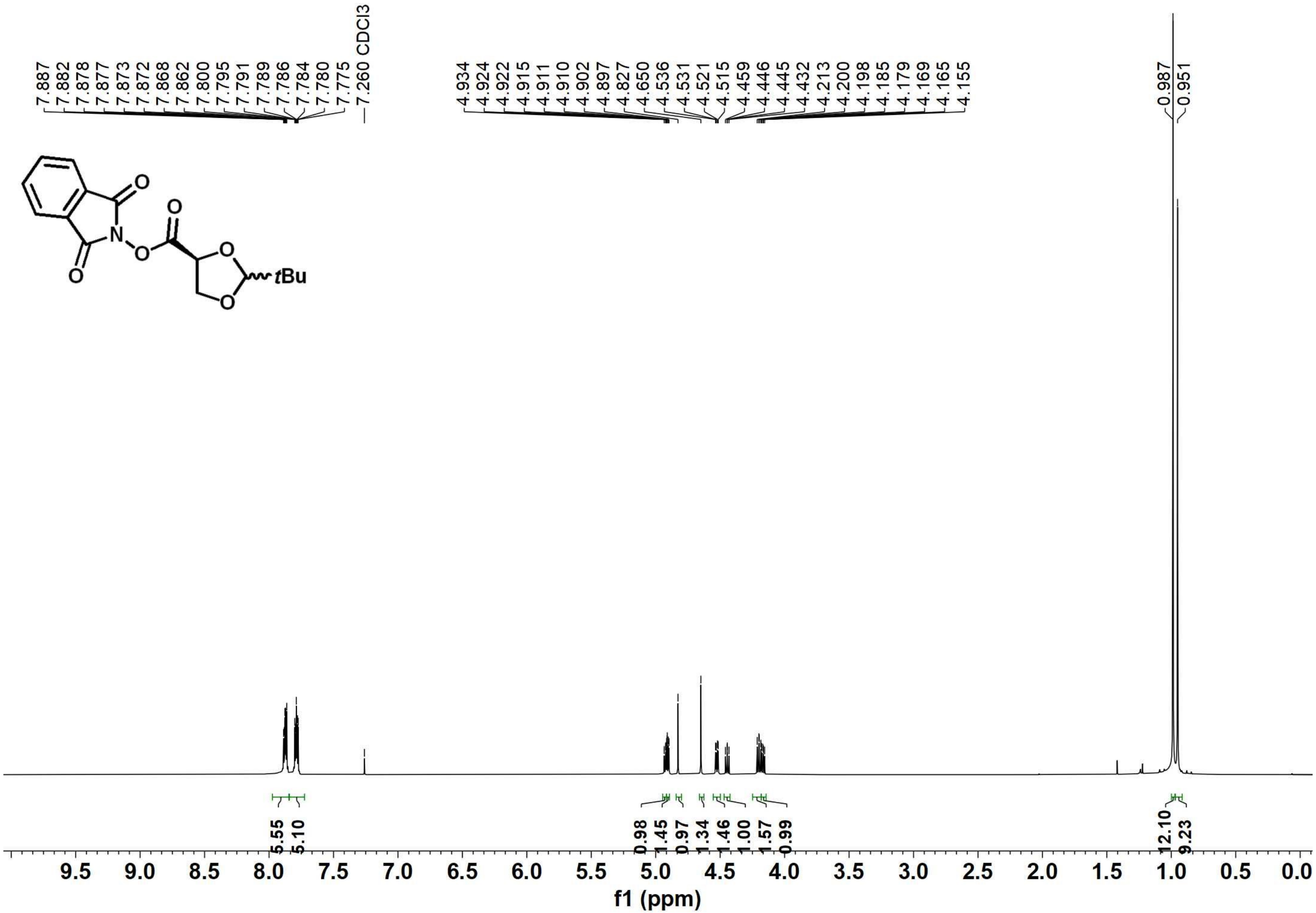

^{1}H NMR of Compound 22 (600 MHz, $CDCl_3$)

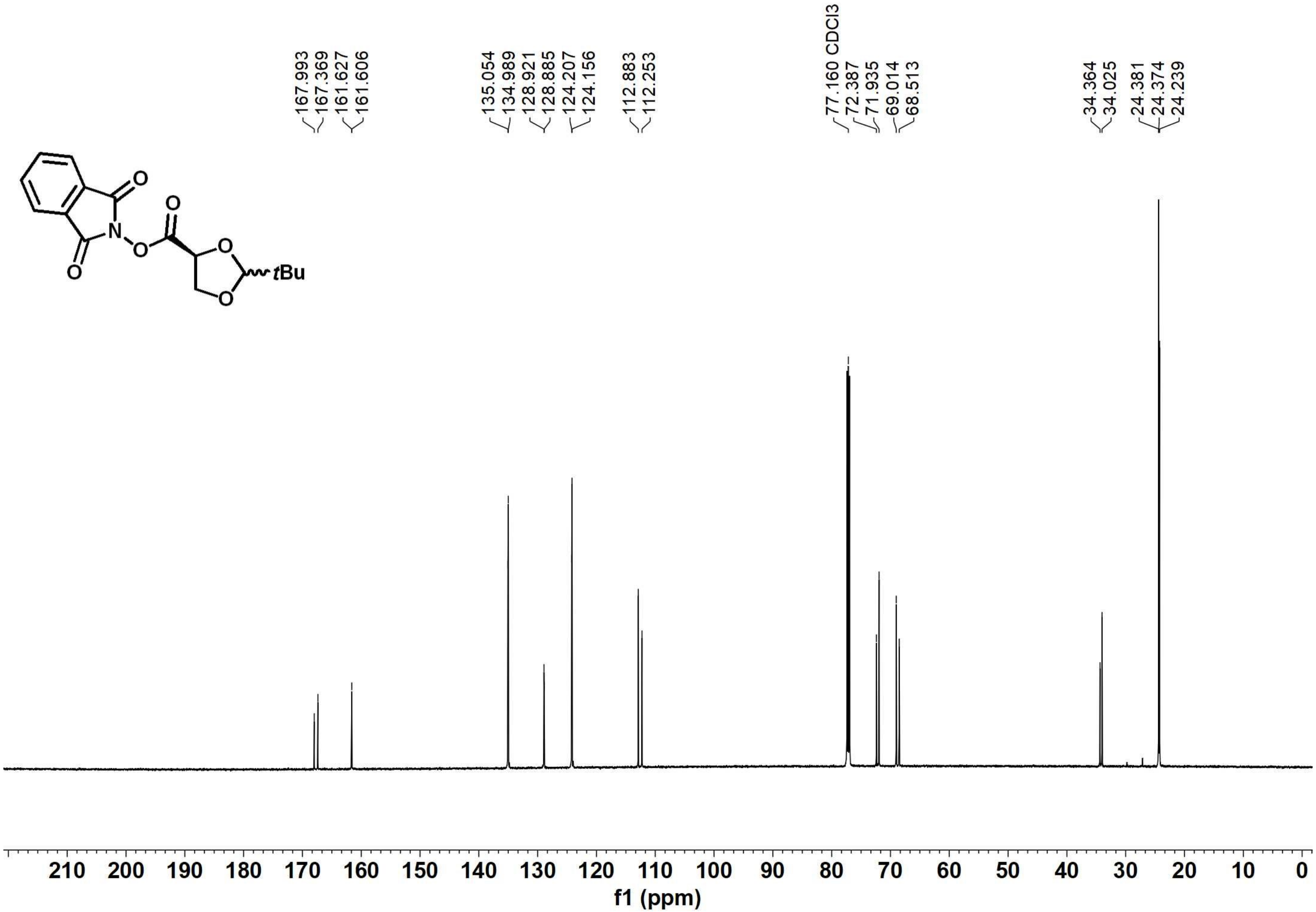

^{13}C NMR of Compound 22 (151 MHz, $CDCl_3$)

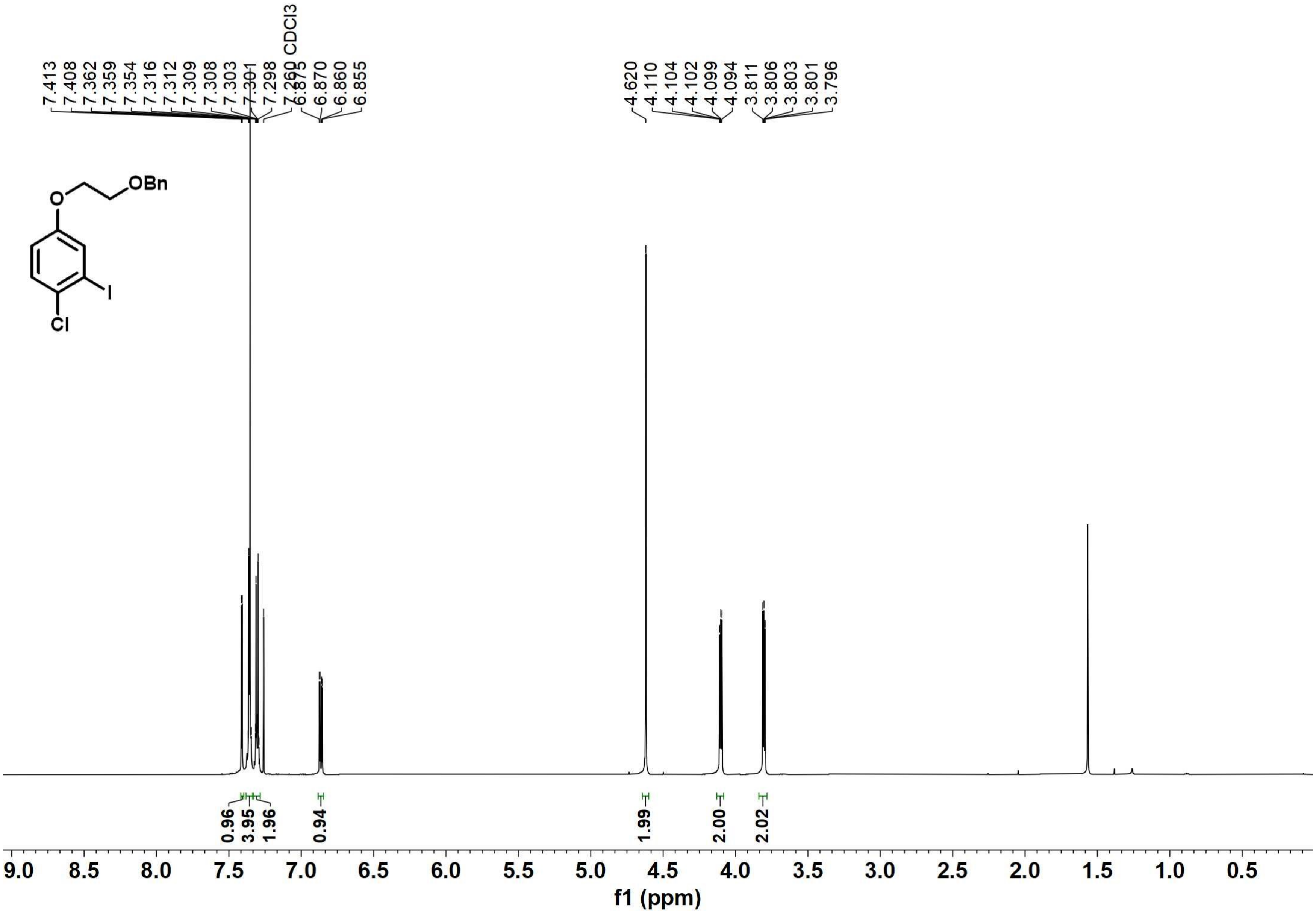

^{1}H NMR of Compound 12 (600 MHz, $CDCl_3$)

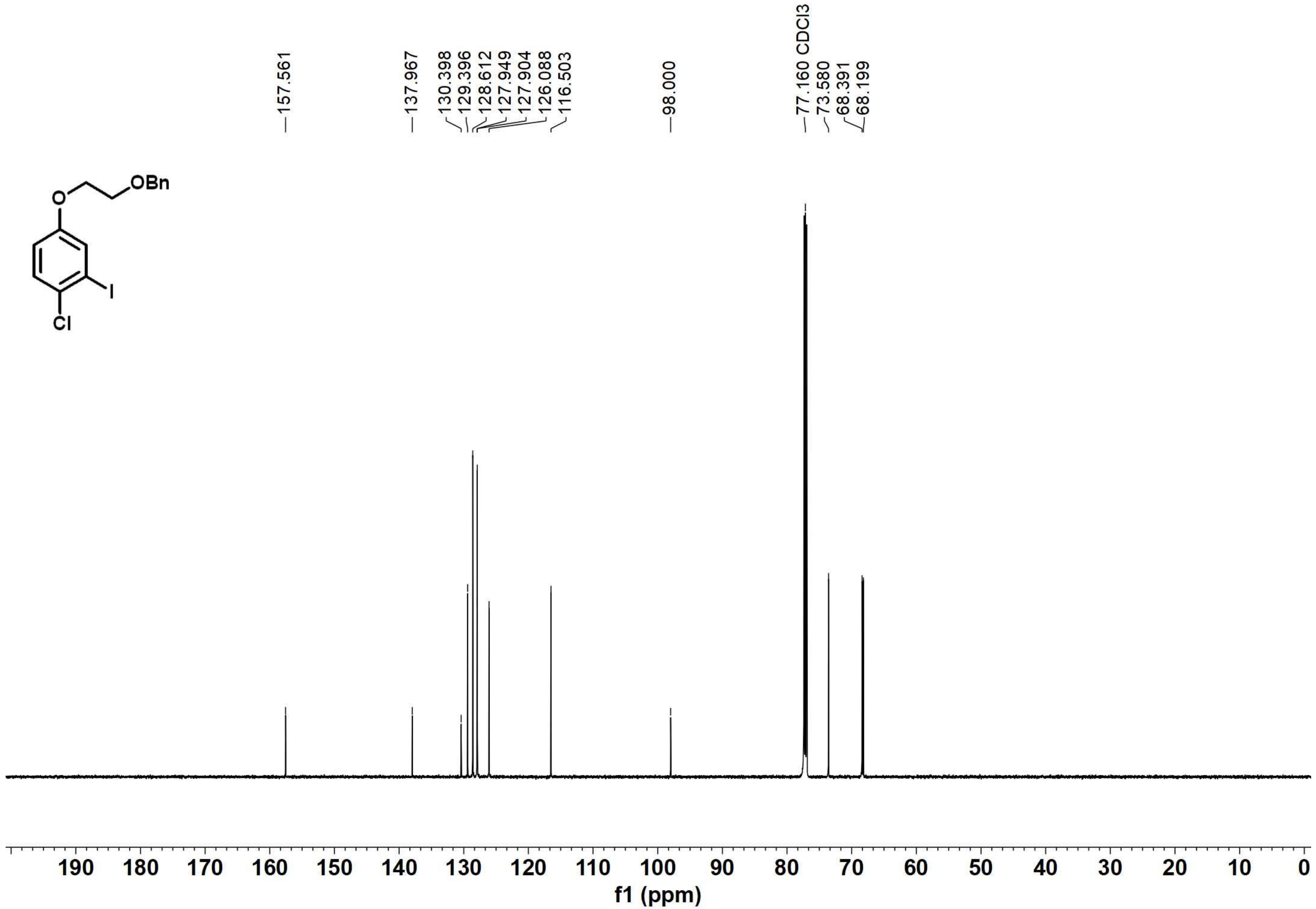

^{13}C NMR of Compound 12 (151 MHz, $CDCl_3$)

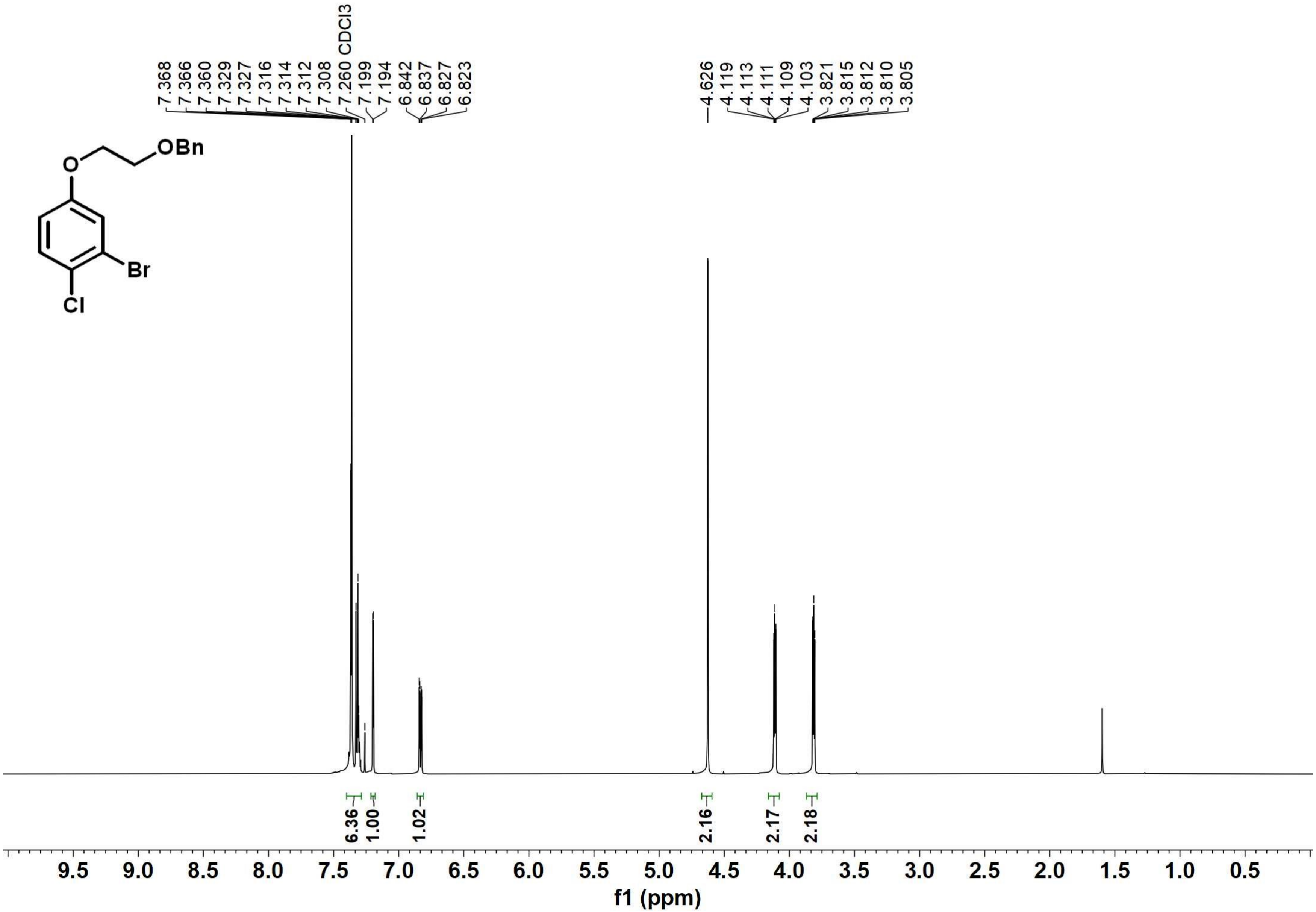

^{1}H NMR of Compound 12-Br (600 MHz, $CDCl_3$)

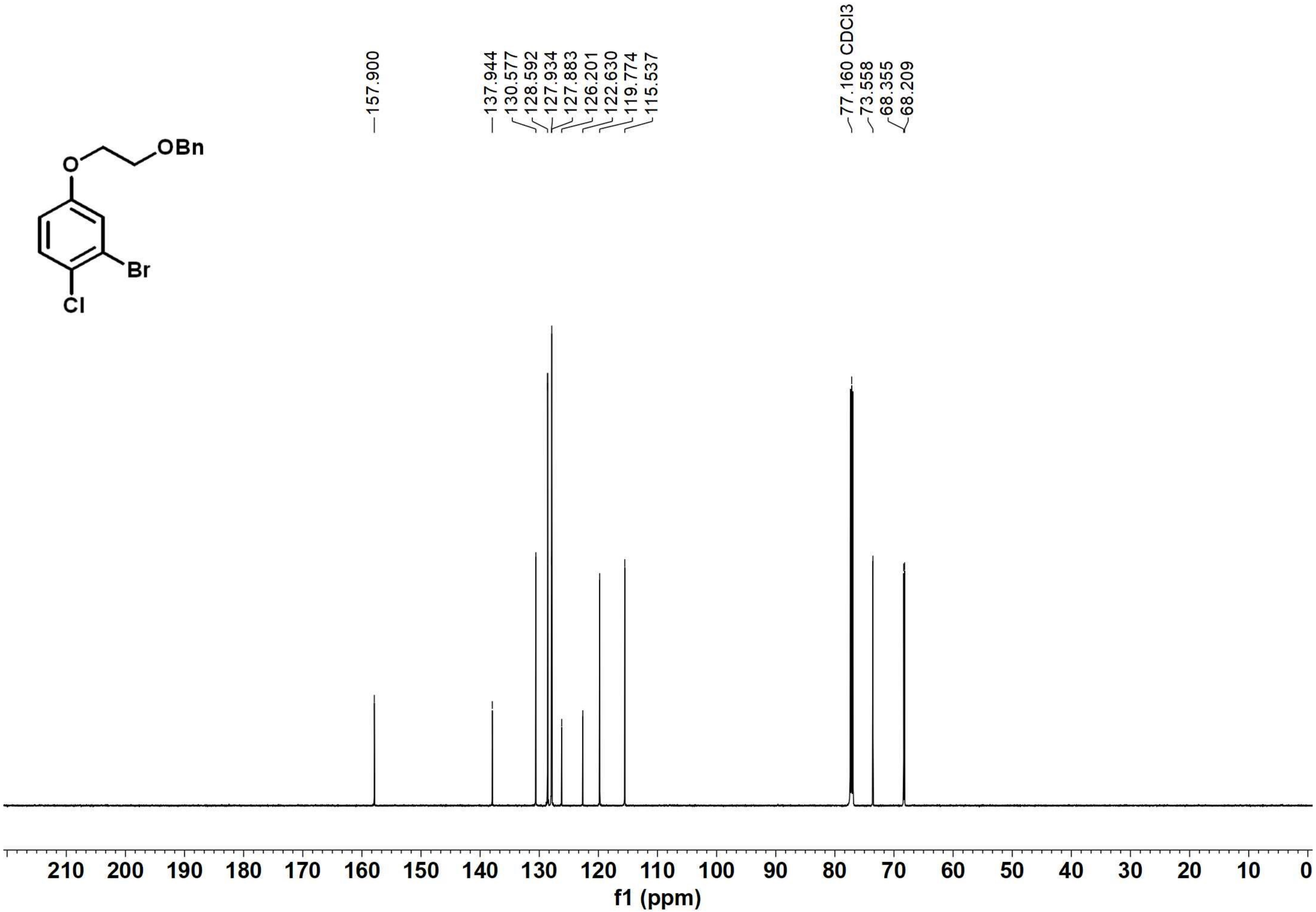

^{13}C NMR of Compound 12-Br (151 MHz, $CDCl_3$)

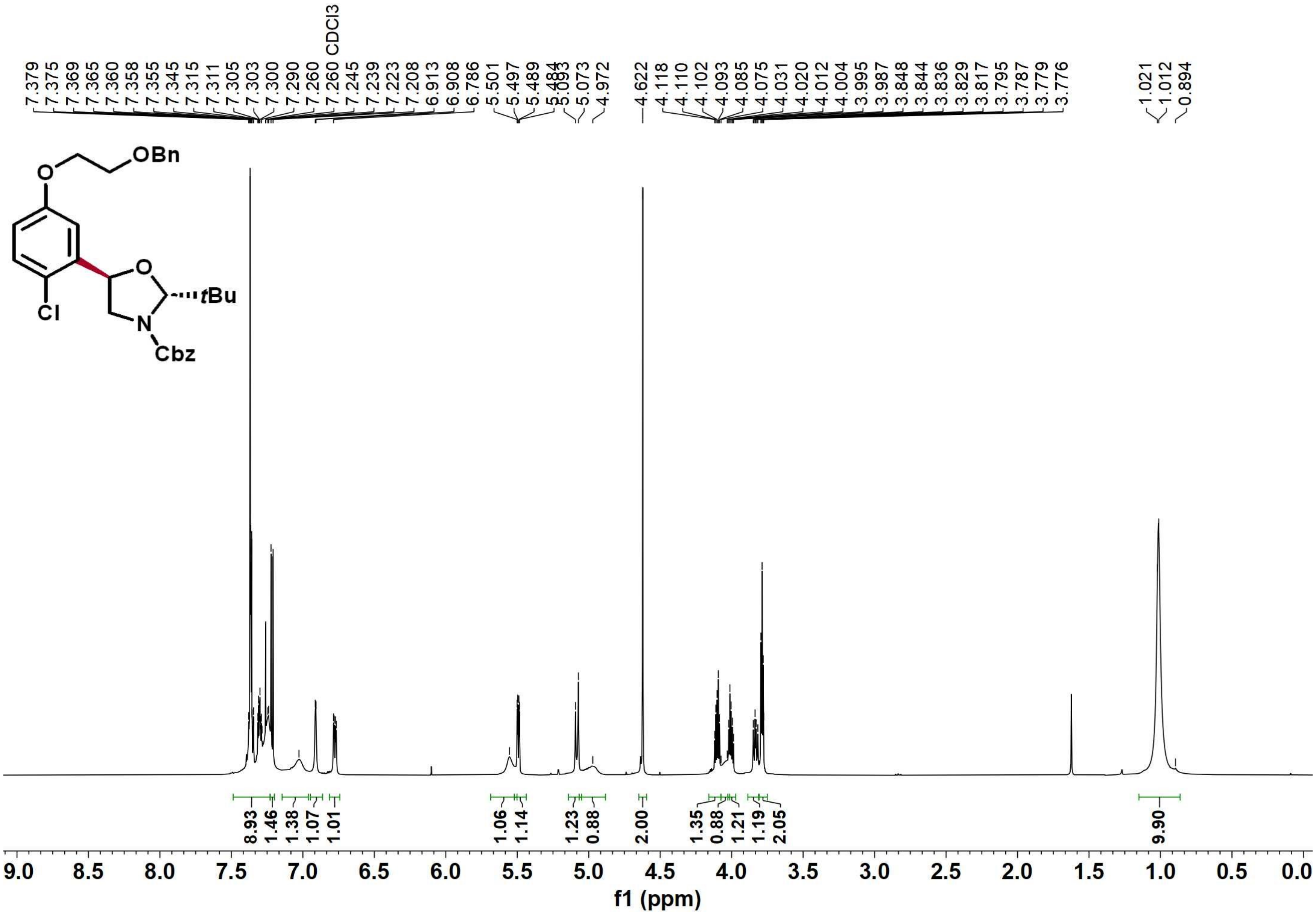

^{1}H NMR of Compound 13 (*trans*) (600 MHz, $CDCl_3$)

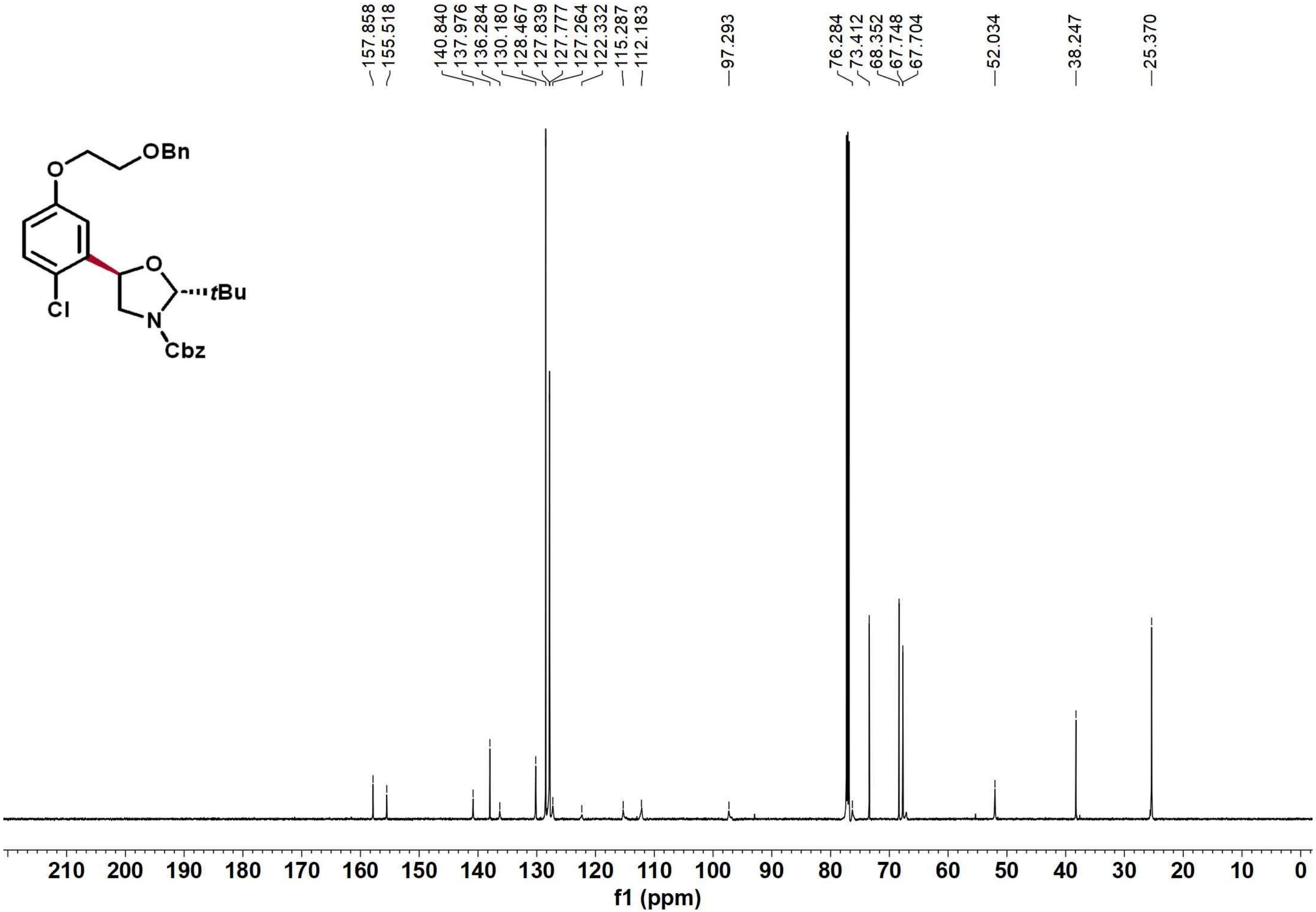

^{13}C NMR of Compound 13 (*trans*) (151 MHz, $CDCl_3$)

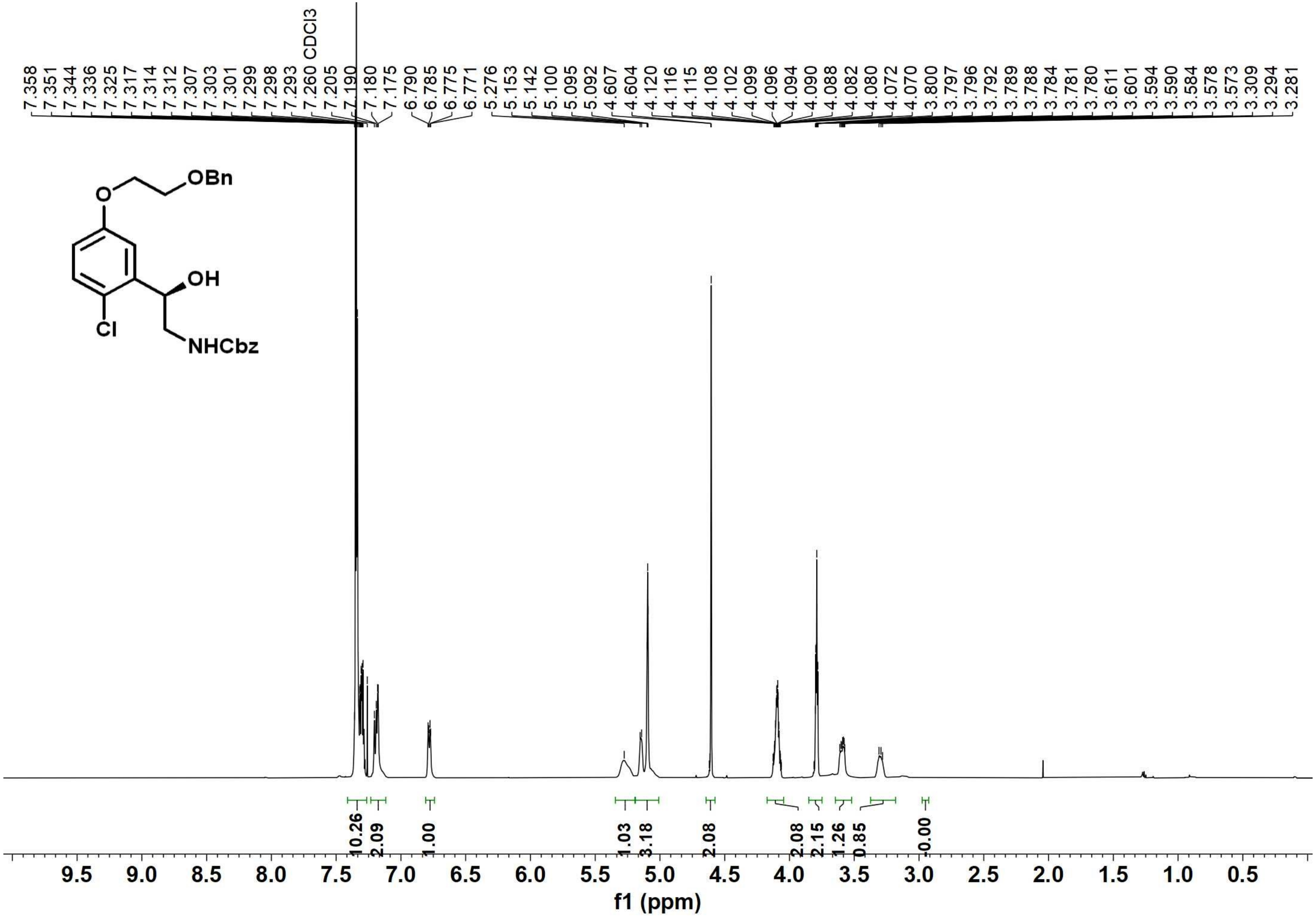

^{1}H NMR of Compound S-10 (600 MHz, $CDCl_3$)

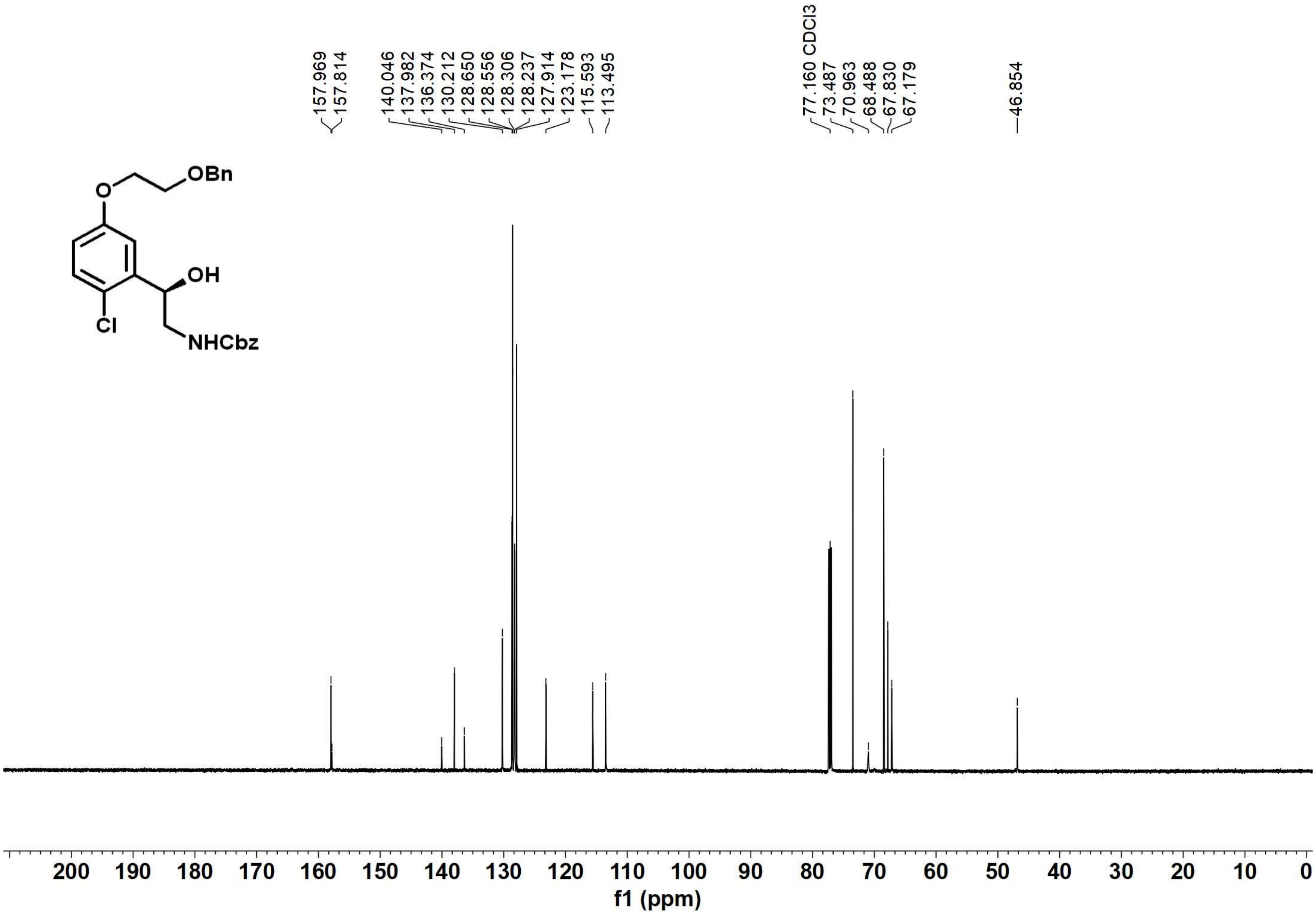

^{13}C NMR of Compound S-10 (151 MHz, $CDCl_3$)

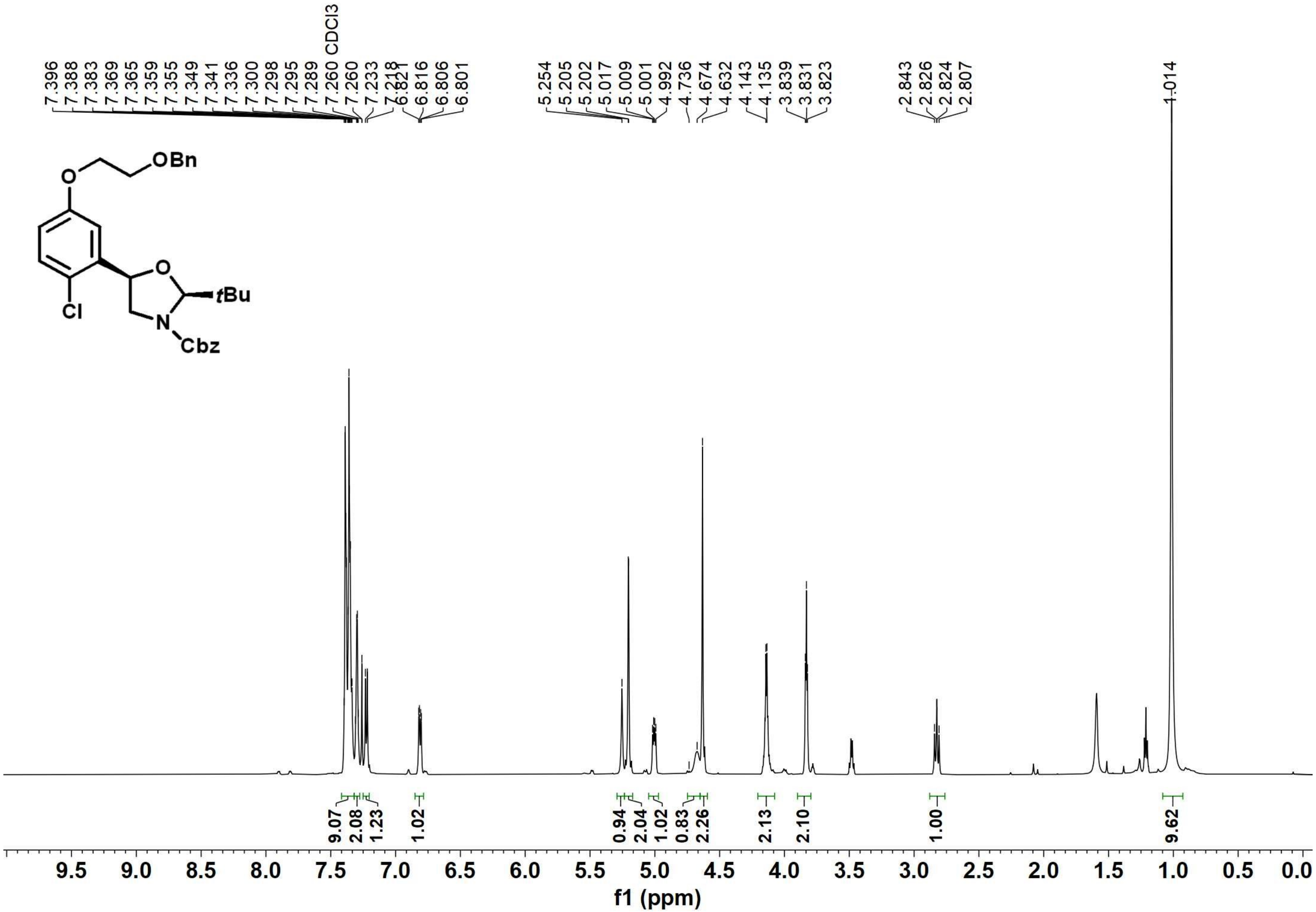

^{1}H NMR of Compound 13 (*cis*) (600 MHz, $CDCl_3$)

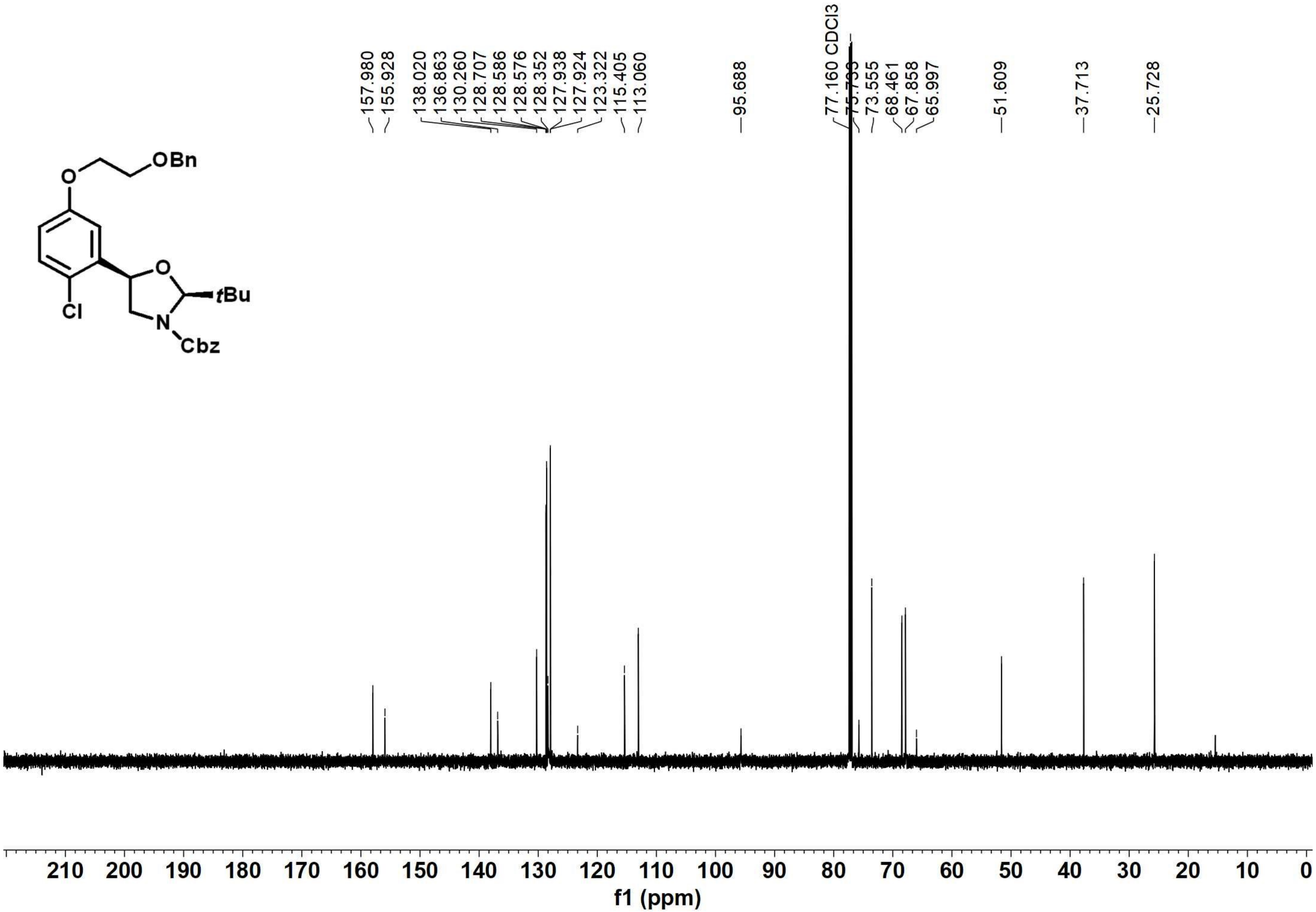

^{13}C NMR of Compound 13 (*cis*) (151 MHz, $CDCl_3$)

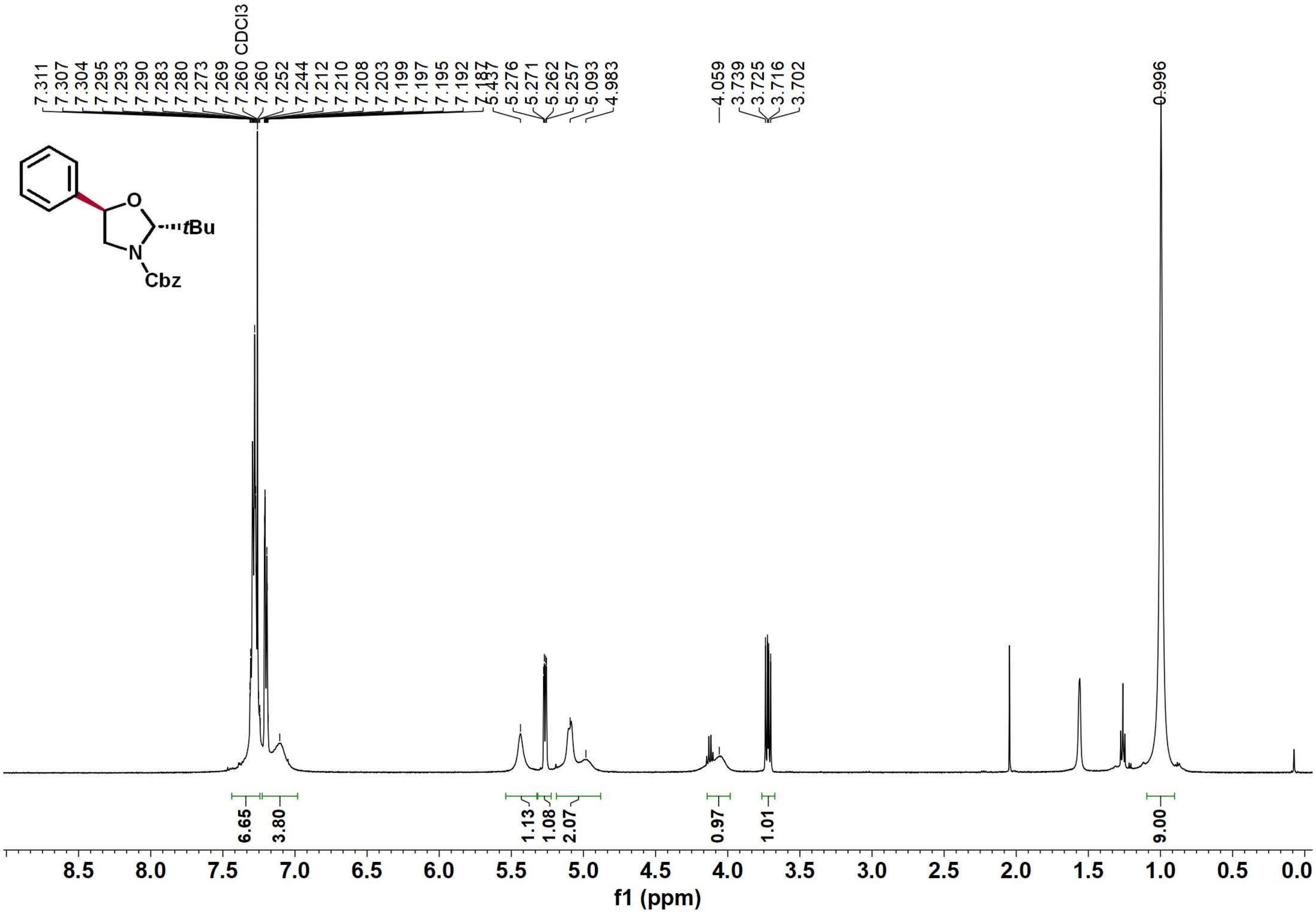

^{1}H NMR of Compound 17a (600 MHz, $CDCl_3$)

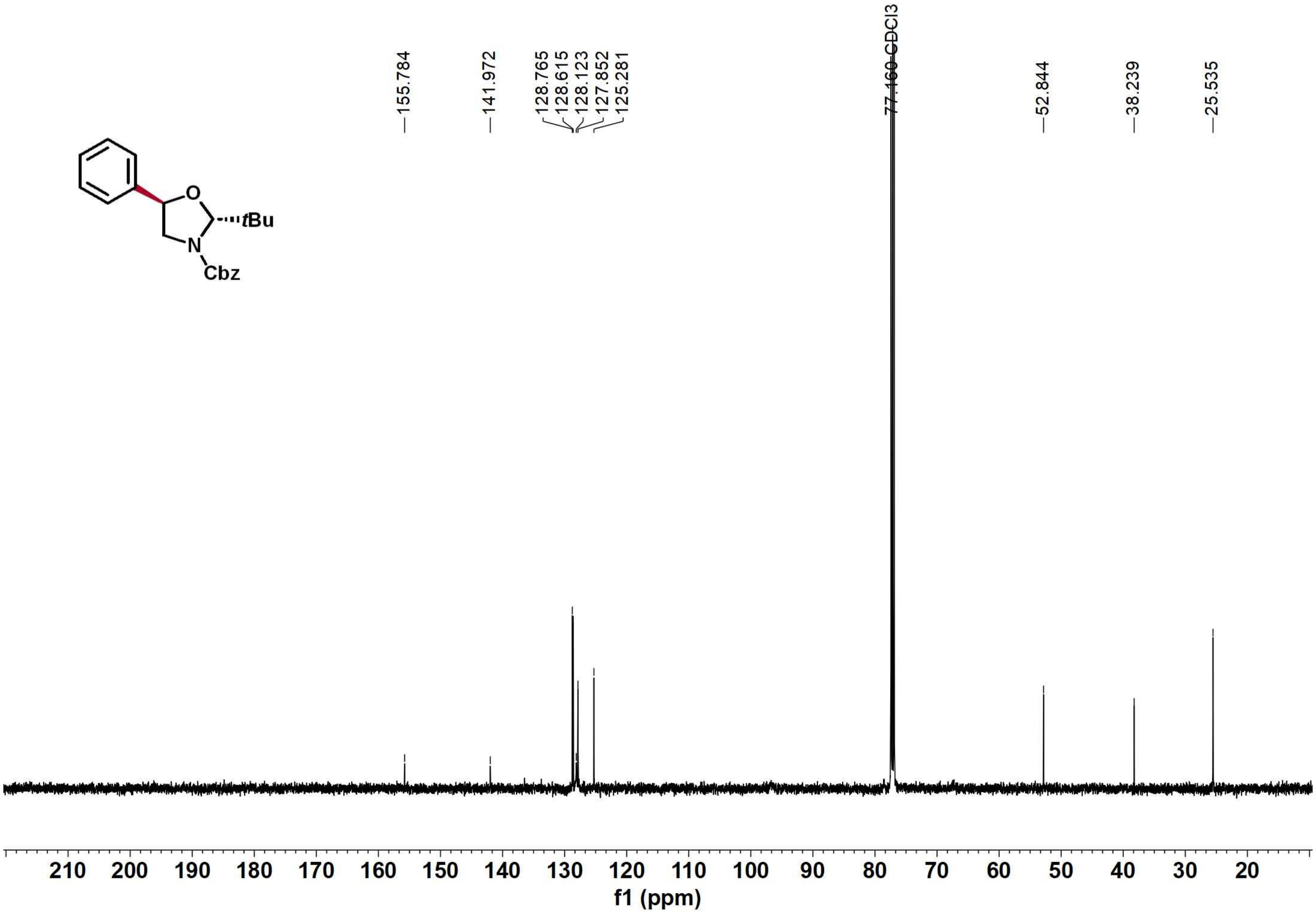

^{13}C NMR of Compound 17a (151 MHz, $CDCl_3$)

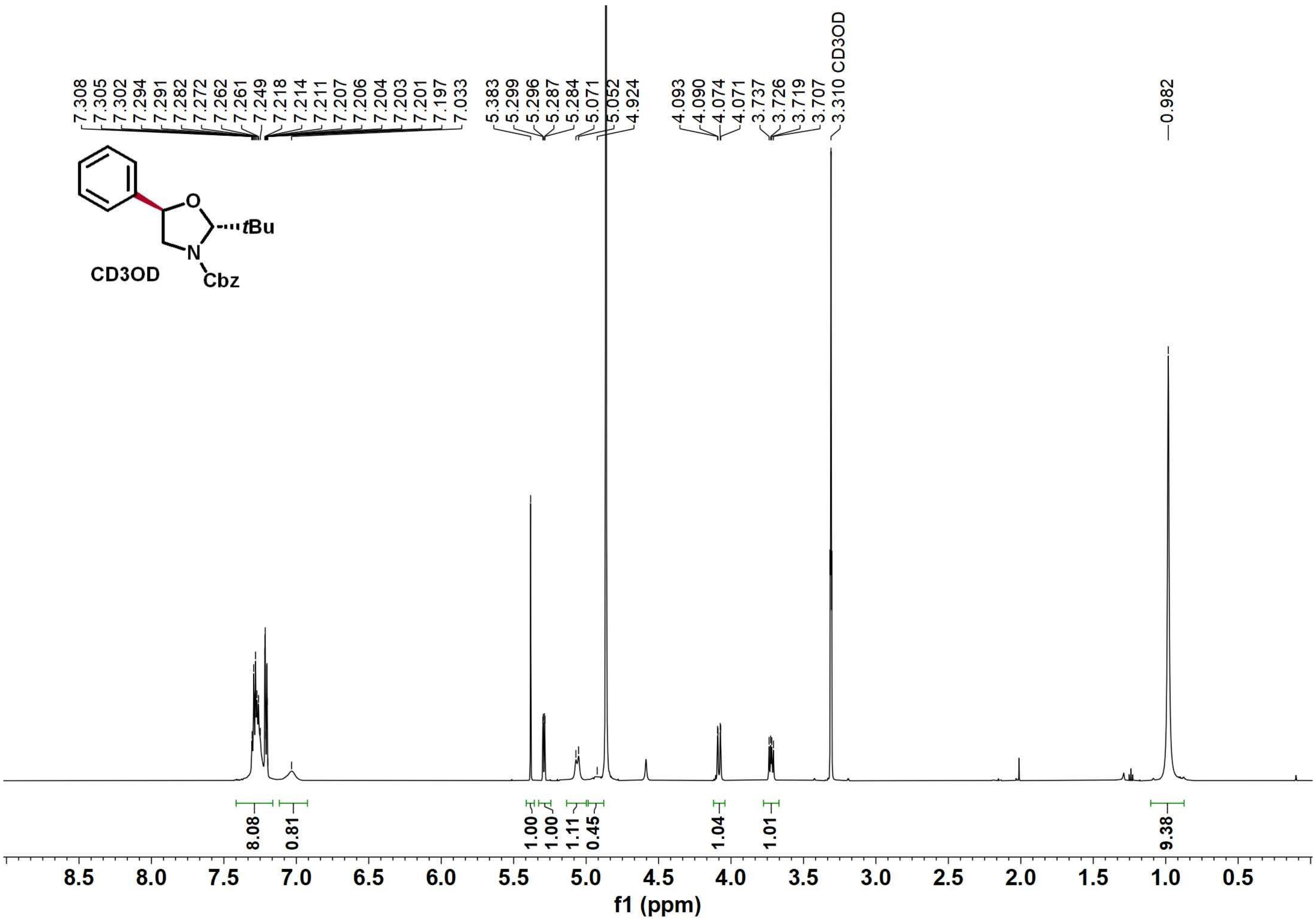

^{1}H NMR of Compound 17a (600 MHz, CD_3OD)

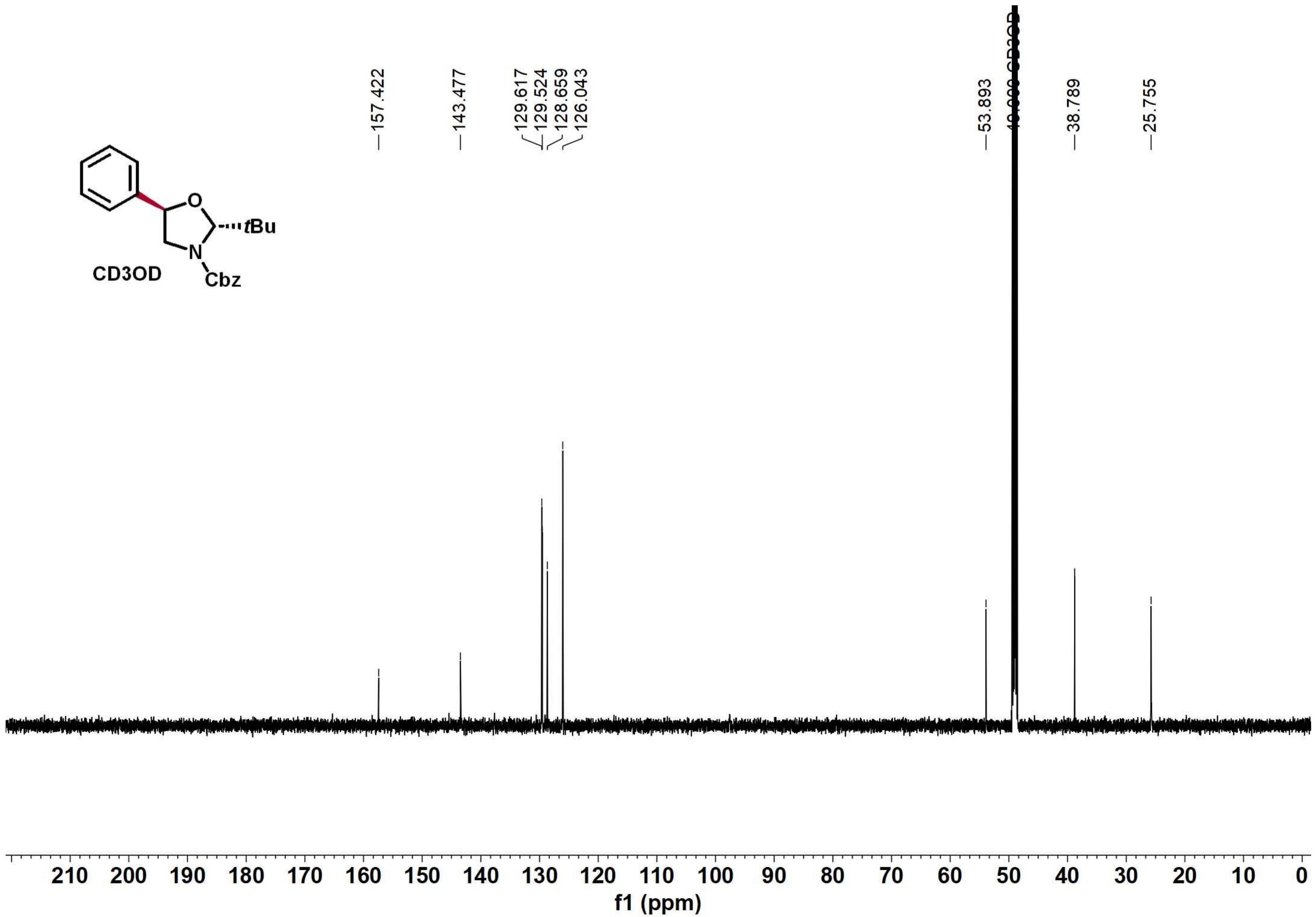

^{13}C NMR of Compound 17a (151 MHz, CD_3OD)

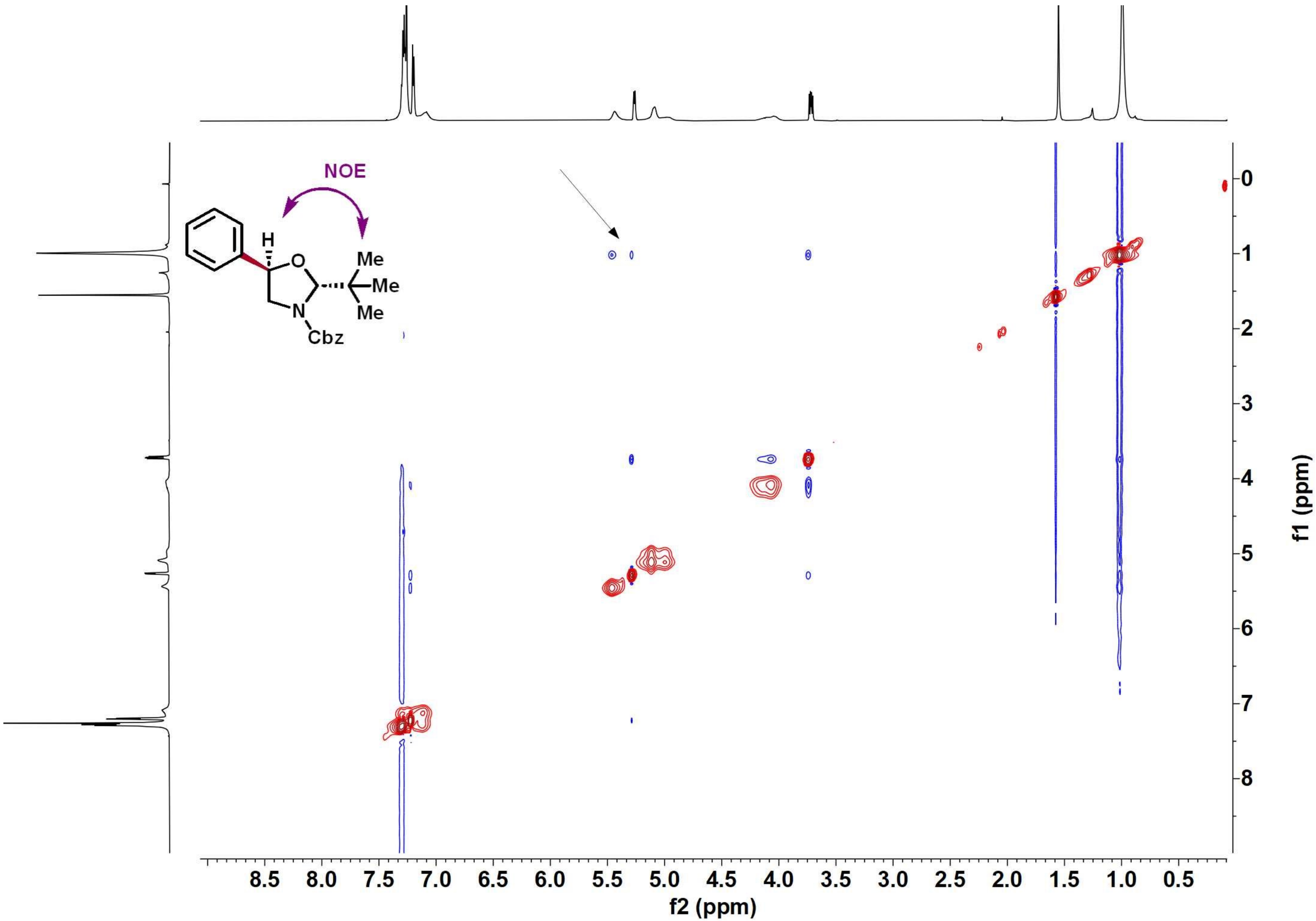

NORSY of Compound 17a (600 MHz, $CDCl_3$)

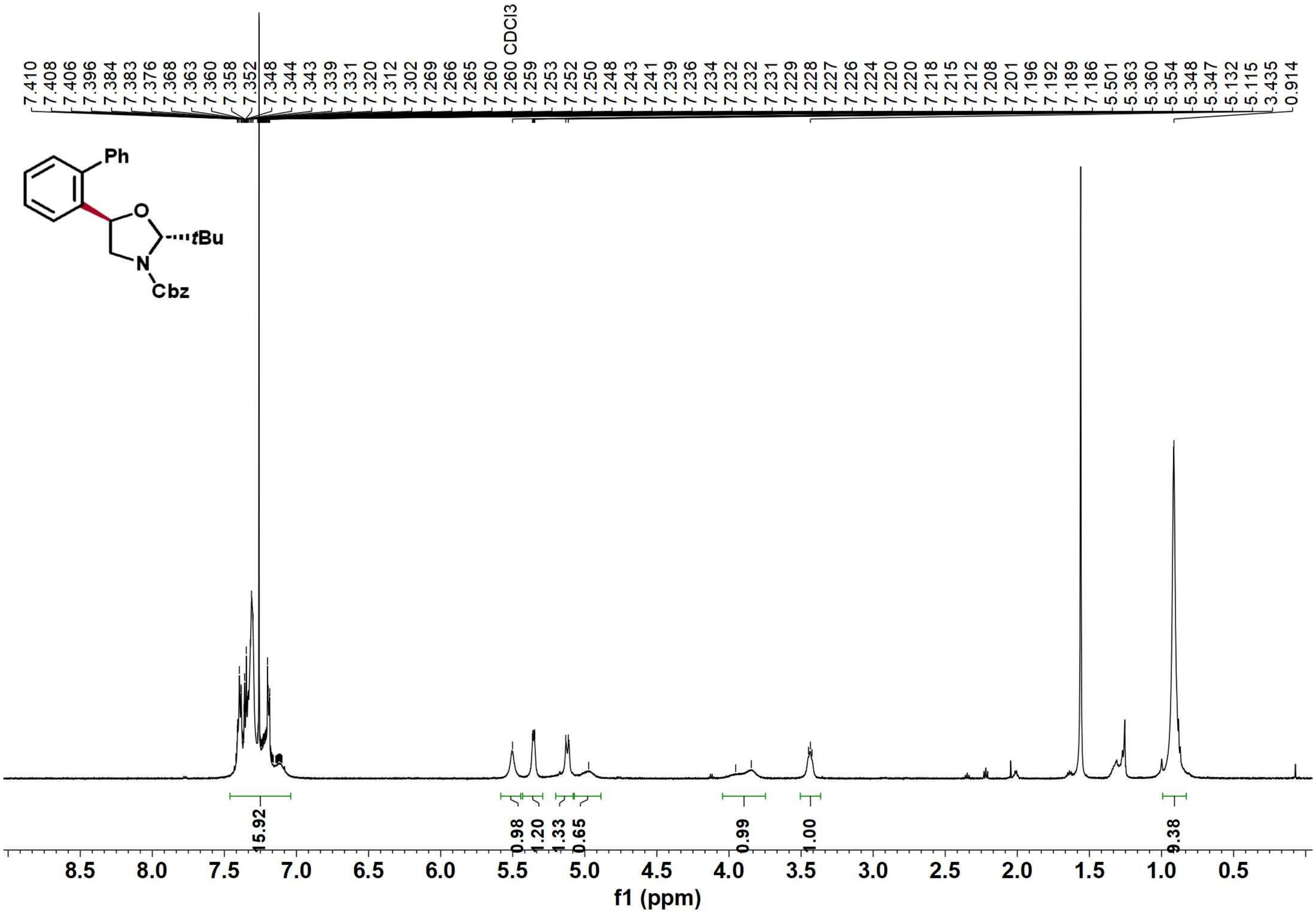

^{1}H NMR of Compound 17b (600 MHz, $CDCl_3$)

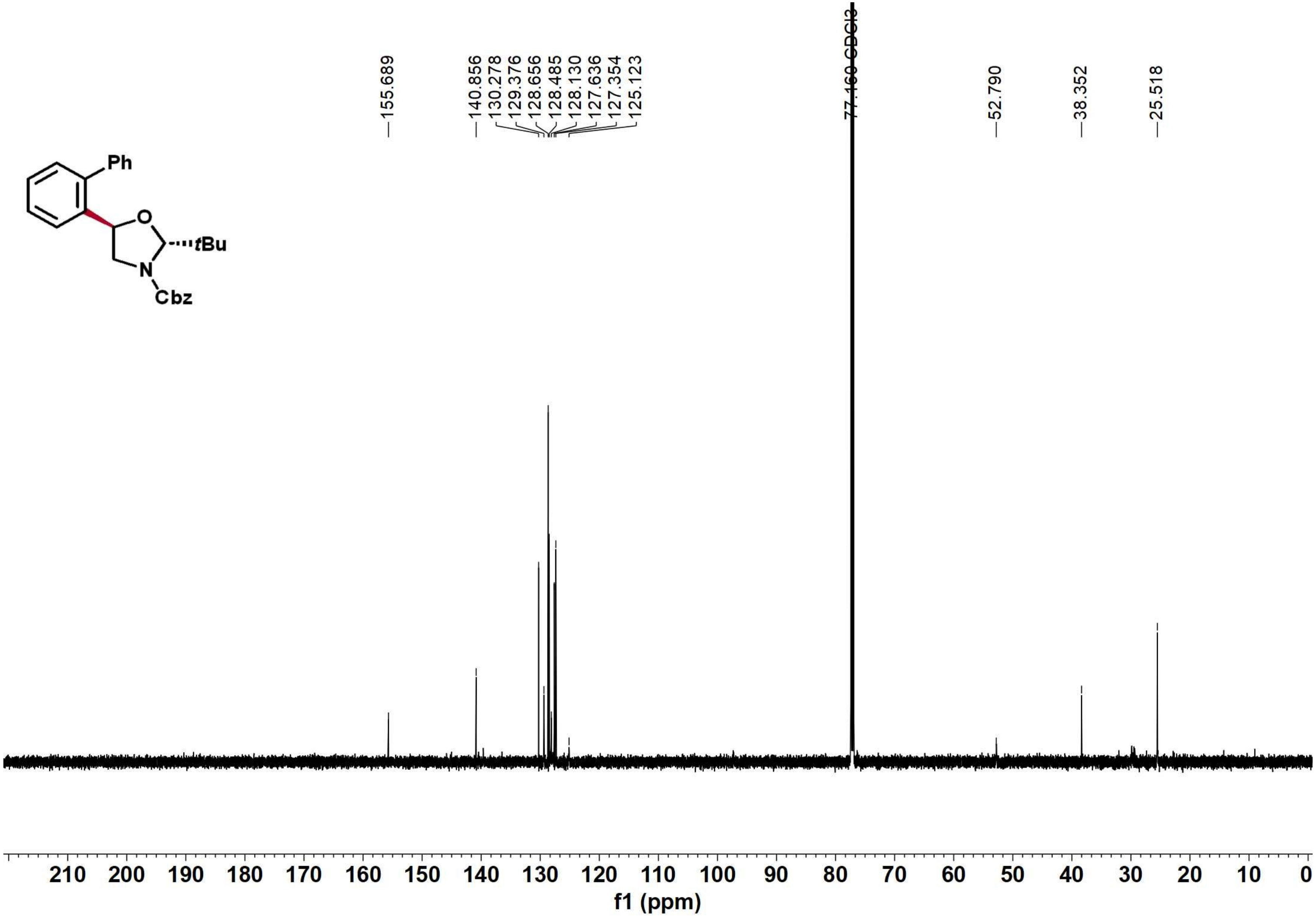

^{13}C NMR of Compound 17b (151 MHz, $CDCl_3$)

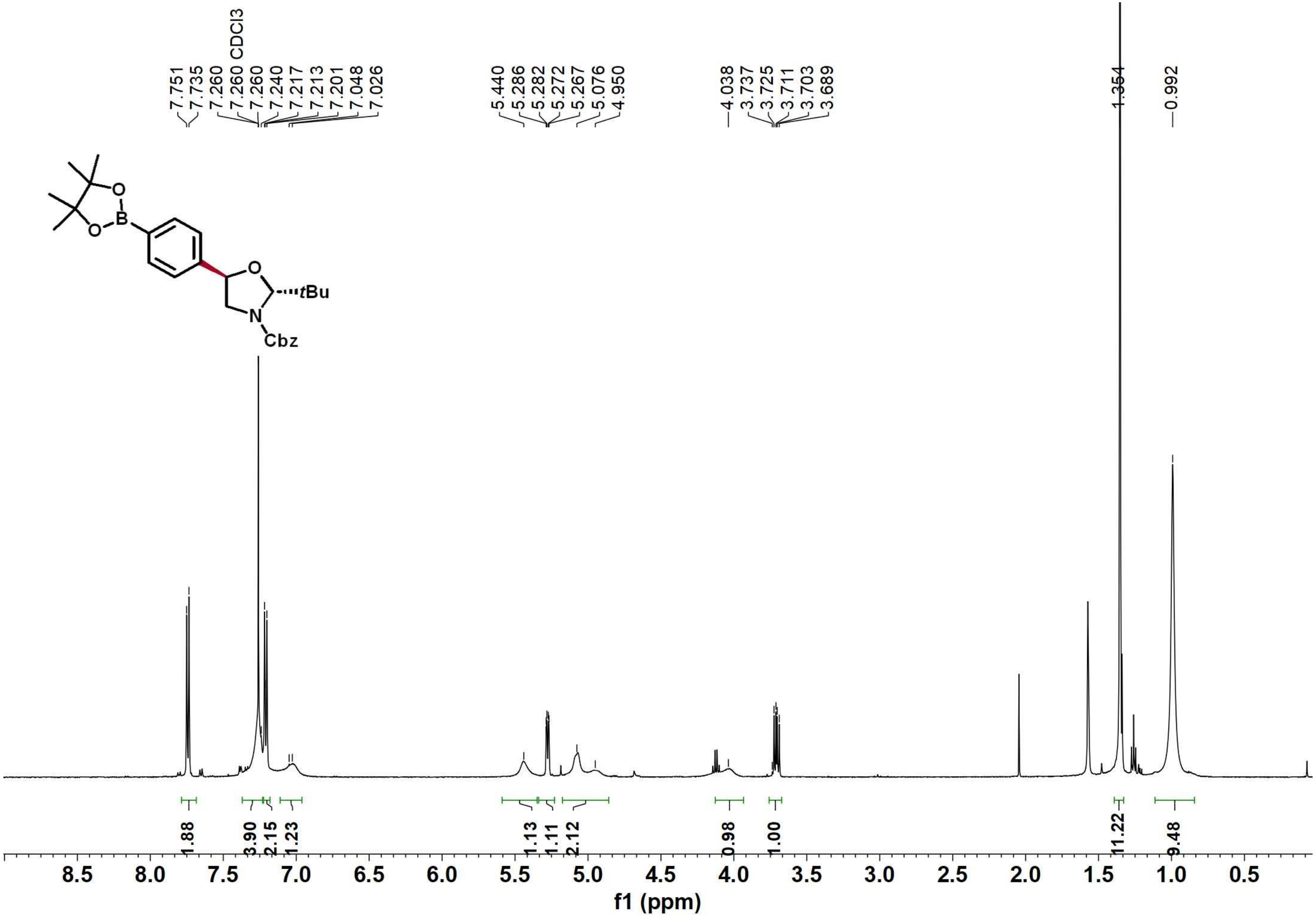

^{1}H NMR of Compound 17c (600 MHz, $CDCl_3$)

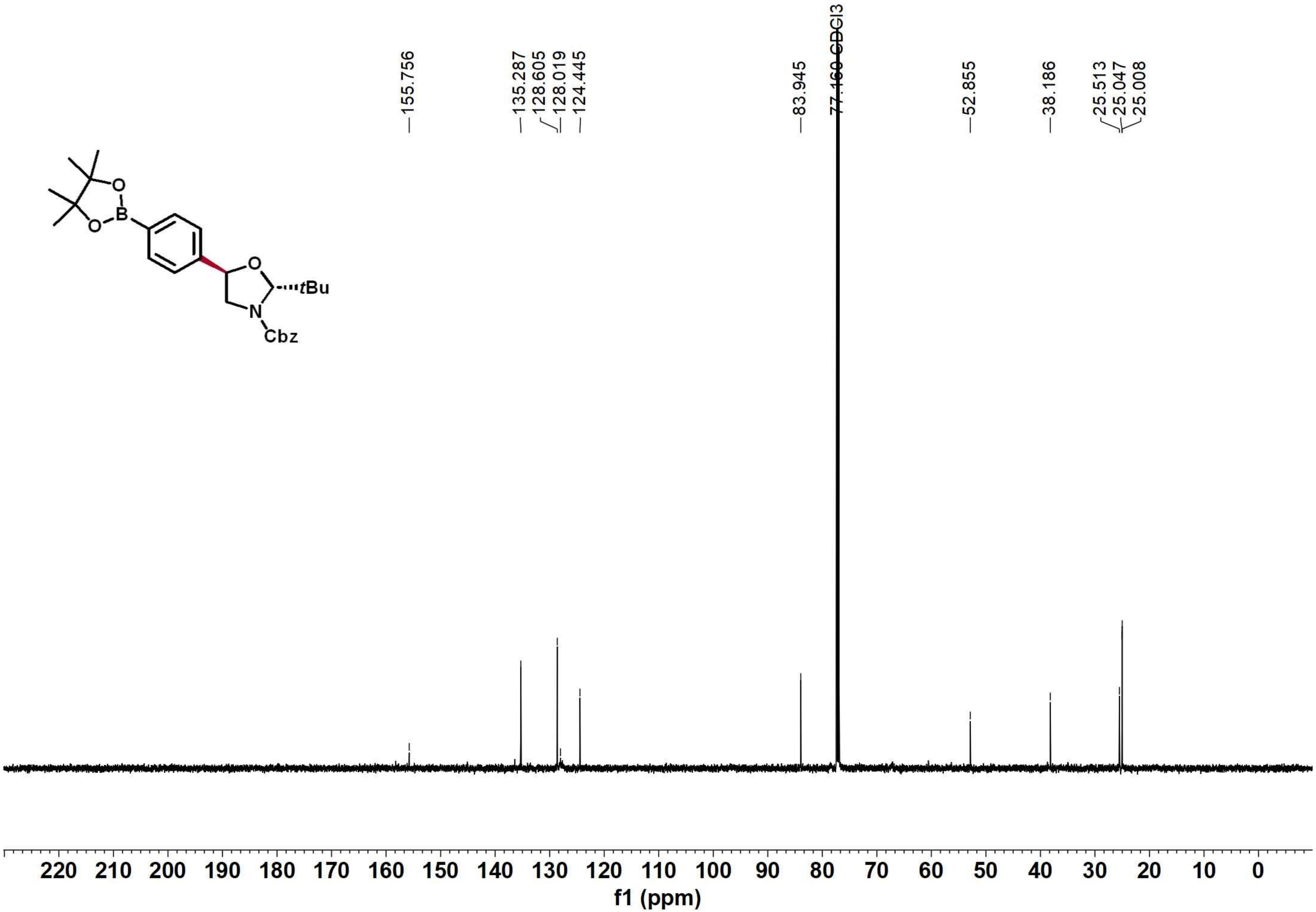

^{13}C NMR of Compound 17c (151 MHz, $CDCl_3$)

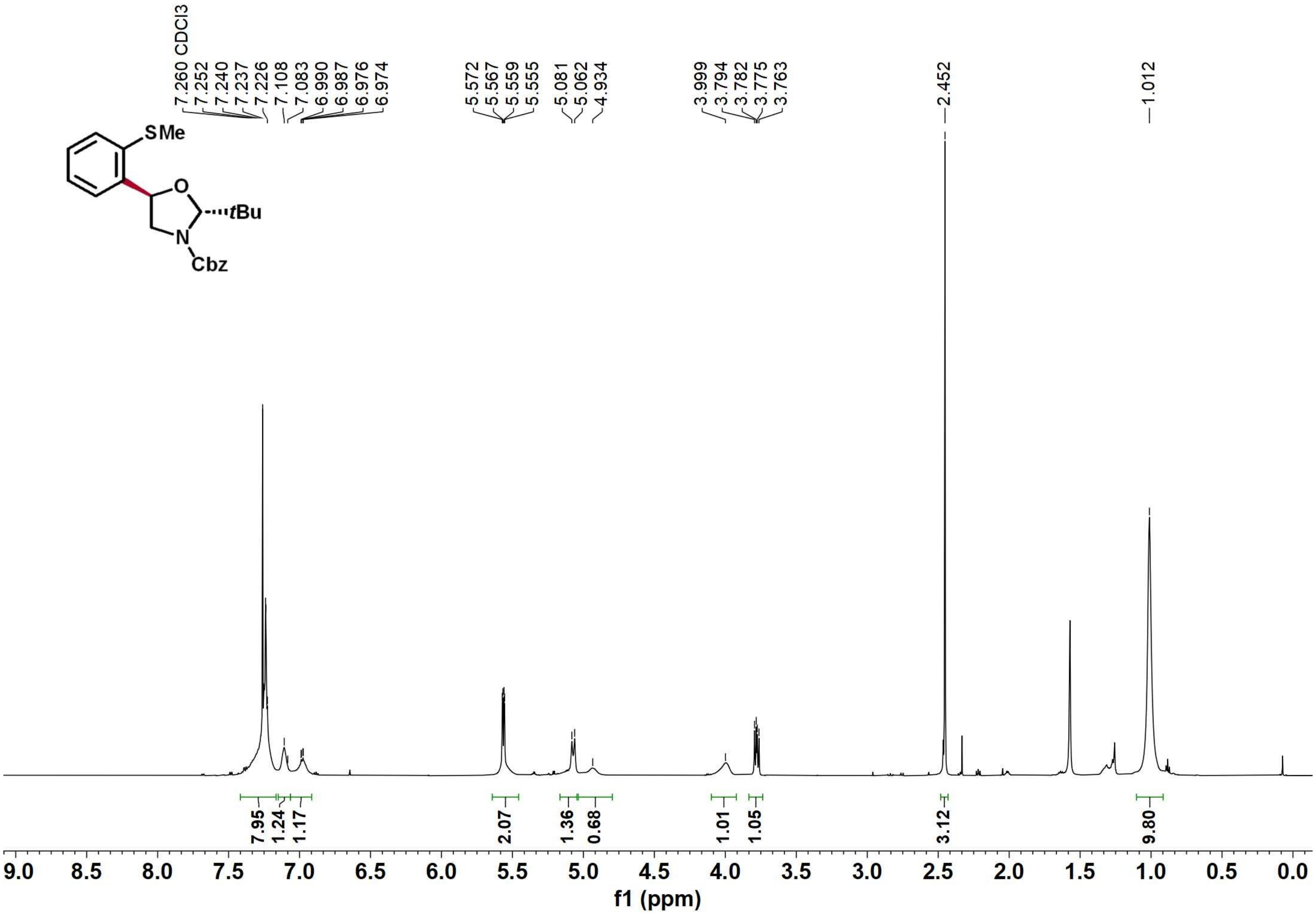

^{1}H NMR of Compound 17d (600 MHz, $CDCl_3$)

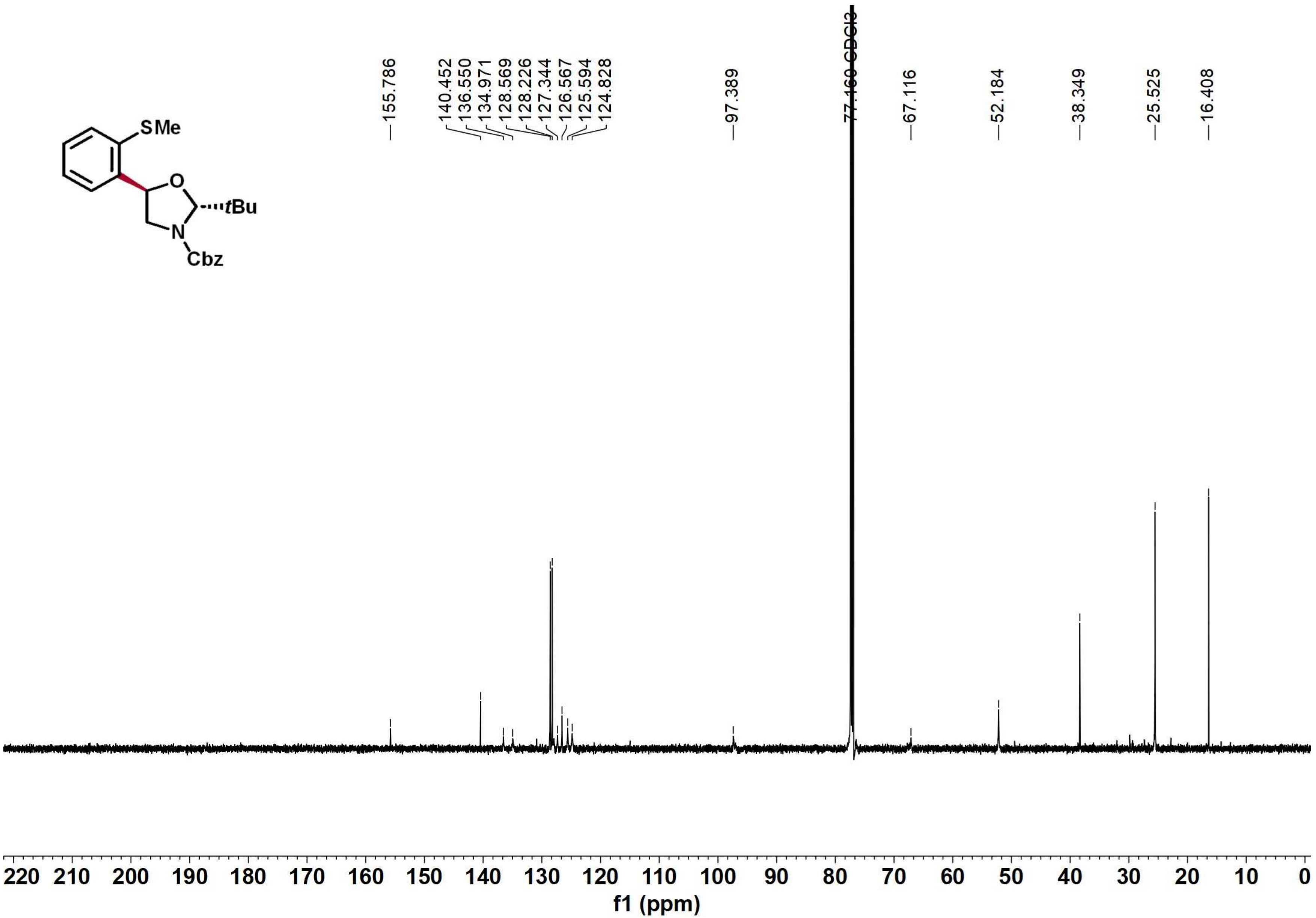

^{13}C NMR of Compound 17d (151 MHz, $CDCl_3$)

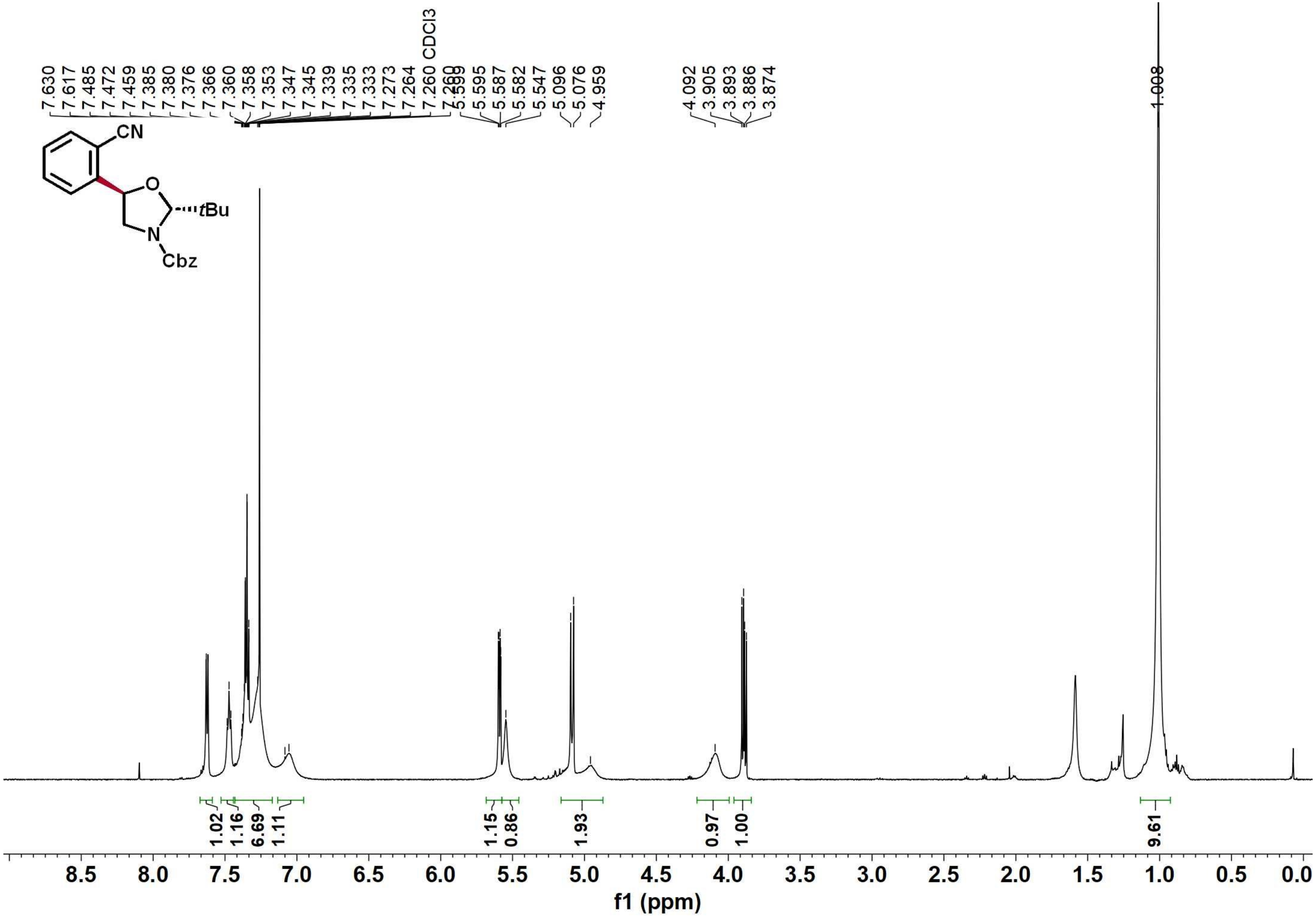

^{1}H NMR of Compound 17e (600 MHz, $CDCl_3$)

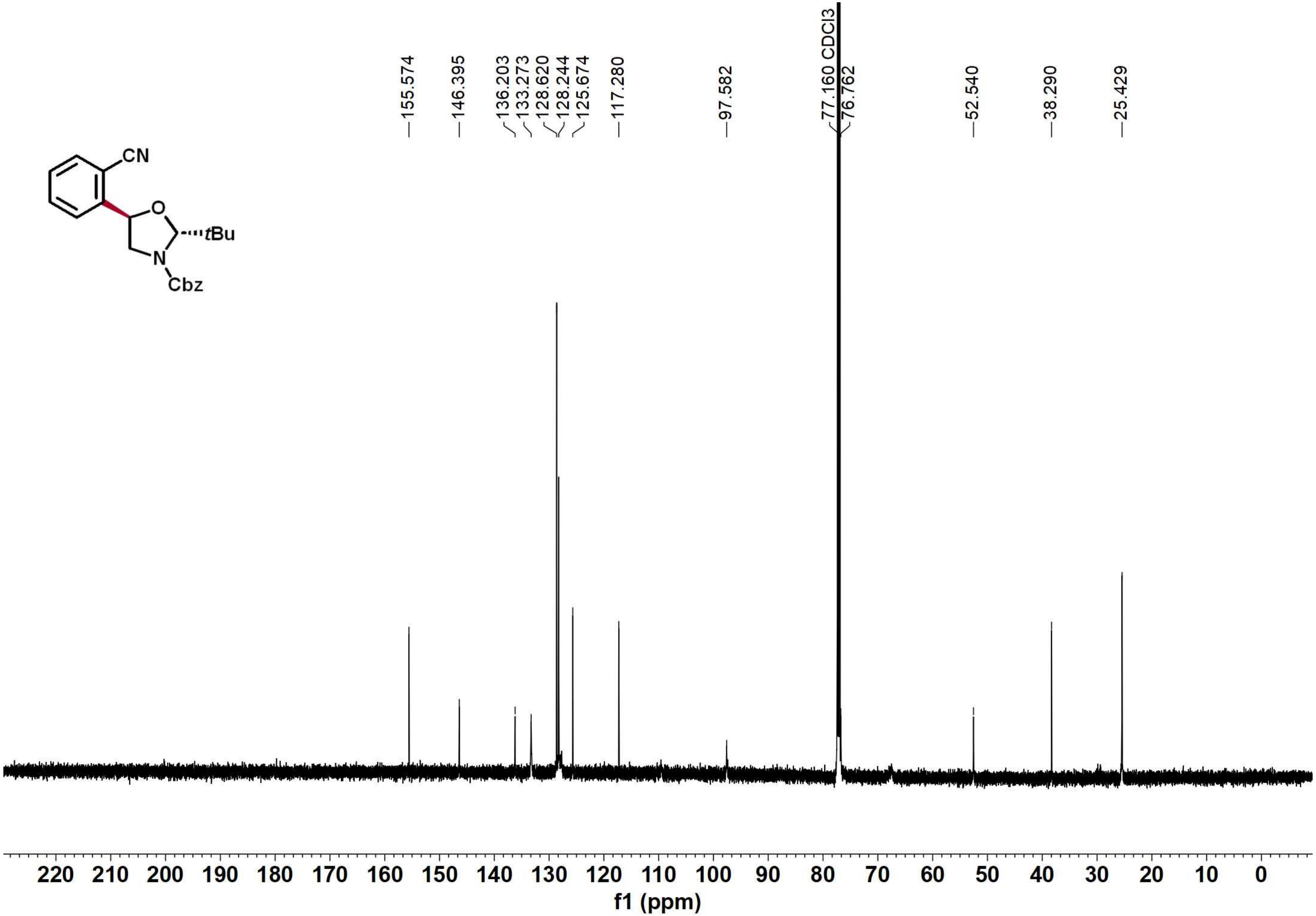

^{13}C NMR of Compound 17e (151 MHz, $CDCl_3$)

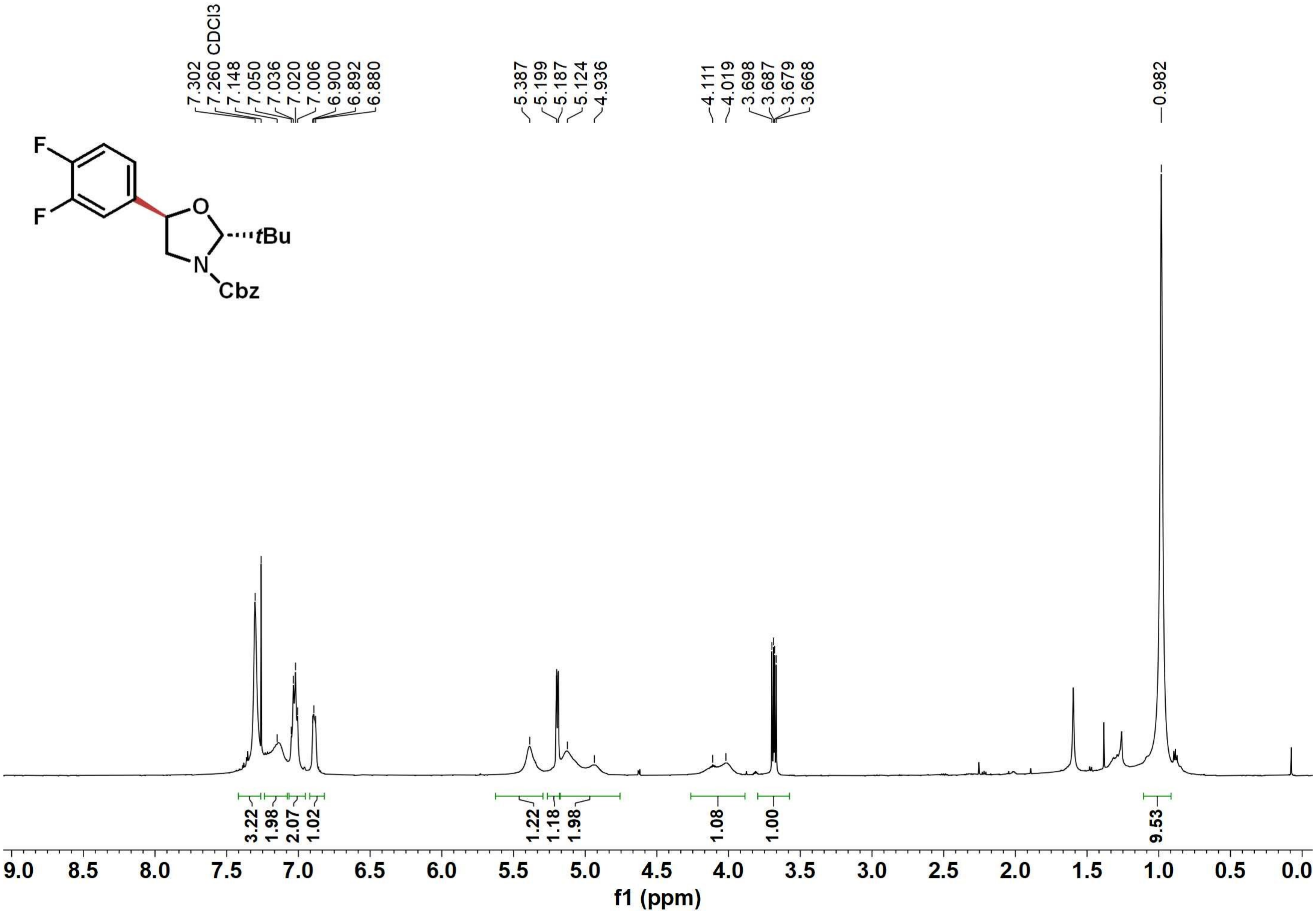

^{1}H NMR of Compound 17f (600 MHz, $CDCl_3$)

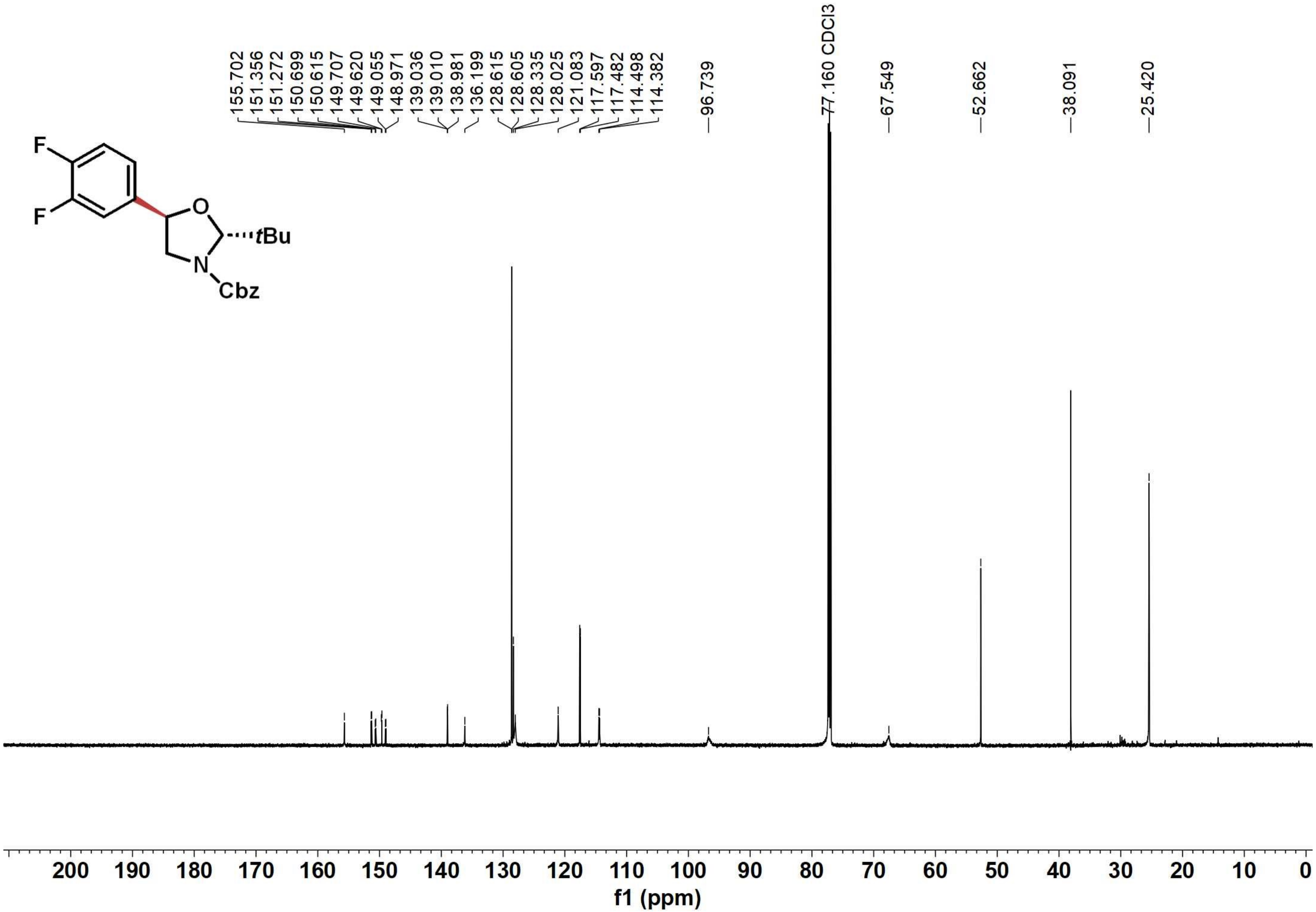

^{13}C NMR of Compound 17f (151 MHz, $CDCl_3$)

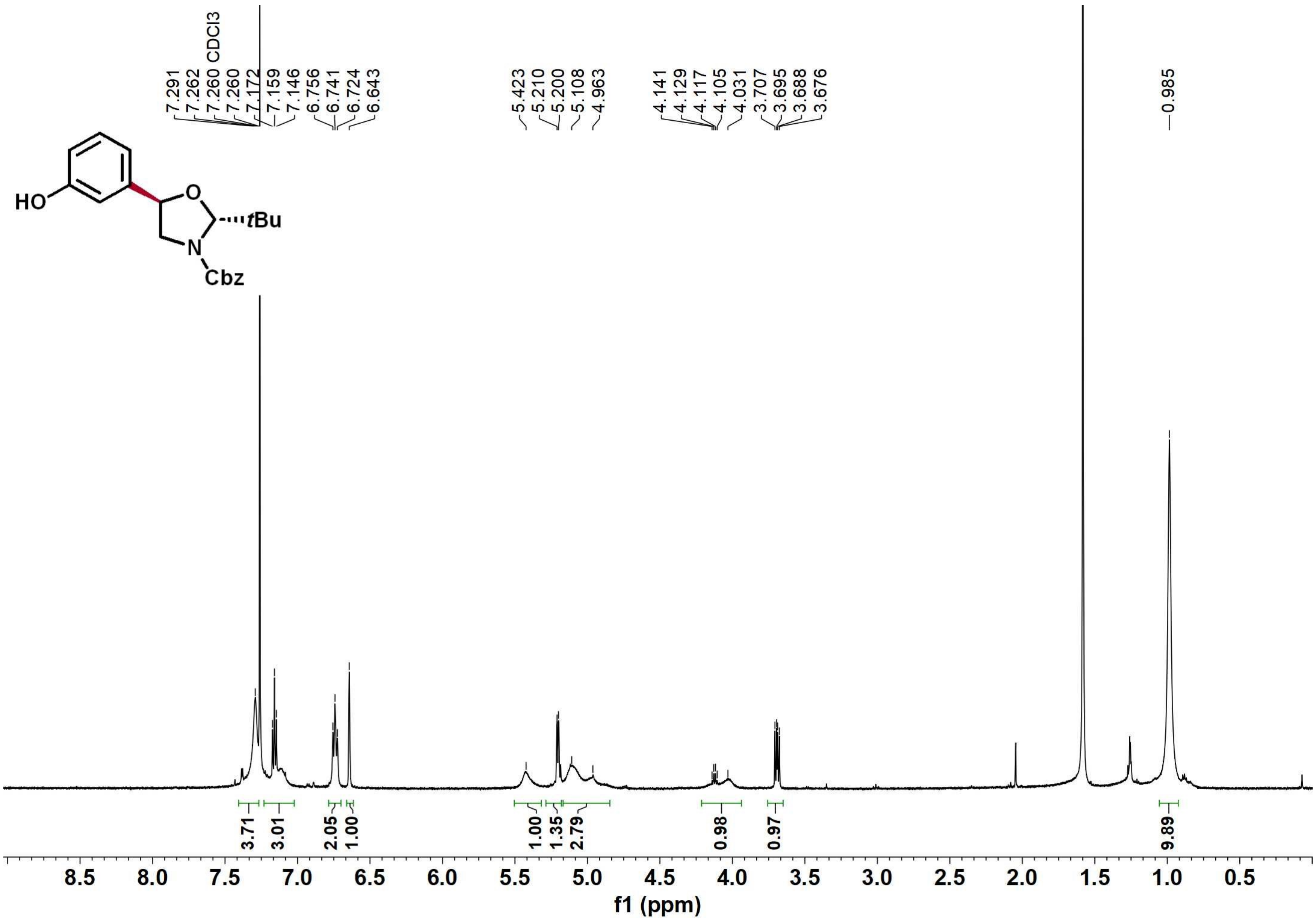

^{1}H NMR of Compound 17g (600 MHz, $CDCl_3$)

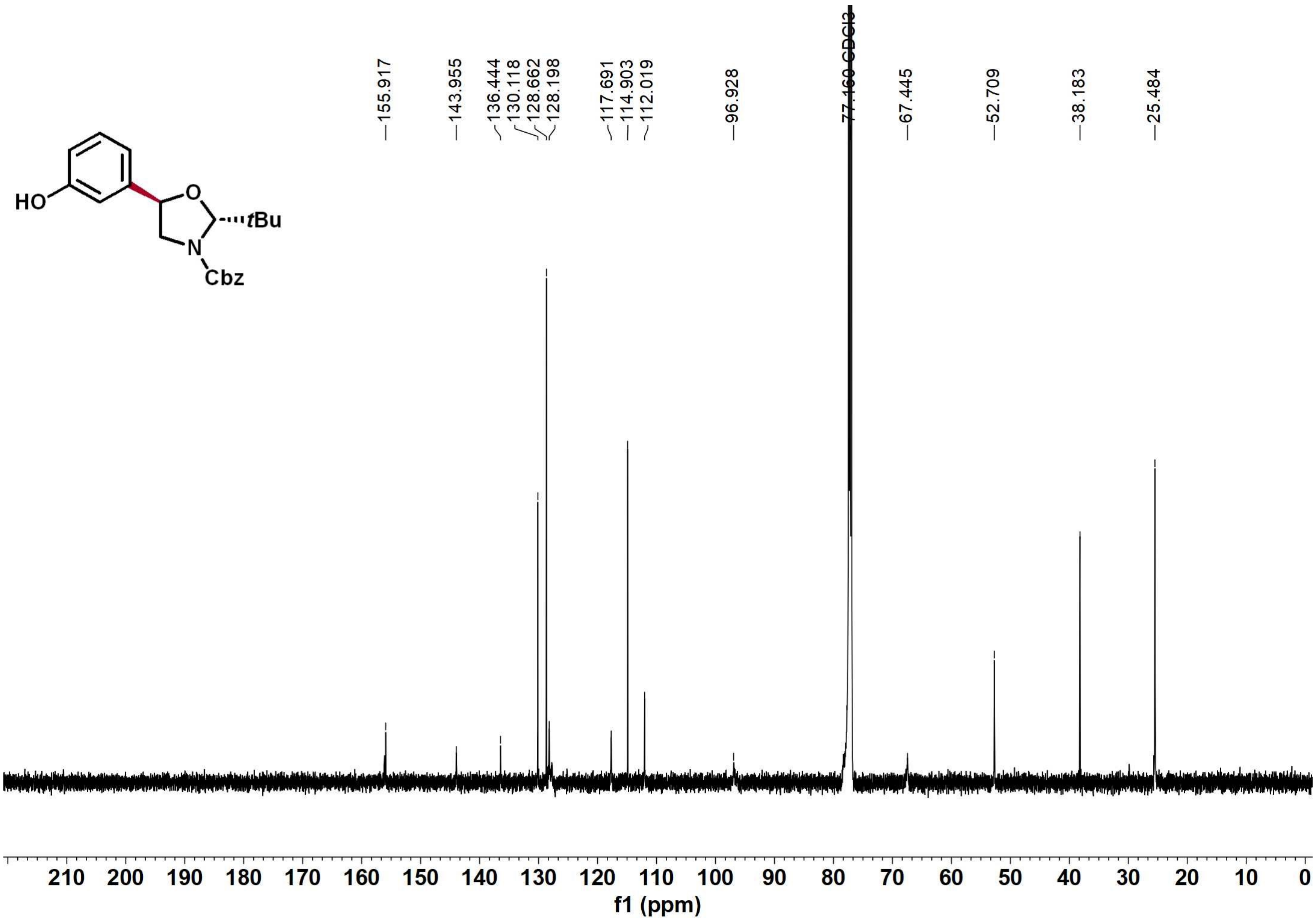

^{13}C NMR of Compound 17g (151 MHz, $CDCl_3$)

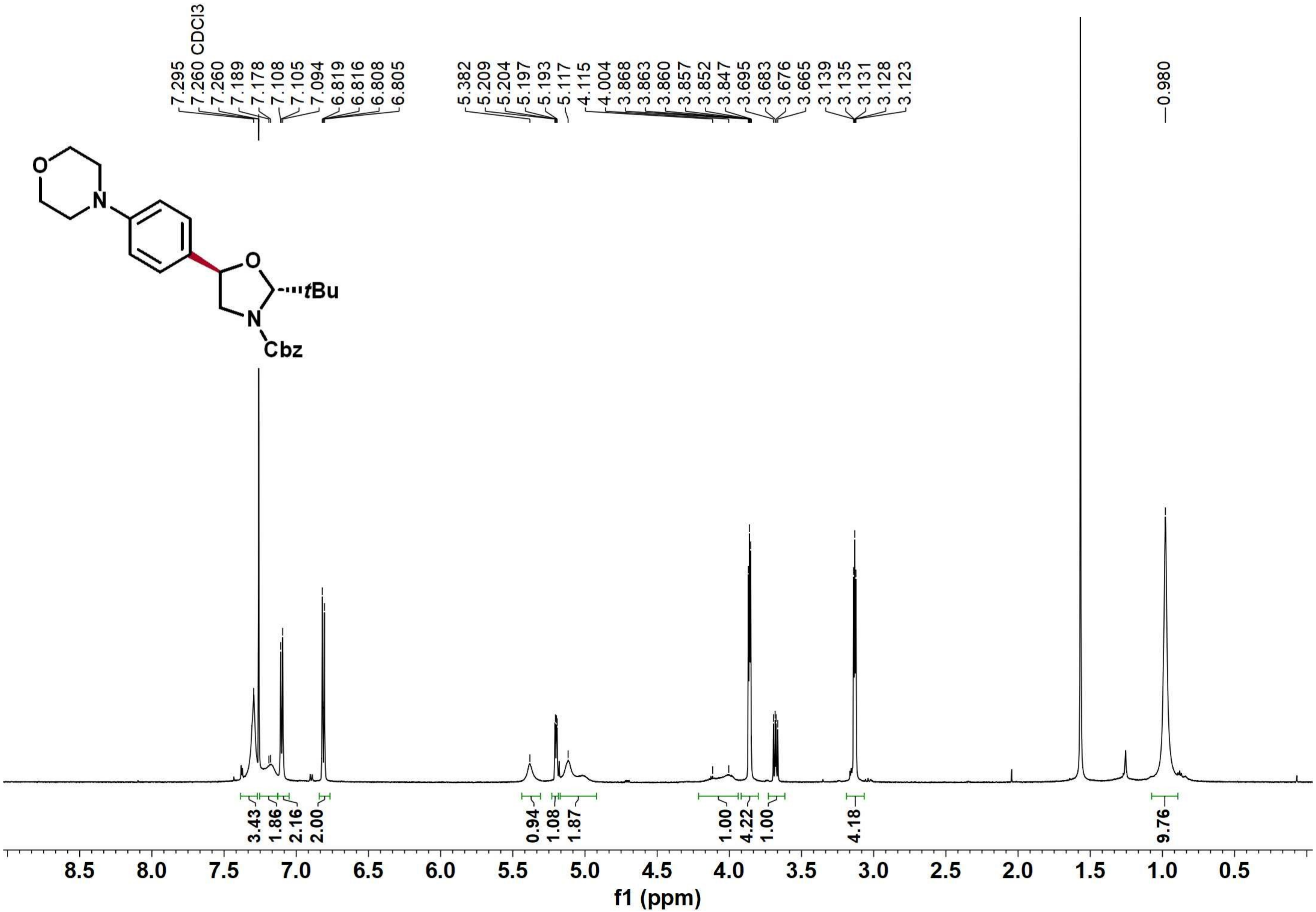

^{1}H NMR of Compound 17h (600 MHz, $CDCl_3$)

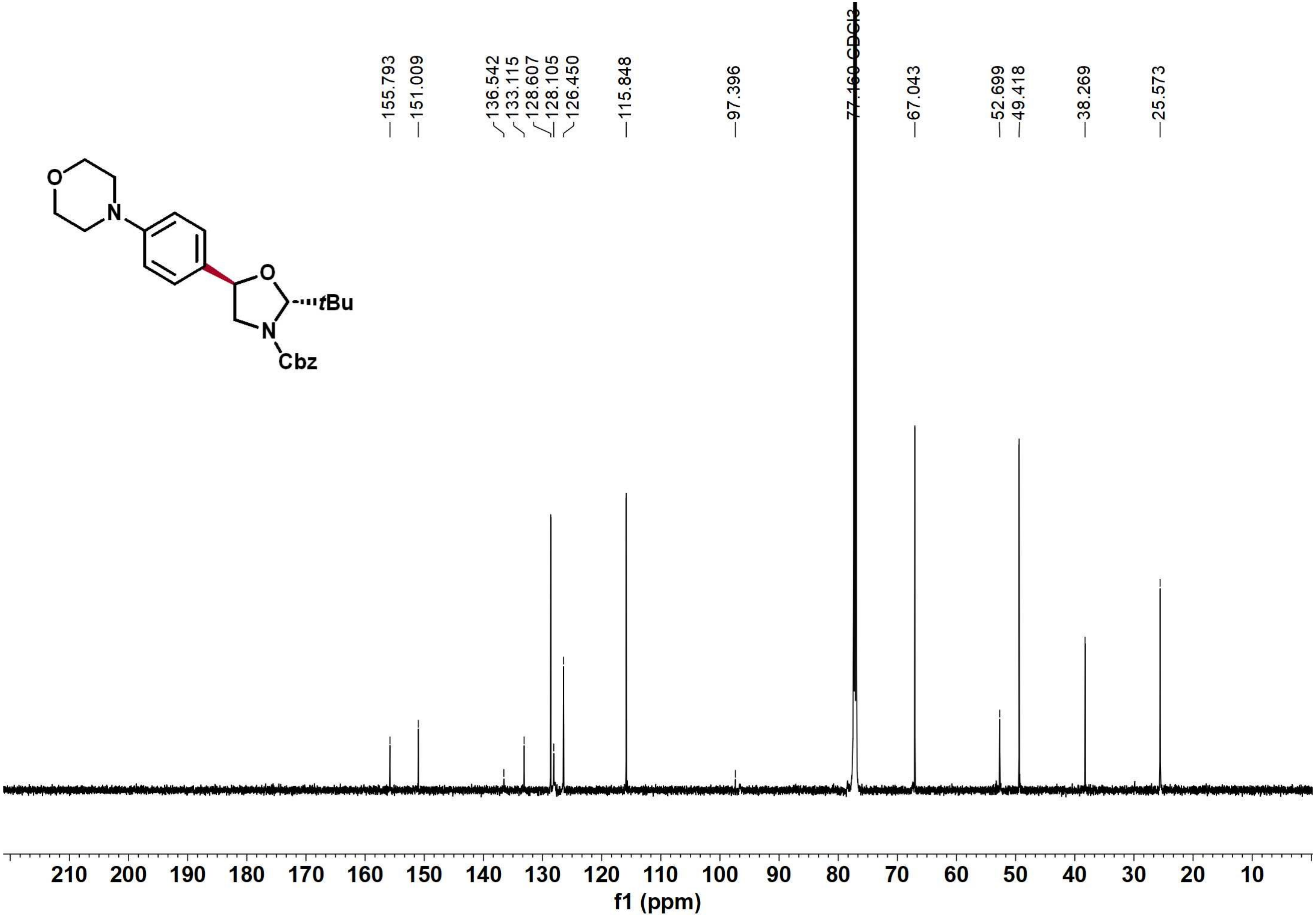

^{13}C NMR of Compound 17h (151 MHz, $CDCl_3$)

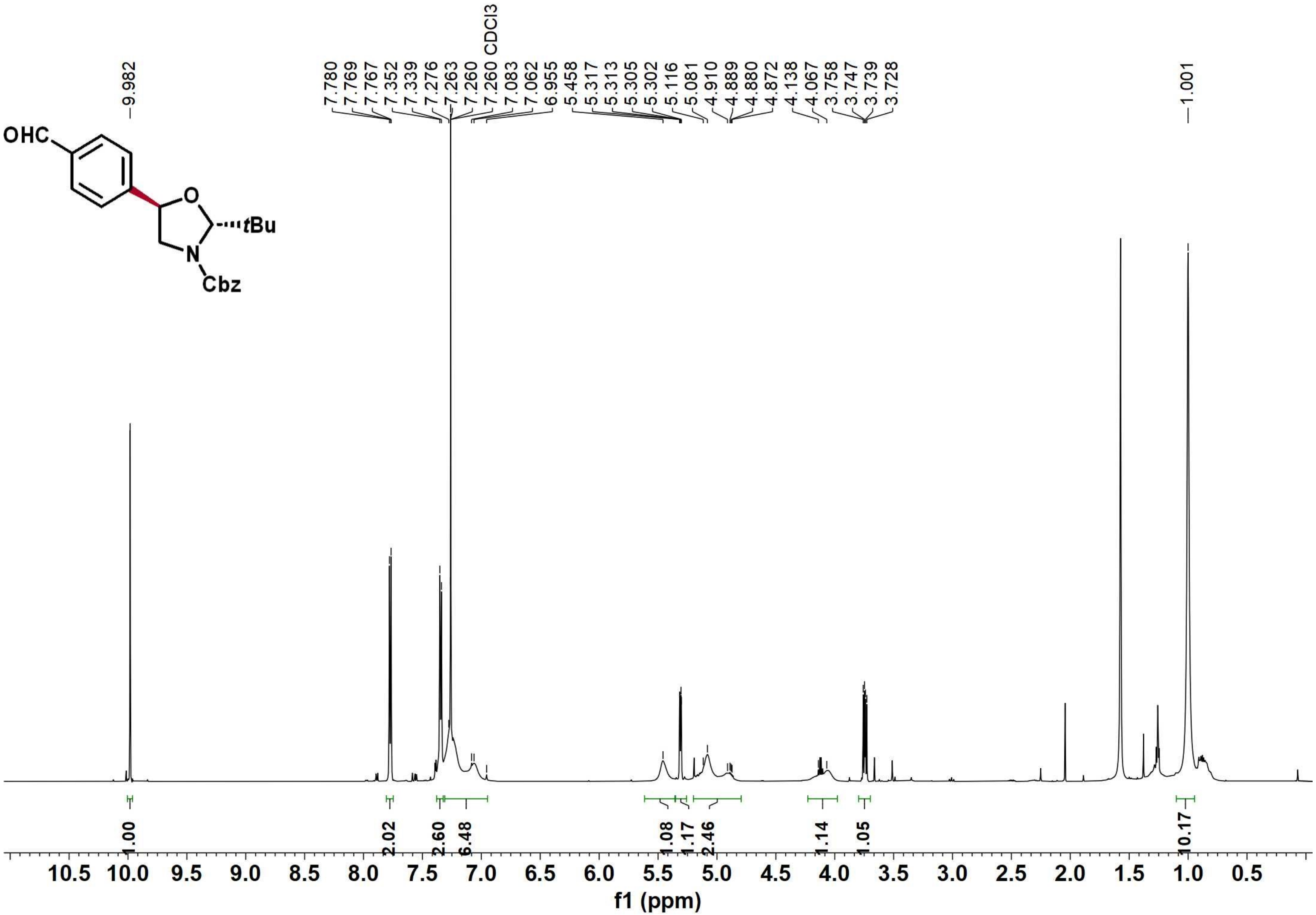

^{1}H NMR of Compound 17i (600 MHz, $CDCl_3$)

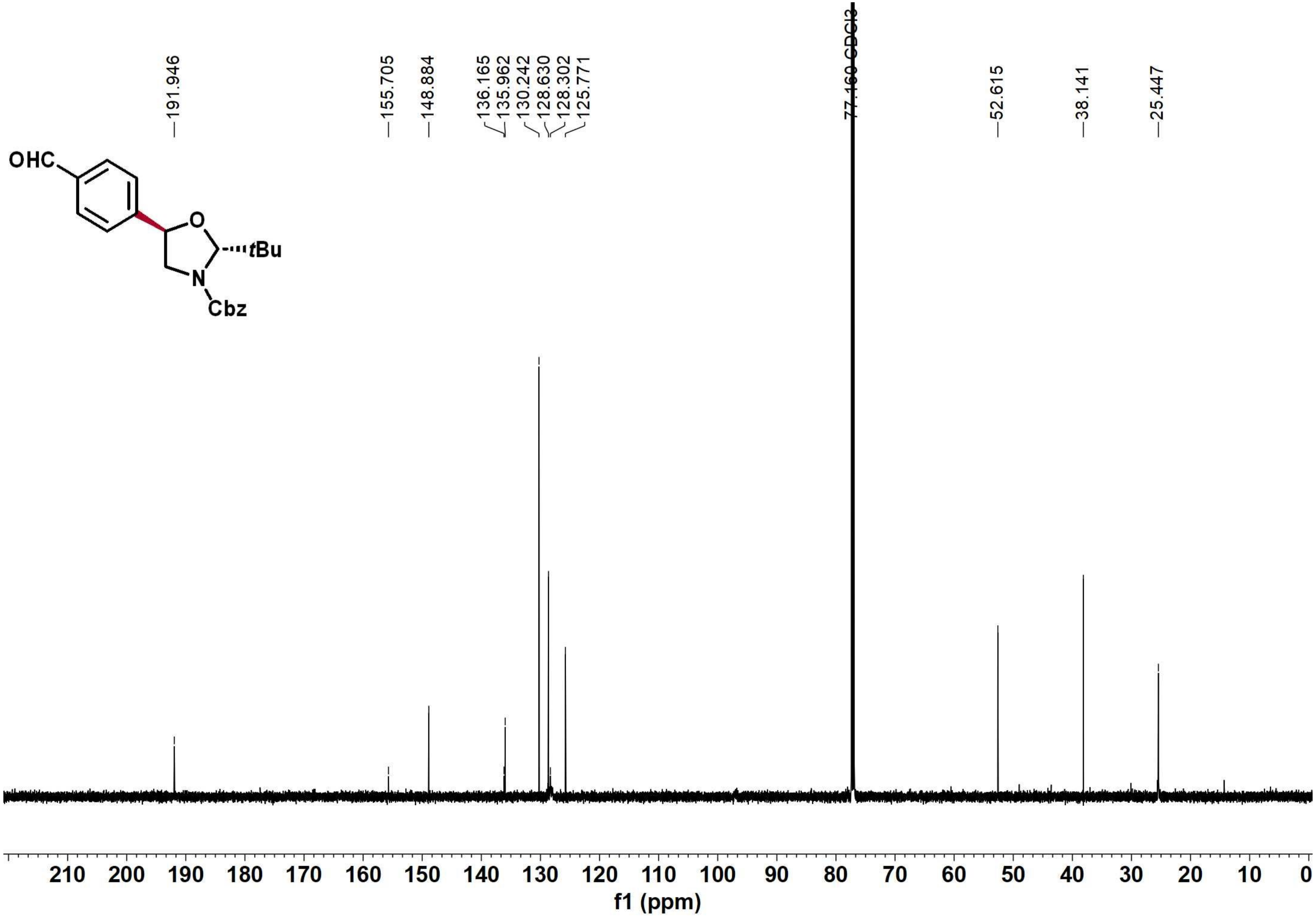

^{13}C NMR of Compound 17i (151 MHz, $CDCl_3$)

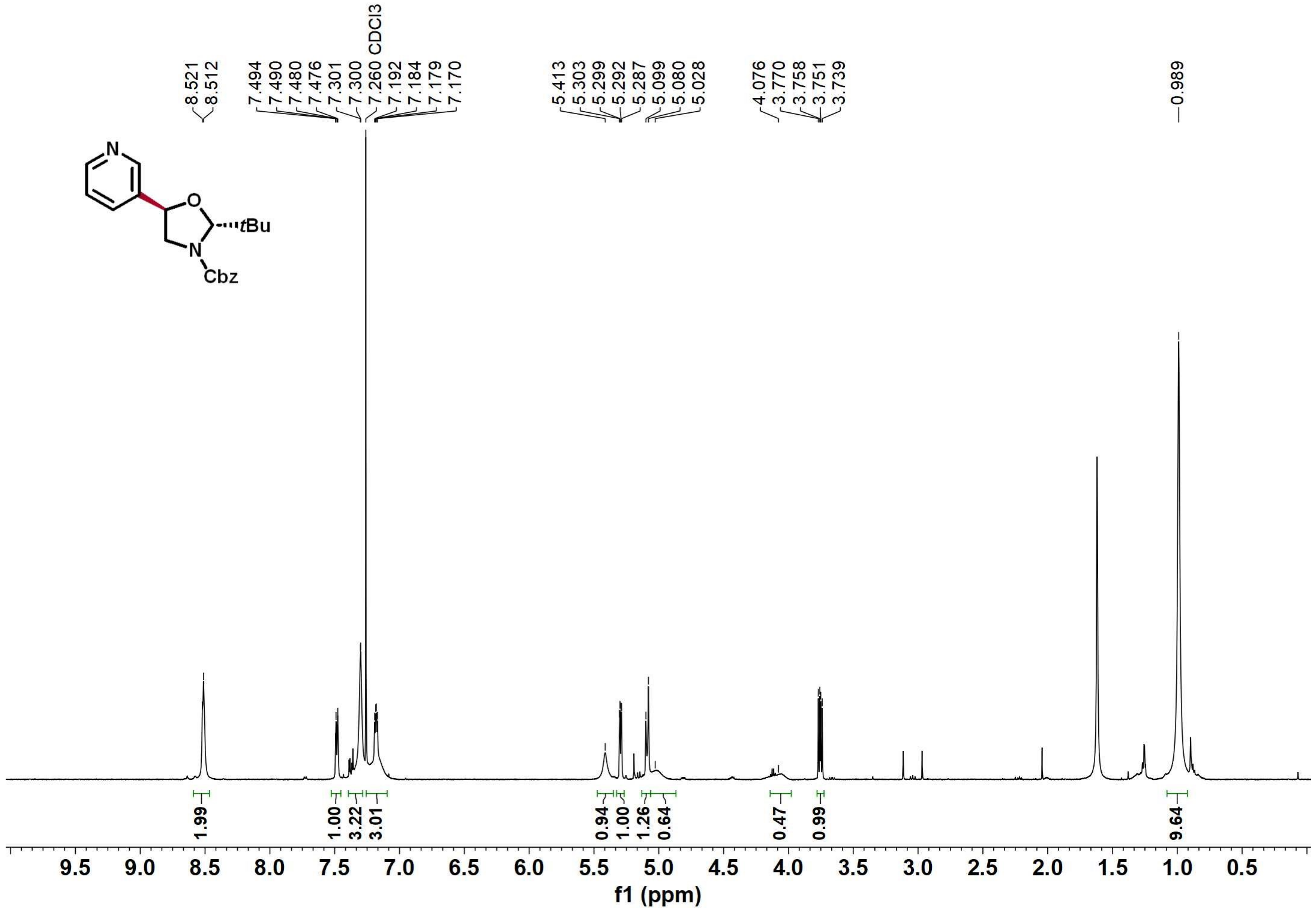

^{1}H NMR of Compound 17j (600 MHz, $CDCl_3$)

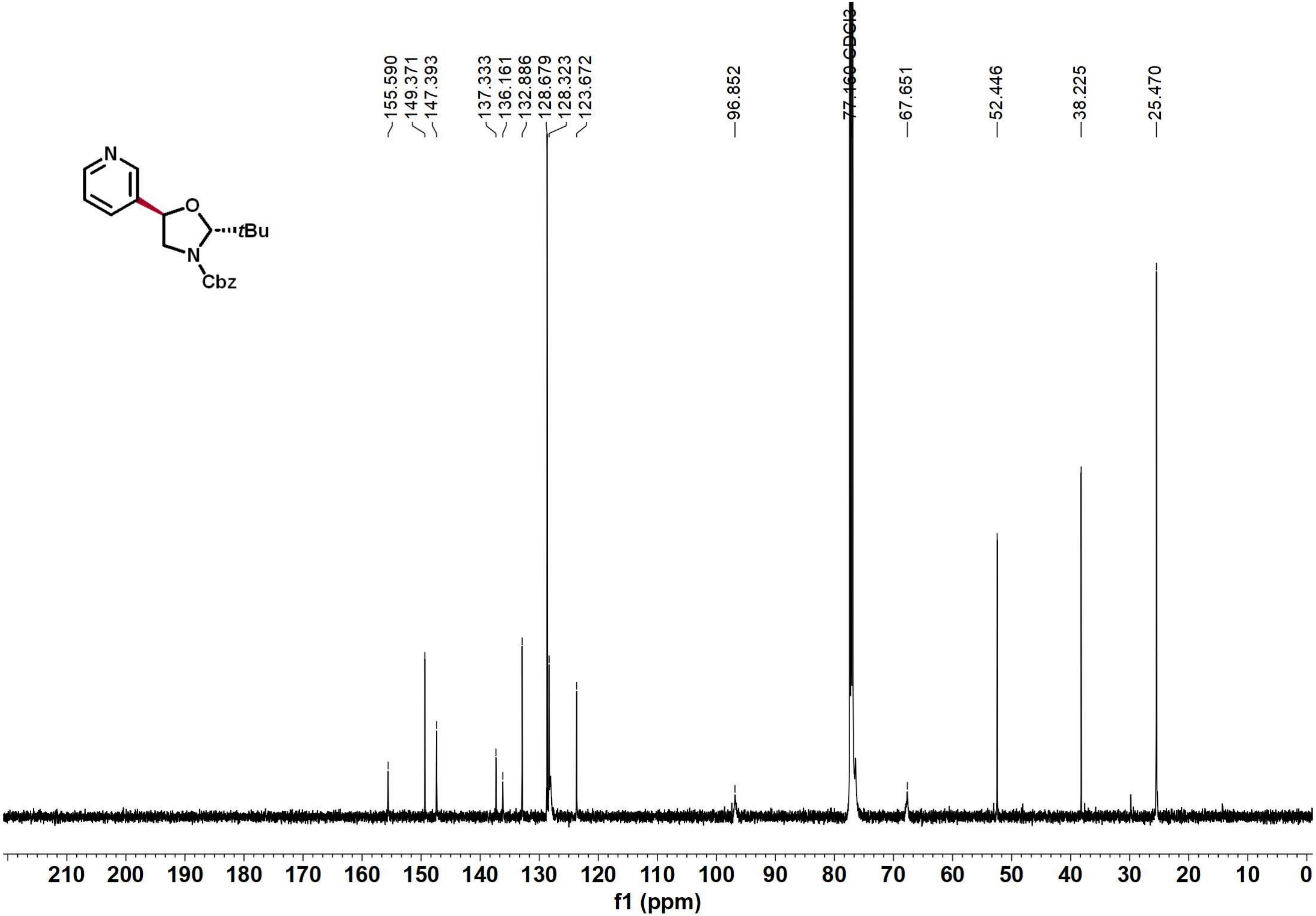

^{13}C NMR of Compound 17j (151 MHz, $CDCl_3$)

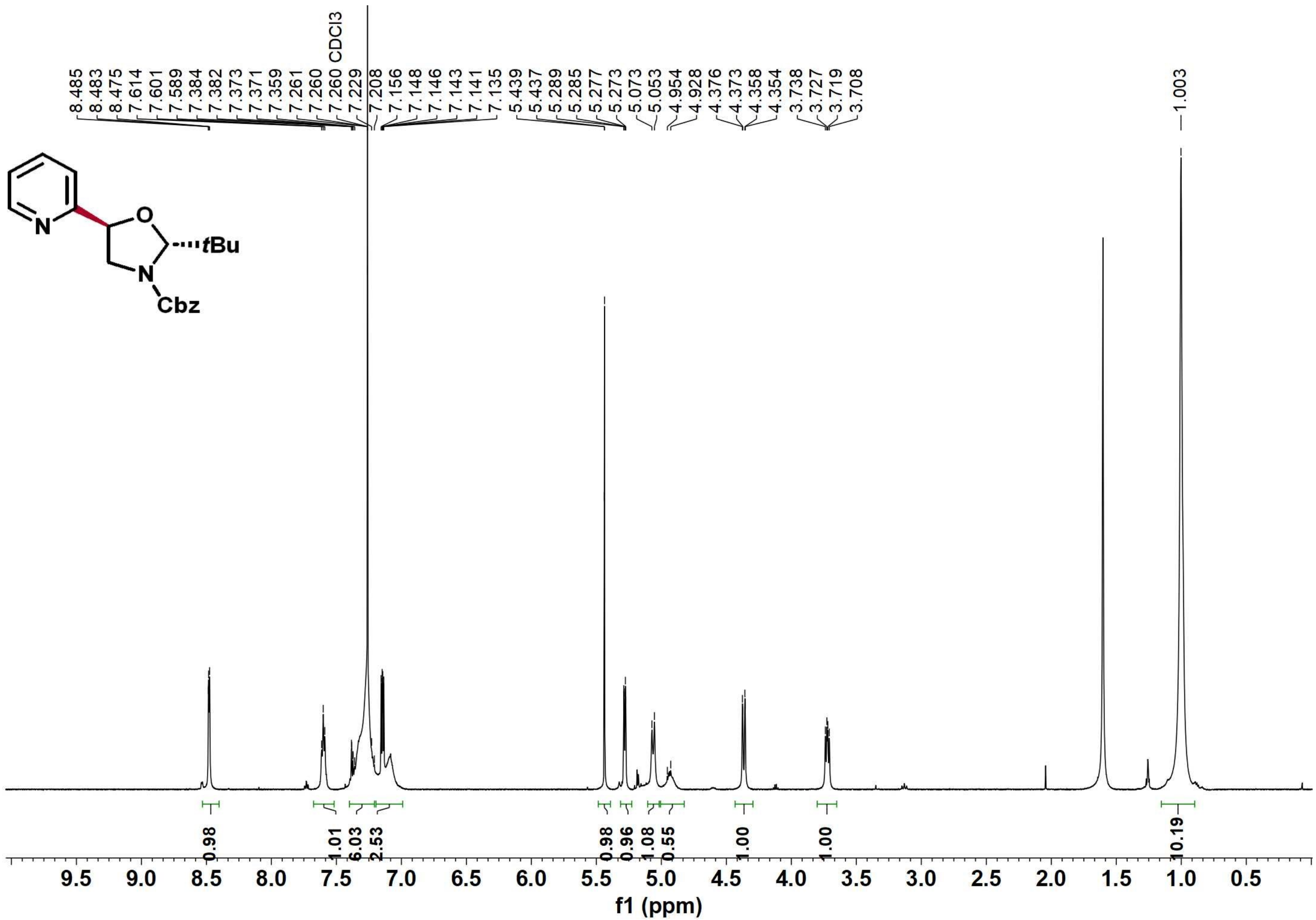

^{1}H NMR of Compound 17k (600 MHz, $CDCl_3$)

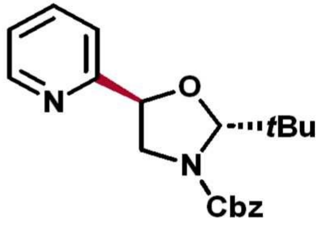

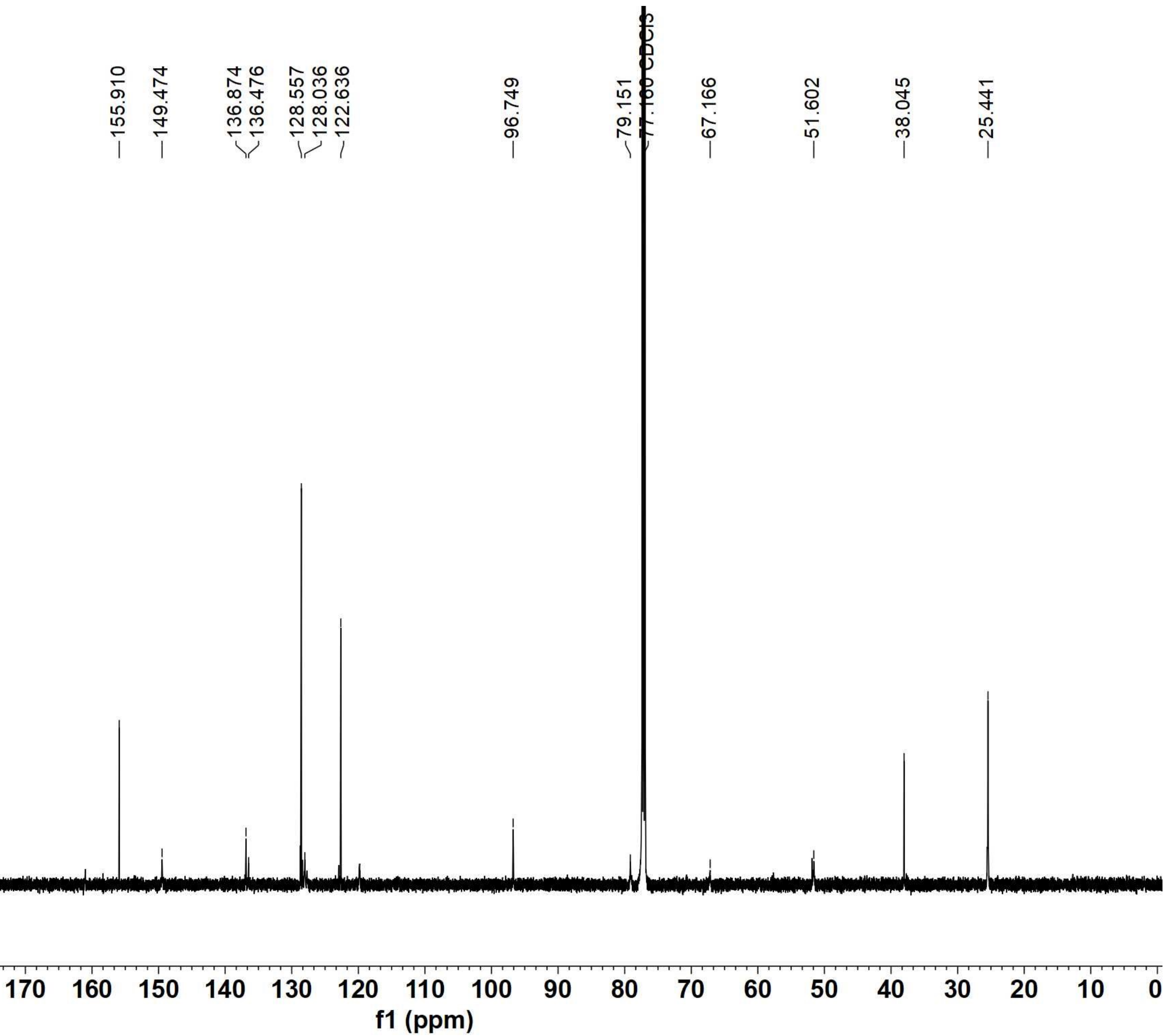

^{13}C NMR of Compound 17k (151 MHz, $CDCl_3$)

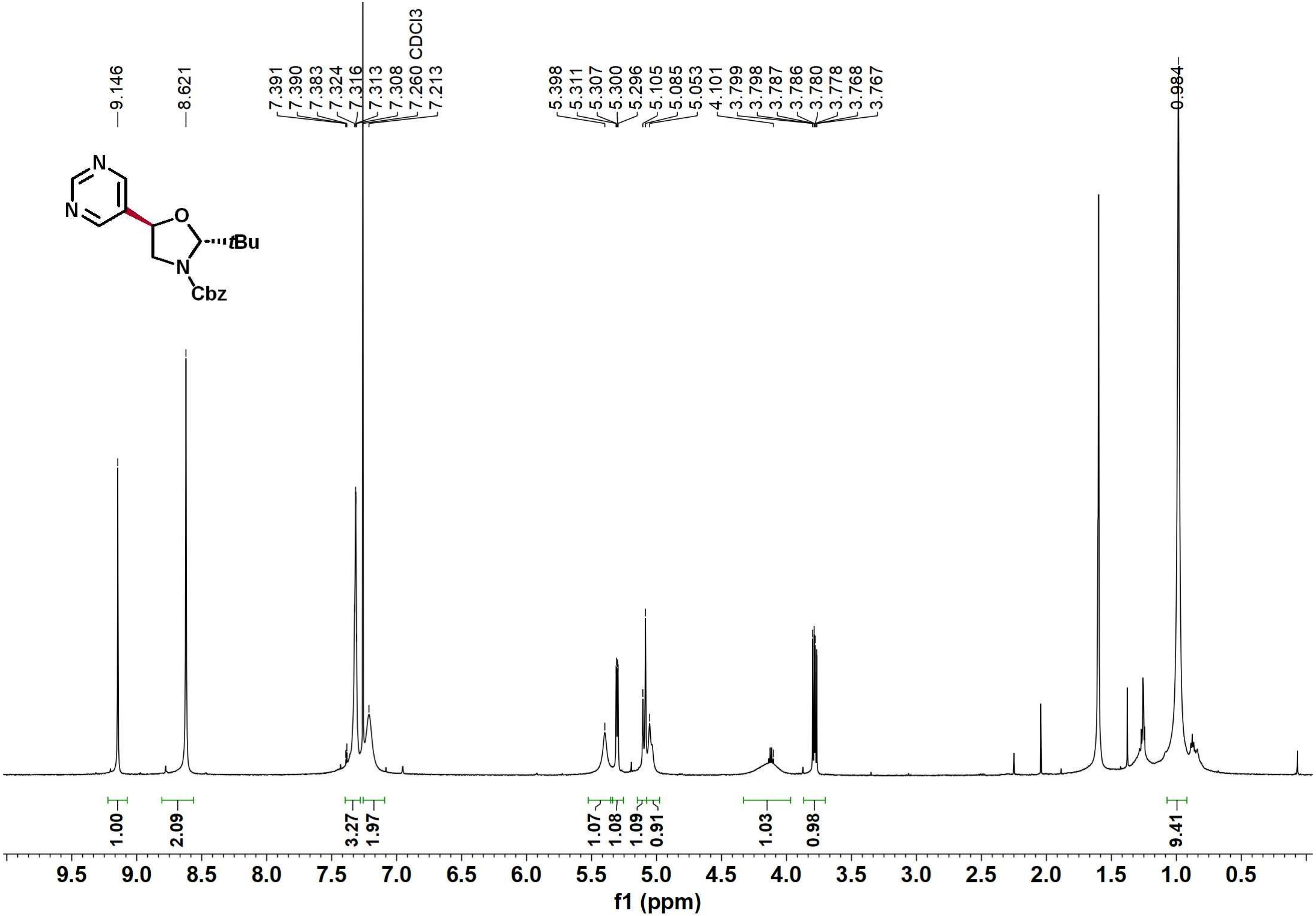

^{1}H NMR of Compound 17l (600 MHz, $CDCl_3$)

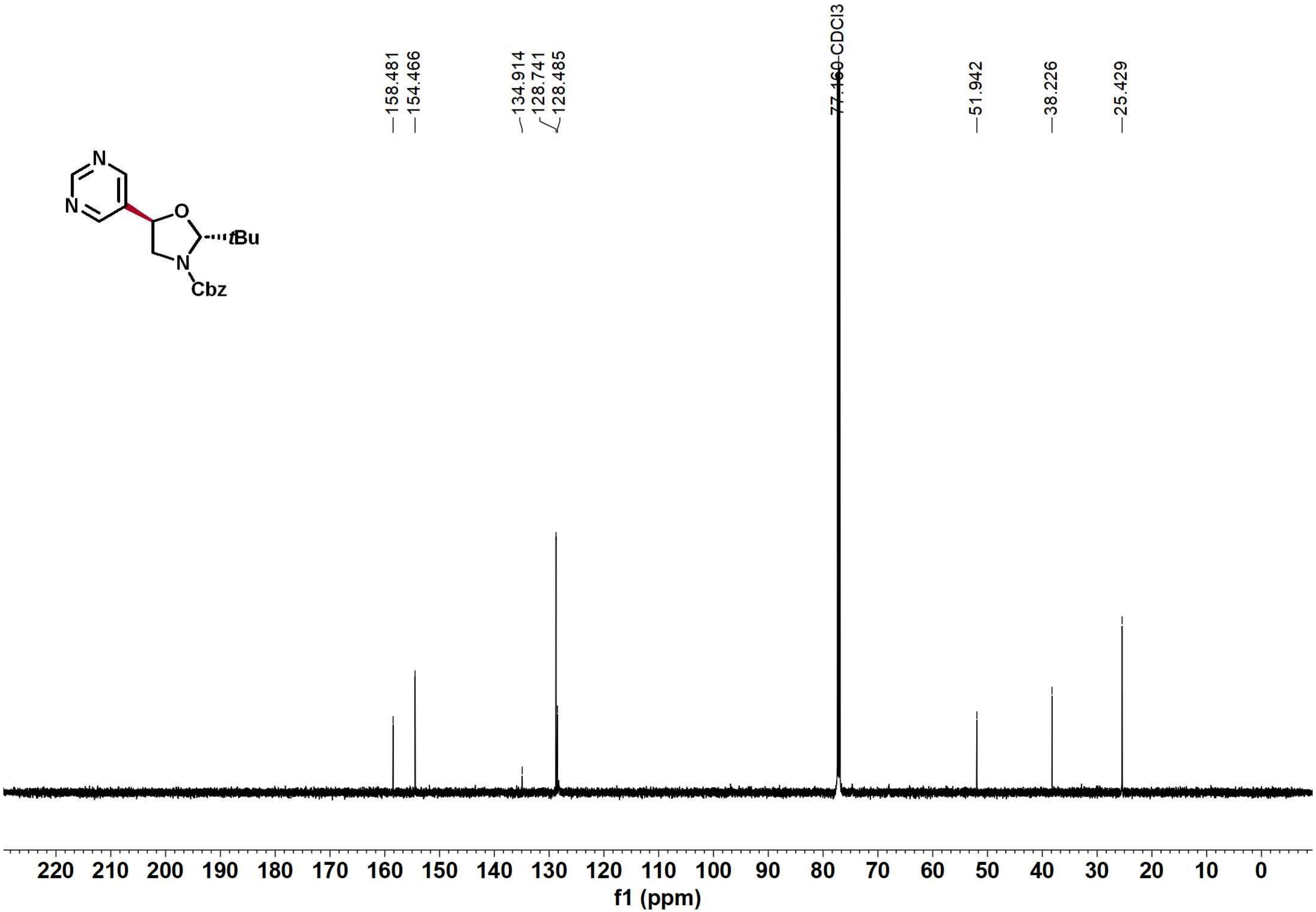

^{13}C NMR of Compound 17l (151 MHz, $CDCl_3$)

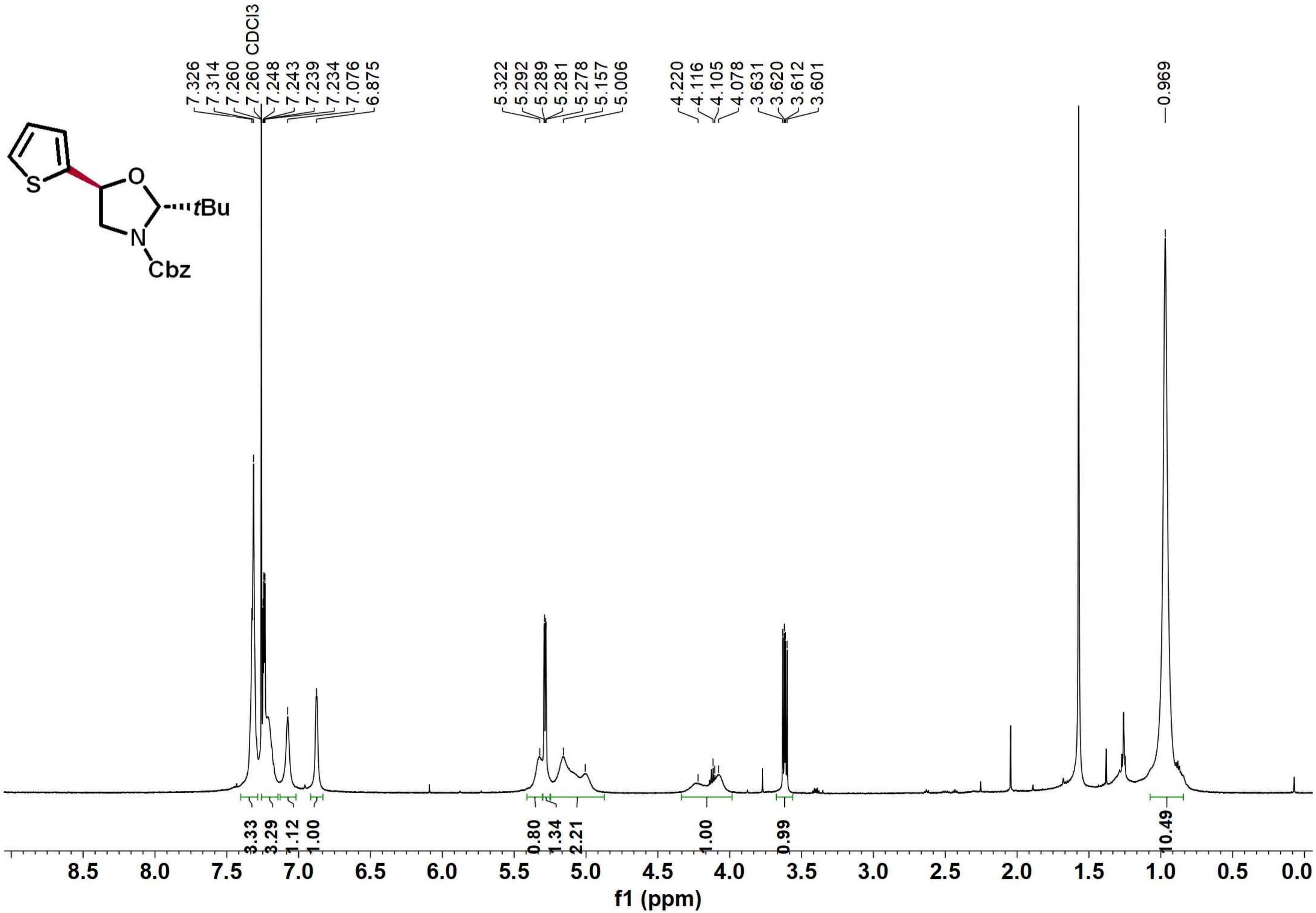

^{1}H NMR of Compound 17m (600 MHz, $CDCl_3$)

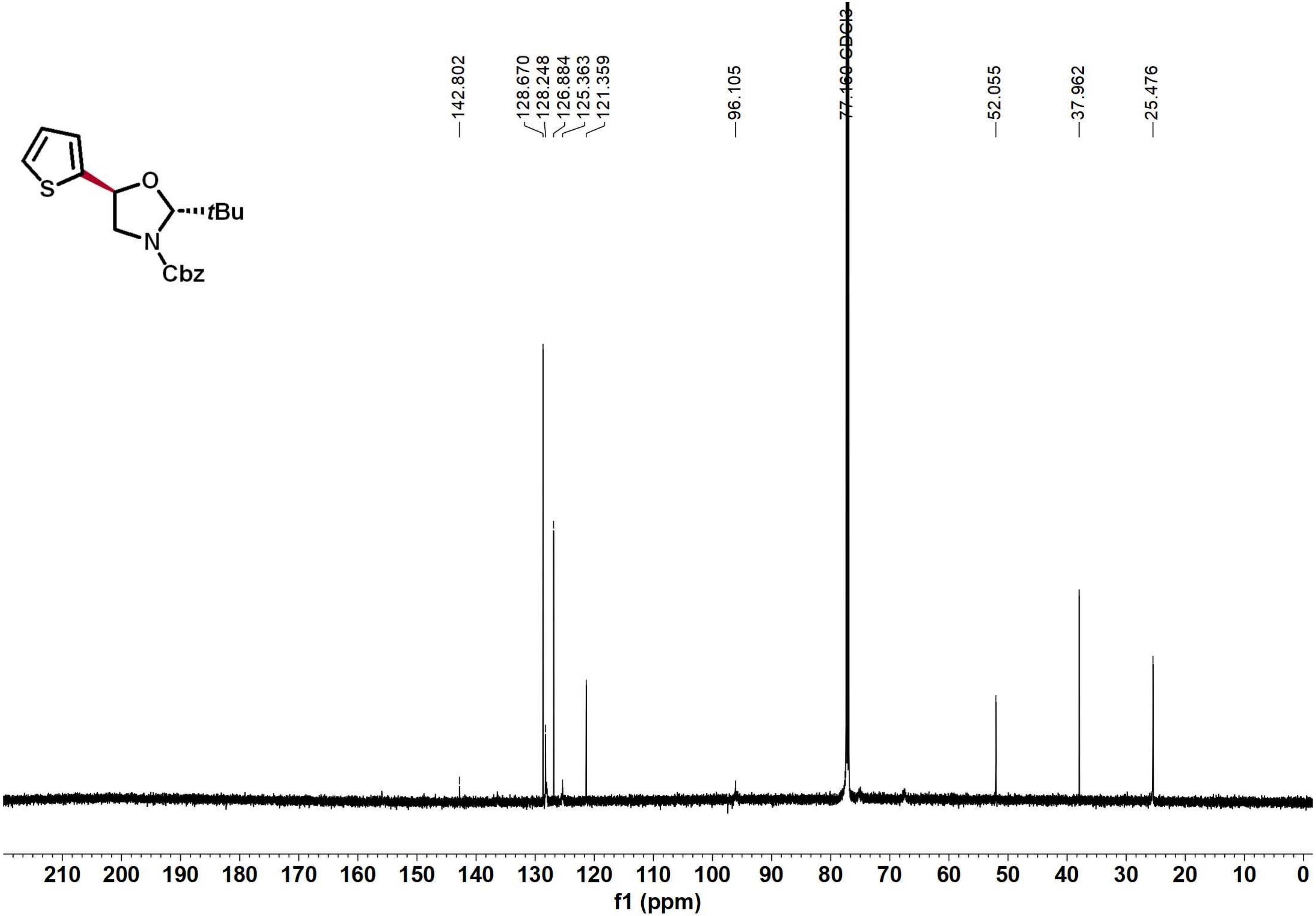

^{13}C NMR of Compound 17m (151 MHz, $CDCl_3$)

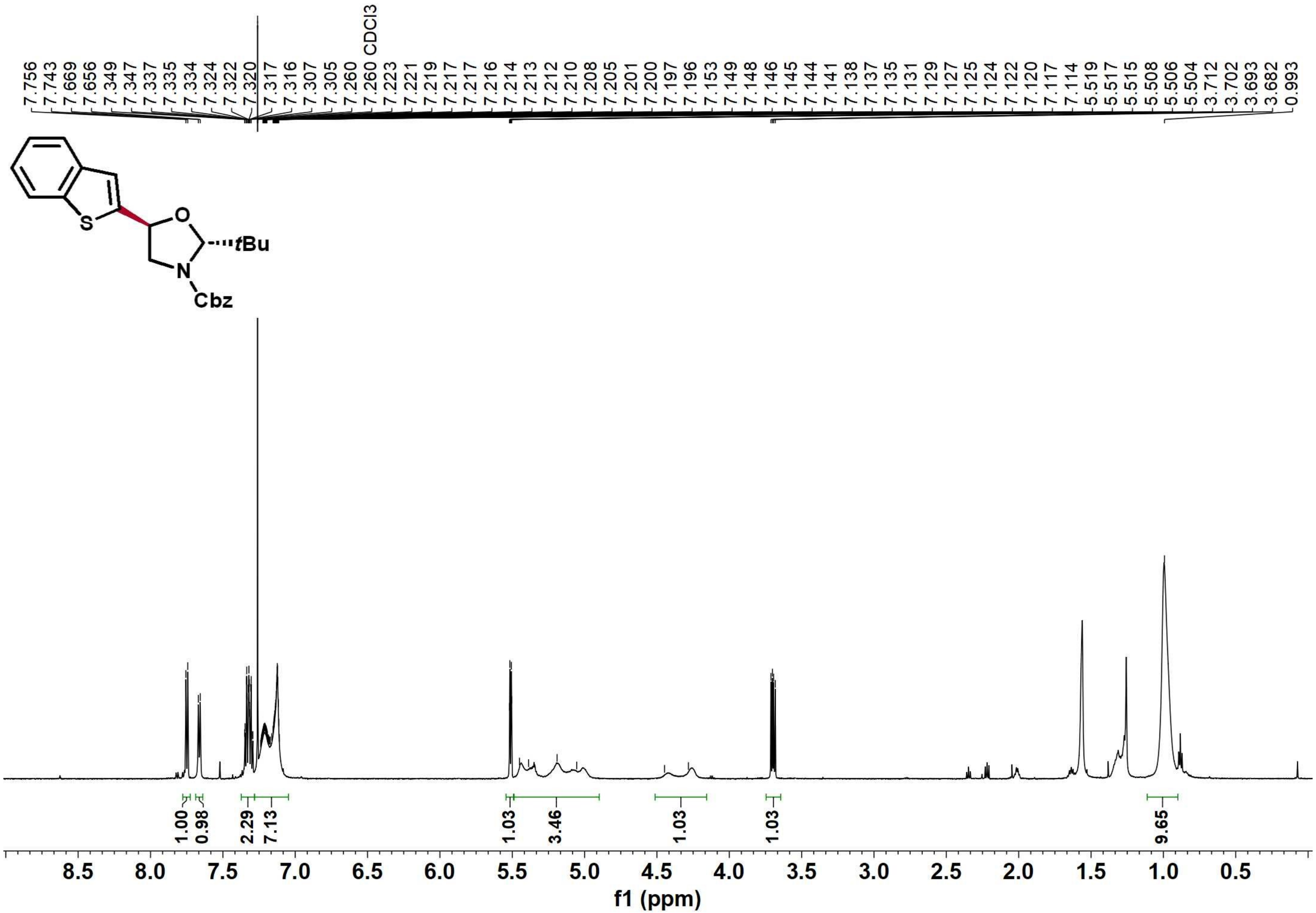

^{1}H NMR of Compound 17n (600 MHz, $CDCl_3$)

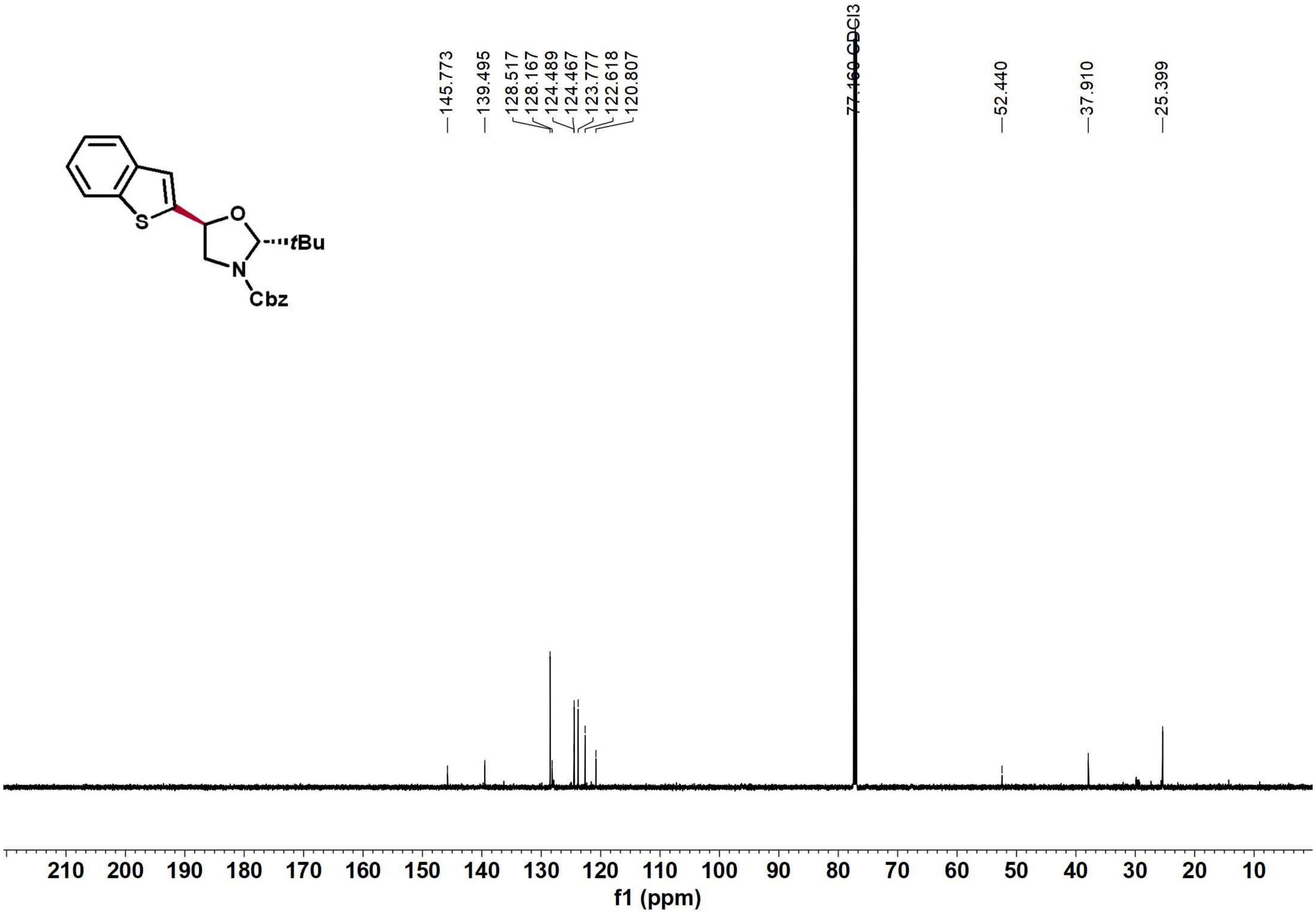

^{13}C NMR of Compound 17n (151 MHz, $CDCl_3$)

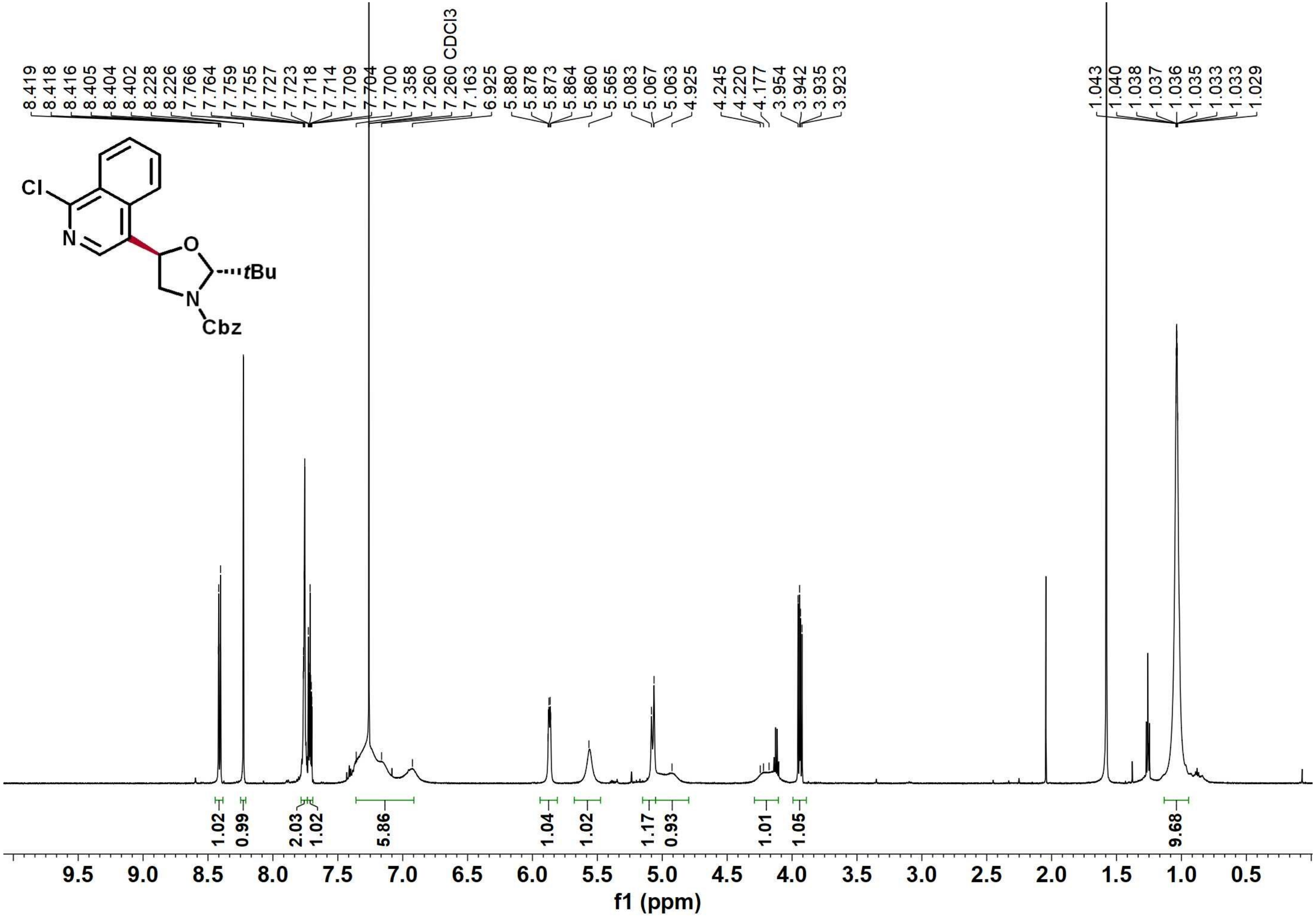

^{1}H NMR of Compound 17o (600 MHz, $CDCl_3$)

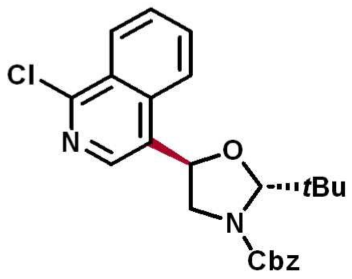

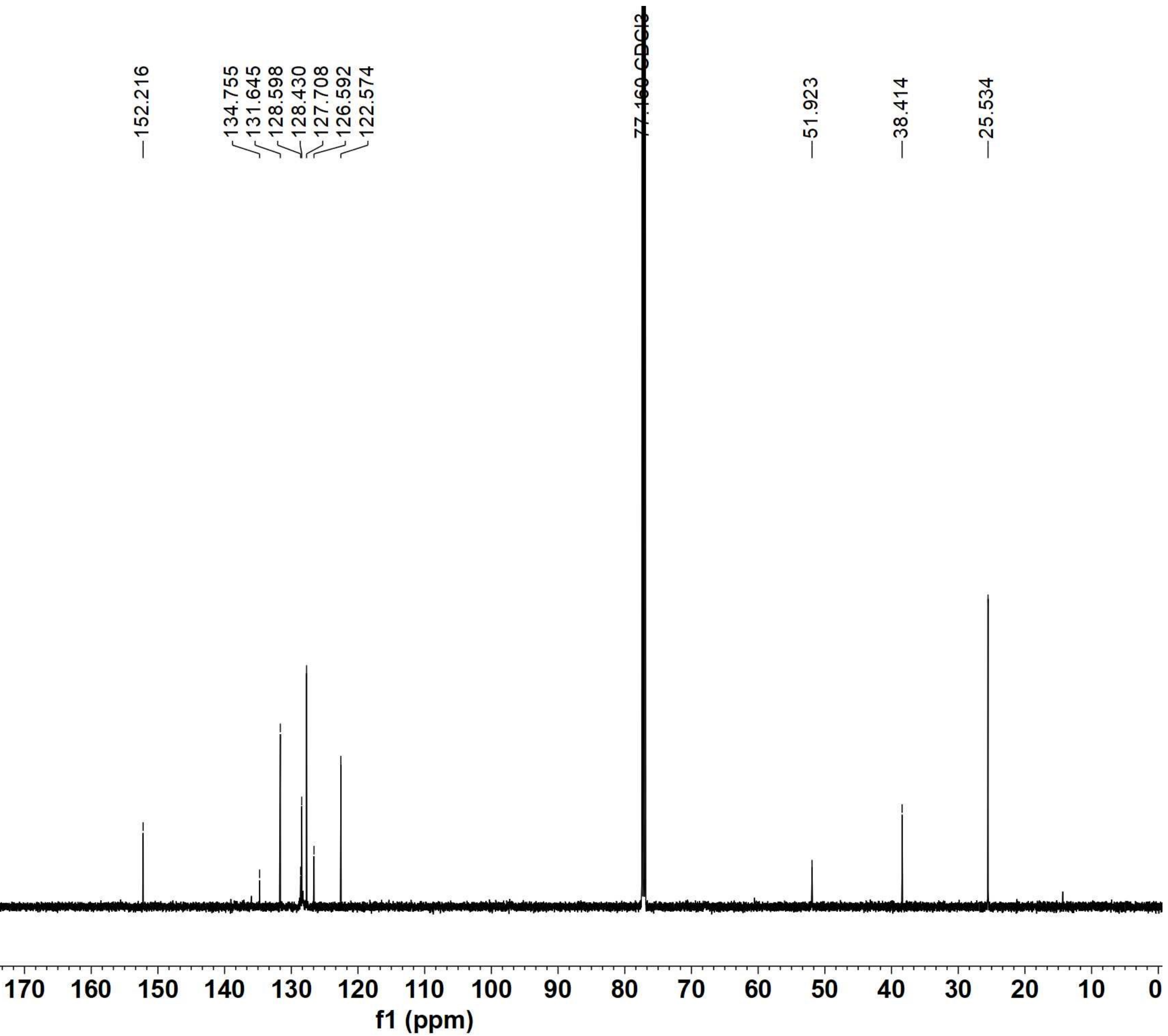

^{13}C NMR of Compound 17o (151 MHz, $CDCl_3$)

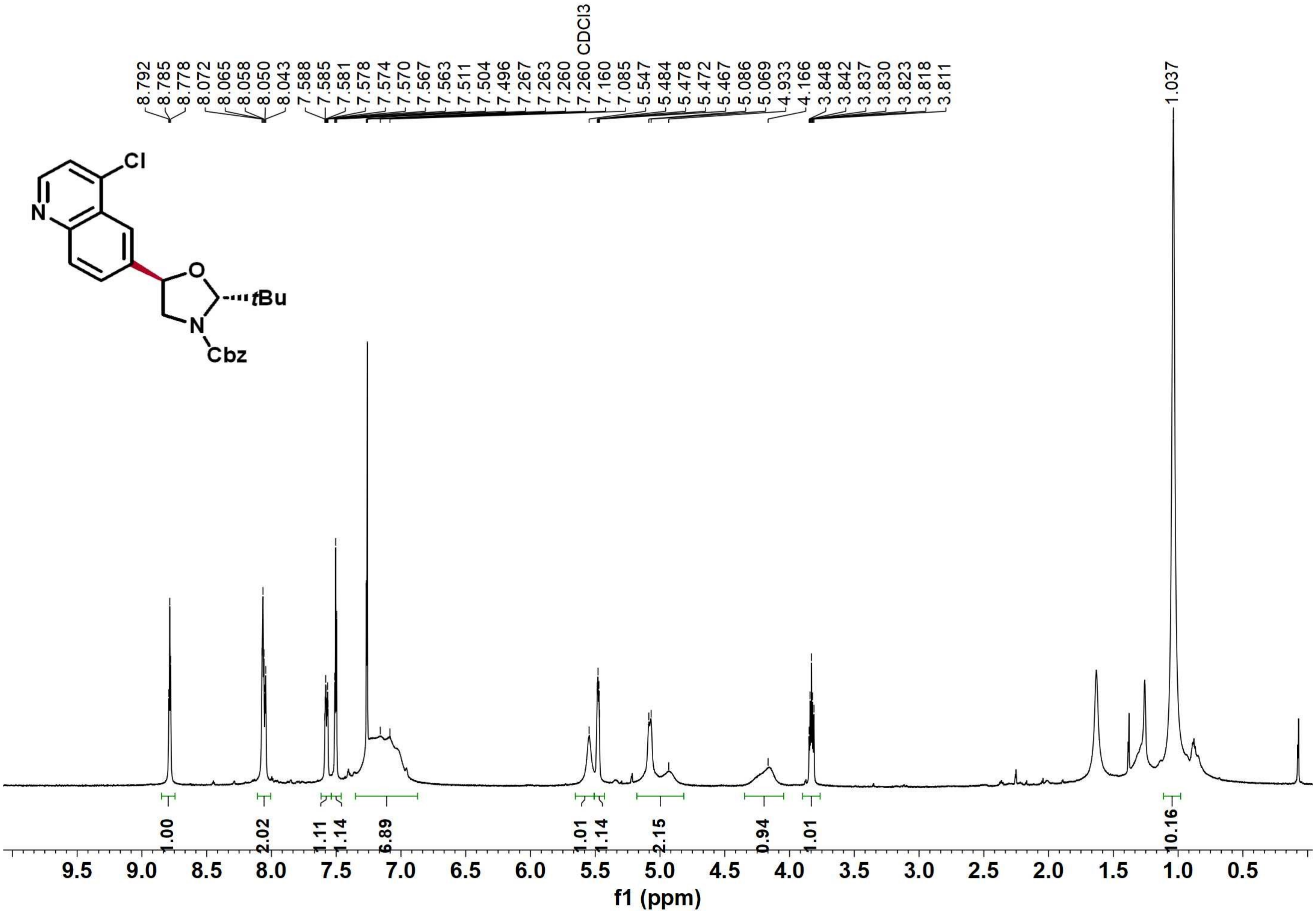

^{1}H NMR of Compound 17p (600 MHz, $CDCl_3$)

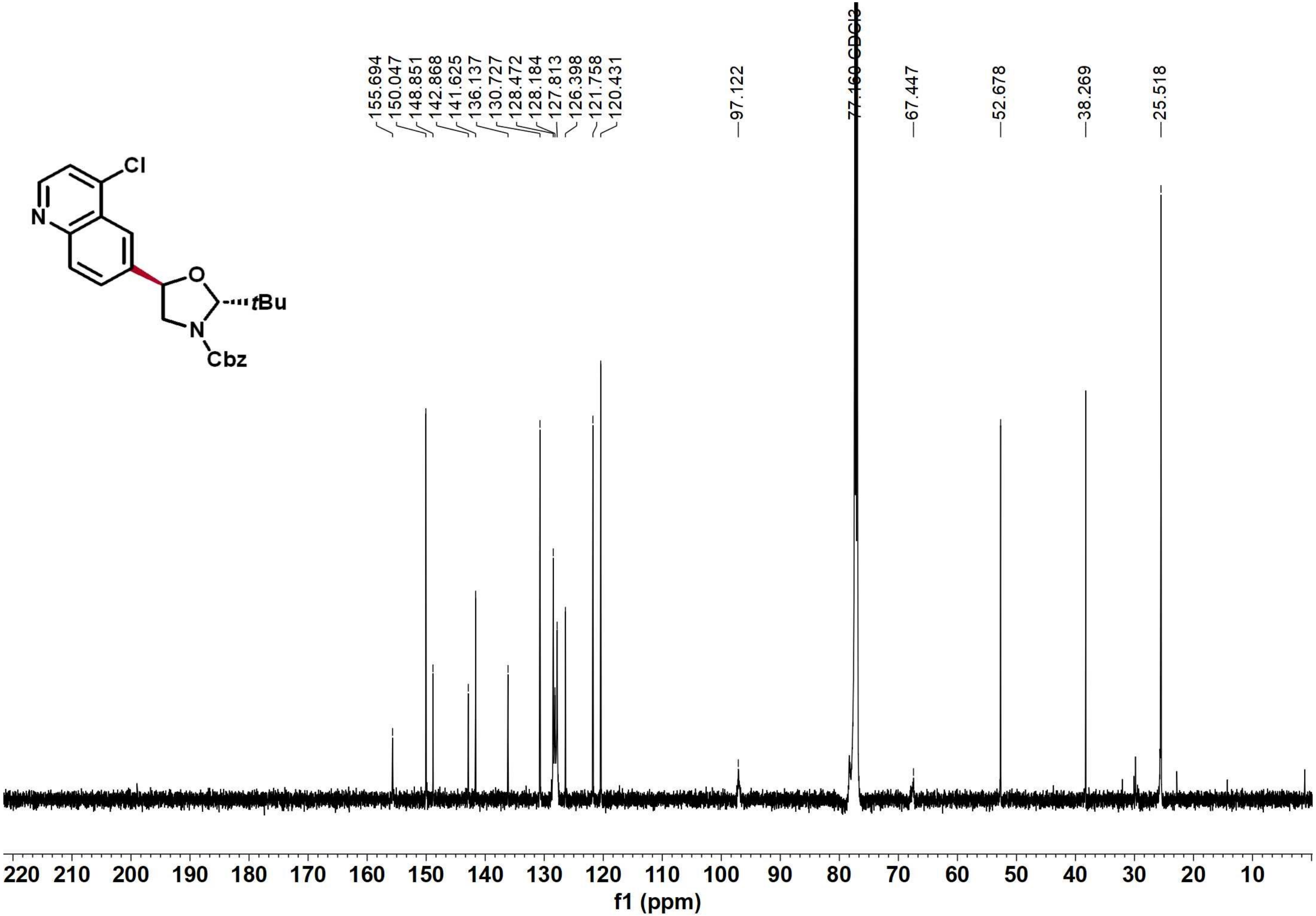

^{13}C NMR of Compound 17p (151 MHz, $CDCl_3$)

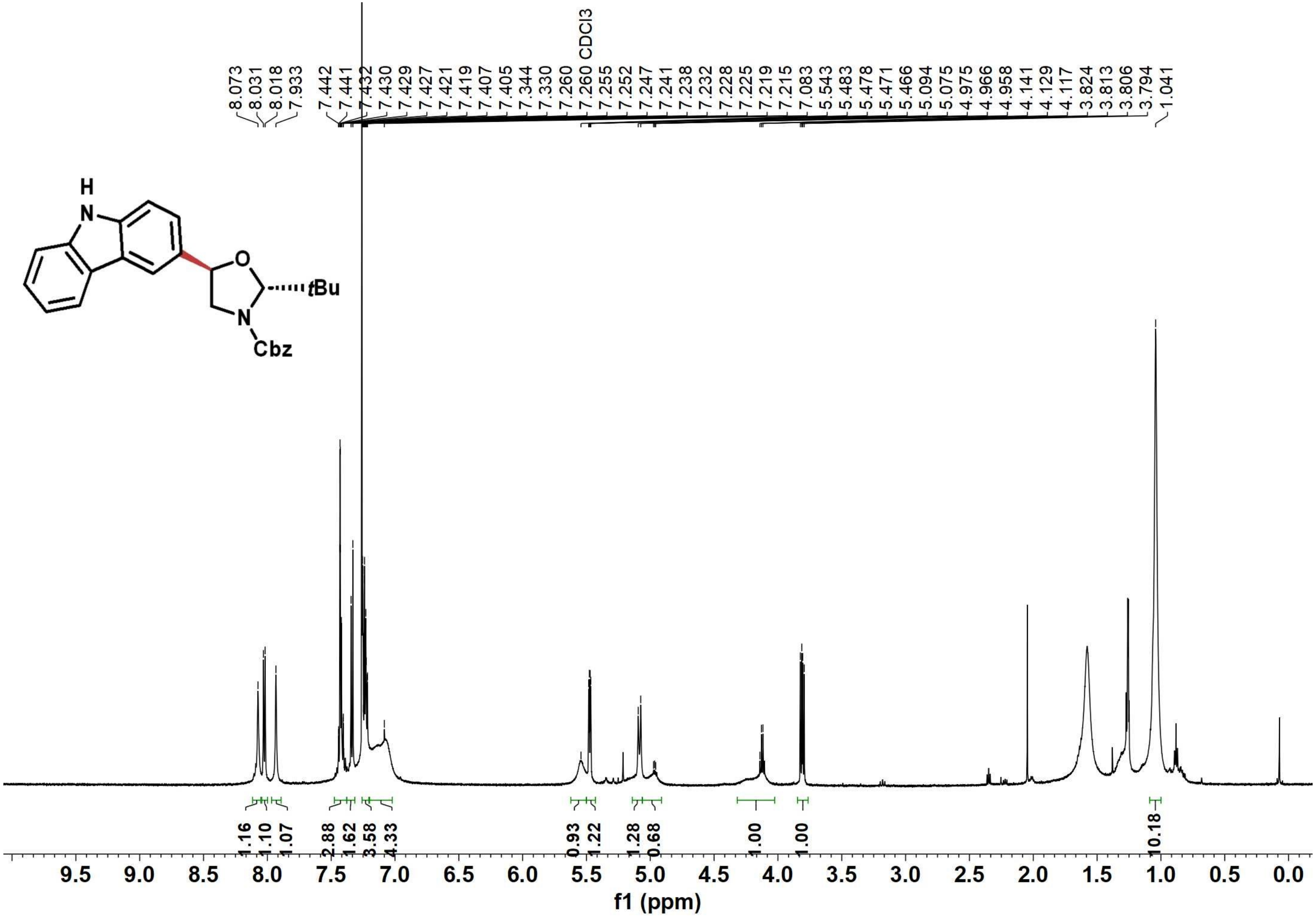

^{1}H NMR of Compound 17q (600 MHz, $CDCl_3$)

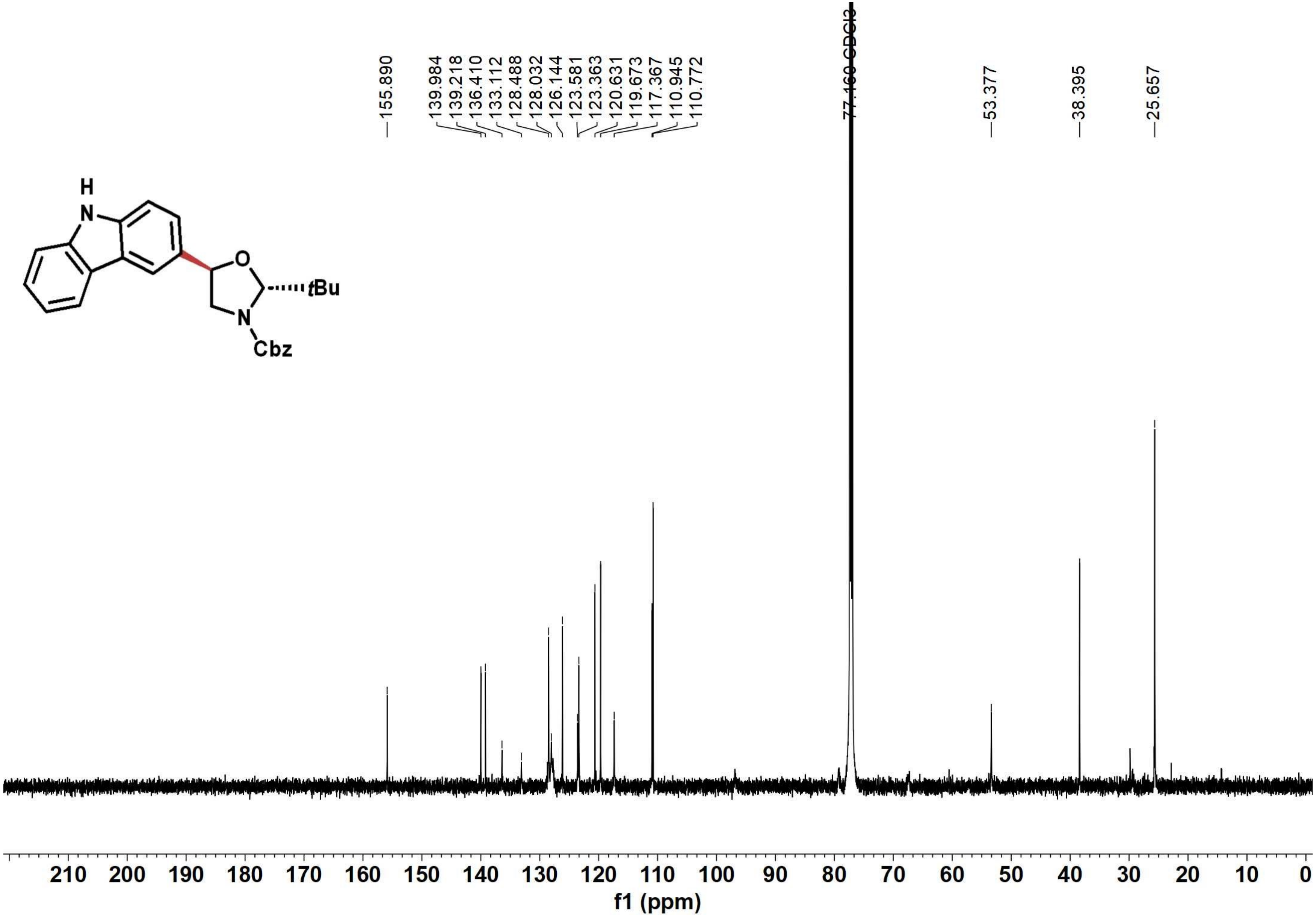

^{13}C NMR of Compound 17q (151 MHz, $CDCl_3$)

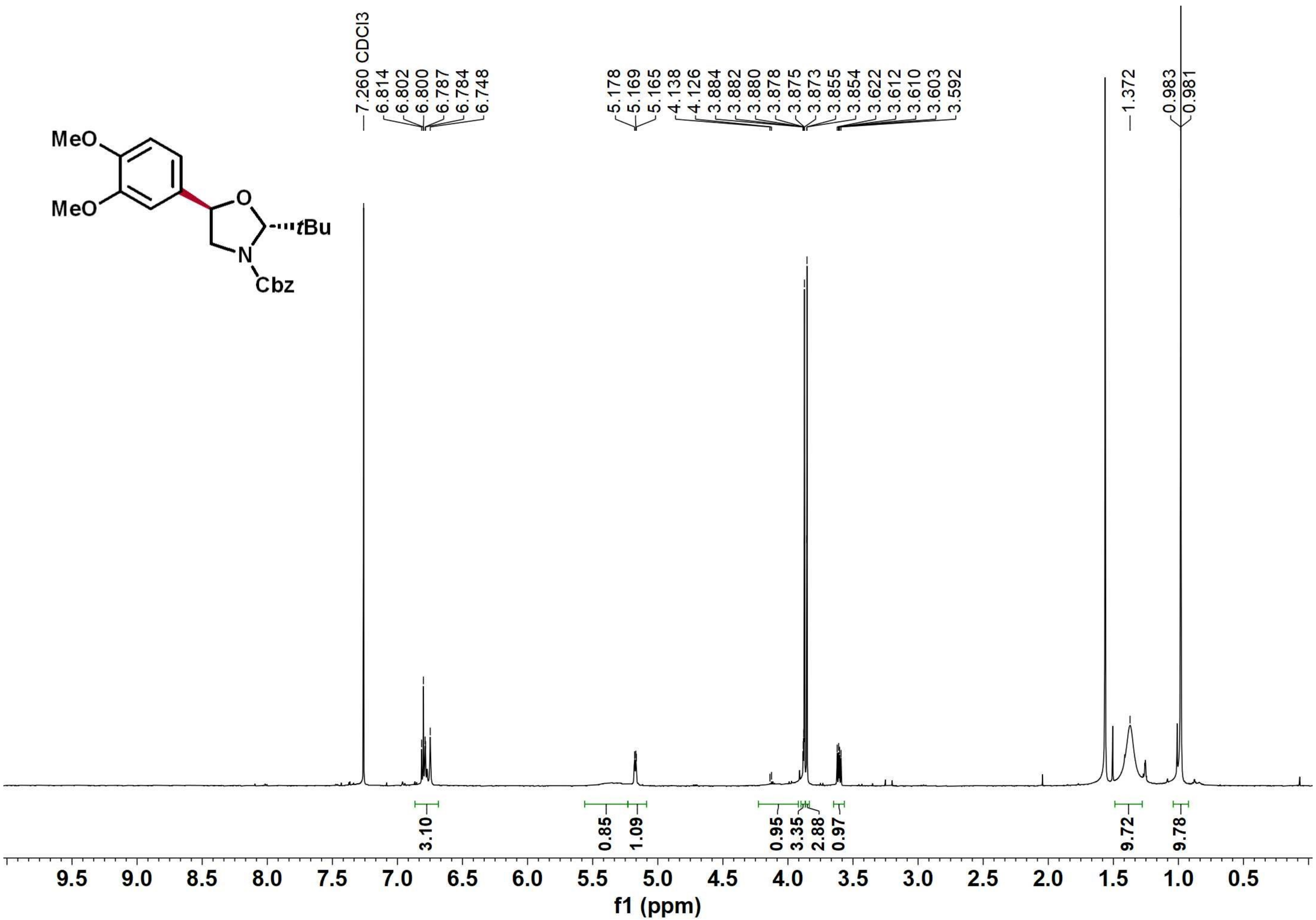

^{1}H NMR of Compound 18a (600 MHz, $CDCl_3$)

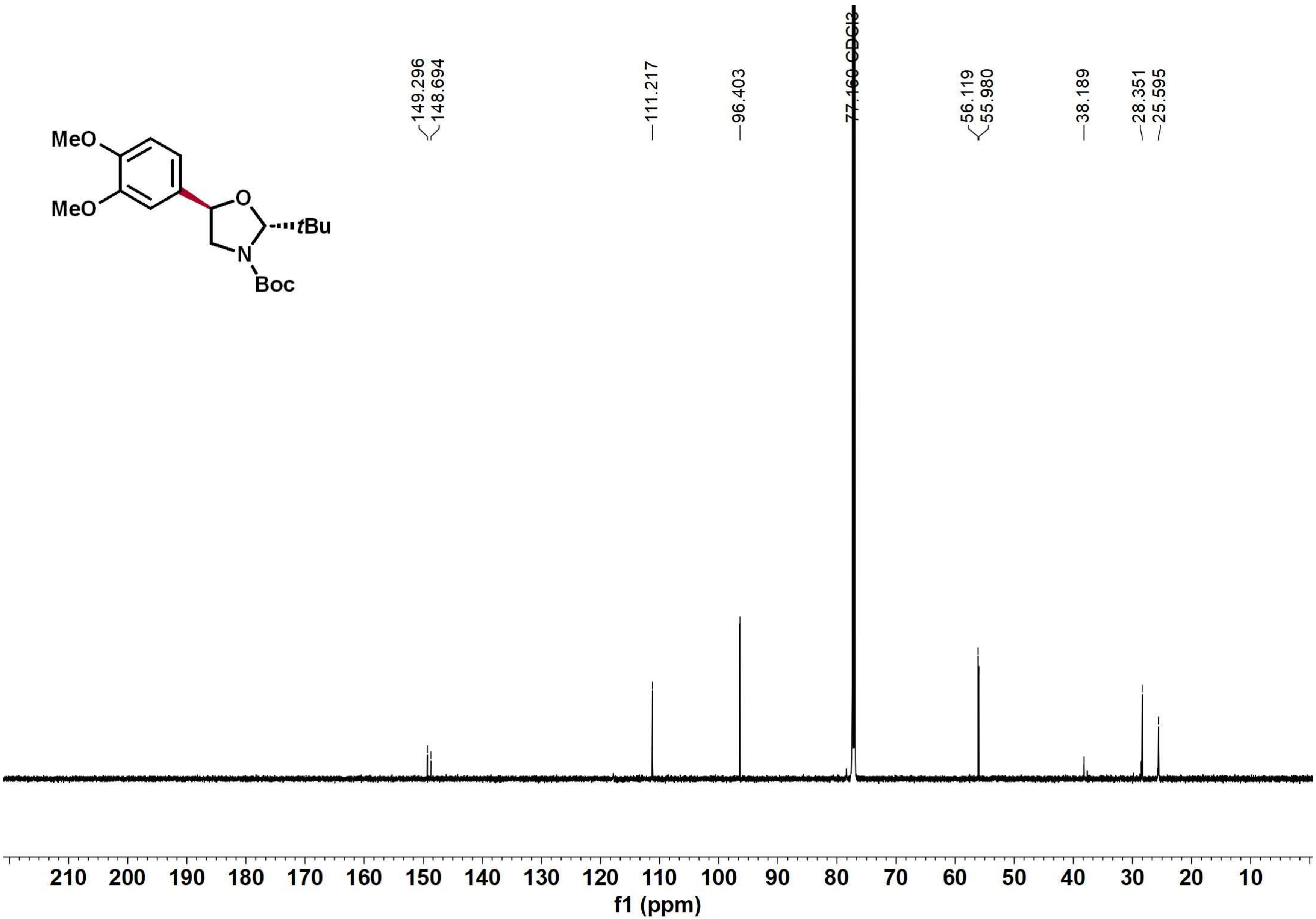

^{13}C NMR of Compound 18a (151 MHz, $CDCl_3$)

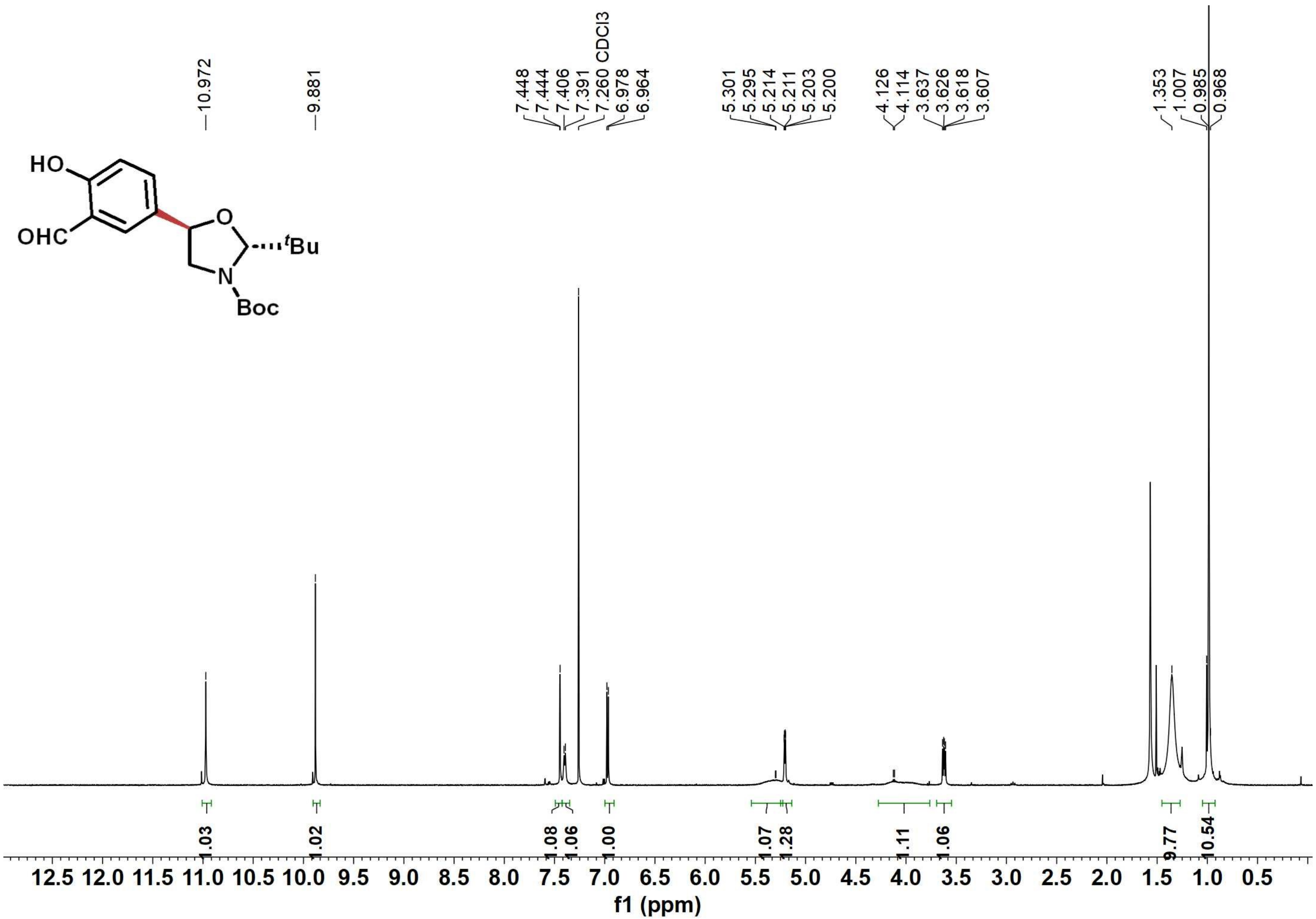

^{1}H NMR of Compound 18b (600 MHz, $CDCl_3$)

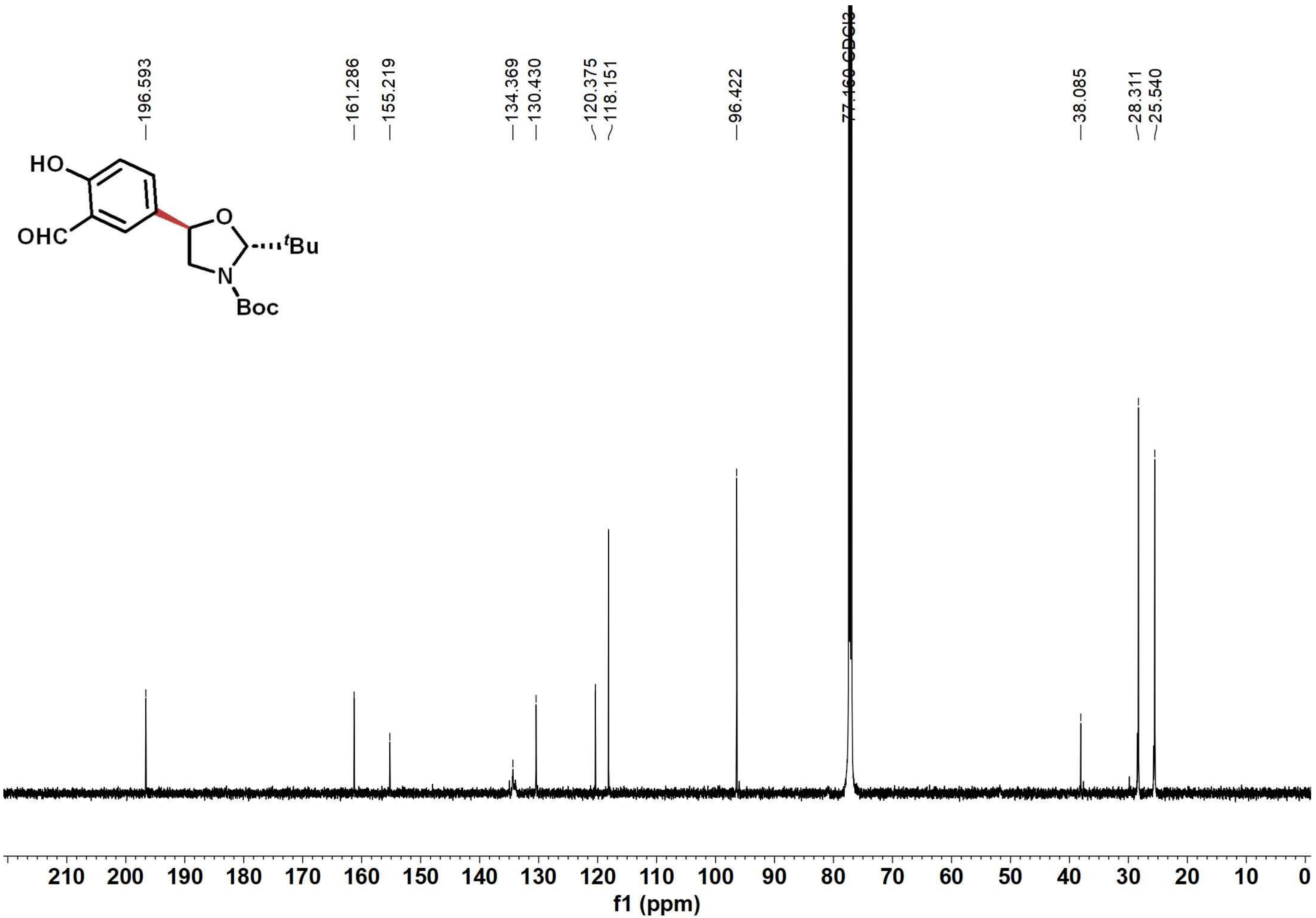

^{13}C NMR of Compound 18b (151 MHz, $CDCl_3$)

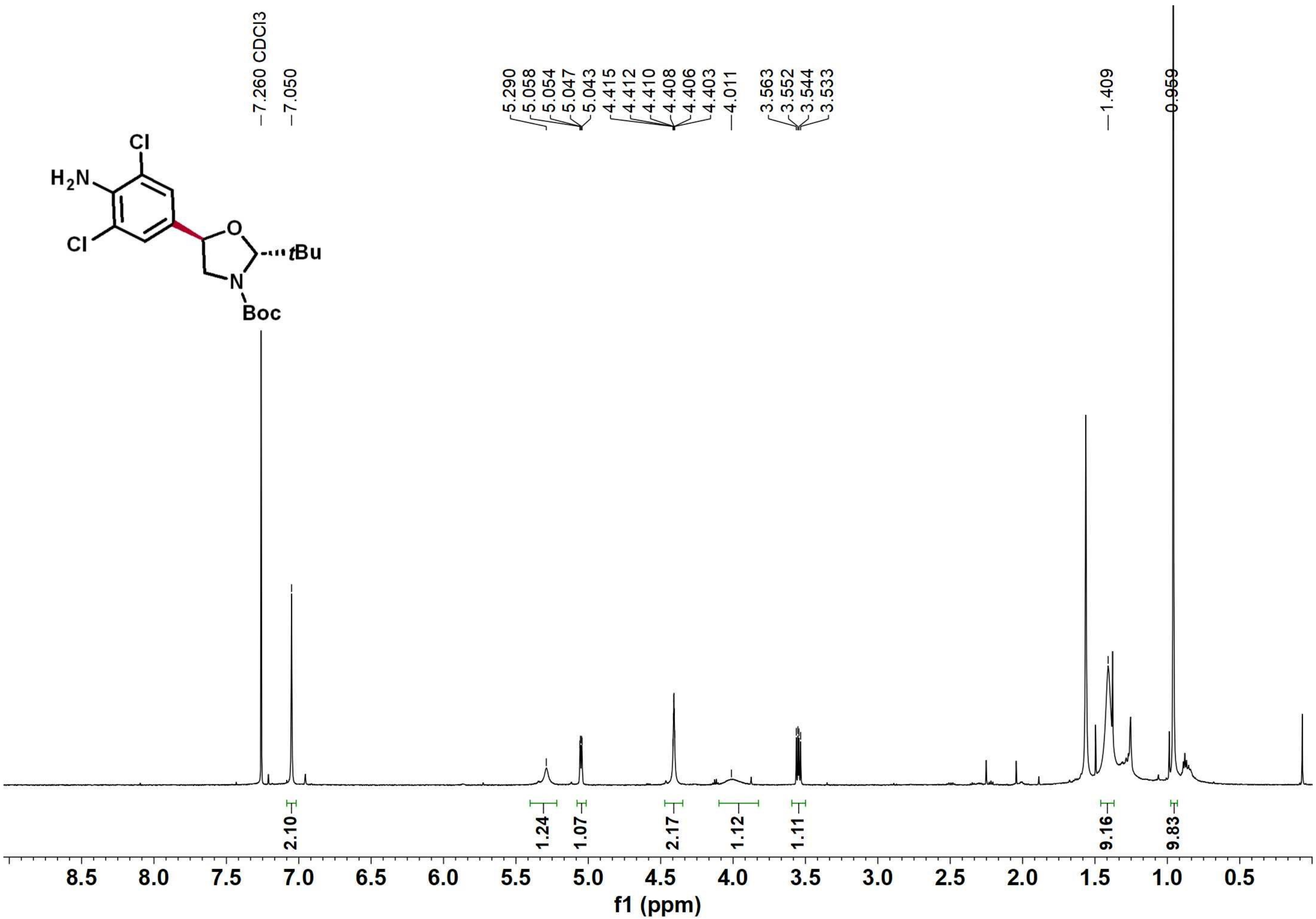

^{1}H NMR of Compound 18c (600 MHz, $CDCl_3$)

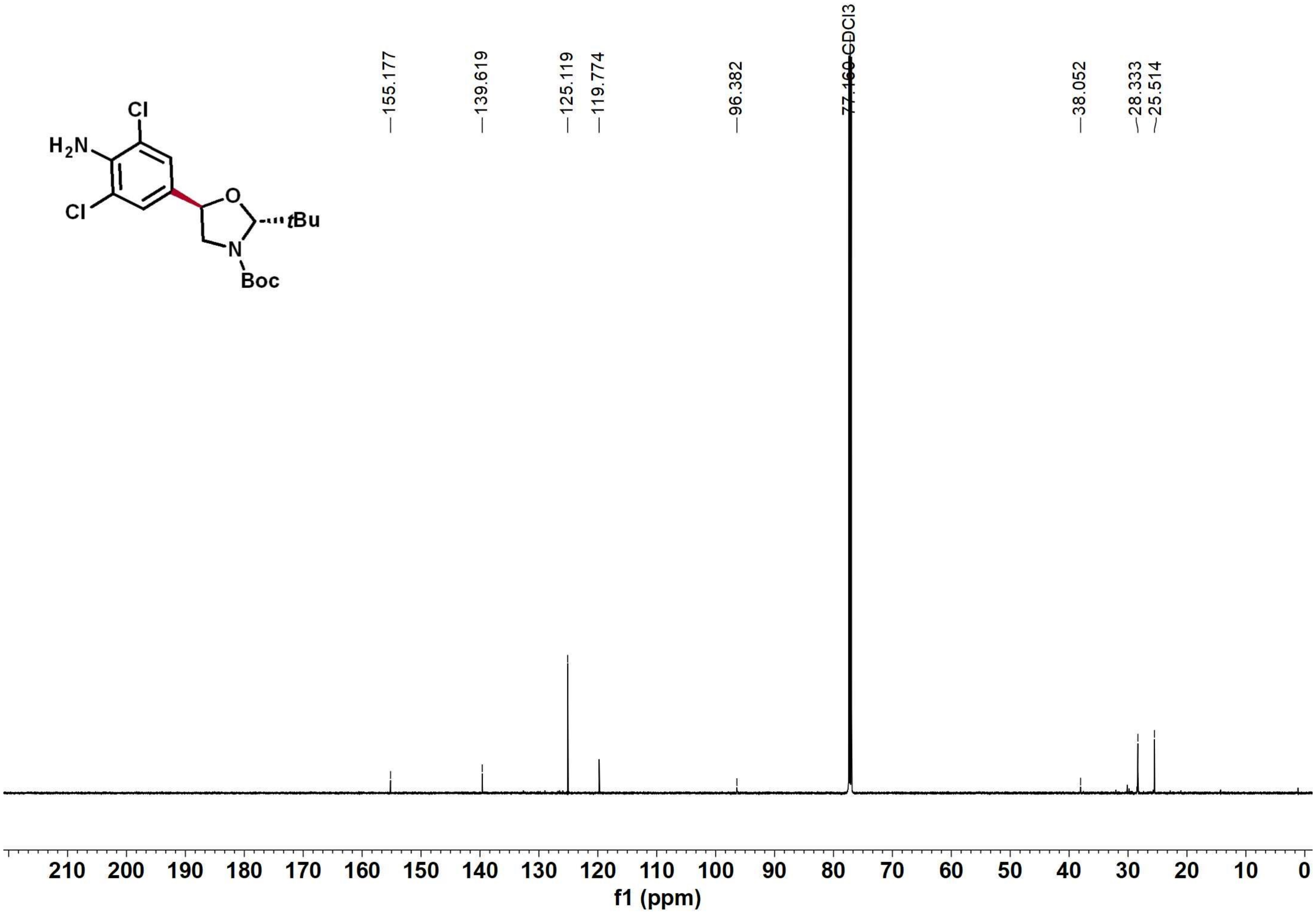

^{13}C NMR of Compound 18c (151 MHz, $CDCl_3$)

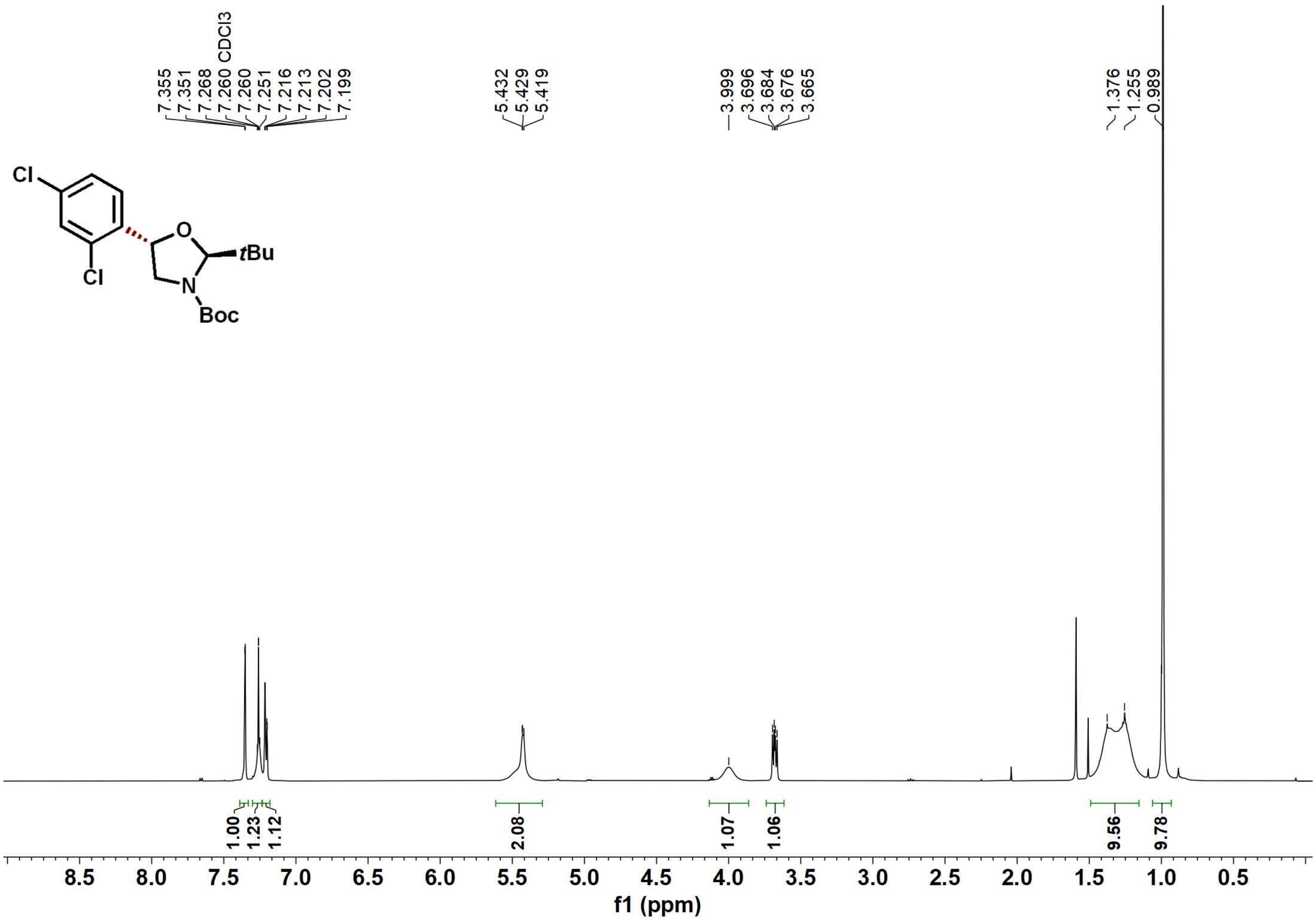

^{1}H NMR of Compound 18d (600 MHz, $CDCl_3$)

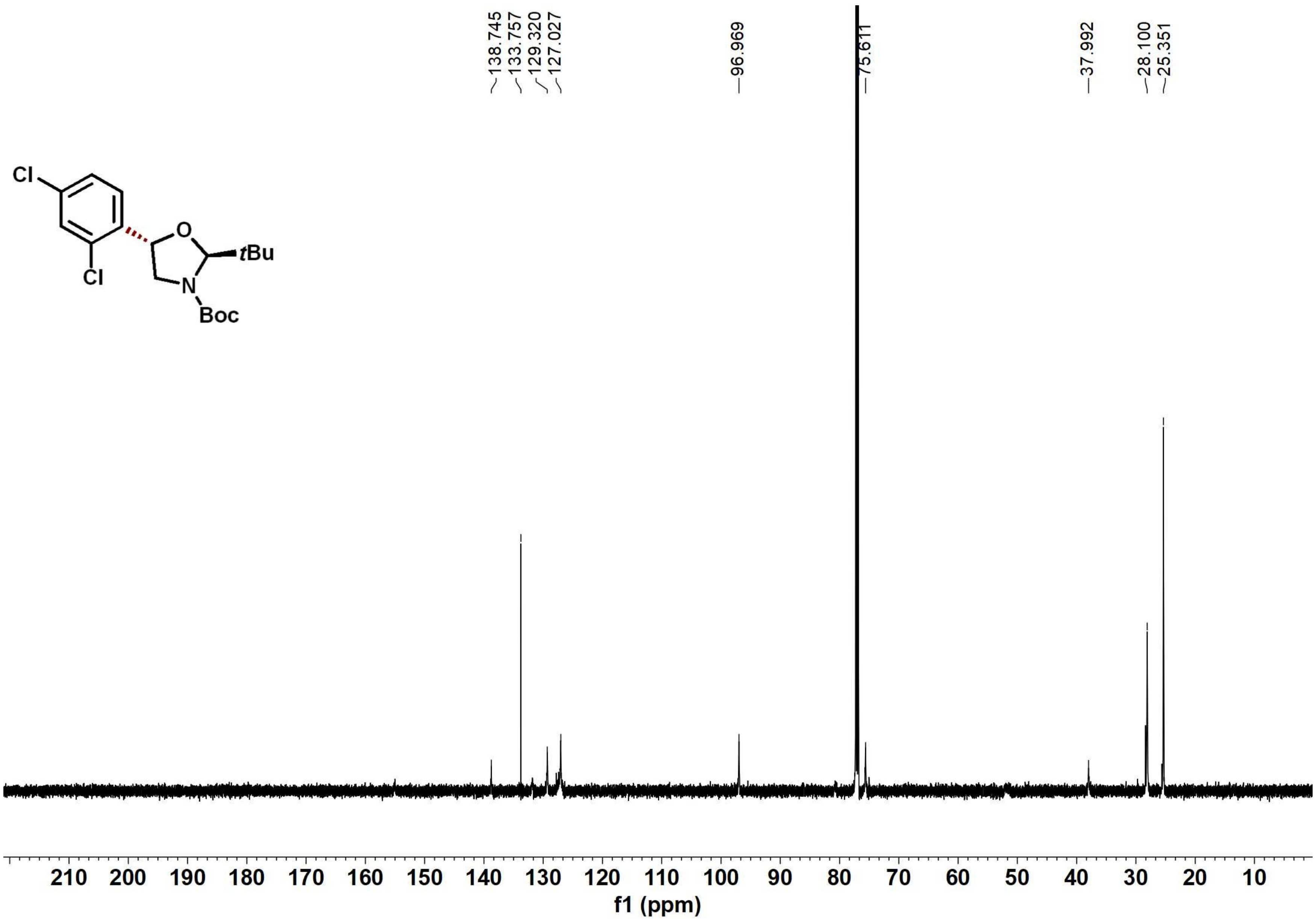

^{13}C NMR of Compound 18d (151 MHz, $CDCl_3$)

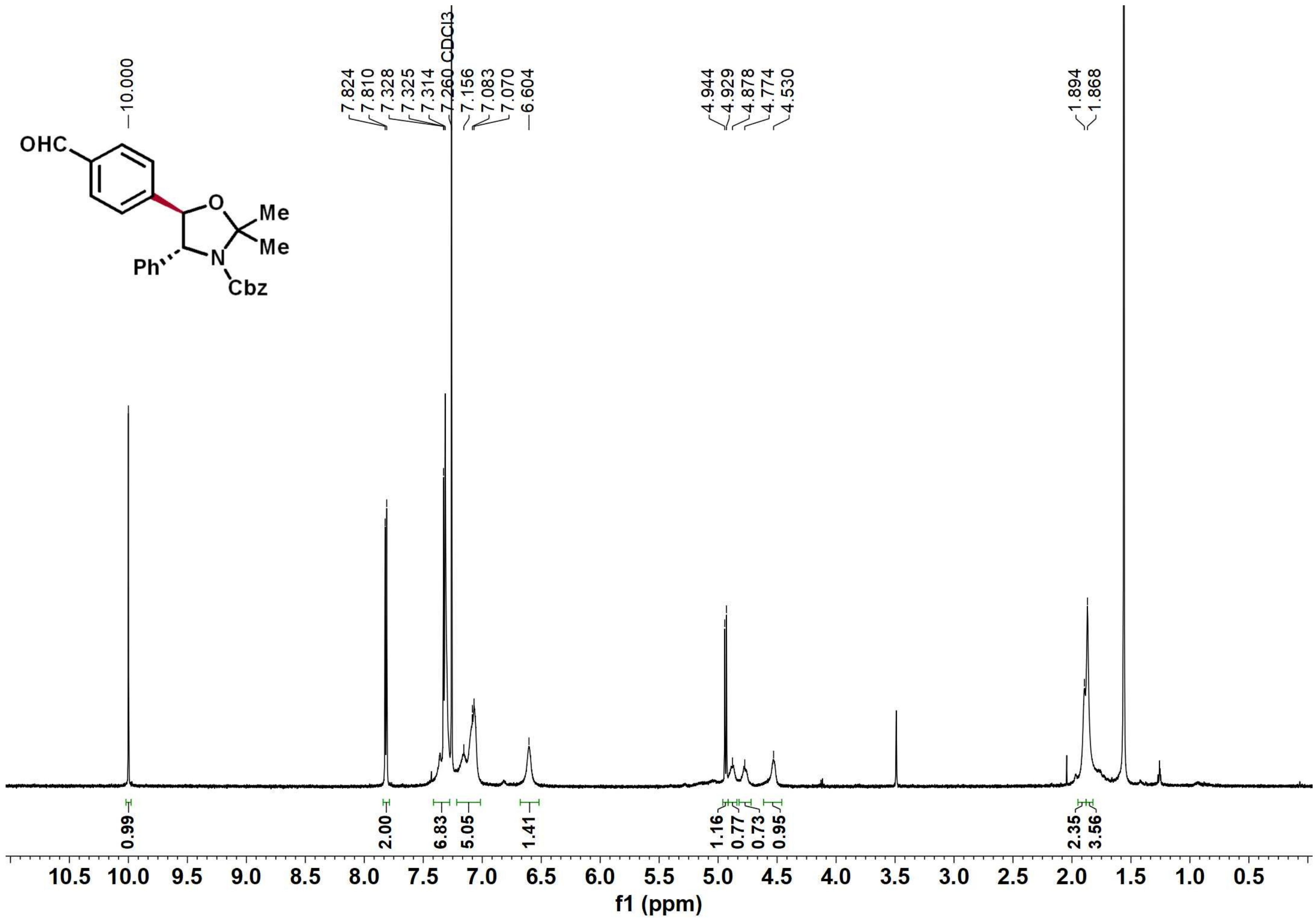

^{1}H NMR of Compound 19 (600 MHz, $CDCl_3$)

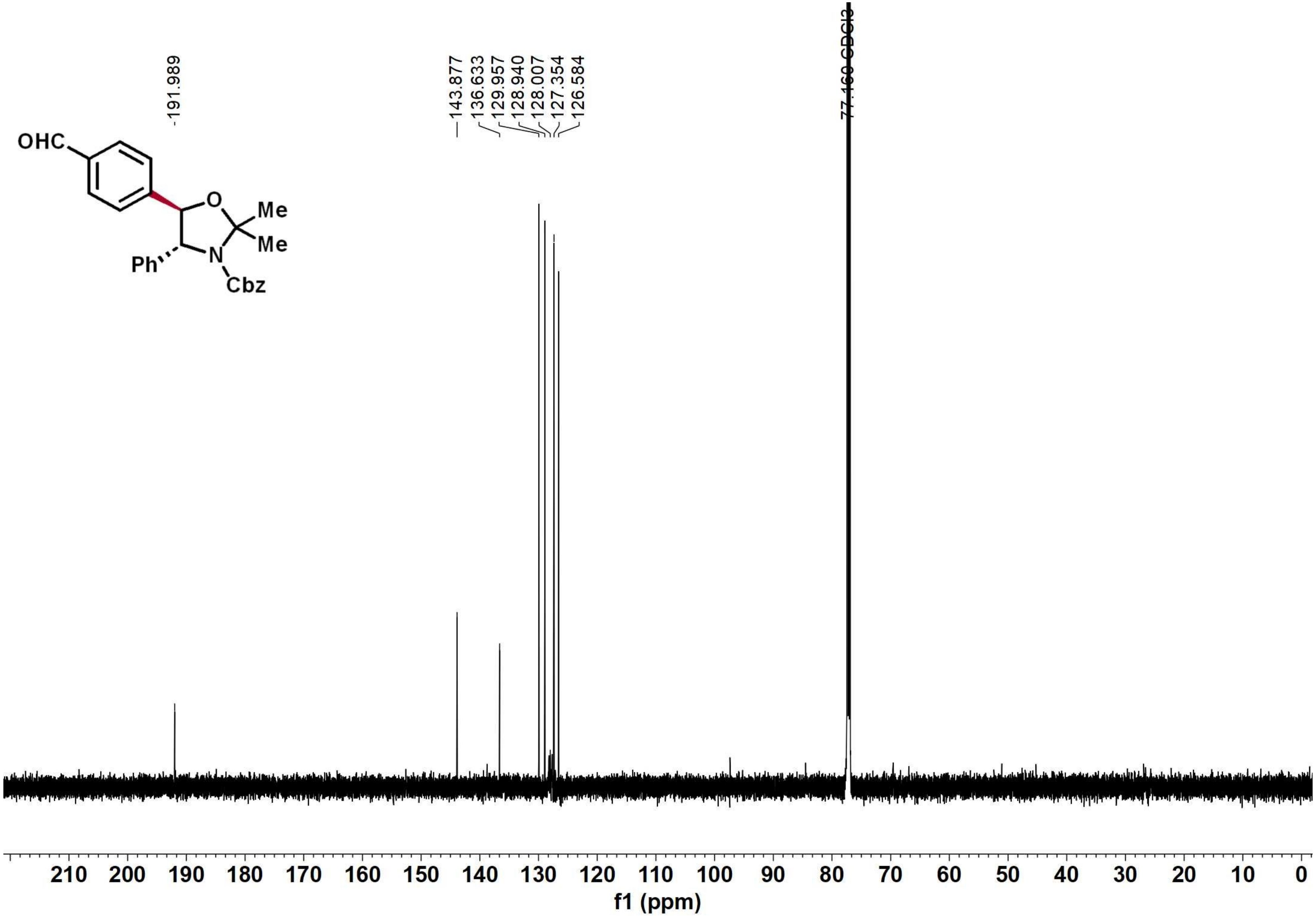

^{13}C NMR of Compound 19 (151 MHz, $CDCl_3$)

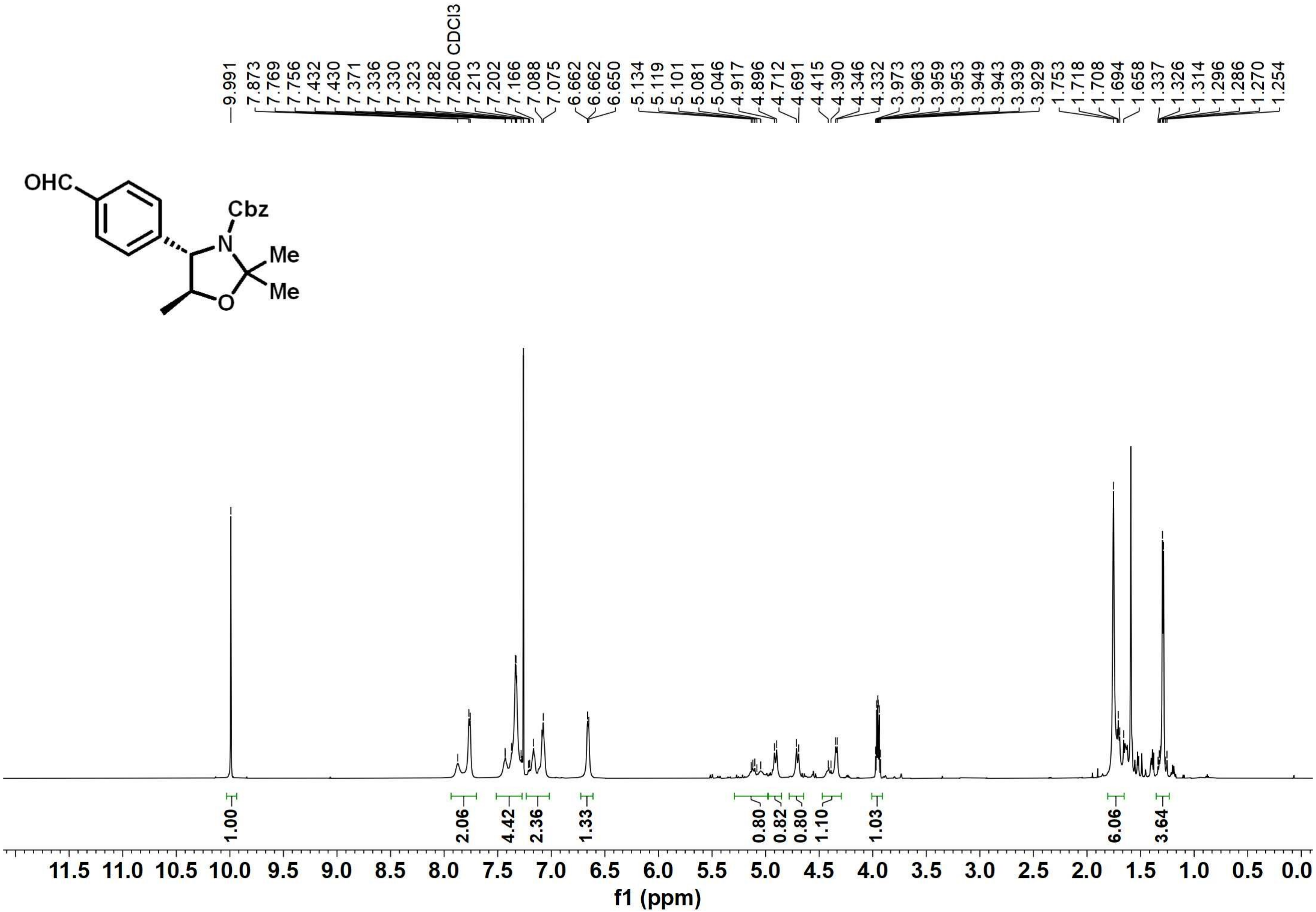

^{1}H NMR of Compound 20 (600 MHz, $CDCl_3$)

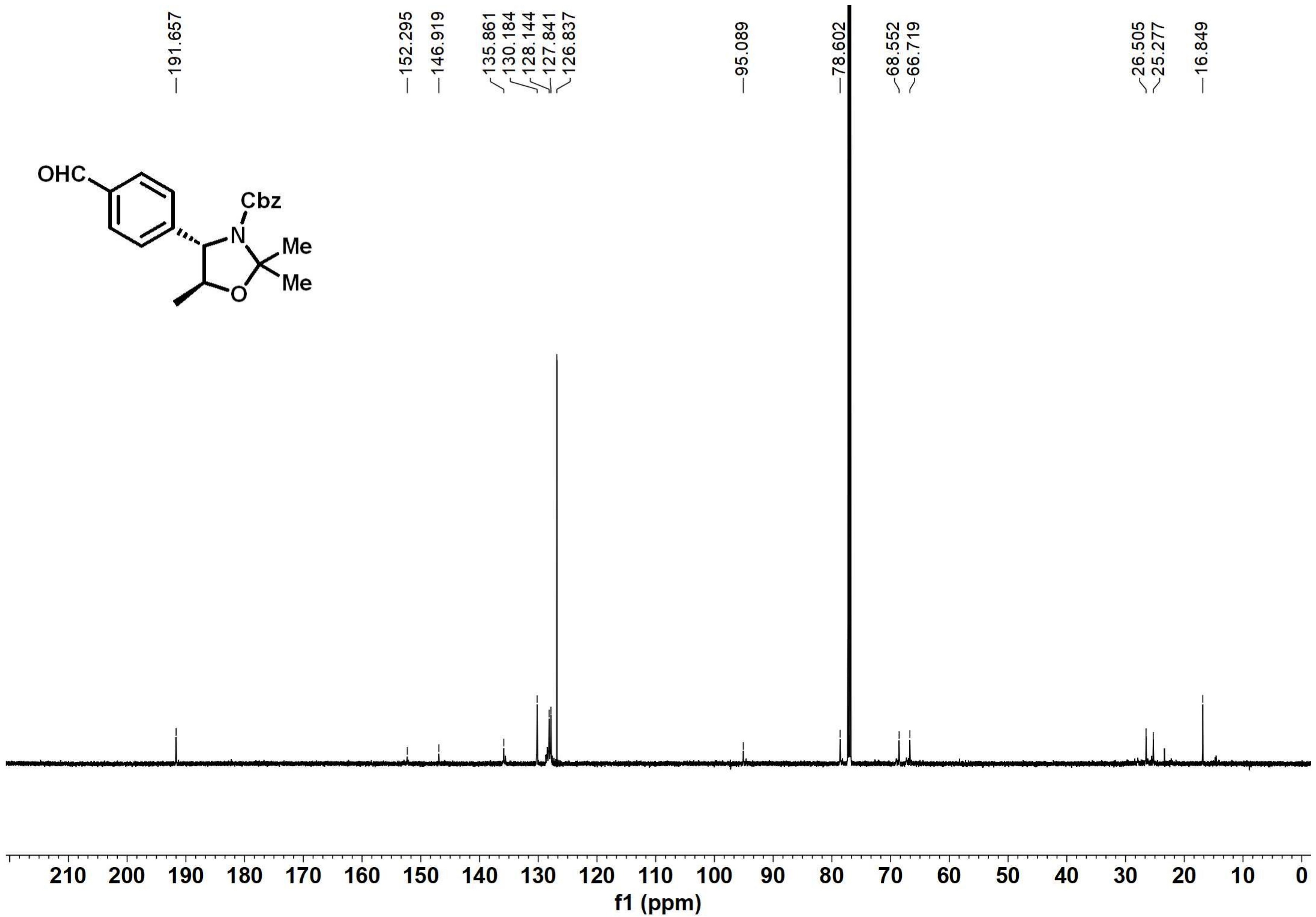

^{13}C NMR of Compound 20 (151 MHz, $CDCl_3$)

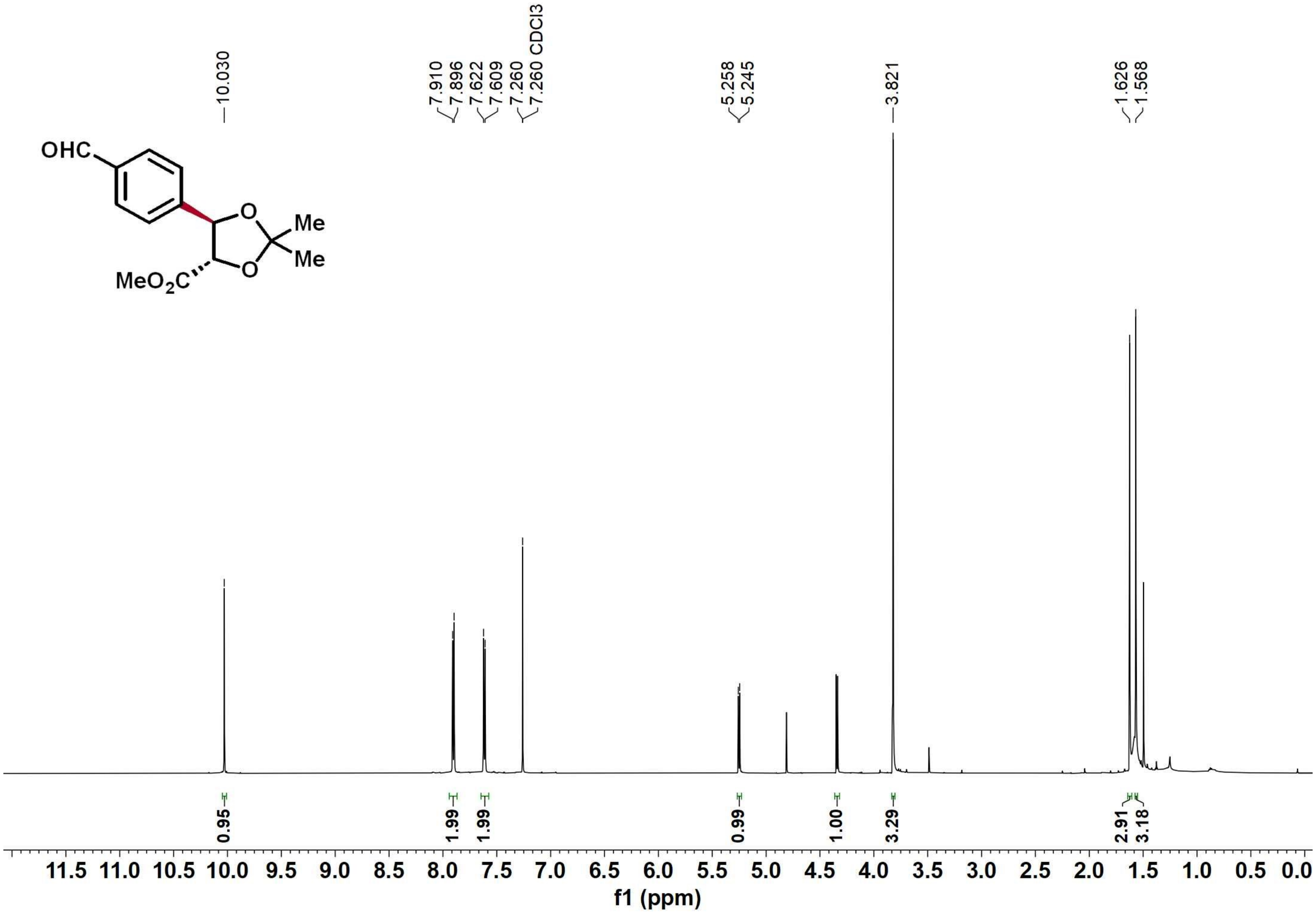

^{1}H NMR of Compound 21a (600 MHz, $CDCl_3$)

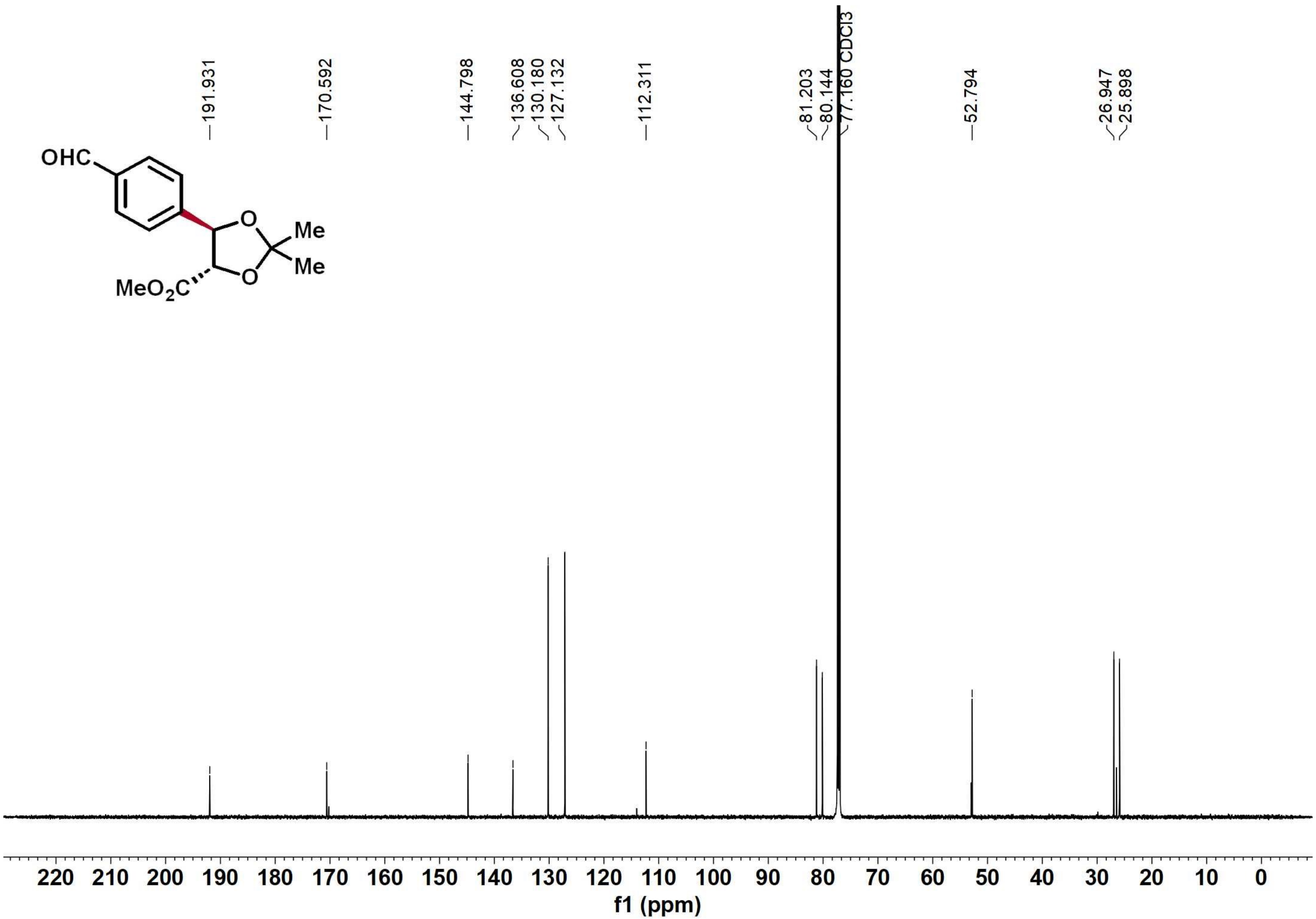

^{13}C NMR of Compound 21a (151 MHz, $CDCl_3$)

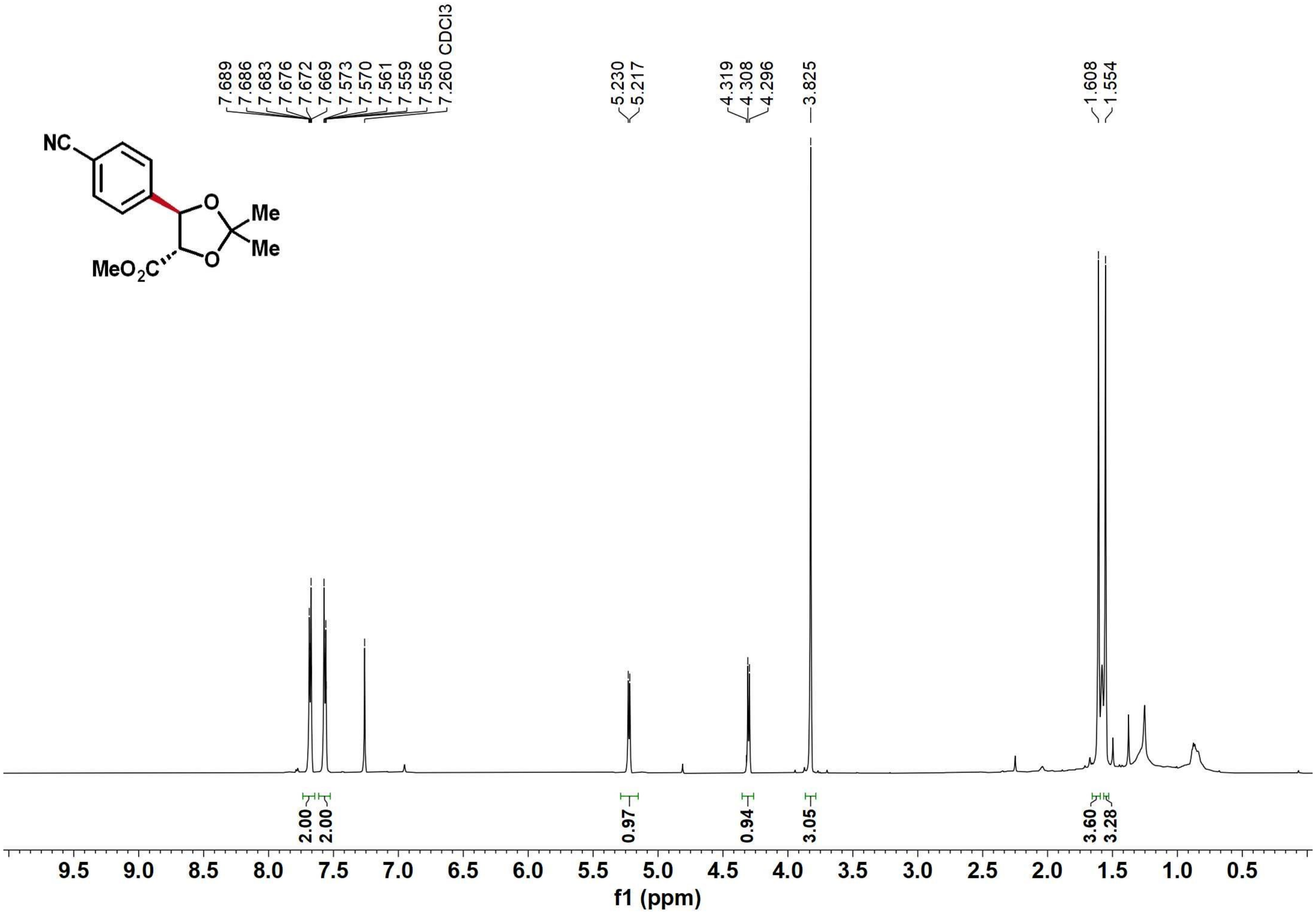

^{1}H NMR of Compound 21b (600 MHz, $CDCl_3$)

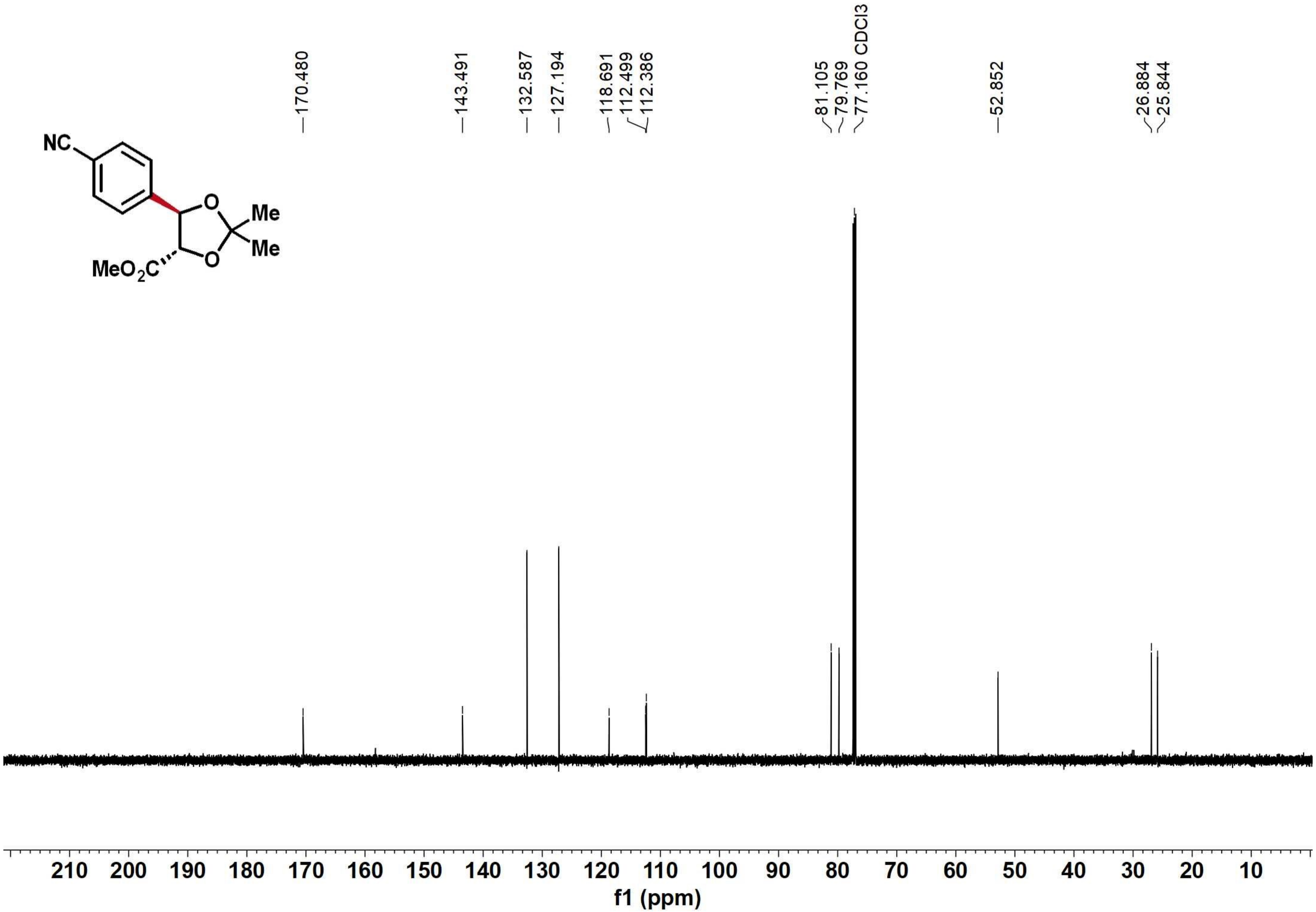

^{13}C NMR of Compound 21b (151 MHz, $CDCl_3$)

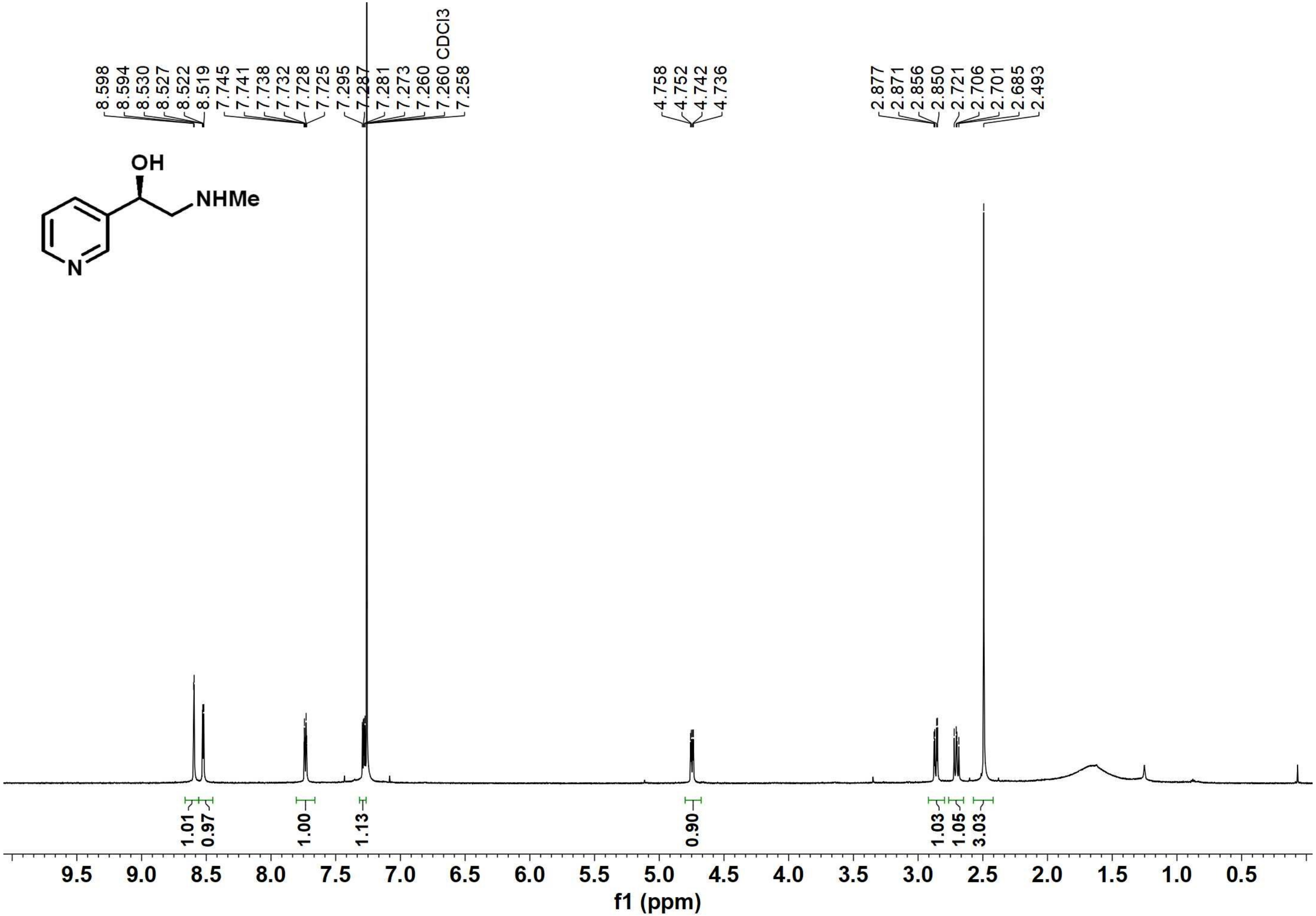

^{1}H NMR of Compound 27 (600 MHz, $CDCl_3$)

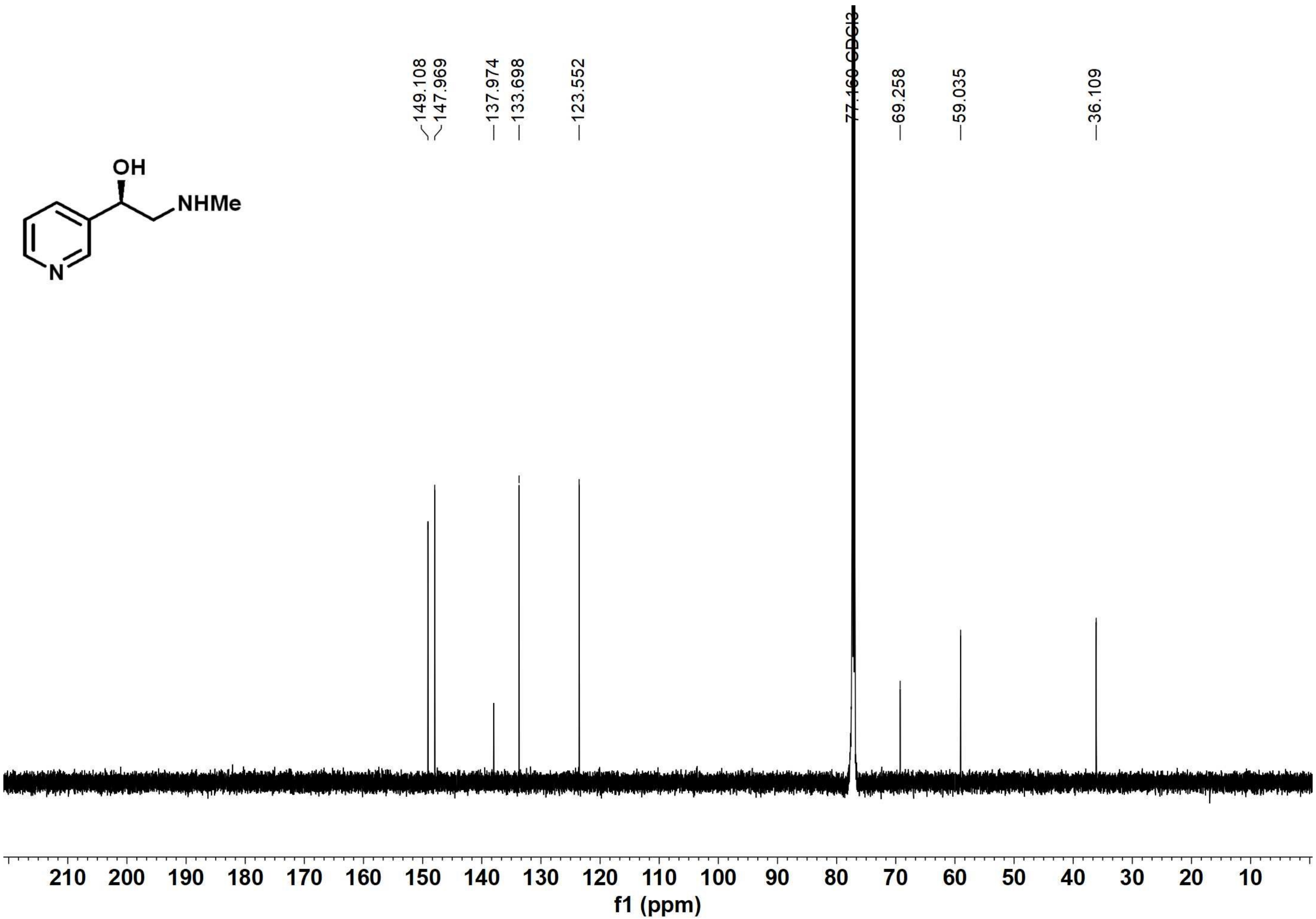

^{13}C NMR of Compound 27 (151 MHz, $CDCl_3$)

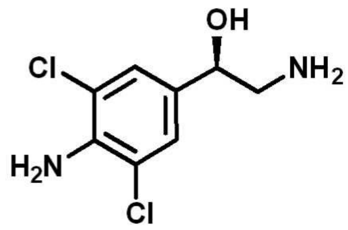

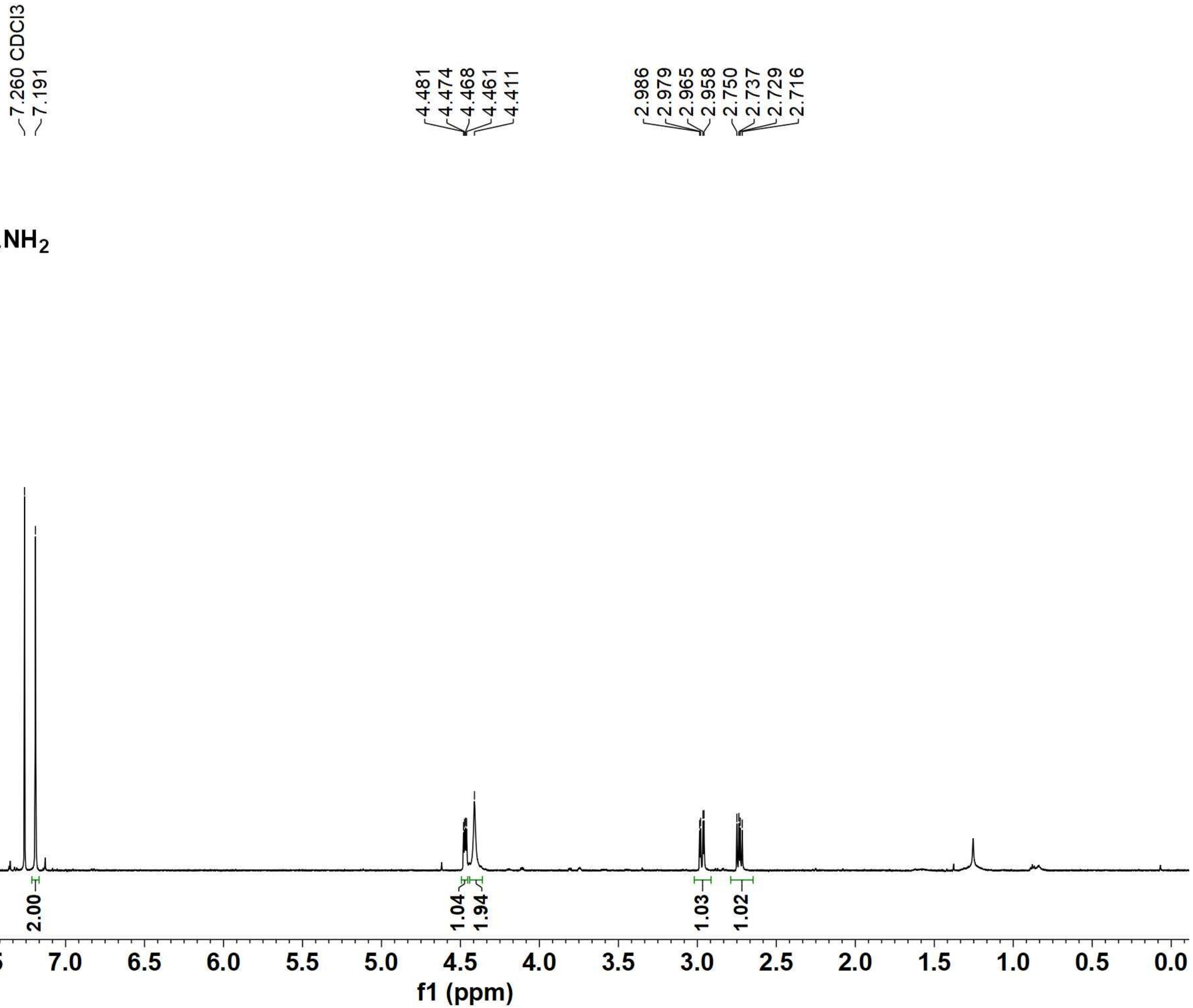

^{1}H NMR of Compound 28 (600 MHz, $CDCl_3$)

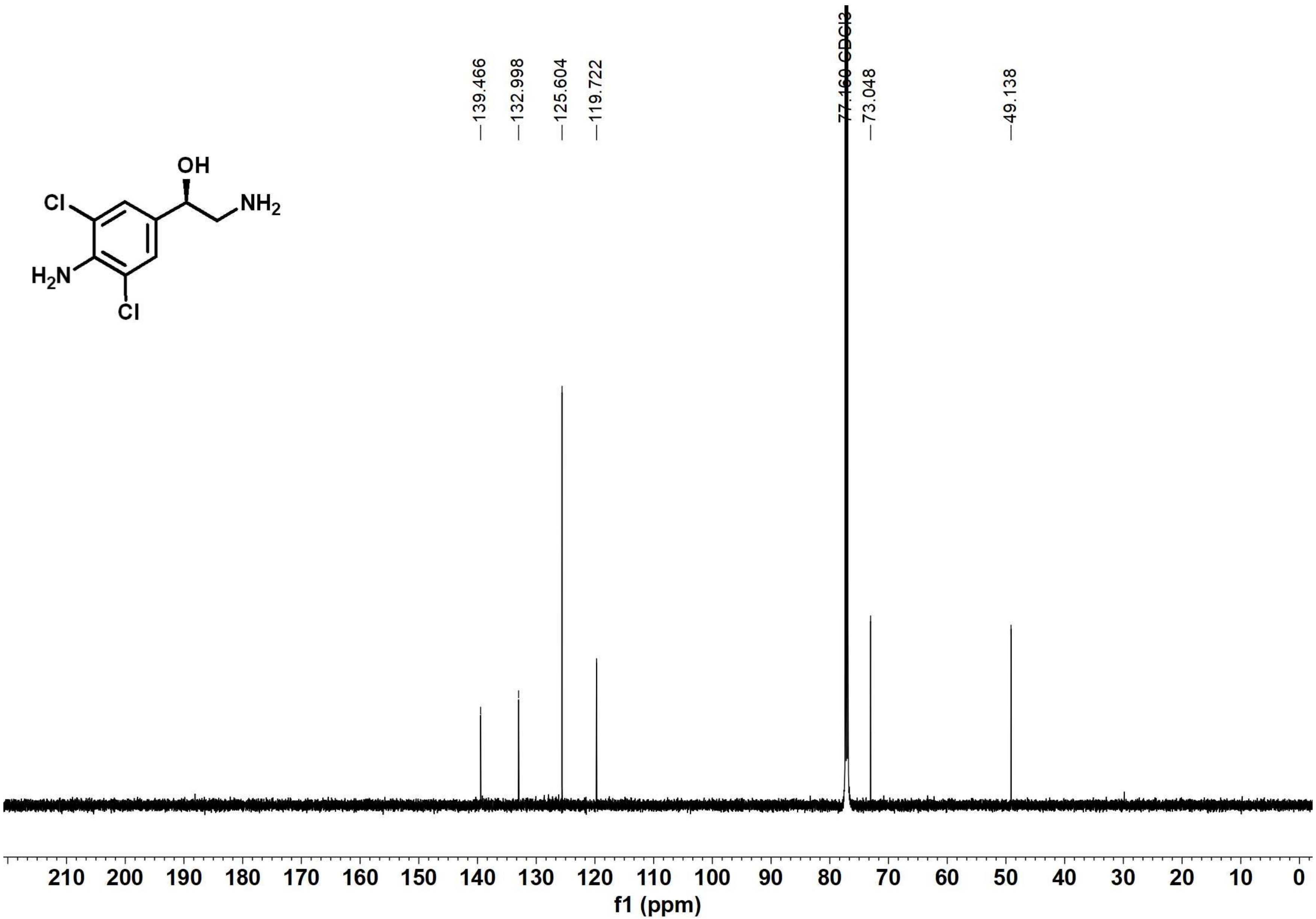

^{13}C NMR of Compound 28 (151 MHz, $CDCl_3$)

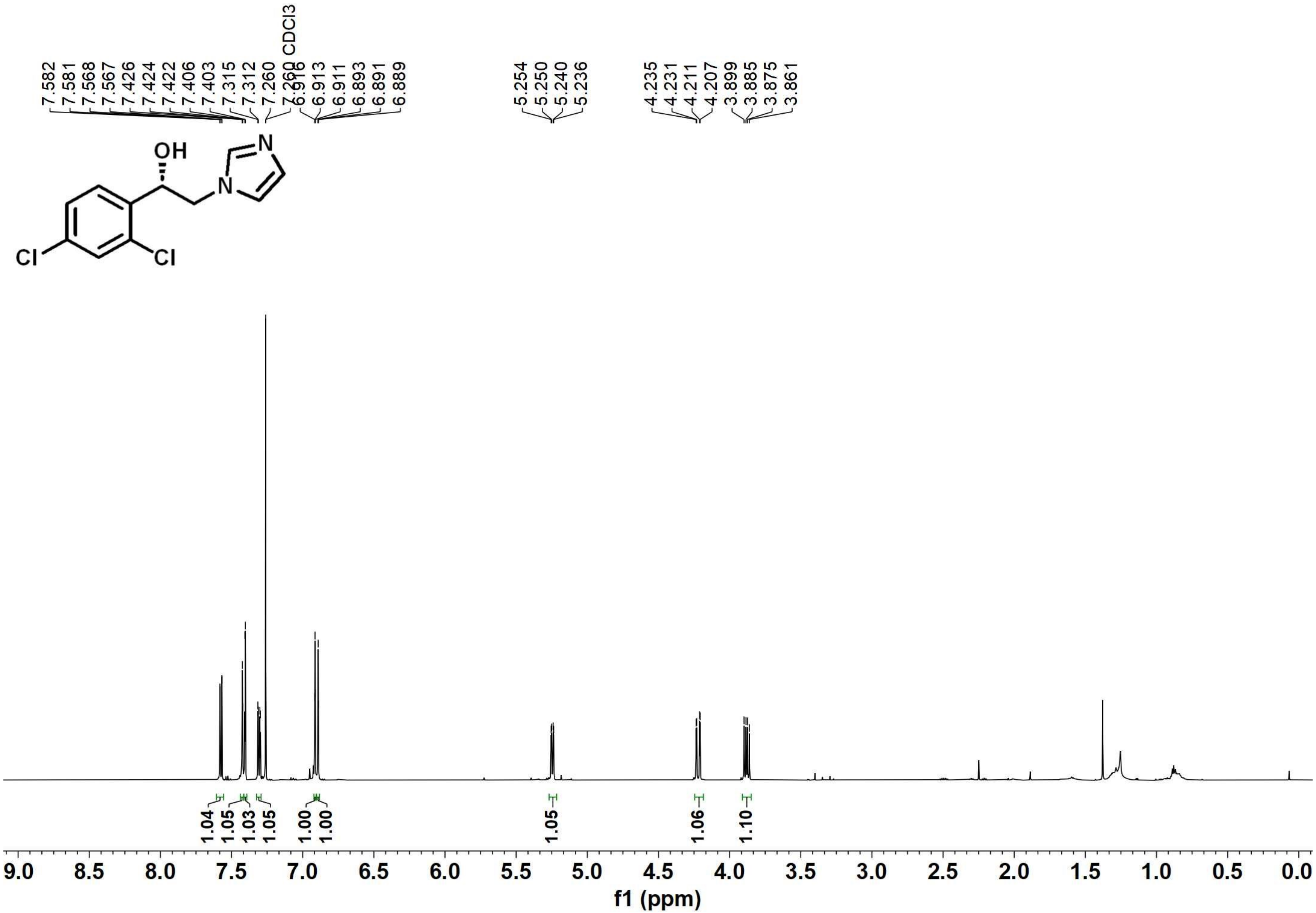

^{1}H NMR of Compound S-11 (600 MHz, $CDCl_3$)

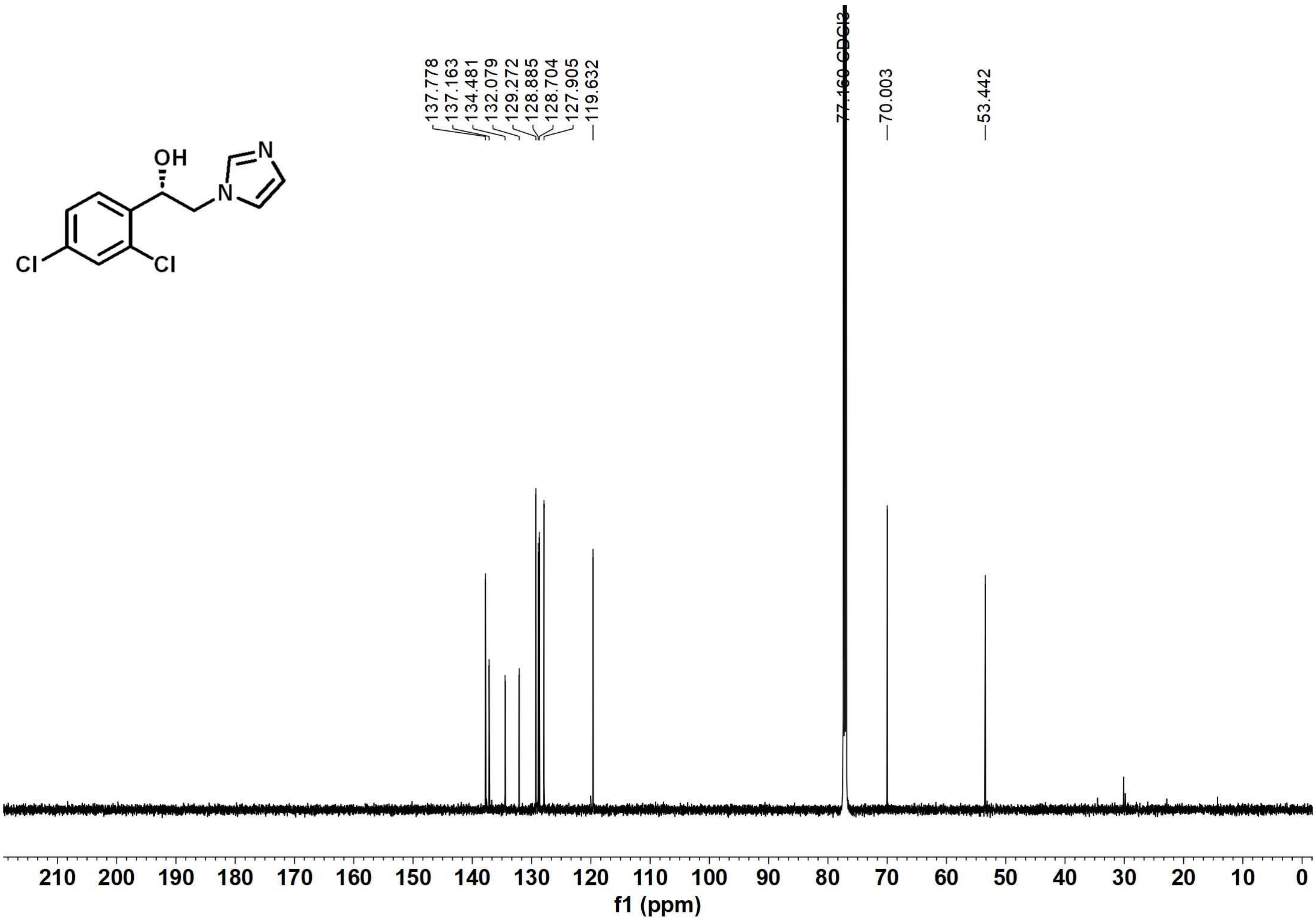

^{13}C NMR of Compound S-11 (151 MHz, $CDCl_3$)

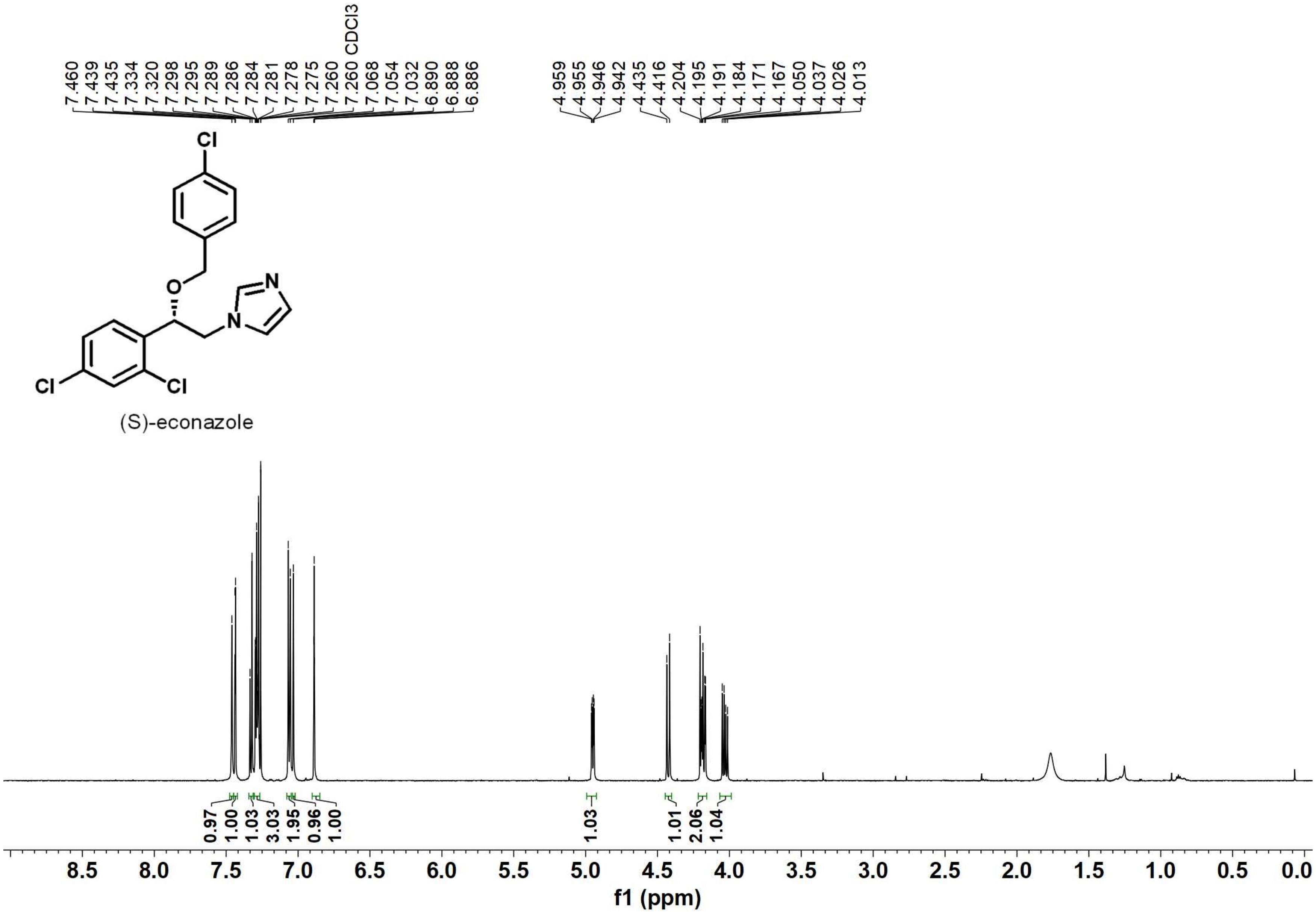

^{1}H NMR of Compound (*S*)-econazole (1) (600 MHz, $CDCl_3$)

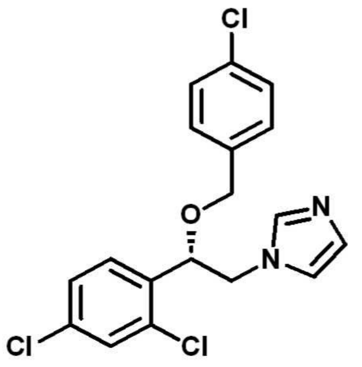

(S)-econazole

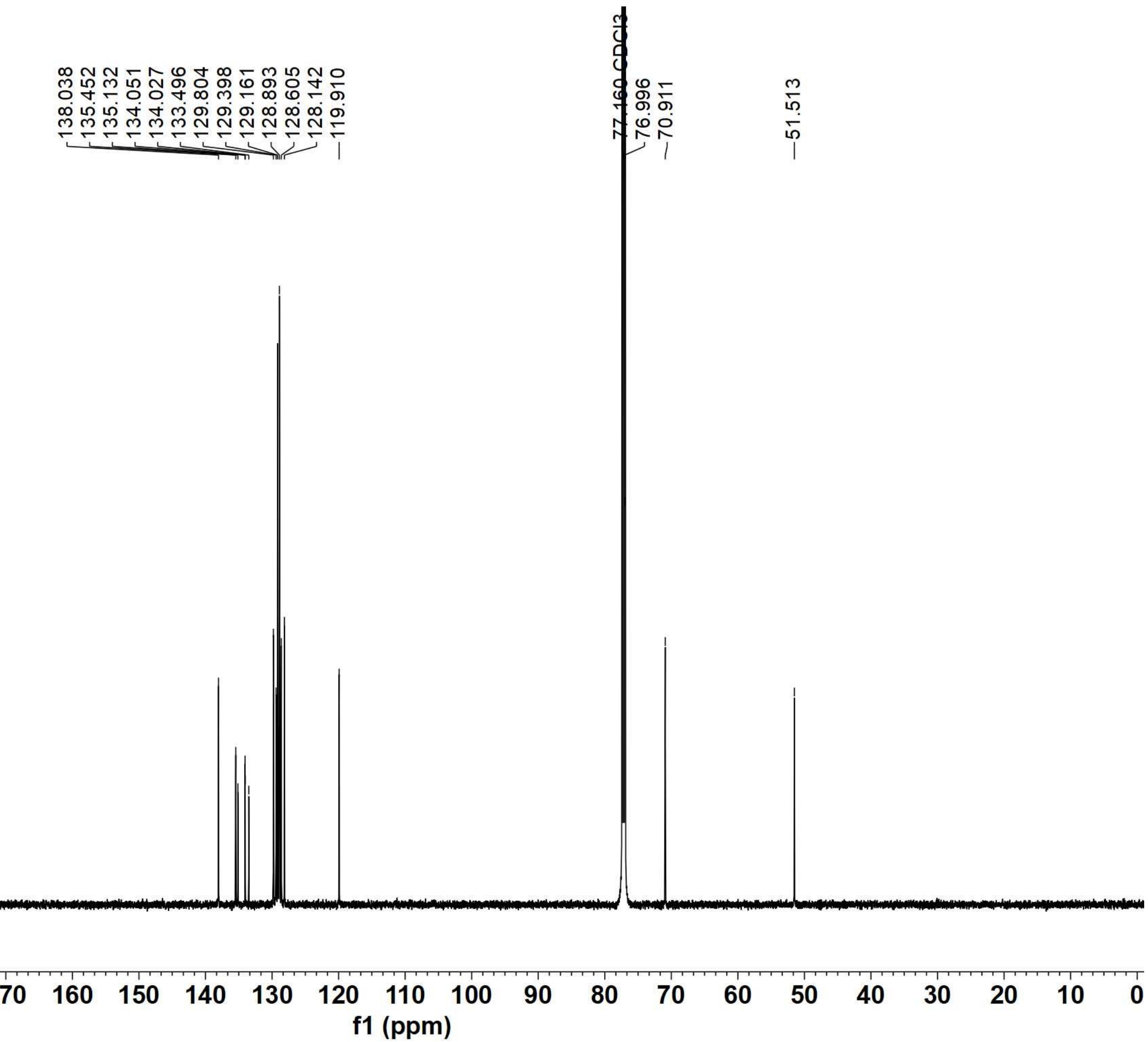

^{13}C NMR of Compound (*S*)-econazole (1) (151 MHz, $CDCl_3$)

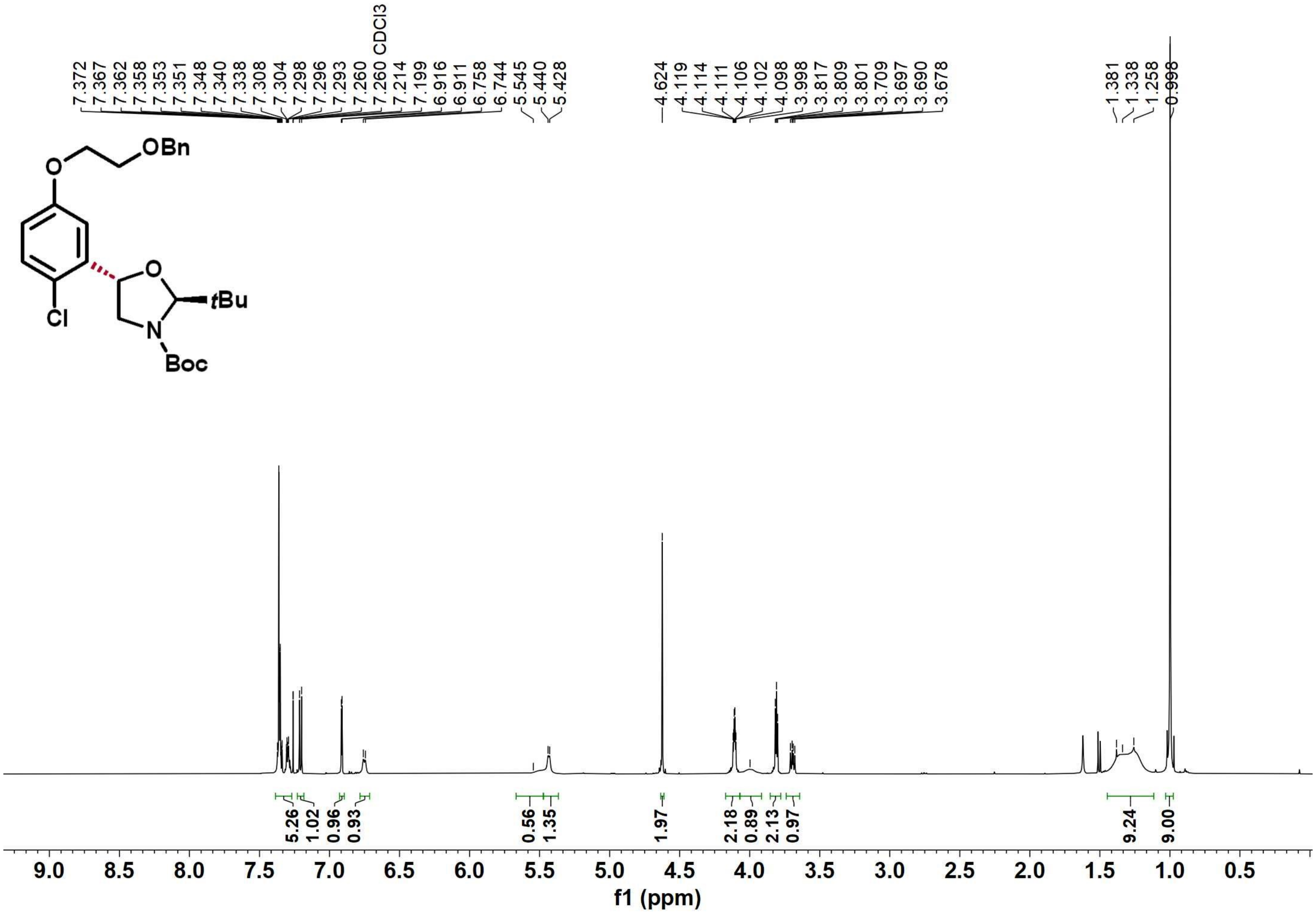

^{1}H NMR of Compound 30 (600 MHz, $CDCl_3$)

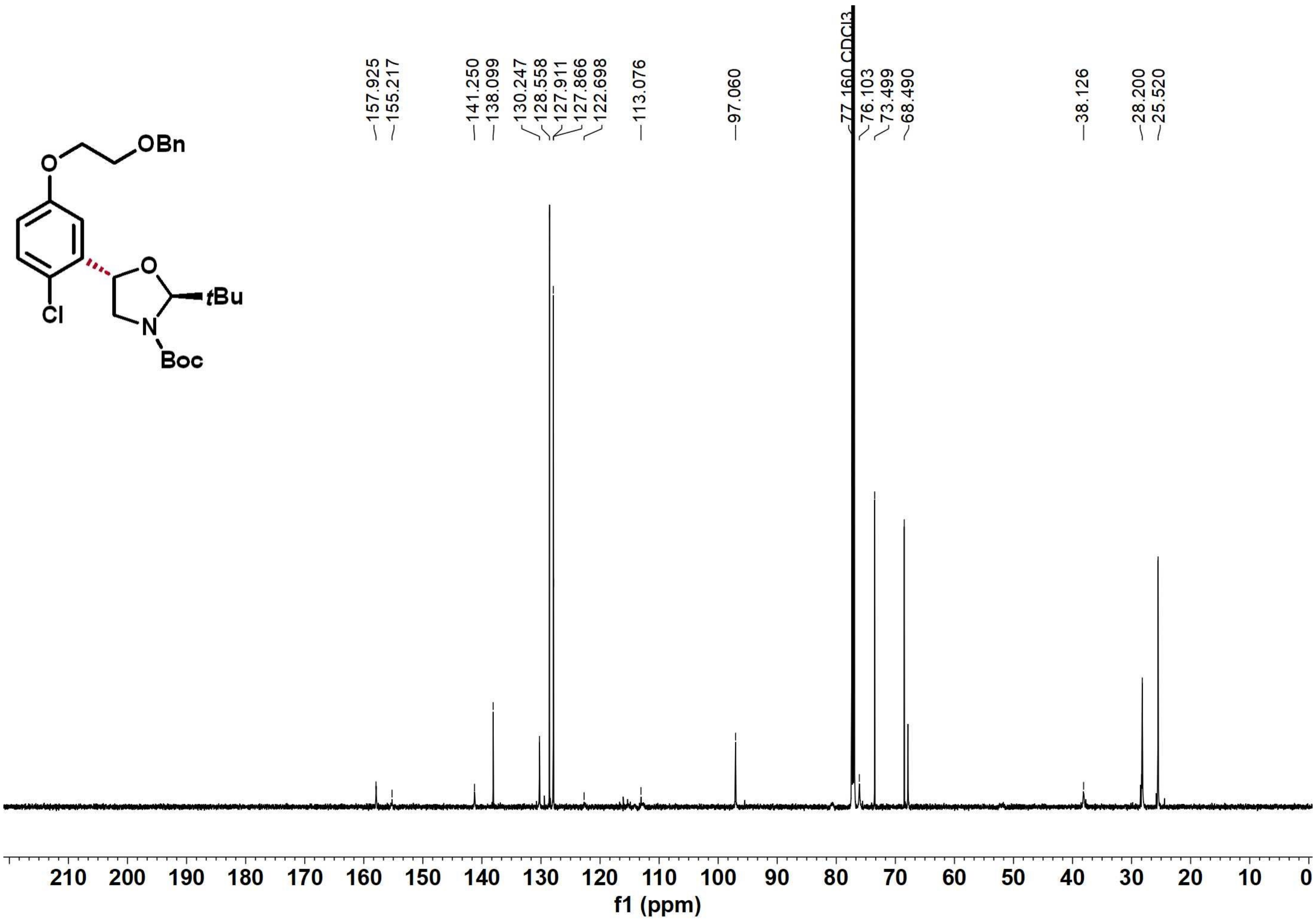

^{13}C NMR of Compound 30 (151 MHz, $CDCl_3$)

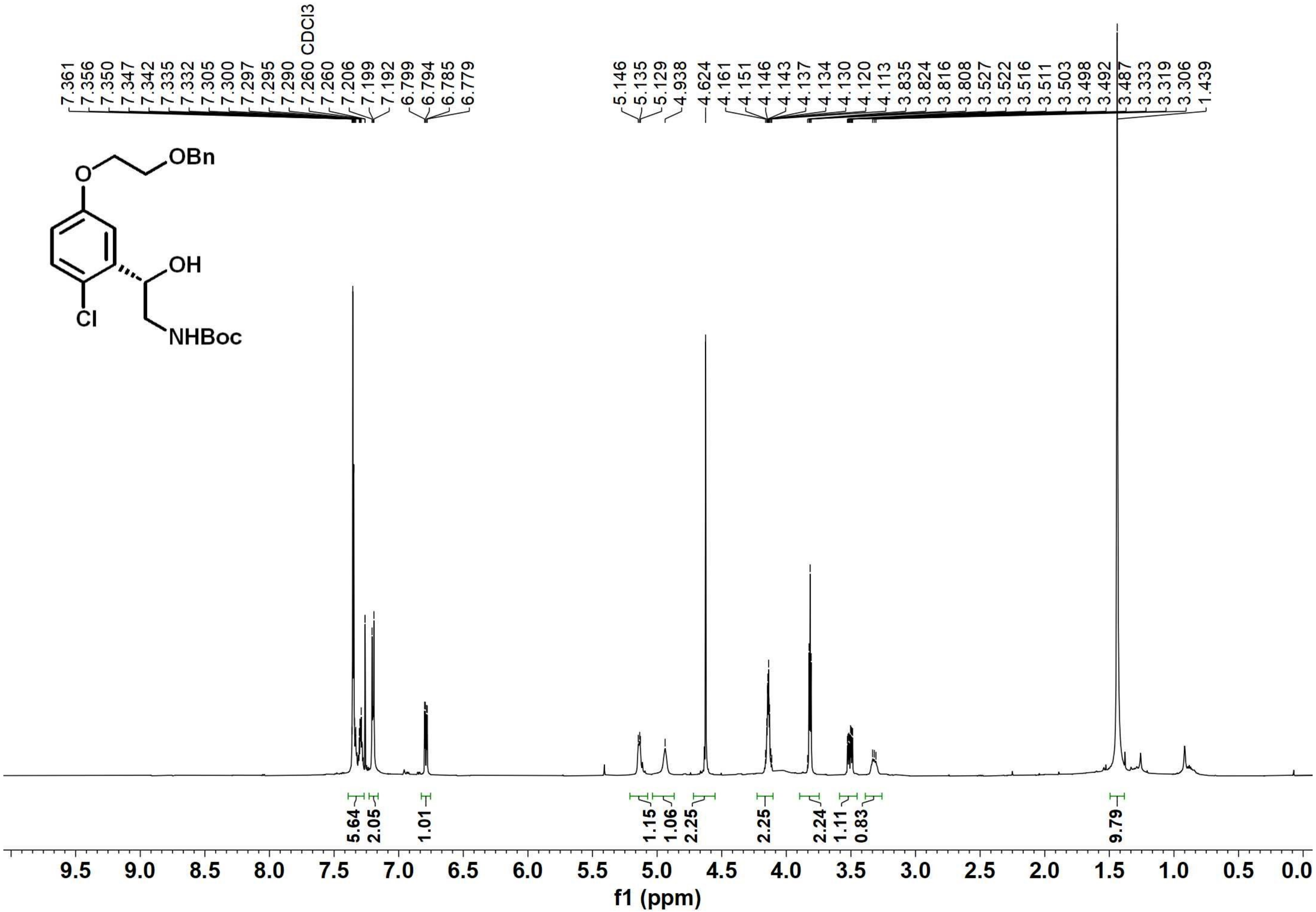

^{1}H NMR of Compound 31 (600 MHz, $CDCl_3$)

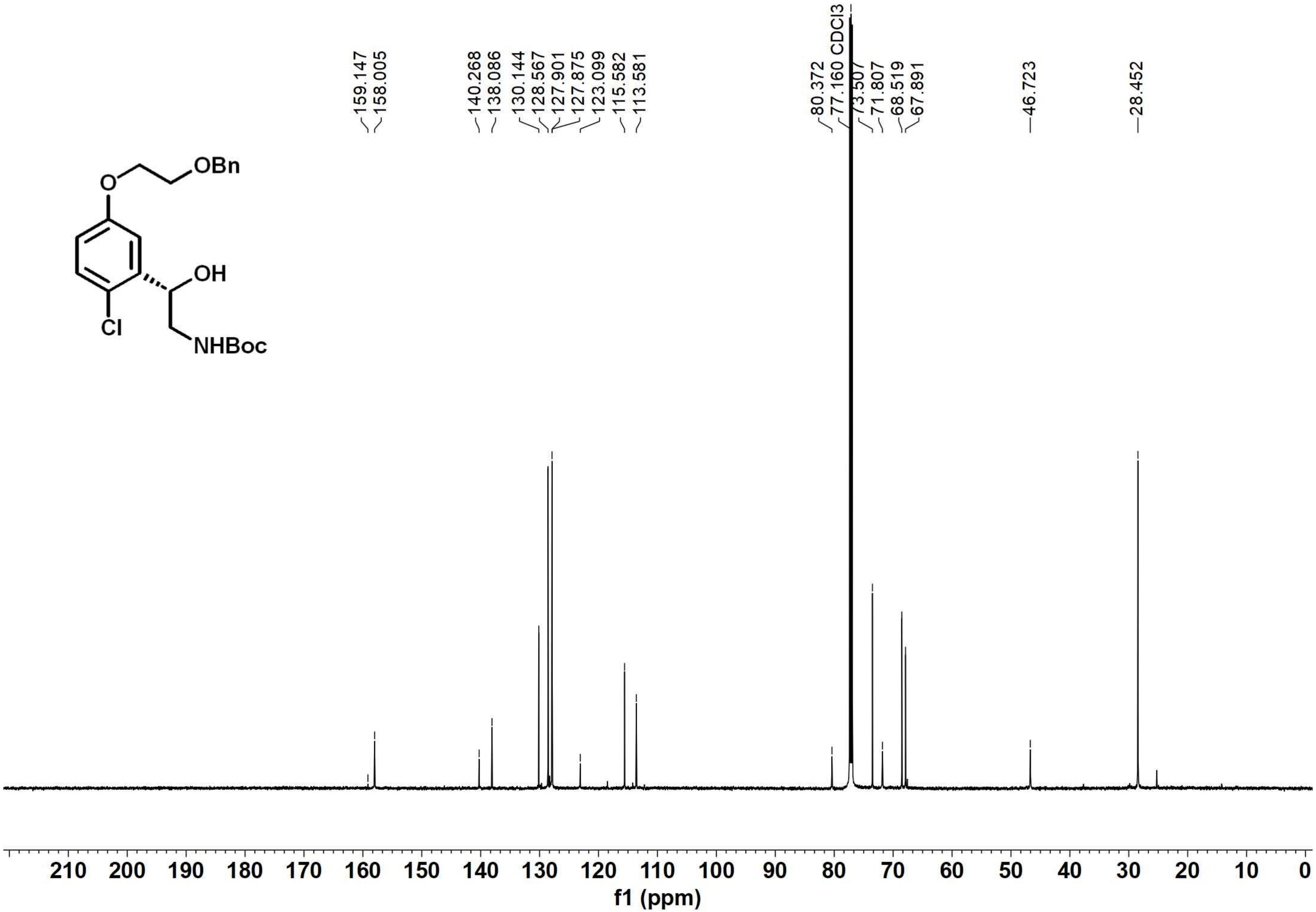

^{13}C NMR of Compound 31 (151 MHz, $CDCl_3$)

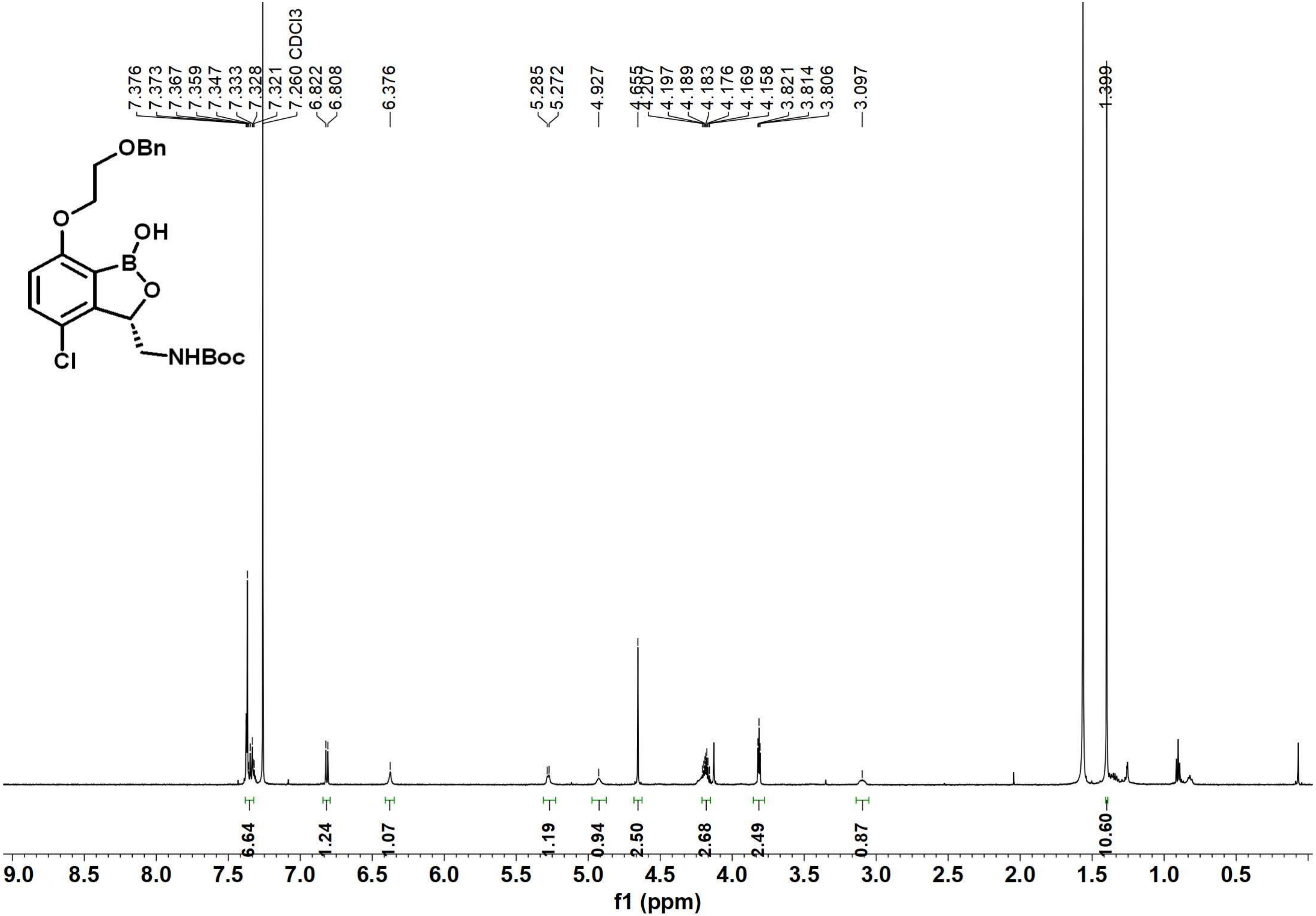

^{1}H NMR of Compound S-12 (600 MHz, $CDCl_3$)

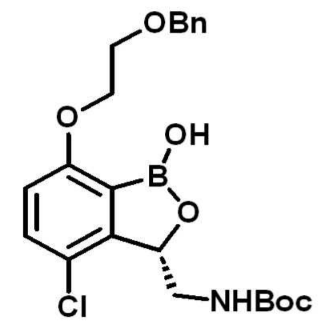

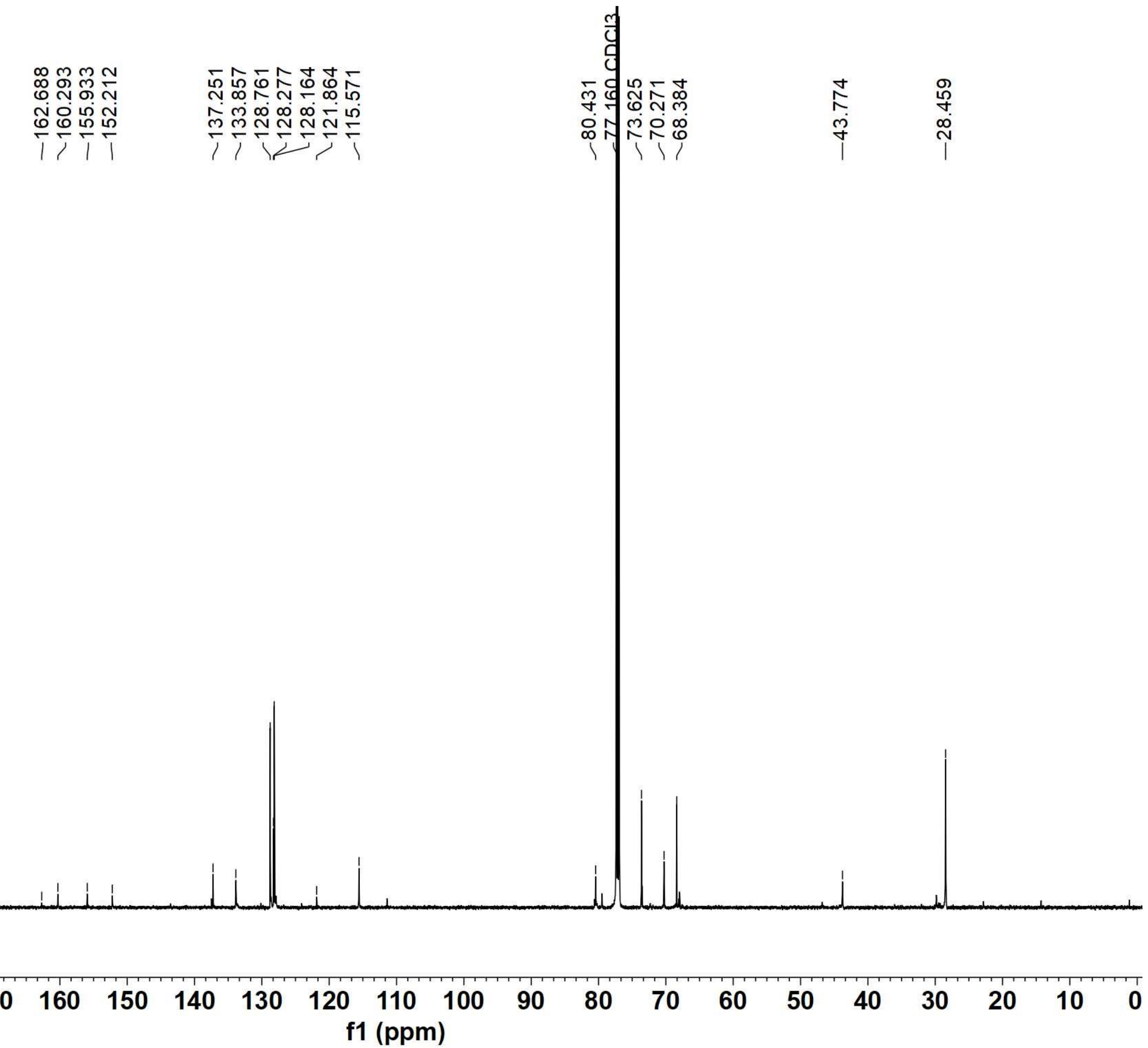

^{13}C NMR of Compound S-12 (151 MHz, $CDCl_3$)

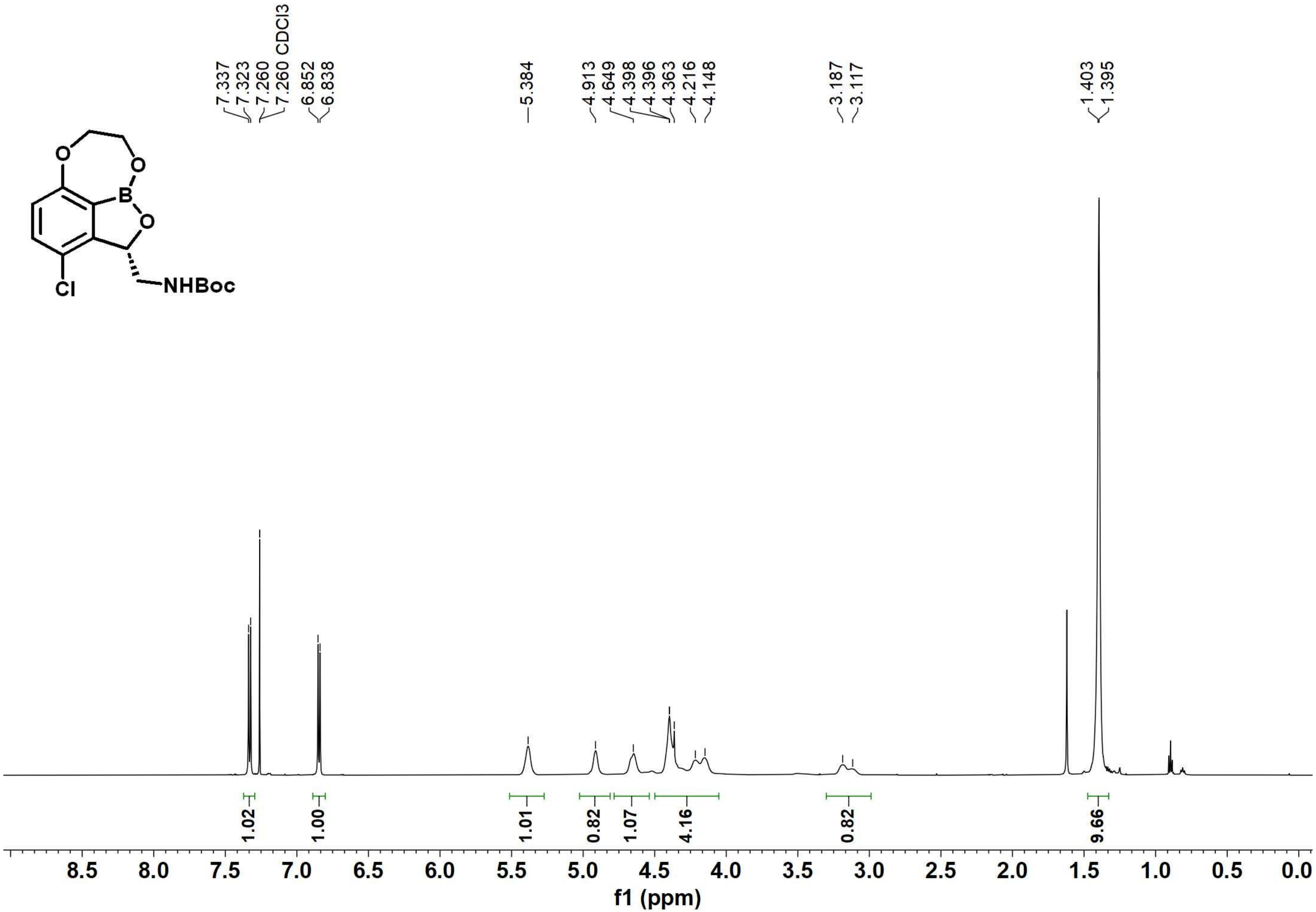

^{1}H NMR of Compound S-13 (600 MHz, $CDCl_3$)

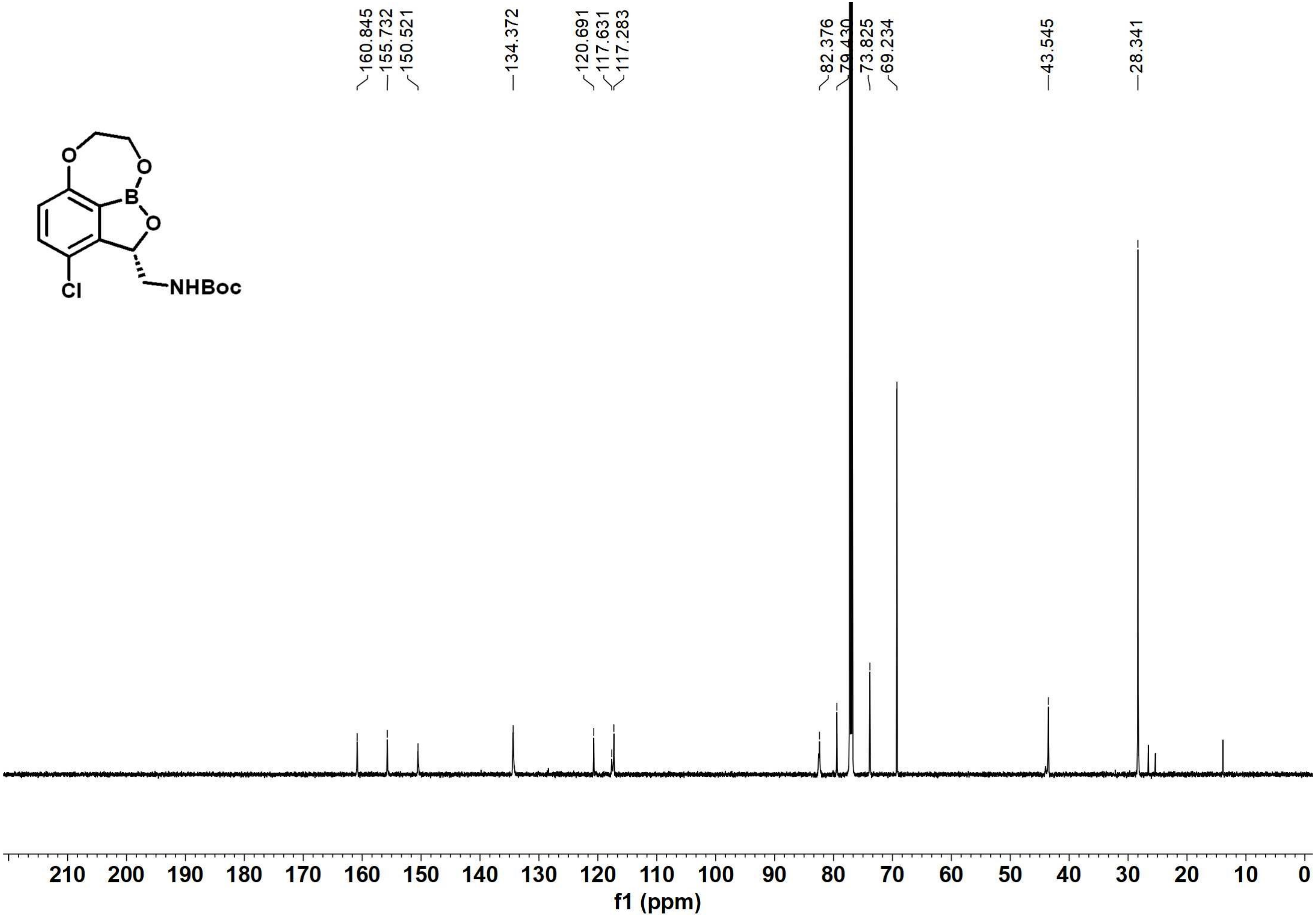

^{13}C NMR of Compound S-13 (151 MHz, $CDCl_3$)

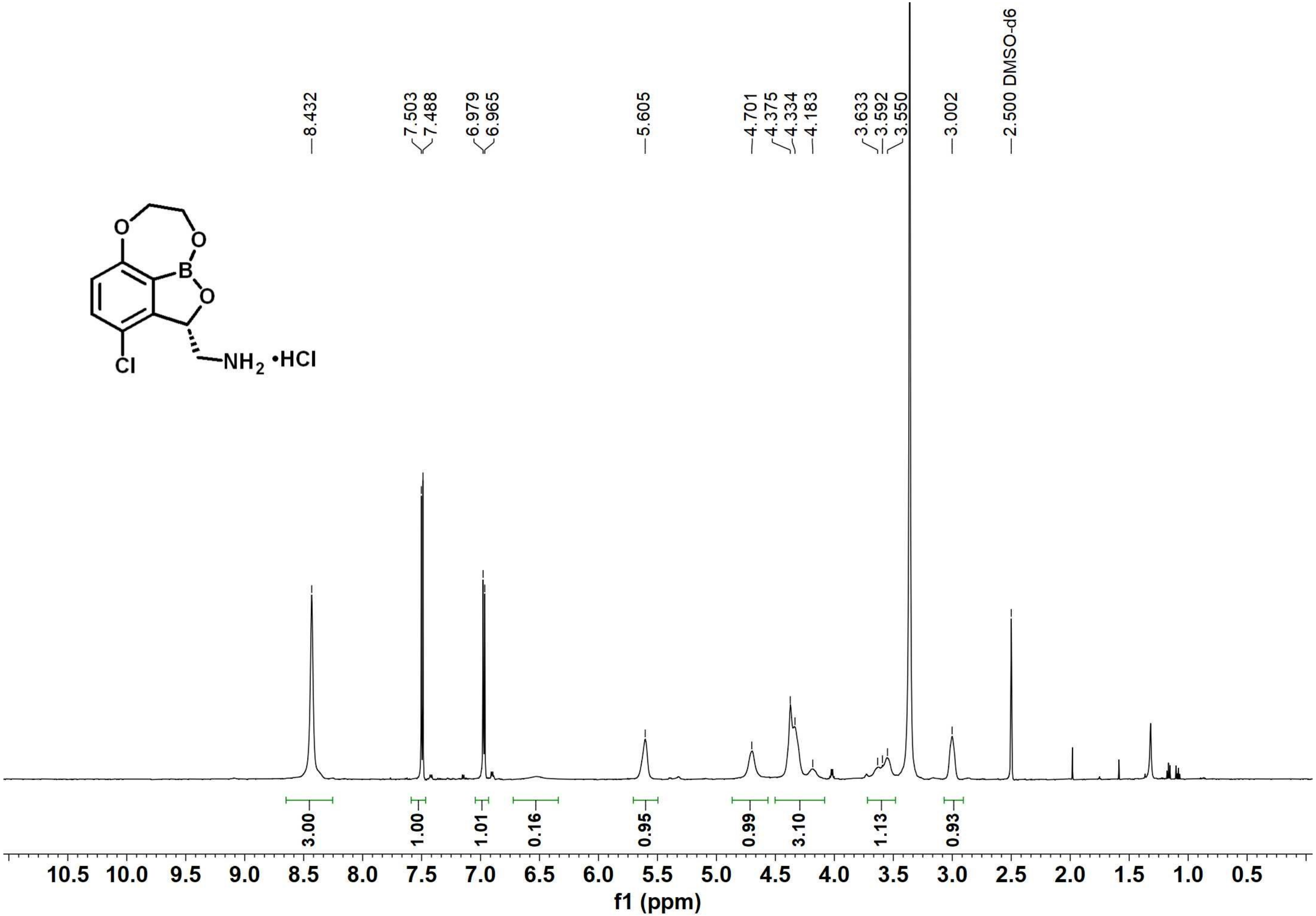

^{1}H NMR of Compound GSK-656 (4) (600 MHz, DMSO-*d6*)

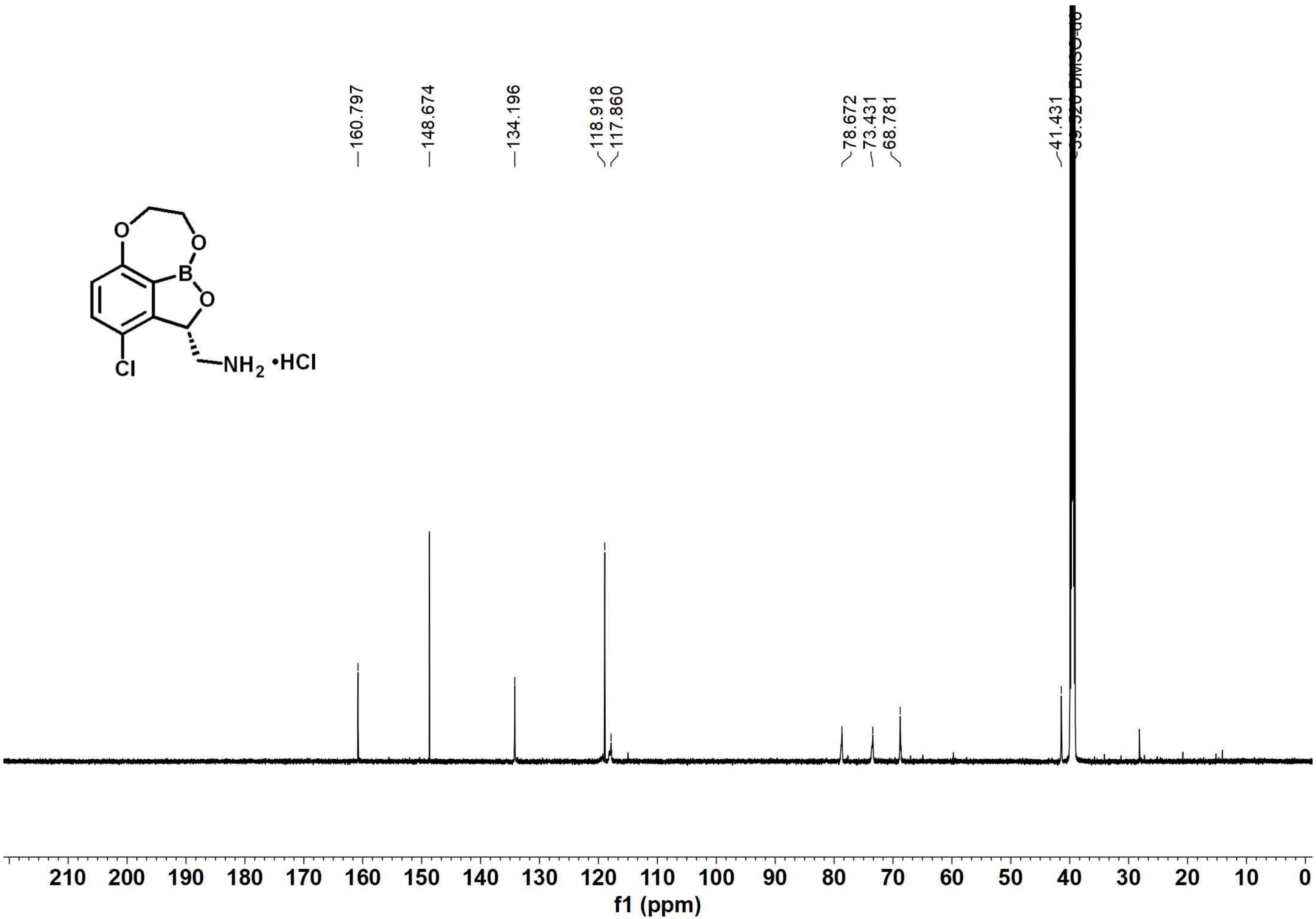

^{13}C NMR of Compound GSK-656 (4) (600 MHz, DMSO-*d6*)

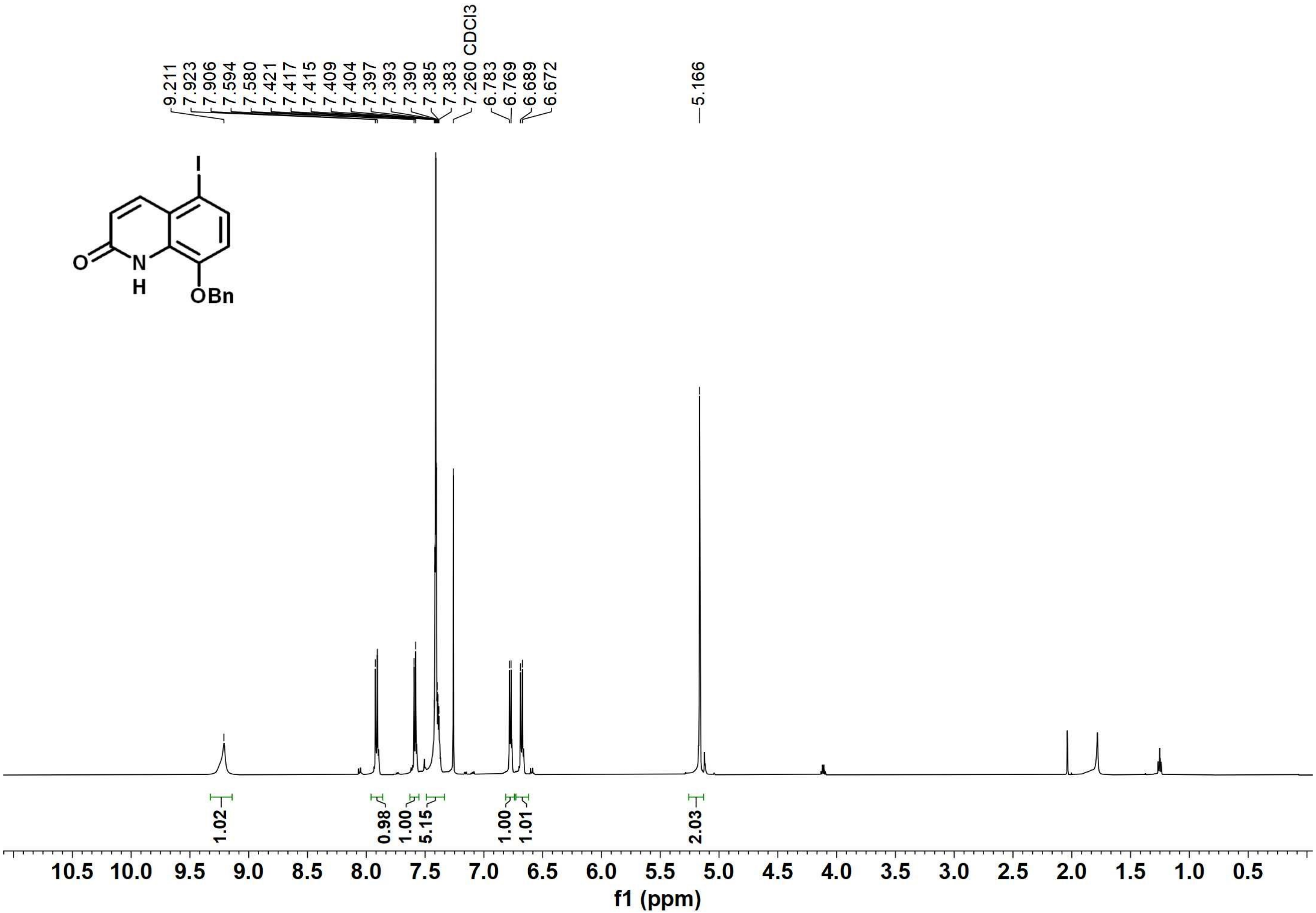

^{1}H NMR of Compound 36 (600 MHz, $CDCl_3$)

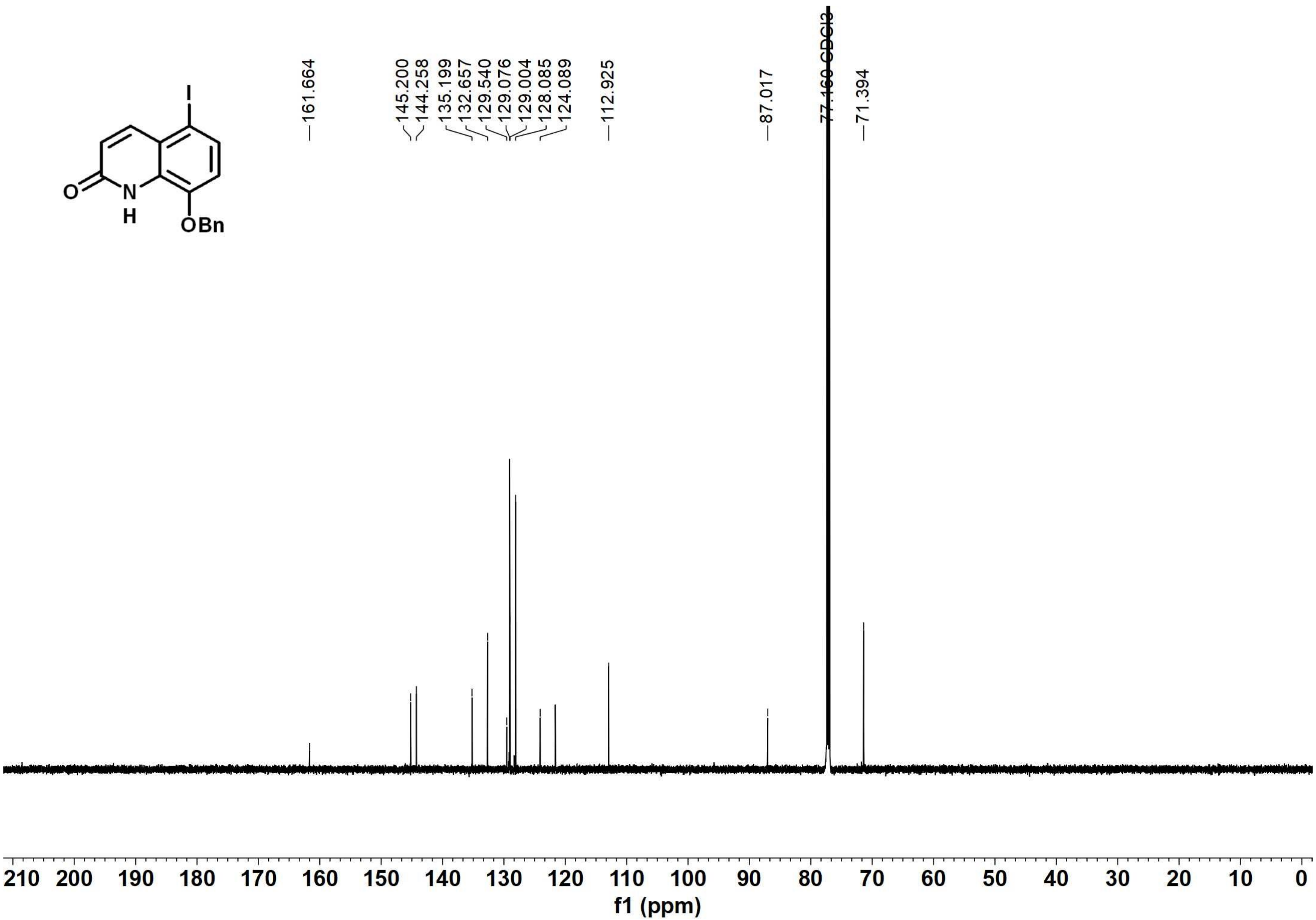

^{13}C NMR of Compound 36 (151 MHz, $CDCl_3$)

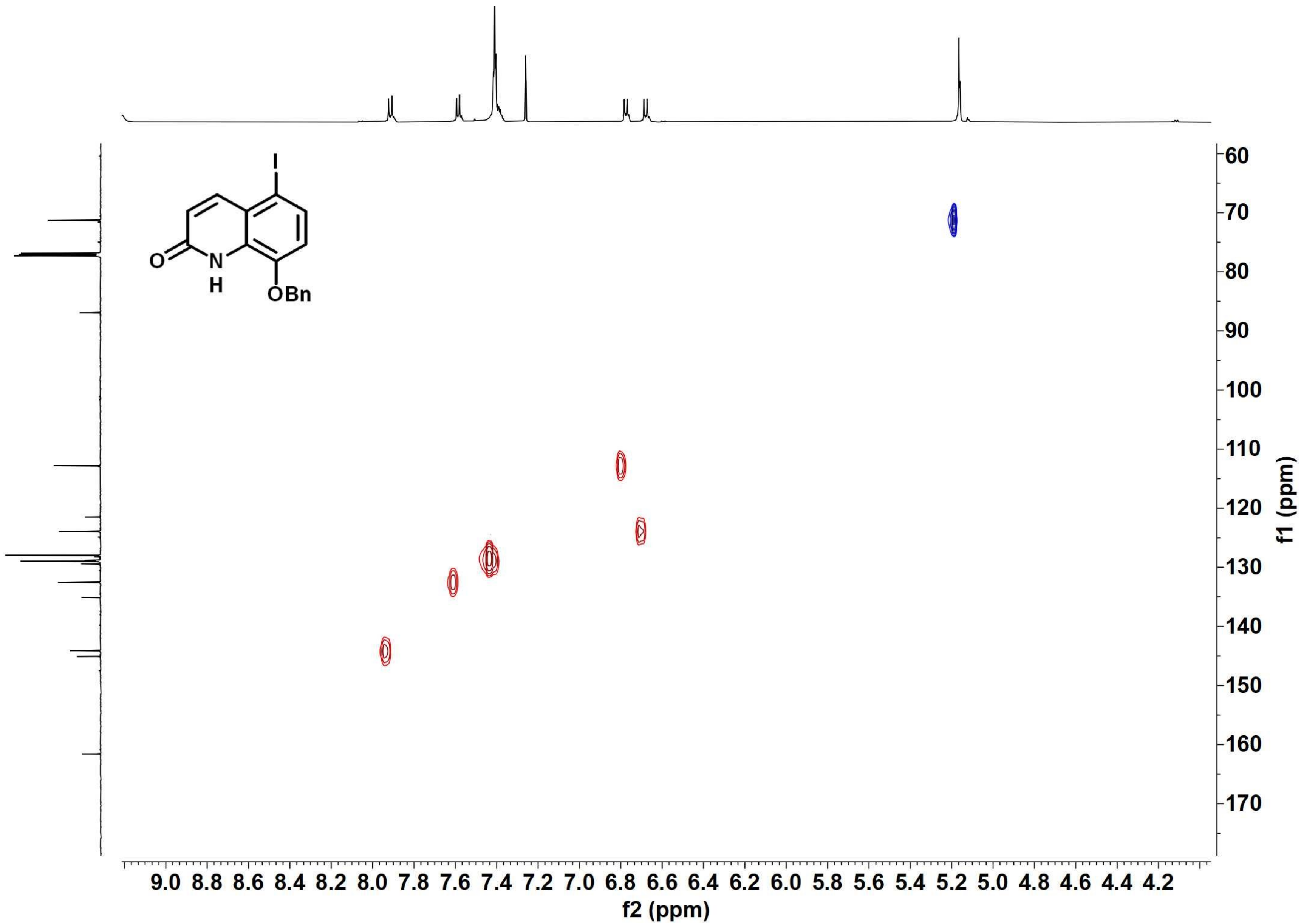

HSQC of Compound 36 ($CDCl_3$)

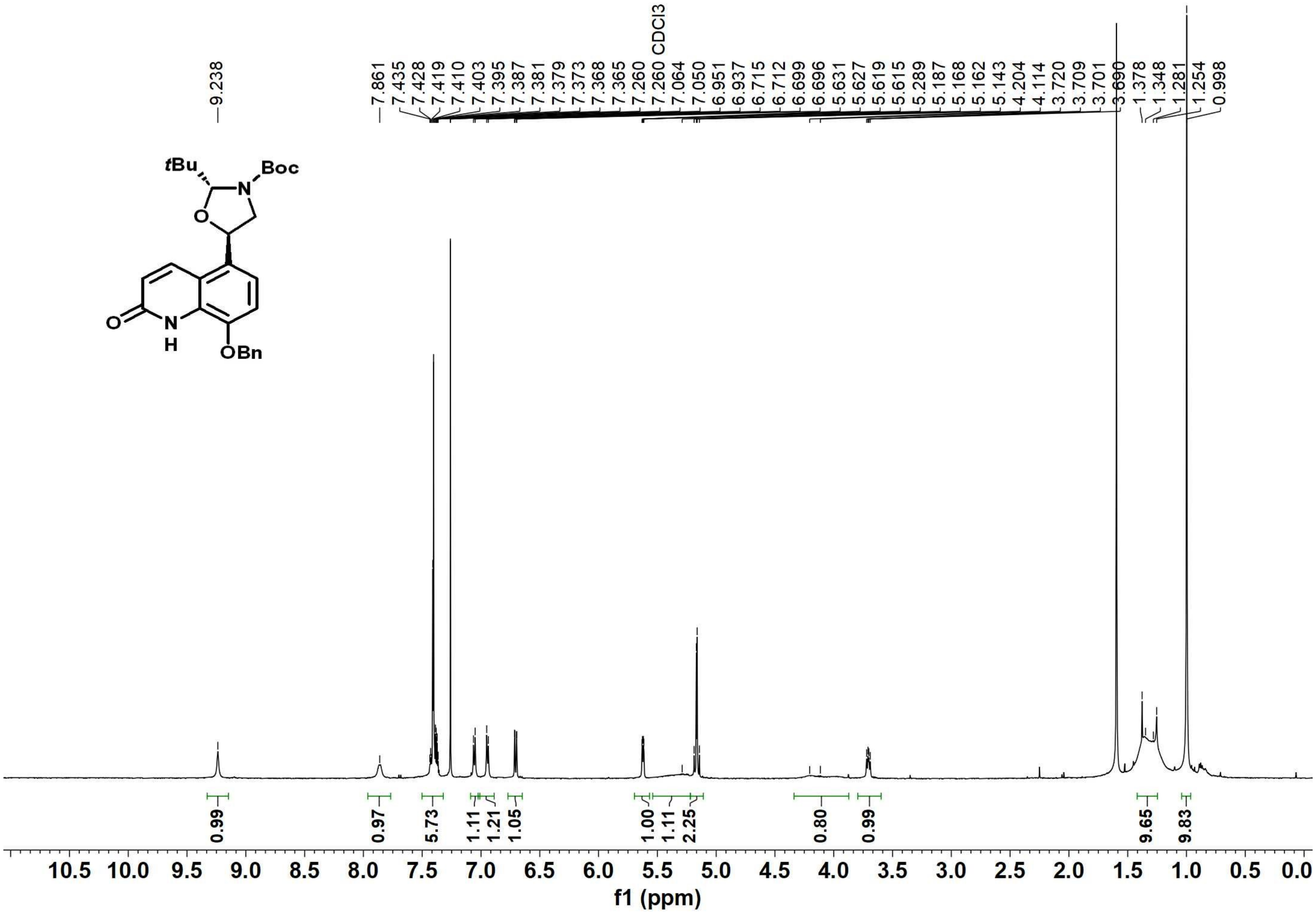

^{1}H NMR of Compound 37 (600 MHz, $CDCl_3$)

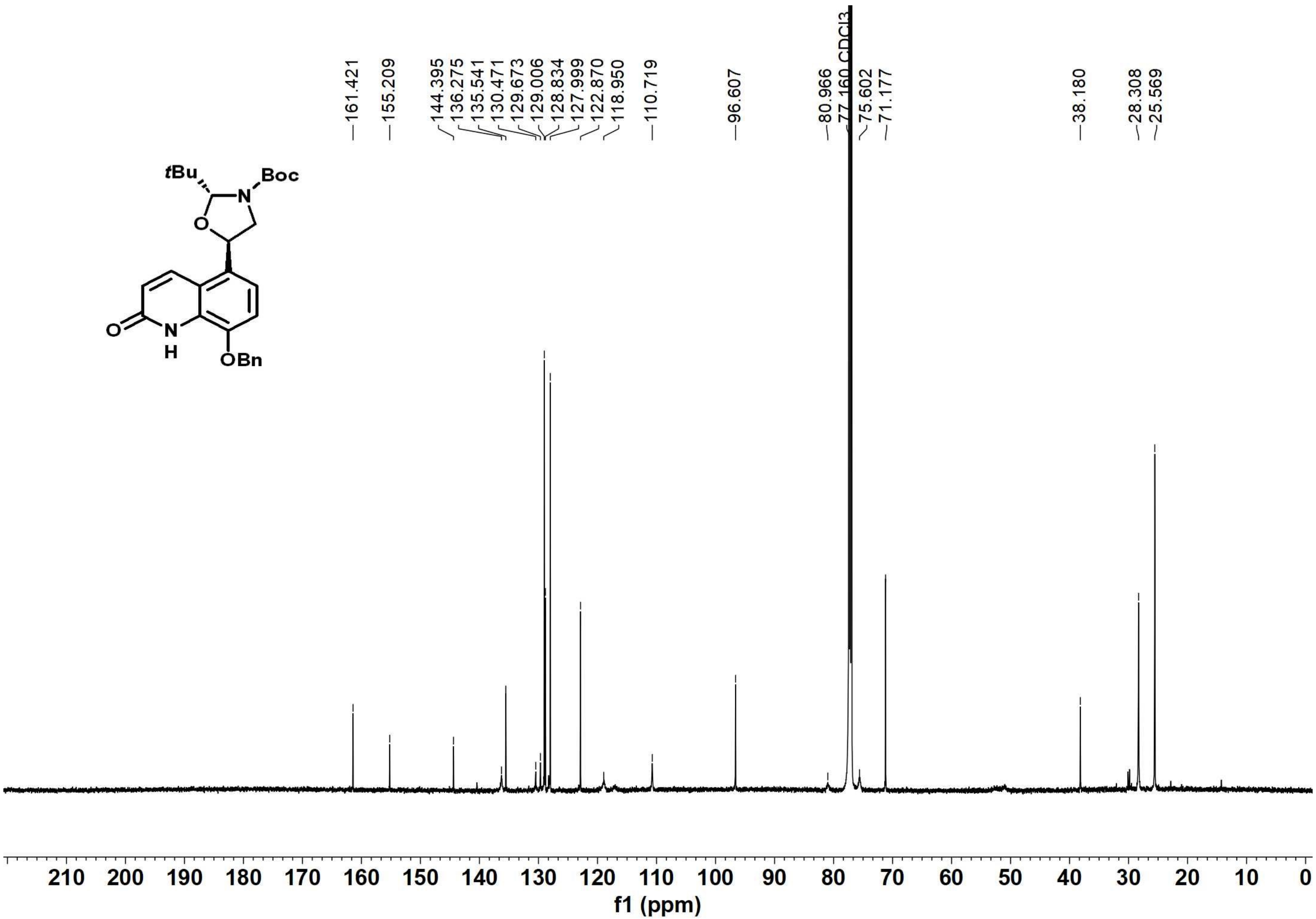

^{13}C NMR of Compound 37 (151 MHz, $CDCl_3$)

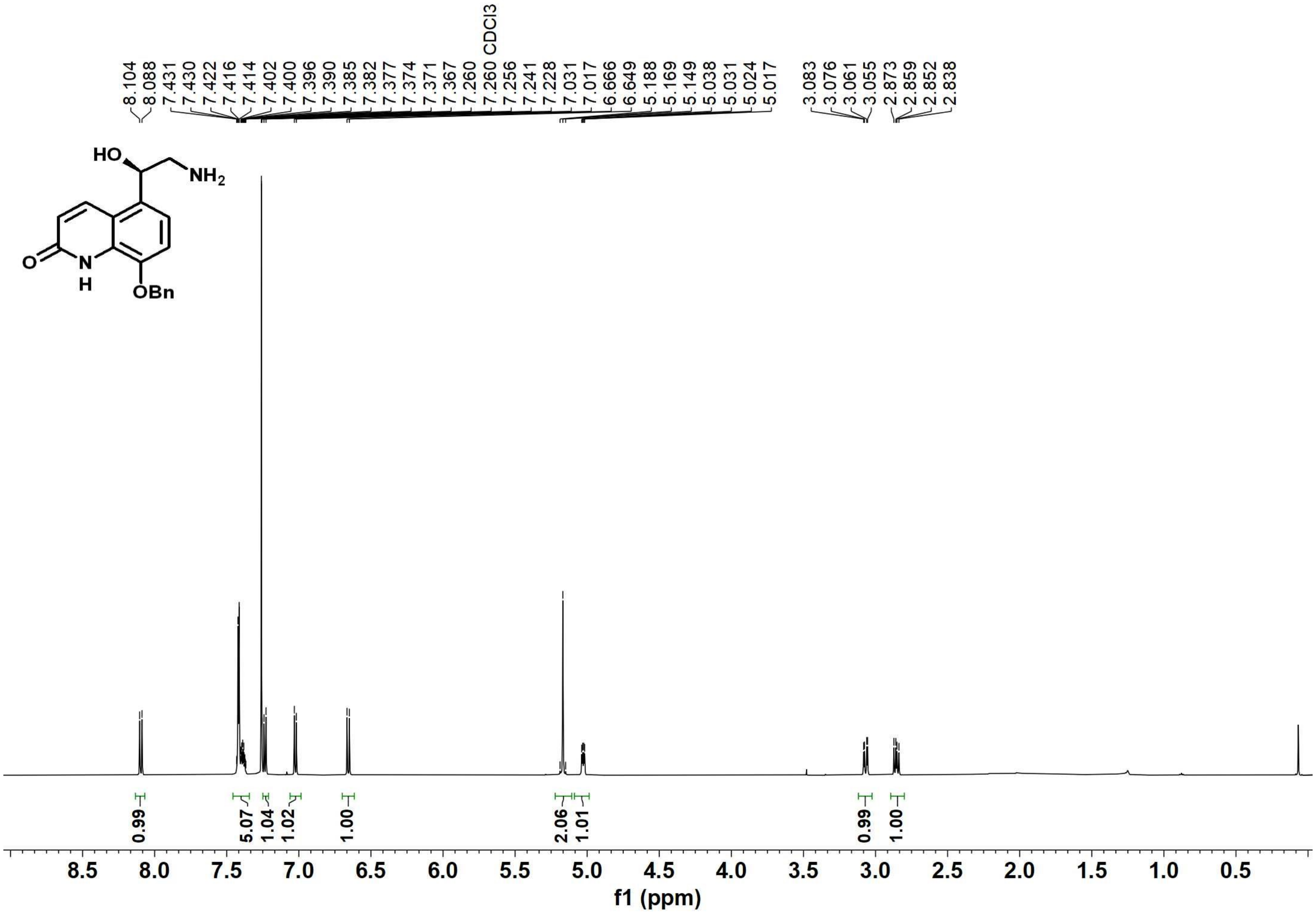

^{1}H NMR of Compound S-15 (600 MHz, $CDCl_3$)

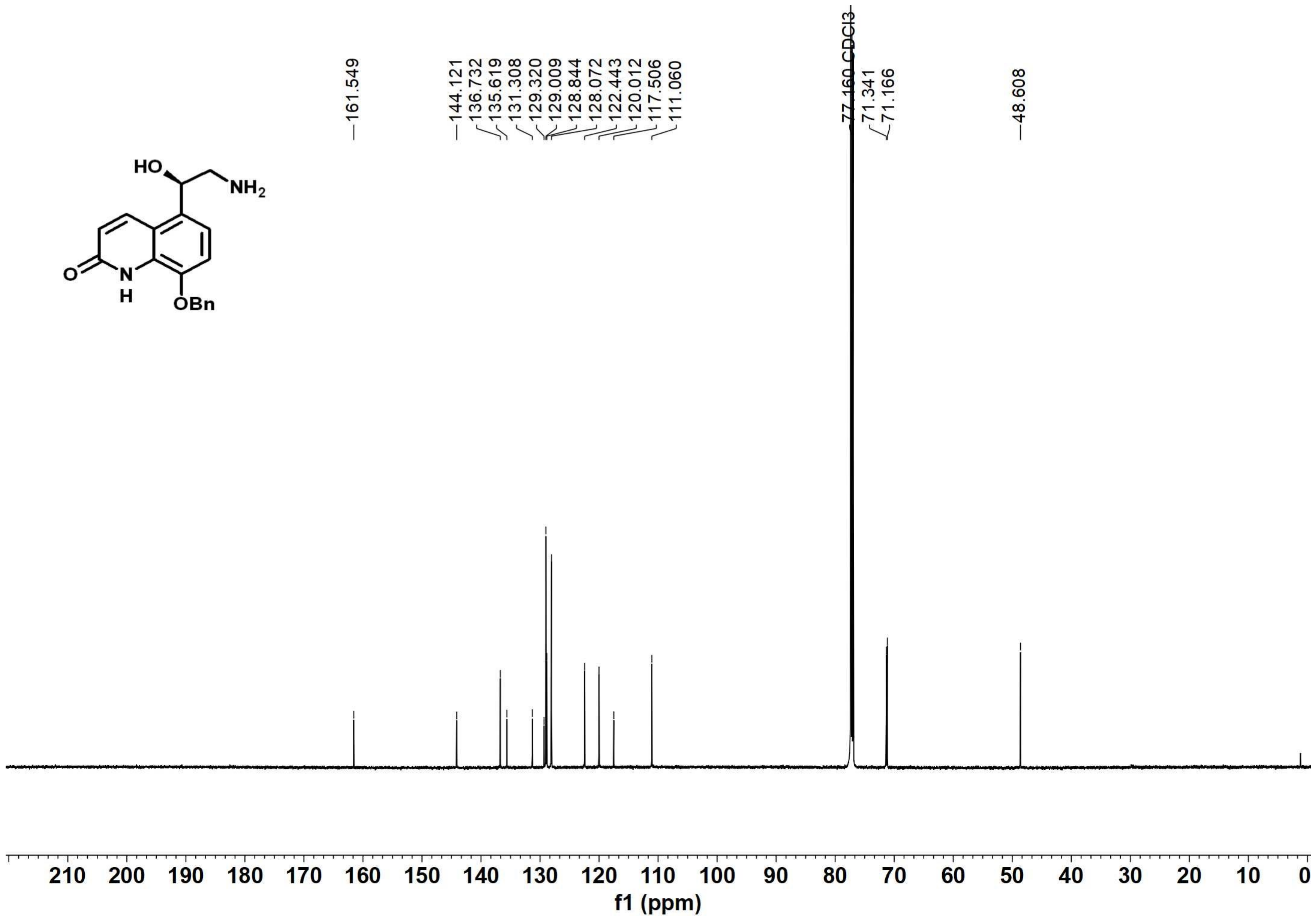

^{13}C NMR of Compound S-15 (151 MHz, $CDCl_3$)

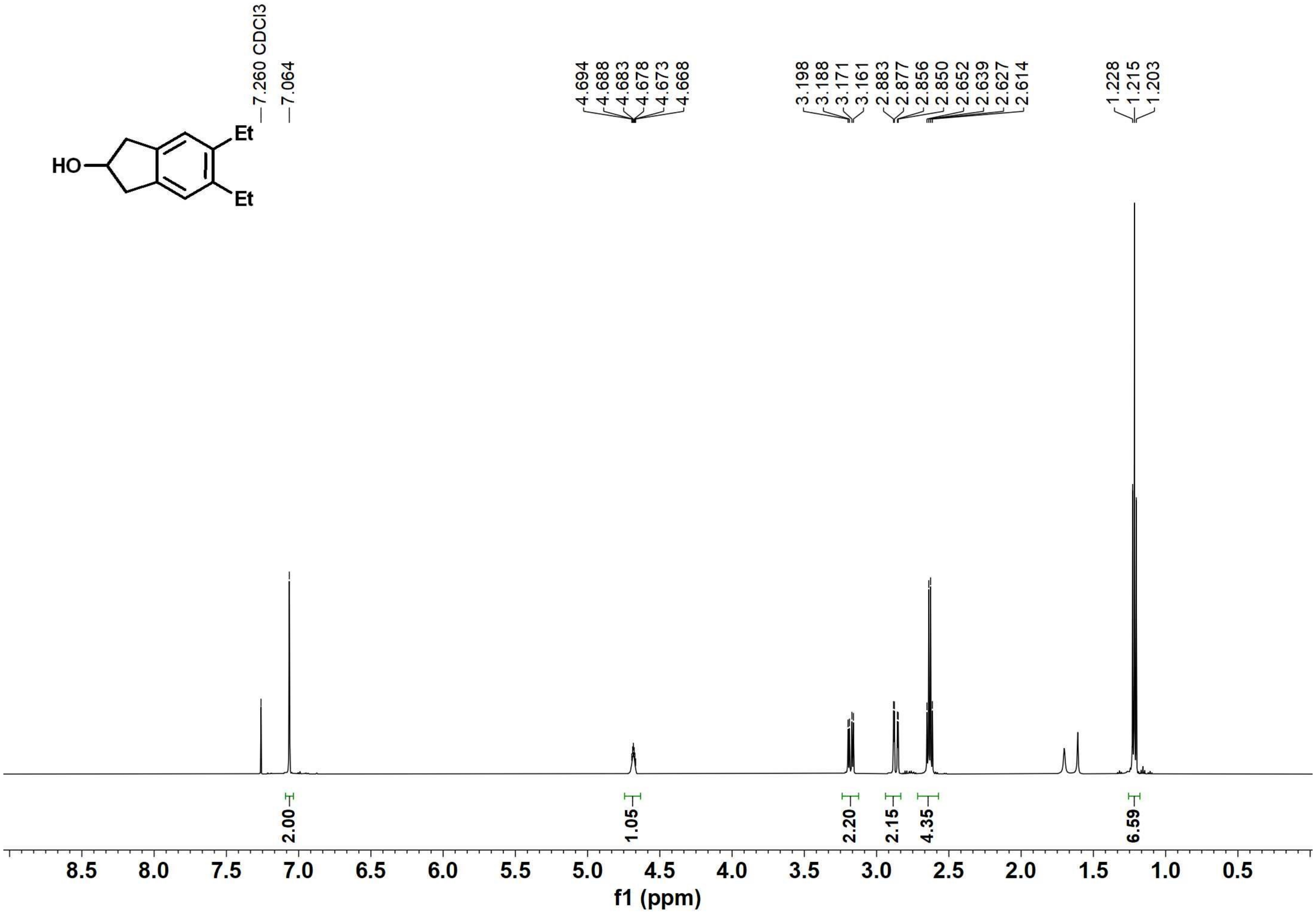

^{1}H NMR of Compound S-16 (600 MHz, $CDCl_3$)

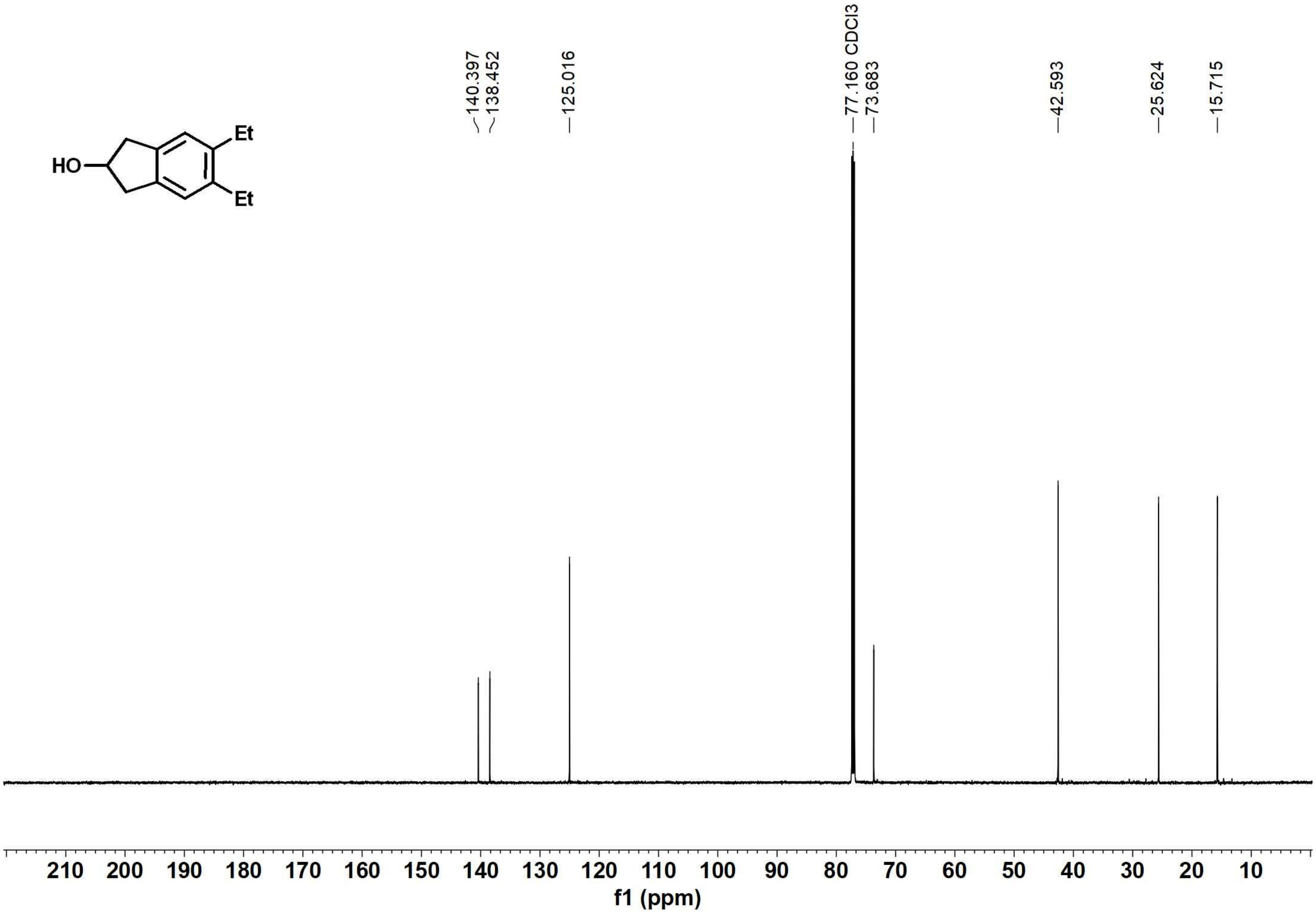

^{13}C NMR of Compound S-16 (151 MHz, $CDCl_3$)

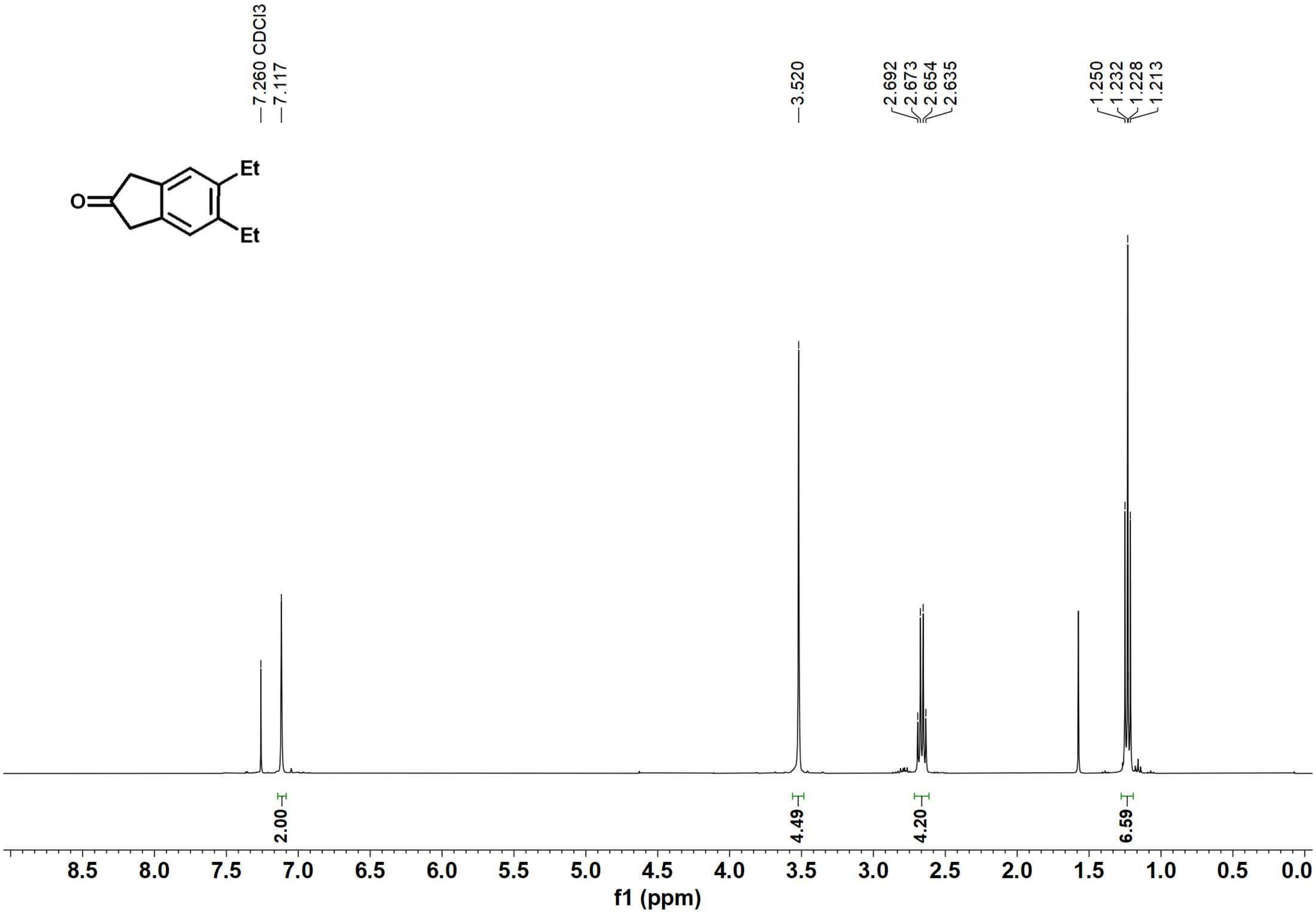

^{1}H NMR of Compound 38 (600 MHz, $CDCl_3$)

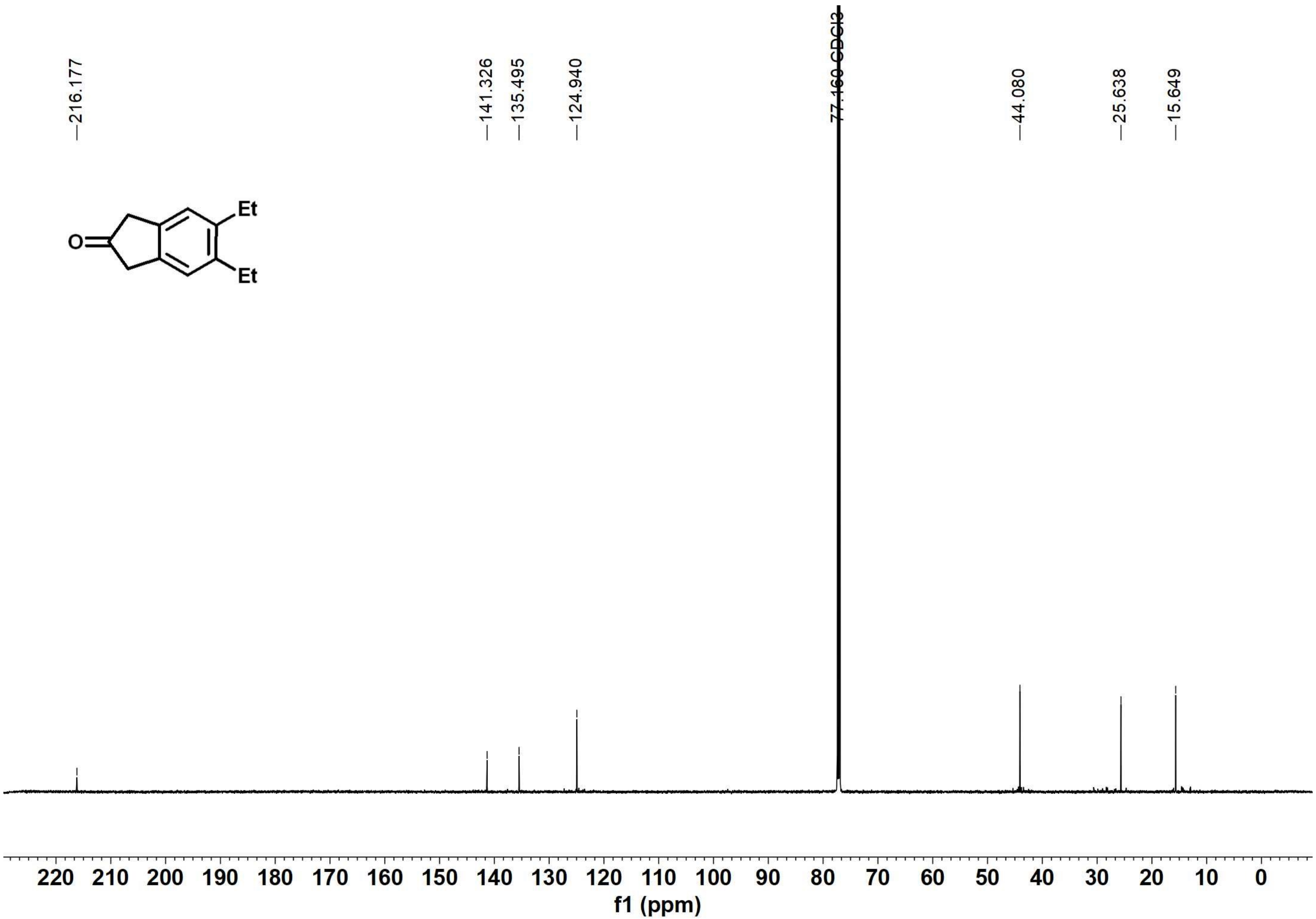

^{13}C NMR of Compound 38 (151 MHz, $CDCl_3$)

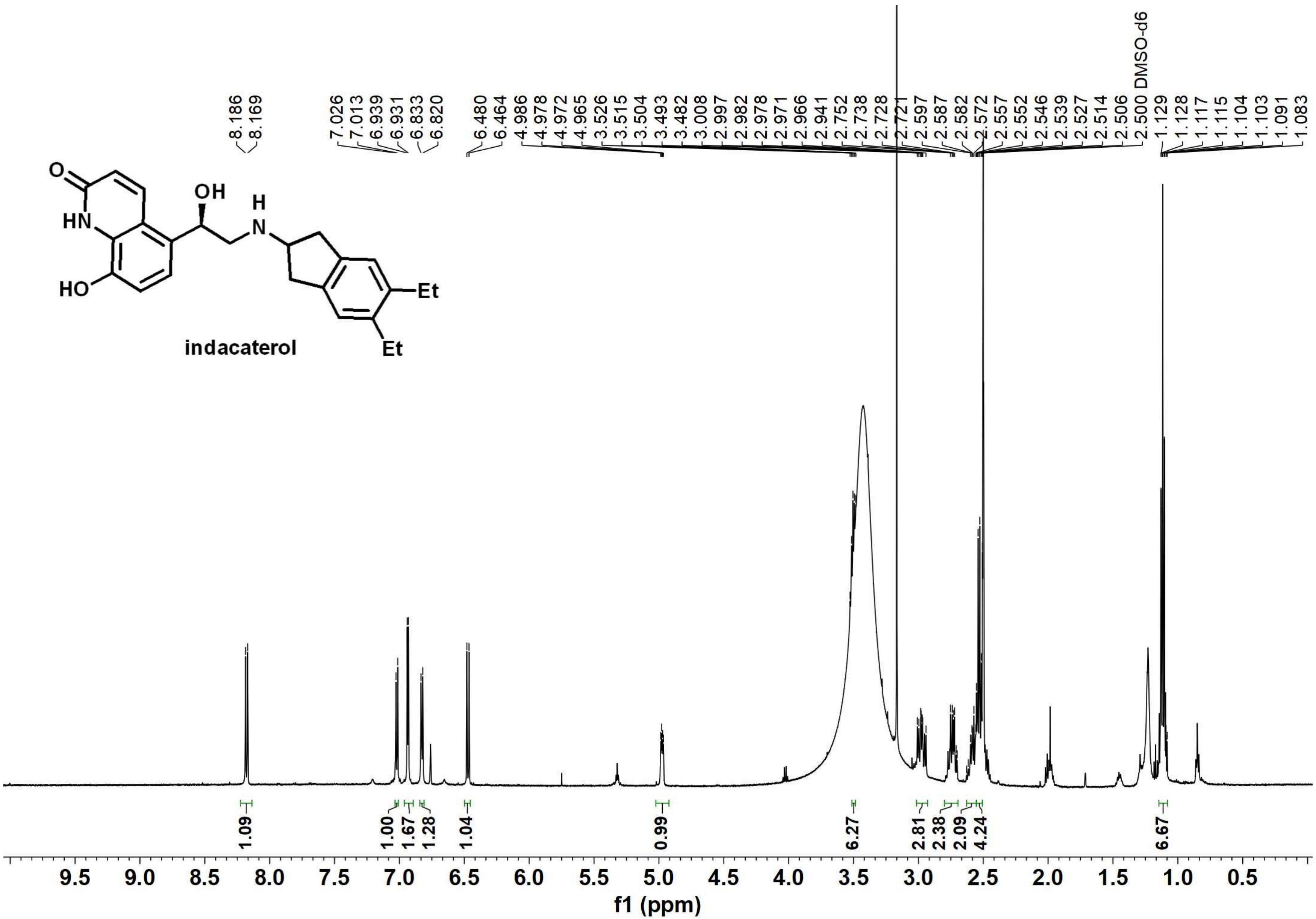

¹H NMR of indacaterol (2) (600 MHz, DMSO-*d6*)

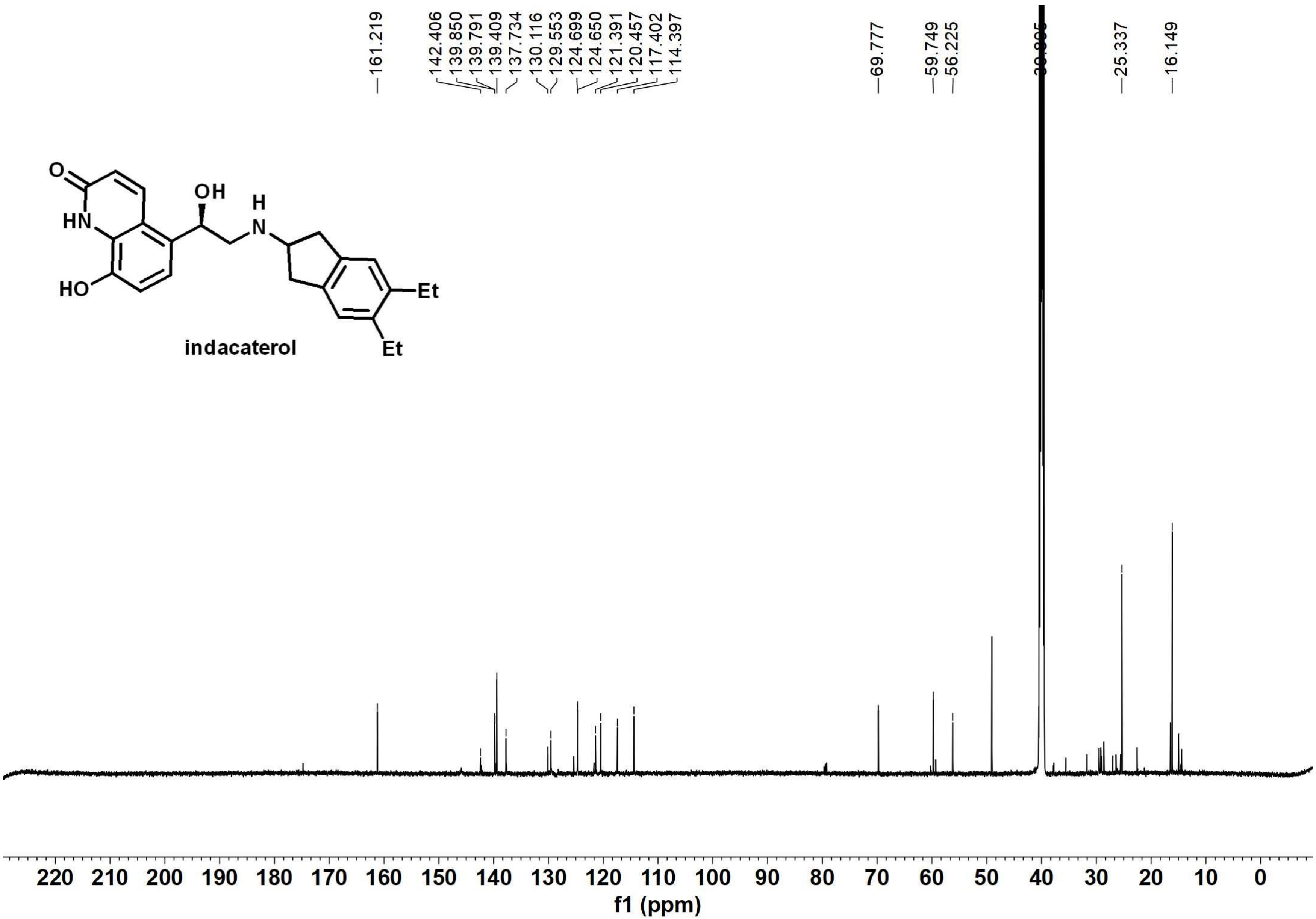

^{13}C NMR of indacaterol (2) (600 MHz, DMSO-*d6*)

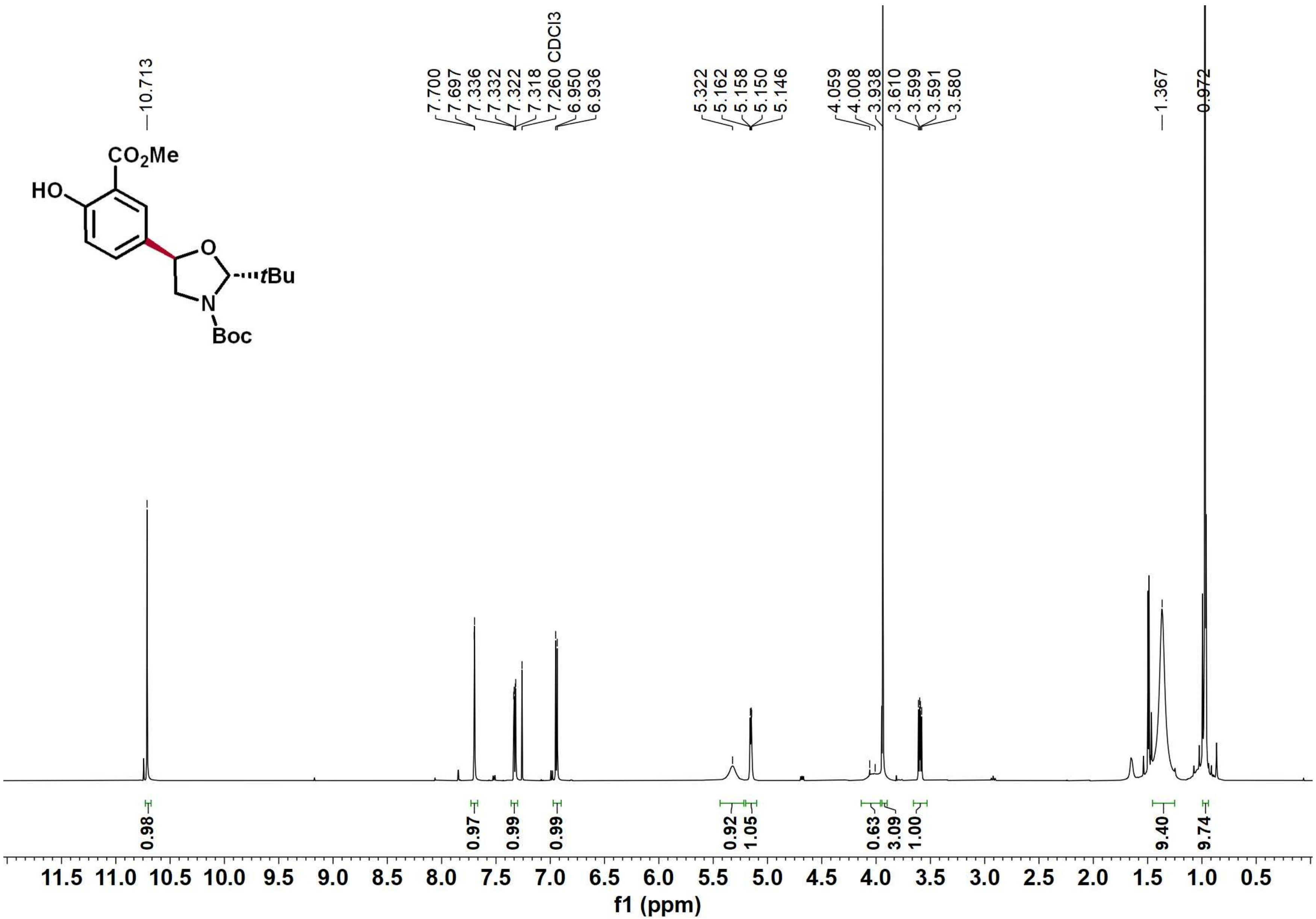

^{1}H NMR of Compound 44 (600 MHz, $CDCl_3$)

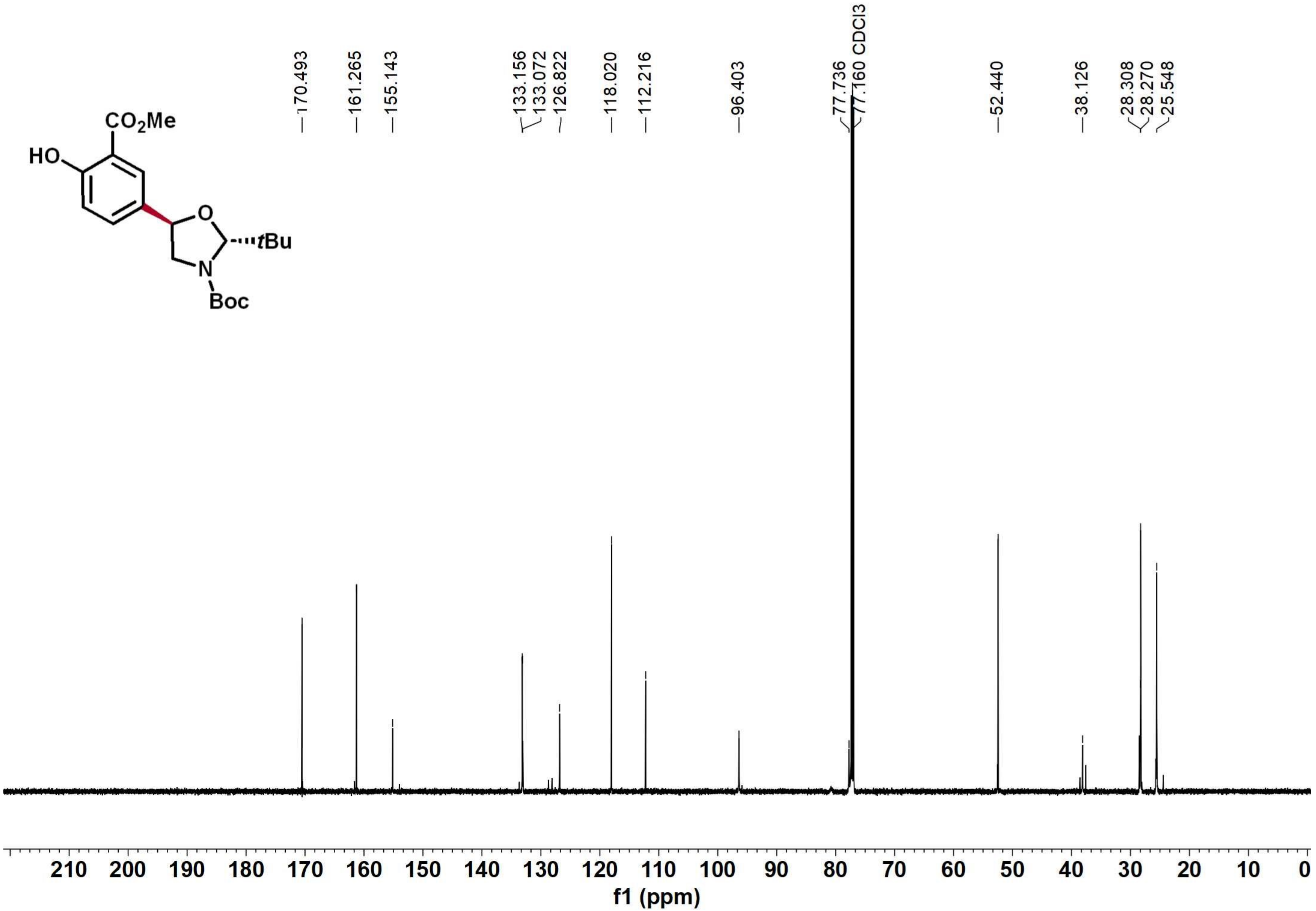

^{13}C NMR of Compound 44 (151 MHz, $CDCl_3$)

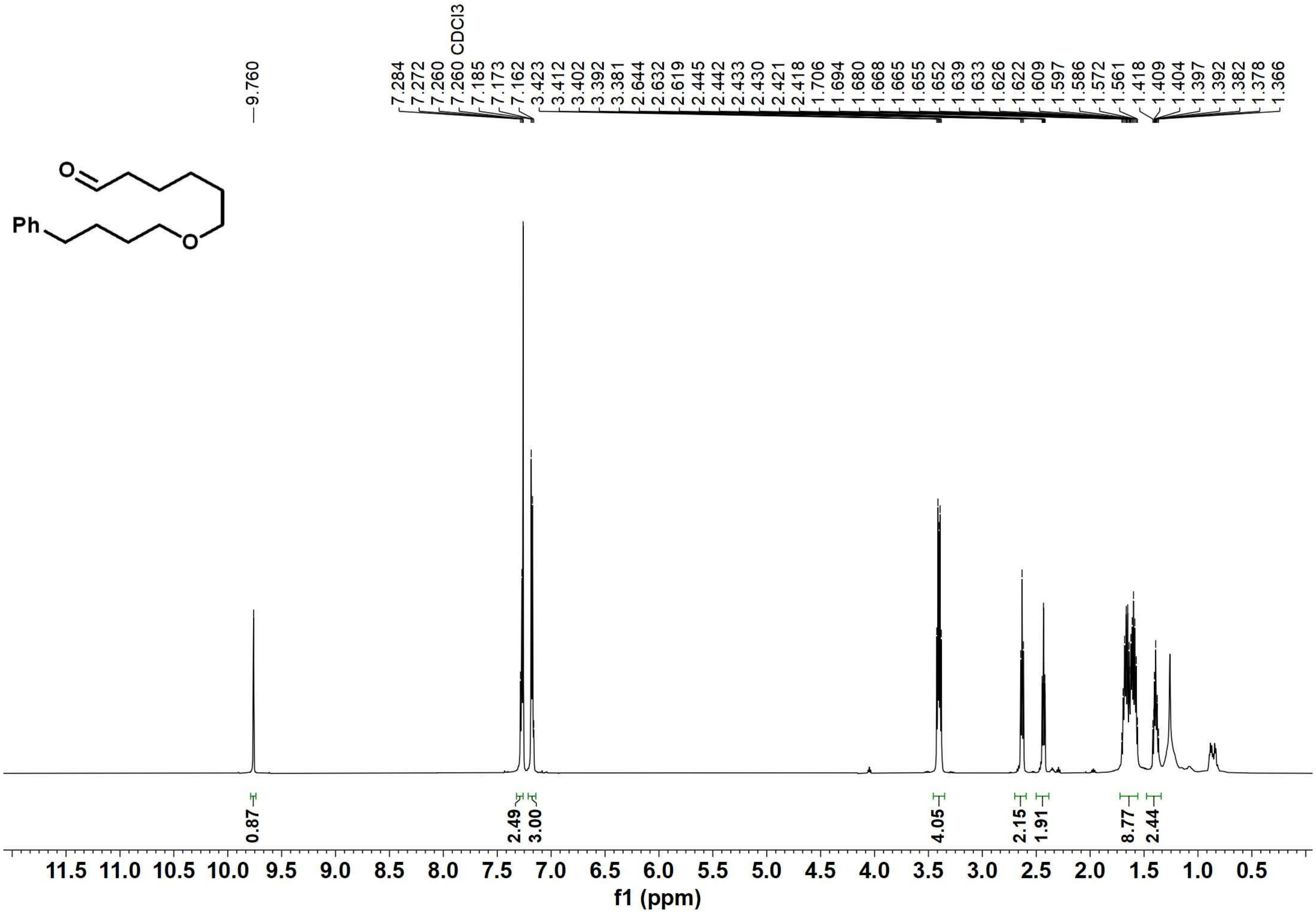

^{1}H NMR of Compound 45 (600 MHz, $CDCl_3$)

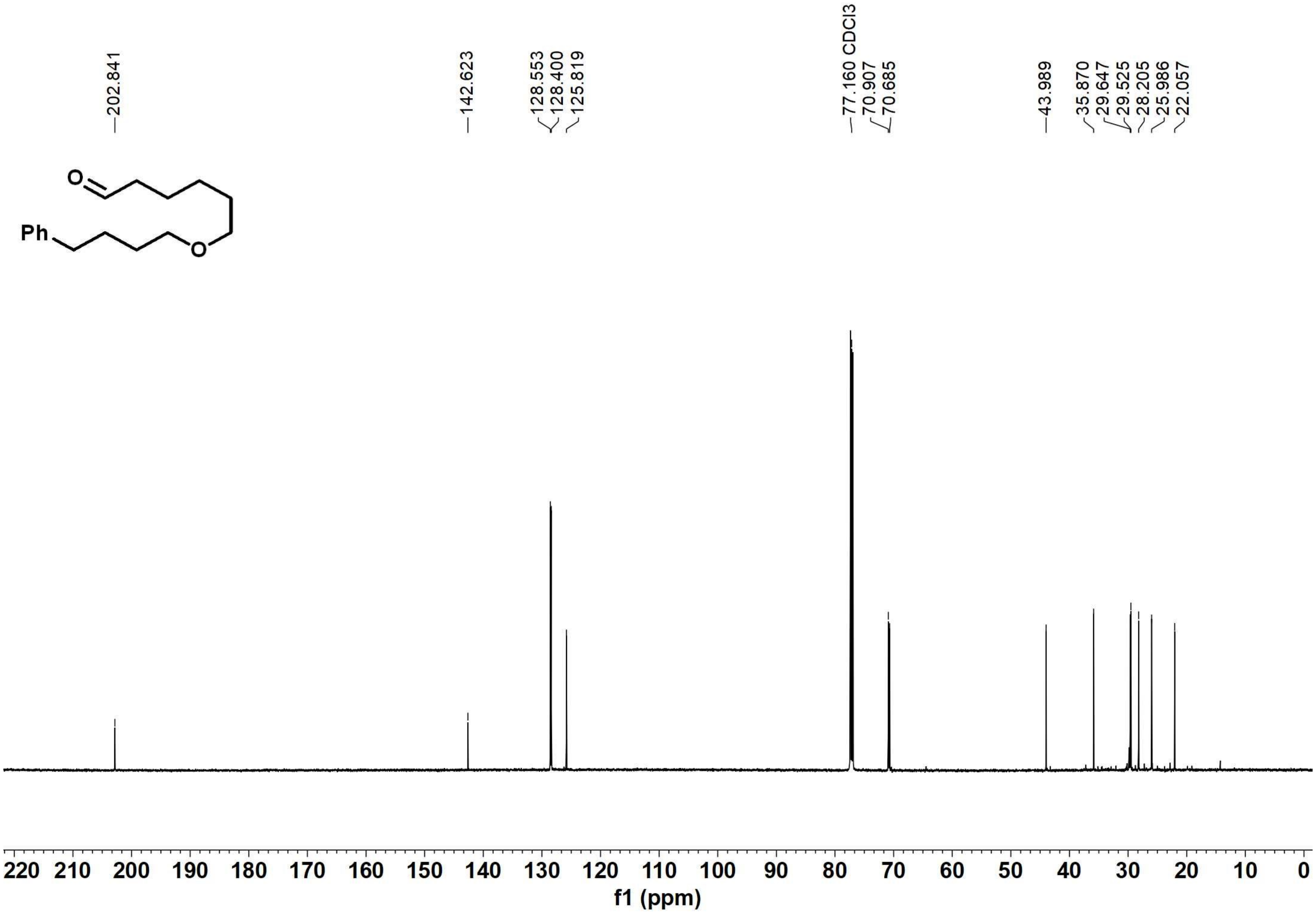

^{13}C NMR of Compound 45 (151 MHz, $CDCl_3$)

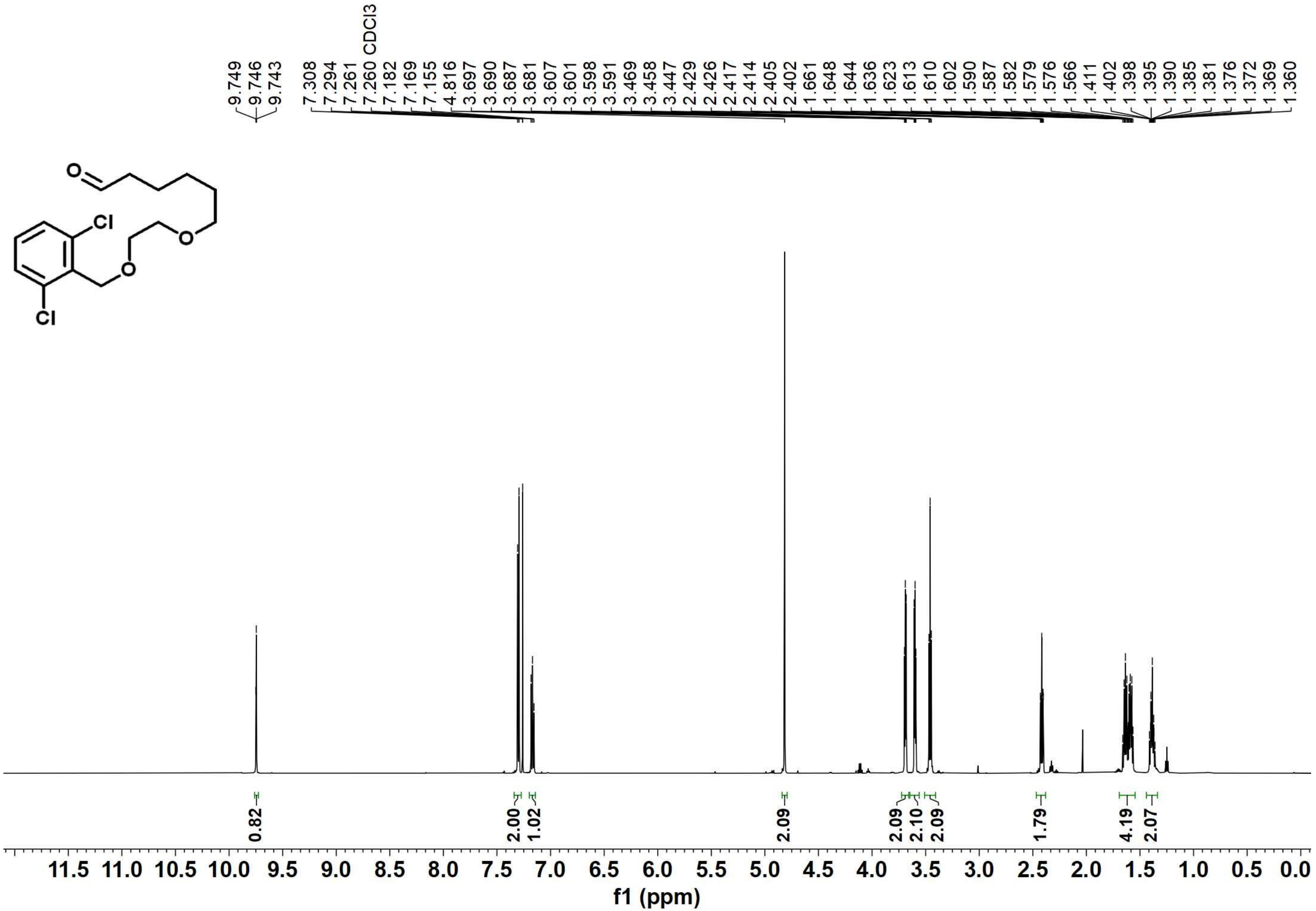

^{1}H NMR of Compound 46 (600 MHz, $CDCl_3$)

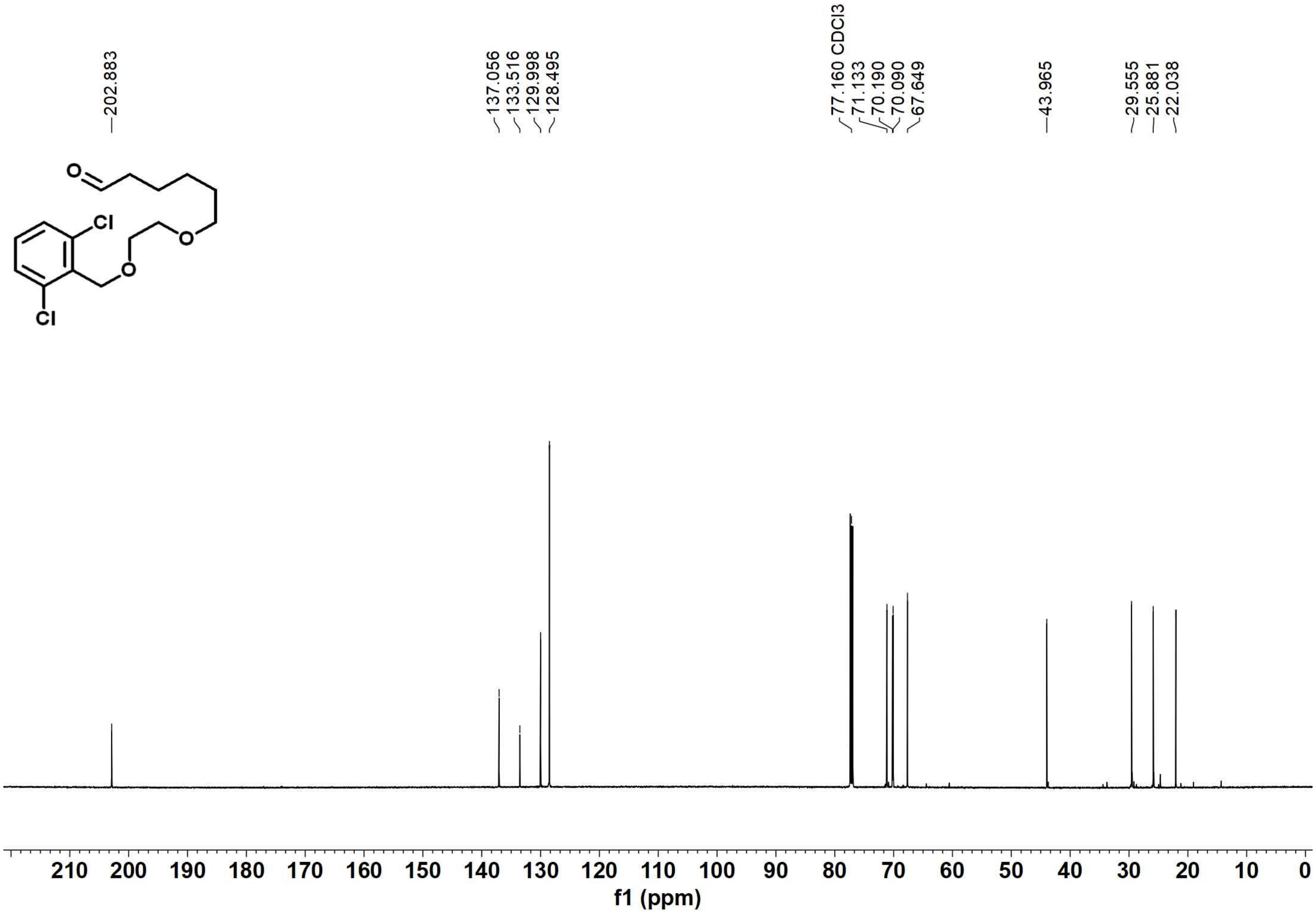

^{13}C NMR of Compound 46 (151 MHz, $CDCl_3$)

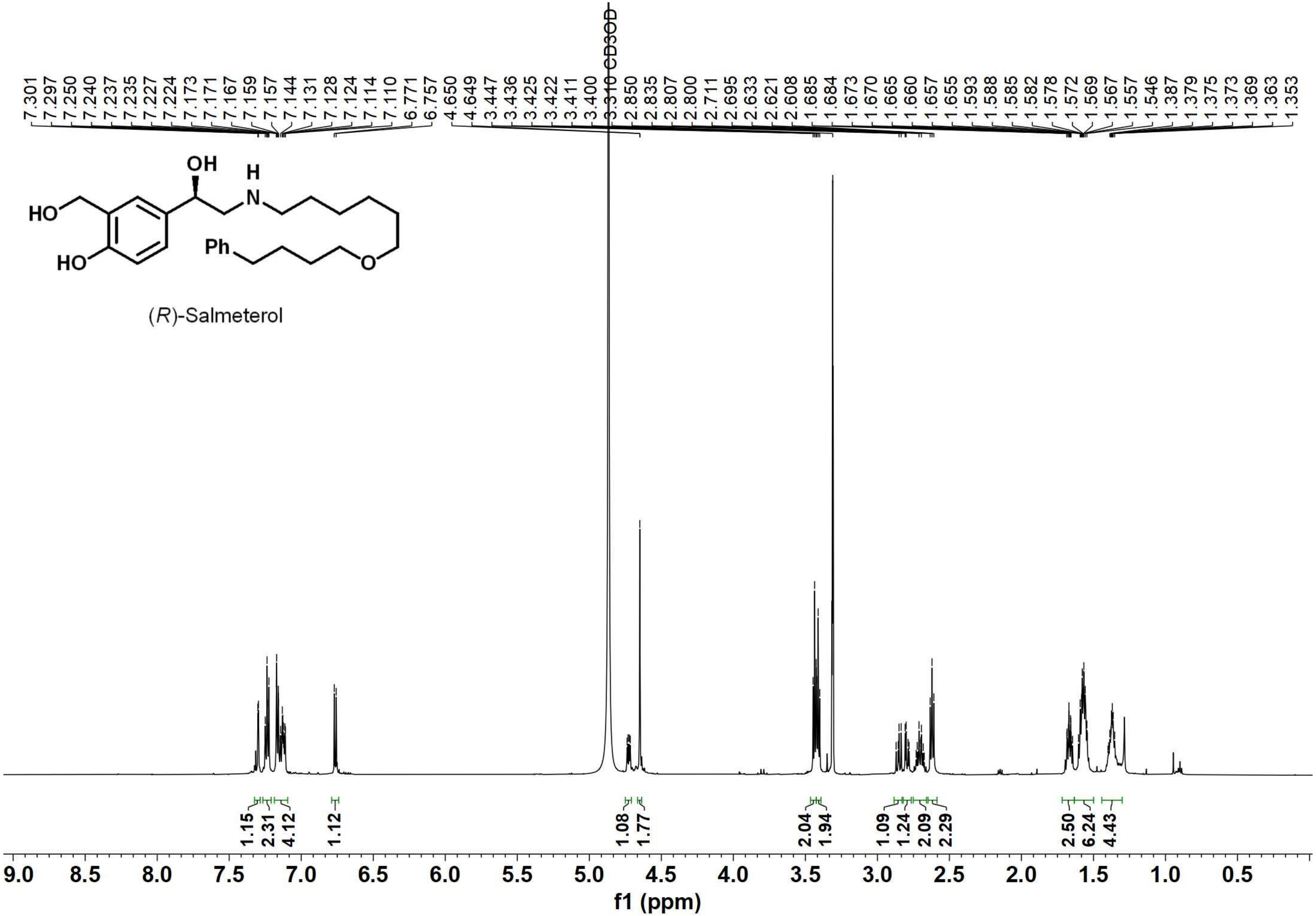

^{1}**H NMR of (*R*)-Salmeterol (3) (600 MHz, CD$_3$OD)**

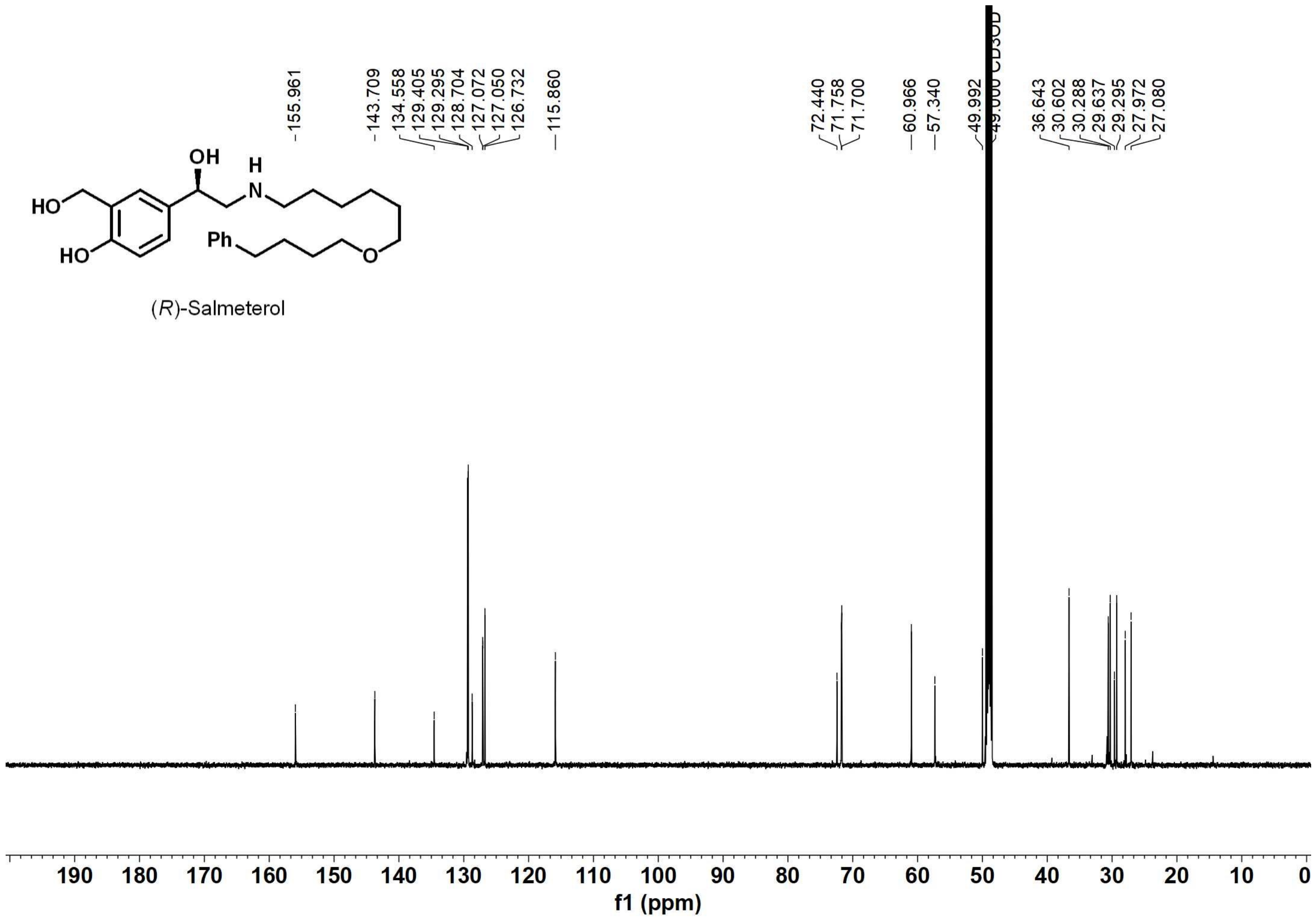

^{13}C NMR of (*R*)-Salmeterol (3) (600 MHz, CD_3OD)

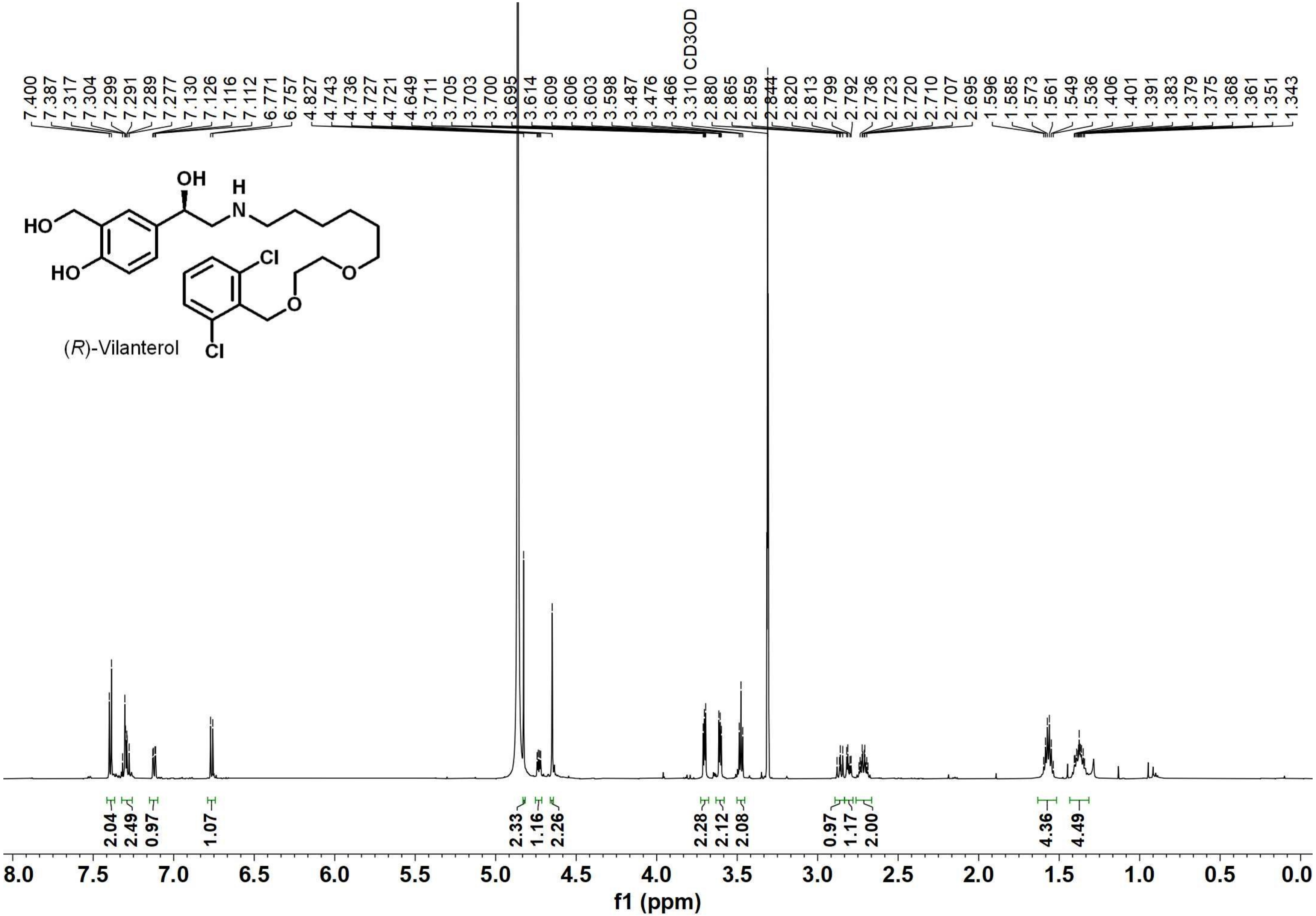

^{1}H NMR of (R)-Vilanterol (3) (600 MHz, CD_3OD)

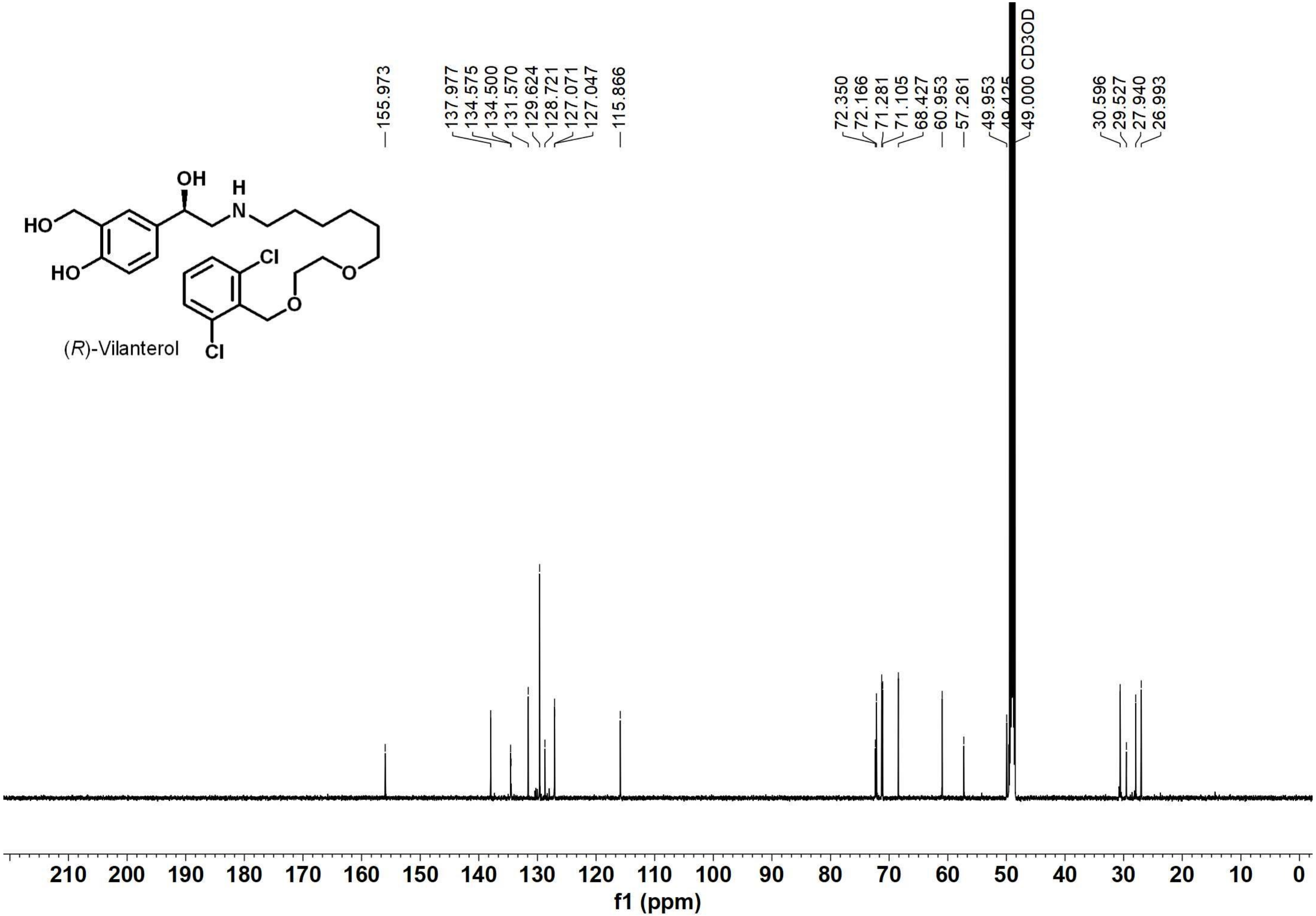

^{13}C NMR of (*R*)-Vilanterol (3) (151 MHz, CD_3OD)
